# Successful Recovery of Nuclear Protein-Coding Genes from Small Insects in Museums Using Illumina Sequencing

**DOI:** 10.1371/journal.pone.0143929

**Published:** 2015-12-30

**Authors:** Kojun Kanda, James M. Pflug, John S. Sproul, Mark A. Dasenko, David R. Maddison

**Affiliations:** 1 Department of Integrative Biology, Oregon State University, Corvallis, Oregon, United States of America; 2 Center for Genome Research and Biocomputing, Oregon State University, Corvallis, Oregon, United States of America; University of California, Berkeley, UNITED STATES

## Abstract

In this paper we explore high-throughput Illumina sequencing of nuclear protein-coding, ribosomal, and mitochondrial genes in small, dried insects stored in natural history collections. We sequenced one tenebrionid beetle and 12 carabid beetles ranging in size from 3.7 to 9.7 mm in length that have been stored in various museums for 4 to 84 years. Although we chose a number of old, small specimens for which we expected low sequence recovery, we successfully recovered at least some low-copy nuclear protein-coding genes from all specimens. For example, in one 56-year-old beetle, 4.4 mm in length, our *de novo* assembly recovered about 63% of approximately 41,900 nucleotides in a target suite of 67 nuclear protein-coding gene fragments, and 70% using a reference-based assembly. Even in the least successfully sequenced carabid specimen, reference-based assembly yielded fragments that were at least 50% of the target length for 34 of 67 nuclear protein-coding gene fragments. Exploration of alternative references for reference-based assembly revealed few signs of bias created by the reference. For all specimens we recovered almost complete copies of ribosomal and mitochondrial genes. We verified the general accuracy of the sequences through comparisons with sequences obtained from PCR and Sanger sequencing, including of conspecific, fresh specimens, and through phylogenetic analysis that tested the placement of sequences in predicted regions. A few possible inaccuracies in the sequences were detected, but these rarely affected the phylogenetic placement of the samples. Although our sample sizes are low, an exploratory regression study suggests that the dominant factor in predicting success at recovering nuclear protein-coding genes is a high number of Illumina reads, with success at PCR of COI and killing by immersion in ethanol being secondary factors; in analyses of only high-read samples, the primary significant explanatory variable was body length, with small beetles being more successfully sequenced.

## Introduction

Natural history collections document the diversity of life on Earth, past and present. They are rich sources of biological discoveries, as well as repositories for vouchers documenting the species studied in previous research. Although traditionally viewed as a resource for taxonomic and systematic research, they are now being utilized across diverse biological disciplines [1–4]. The vast majority of specimens in museums were collected and preserved before the widespread sequencing of DNA, and were not killed or stored using methods designed to maintain DNA. In spite of this, museum specimens, especially those in herbaria and vertebrate collections, are also being used in molecular studies [5–7]. Although many organismal disciplines that rely on natural history collections are transitioning towards a reliance on molecular data, biologists, especially those who work on small organisms such as insects, do not often view museum specimens as a primary source for DNA sequences, perhaps because DNA degradation prevents traditional PCR based methods of sequence recovery [4,8–10].

A number of studies of old DNA from dry, mounted museum insects have documented the use of PCR and Sanger sequencing to obtain short fragments of genes [11–13]. However, these methods often depend on the design of very specific primers and the sequence return relative to the cost investment may be prohibitively low, especially for specimens with highly fragmented DNA. Shotgun sequencing approaches using High-Throughput Sequencing (HTS) have opened the door to sequencing old or ancient DNA, and are becoming more widely accepted among biologists working on larger animals [5,7,14–16]. These methods have been used to sequence ancient DNA from subfossils, including specimens several hundred thousand years in age (e.g., [17,18,19]).

Sequencing old DNA (decades to centuries old) from small insects poses a challenge as their bodies contain lower total quantities of DNA. Although pooling DNA from multiple specimens is possible, it is often undesirable, especially when species boundaries are unclear or when the number of available specimens is low. To date, only a few studies have used HTS to obtain DNA sequences from old insect specimens [20–23]. Two of these studies demonstrated the potential to generate DNA sequences of regions from mitochondrial or ribosomal DNA [20,22], both of which exist in multiple copies per cell. Tin *et al*. [23] recovered RAD-Seq data from museum specimens but for many research studies, specific gene regions are desired. Phylogenetics, population genetics, molecular ecology, comparative genomics, and other fields are increasingly reliant upon accurately sequencing many low-copy regions of the genome. For this reason, we began to explore acquisition of low-copy, nuclear protein-coding genes from small, dried insects in museums.

Our first attempt (briefly described in [21]) was surprisingly successful, and we decided to utilize Illumina HTS on a larger sample of dried museum specimens. Our goal was to use the data obtained in our own research, not to explore the capability of HTS in acquiring nuclear protein-coding genes. However, our results were so encouraging that we present here an initial documentation of the extent and accuracy of HTS in obtaining these low-copy genes. Our hope in so doing is that the incredible genetic resources contained within the world’s museums will be better appreciated, explored more thoroughly, and used more efficiently across biological disciplines.

The museum specimens included in this study span a diversity of ages, preservation methods, and DNA quality. We compare gene recovery between *de novo* assemblies and reference-based assemblies and validate our data by comparison to sequences obtained through PCR and Sanger sequencing, as well as through phylogenetic analyses that test the placement of the HTS sequences. For the specimens we sampled, we recorded detailed specimen histories and measured quantity and quality of their DNA in an attempt to document metrics that might help predict sequencing success of museum specimens. Our results add to the growing body of evidence suggesting that the millions of specimens stored in insect collections should be viewed as a potential source of molecular data, and that these specimens can yield valuable data for even low-copy nuclear protein-coding genes.

## Materials and Methods

### Overview

**Fig 1:**
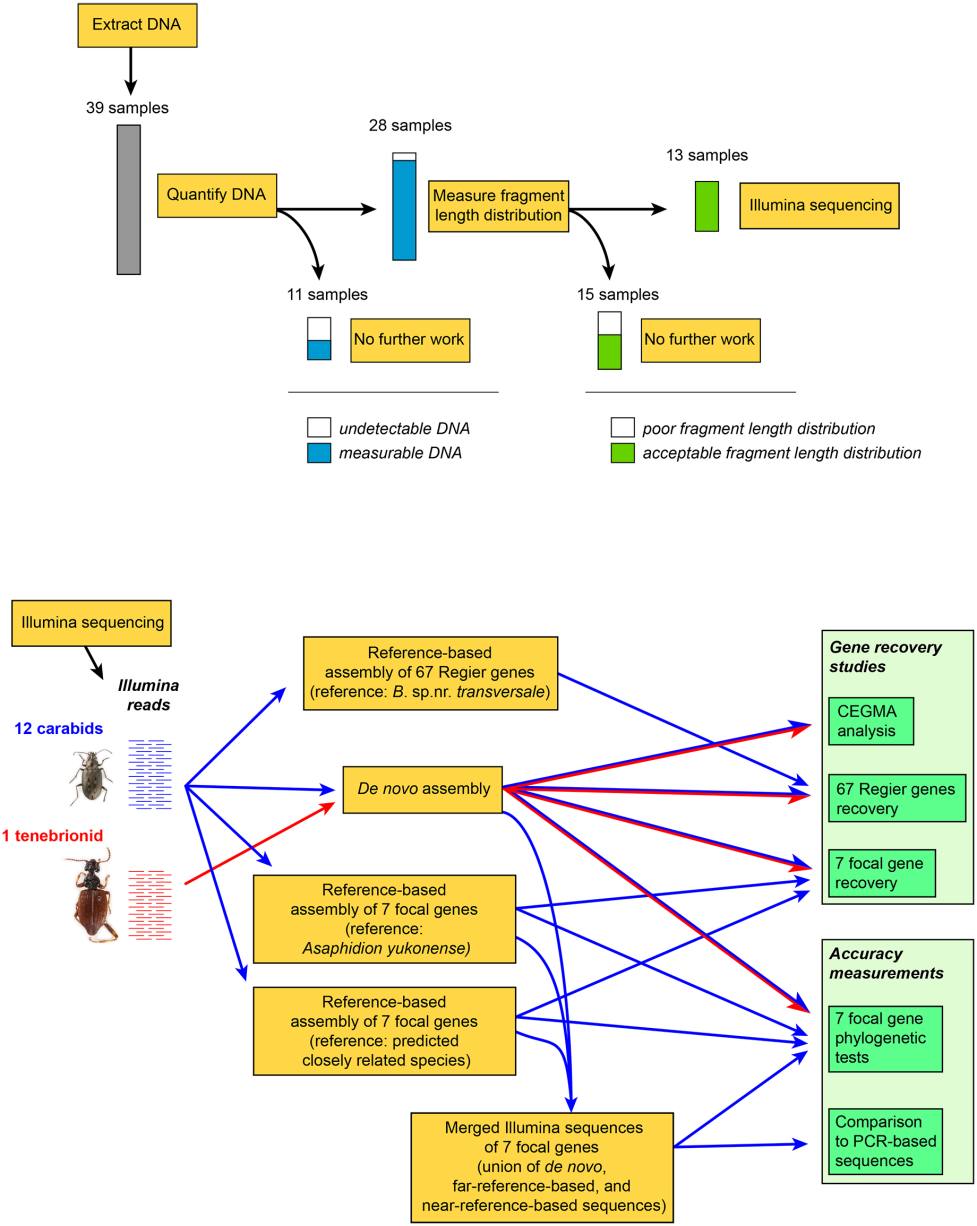
A flow chart providing an overview of our methodological approach.

**Fig 2:**
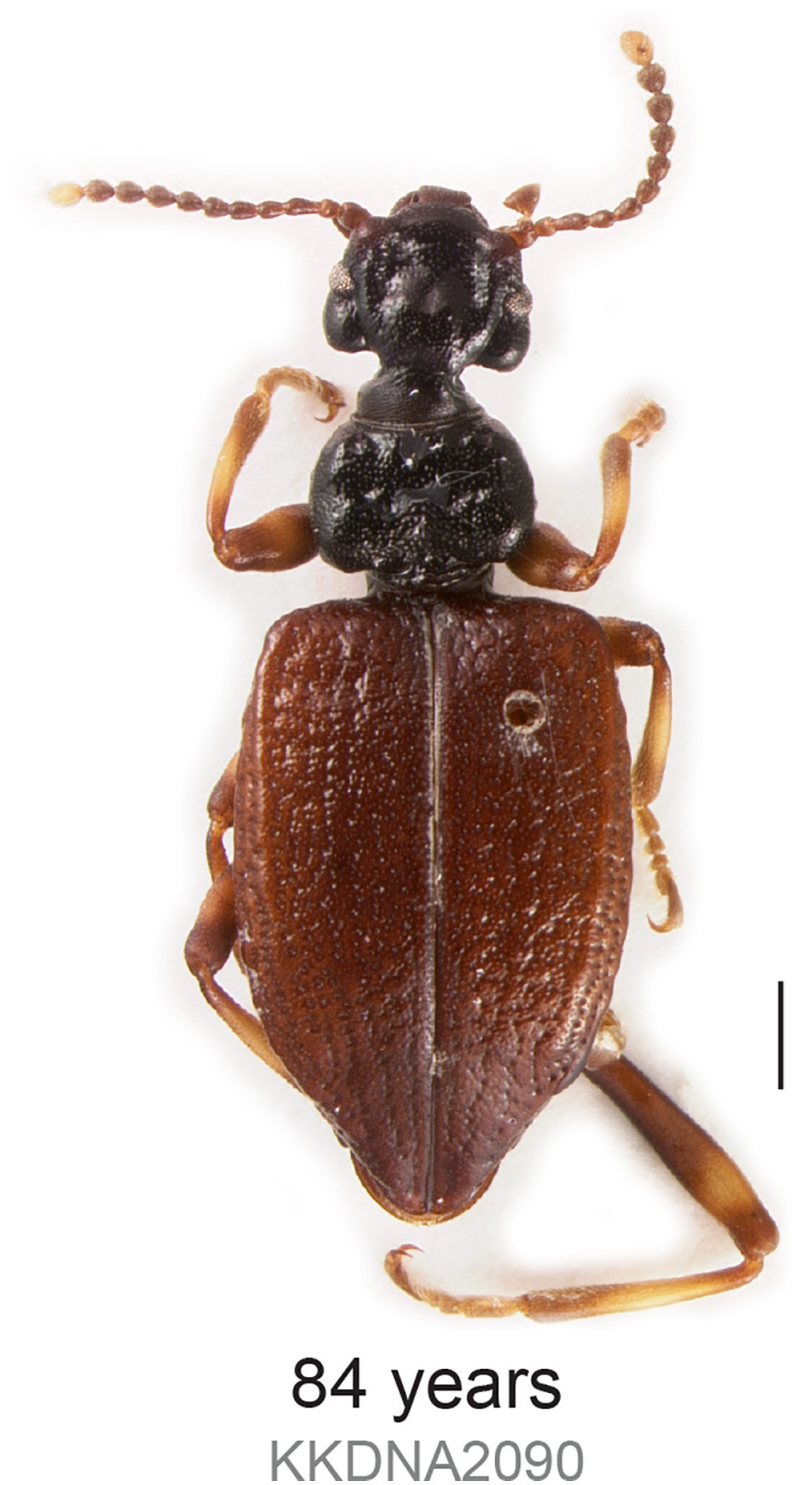
Habitus of Lagriinae n. gen. KK0290. Image taken after DNA extraction. Scale bar is 1 mm.

**Fig 3:**
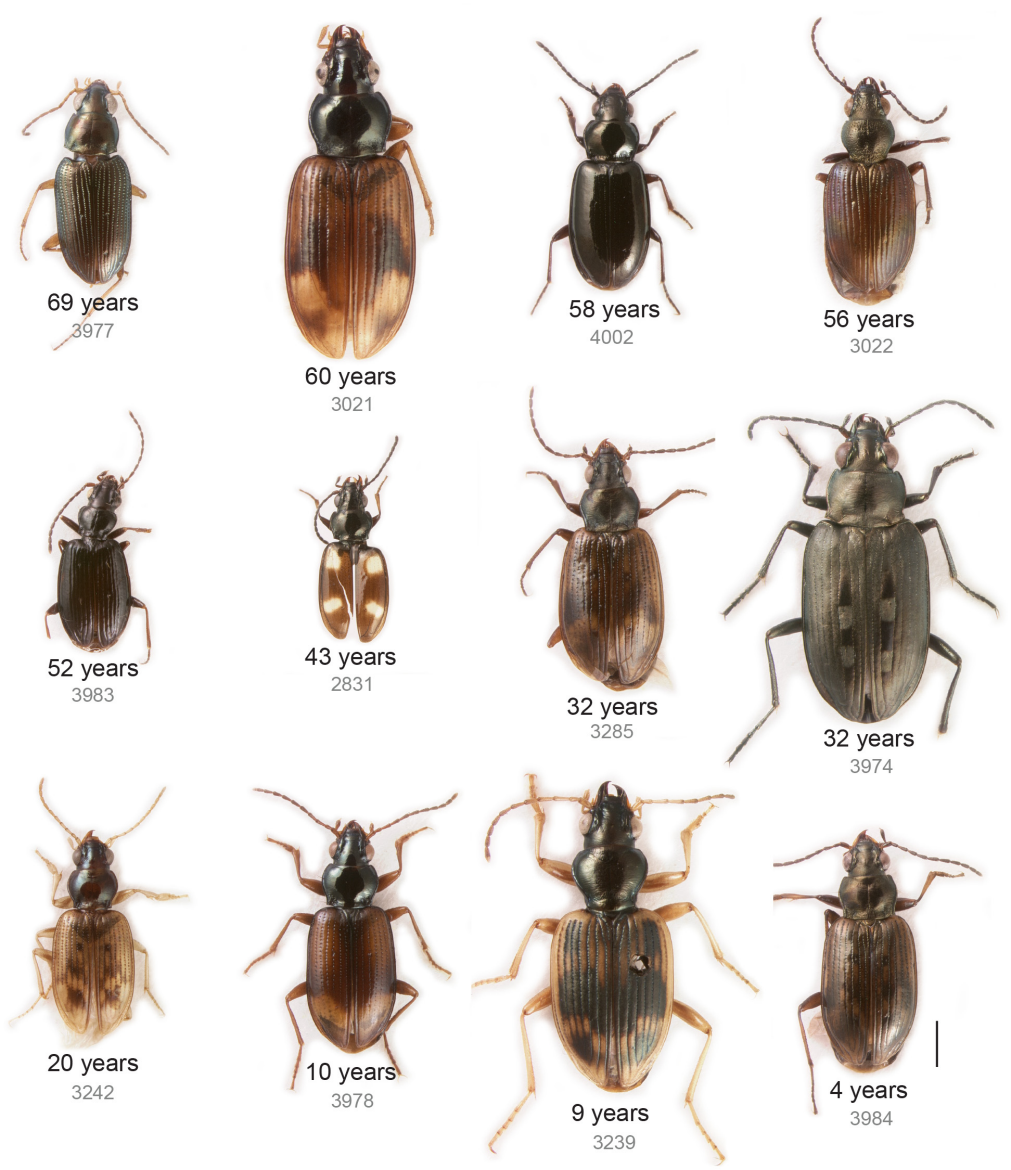
Habitus of HTS carabid specimens. Images taken after DNA extraction. Scale bar is 1 mm.

A flowchart of our methods is provided in Fig 1. We initially extracted DNA from 39 museum specimens from the beetle families Tenebrionidae and Carabidae for potential HTS. In this paper, we use “museum specimen” to refer to specimens in a museum that were preserved dry or in low-concentration ethanol, that is, any specimen killed and stored to preserve exoskeletal characters but not to intentionally preserve DNA. After measuring total DNA and characterizing fragment-length distribution of the extractions, we selected 13 museum specimens for HTS (Figs 2 and 3). We intentionally chose specimens from across the range of DNA quantity and fragment-length distribution that we observed. We also sequenced two specimens (hereafter referred to as “reference specimens”) that had been stored in 95–100% ethanol (which is expected to preserve DNA), to serve as points of comparison. We tested the extent of gene recovery and accuracy of the sequences in three ways.

1. Recovery of core eukaryotic genes (CEGs) using CEGMA [24].
2. Recovery of 67 nuclear protein-coding gene fragments used in the study of Regier *et al*. [25]. For all HTS specimens, we BLASTed our *de novo* assemblies for these gene fragments. For carabids, we also used a partially sequenced genome of *Bembidion* sp. nr. *transversale* to build reference-based assemblies of these gene fragments.
3. Recovery of 7 genes used by KK and DRM in their ongoing work on the phylogeny of Tenebrionidae and Carabidae. These genes were extracted from *de novo* assemblies of our HTS data using BLAST. For the carabids, we also attempted to recover the genes using reference-based assemblies. General accuracy of sequences was validated by incorporating the HTS data in phylogenetic analyses with other carabids or tenebrionids and testing for placement of the HTS sequences in expected clades, as well as by comparison to sequences obtained from conspecifics using PCR and Sanger sequencing

We also attempted to identify characteristics of the museum specimens which could predict success of HTS in sequencing low-copy nuclear genes. Factors explored include age and size of specimen, DNA quantity, DNA fragment-length distribution, preservation history, and ability to sequence short fragments using PCR and Sanger sequencing. In the following sections we provide a detailed account of our methods.

### Museum specimens examined

We considered four specimens representing three species of Tenebrionidae for HTS (Tables 1–3, S1 Table). Three of the specimens are undescribed species in the genus *Chaetyllus* Pascoe 1860 (Tenebrionidae: Lagriinae) and one is an undescribed genus of Lagriinae, which will be referred to as “Lagriinae n. gen.”. The latter specimen, collected in 1929, is the oldest included in this study, and is the only tenebrionid that we eventually chose to sequence using HTS.

**Table 1:** Specimens sequenced using Illumina methods, with details about specimen histories.

Taxon	Sample	Length (mm)	Year	Years death to extraction	Collector	Killing method	Storage before mounting	Relaxed before mounting?	Time from death to mounting	Source
Lagriinae n. gen.	KK0290	9.7	1929	84	PJD	possibly 70% EtOH	?	?	?	PJD1971
*Bembidion subfusum*	3977	4.4	1945	69	PJD	probably 70% EtOH	?	?	?	PJD1971
*B*. sp. nr. *transversale*	3021	6.9	1952	60	BM	probably EtOH	?	?	?	WM
*Lionepha chintimini*	4002	4.3	1956	58	HBL	probably EtOAc	dried	probably	> 1 month	RLeech
*B*. *lachnophoroides*	3022	4.4	1956	56	GEB	probably 95% EtOH	95% EtOH	?	< 1 year	GEB
*Bembidarenas*	3983	3.8	1962	52	PJD	possibly 70% EtOH	?	?	?	PJD1971
*B*. *orion*	2831	3.5	1968	43	KWC	probably EtOH, possibly mixed with benzene and EtOAc	killing fluid	?	probably < 1 week	GCoop
*B*. "Inuvik"	3285	5.0	1981	32	DRM	EtOAc	dried	yes	< 1 year	DRM
*B*. *lapponicum*	3974	6.5	1982	32	DRM	EtOAc	dried	yes	< 1 year	DRM
*B*. "Arica"	3242	4.3	1993	20	RDW	75% EtOH, 5% EtOAc, .25% AA	killing fluid	no	<1 year	RDW
*B*. *cf*. "Desert Spotted"	3978	4.8	2004	10	KK	EtOAc	75% ethanol <24 hours later	no	2 weeks	KK
*B*. *musae*	3239	6.4	2004	9	RDW	75% EtOH, 5% EtOAc, .25% AA	80% EtOH	no	2–4 months	RDW
*B*. "Inuvik"	3984	5.0	2010	4	DSS	100% EtOH	killing fluid	no	7–8 months	DSS
*B*. *orion*	3079	3.0	2012	2	DRM	100% EtOH	-	-	-	-
*B*. sp. nr. *transversale*	3205	7.1	2012	0	DRM	100% EtOH	-	-	-	-

Specimens sequenced using Illumina methods, with details about specimen histories. All specimens were stored as dried specimens in museum drawers, except for 3079 and 3205, which were preserved in 100% ethanol. Specimen 3984 is preserved in the University of Alaska Museum, with voucher code UAM:Ento: 167080. **Length**: approximate body length of specimen. **Year**: year the specimen was collected. **Abbreviations for Collectors**: BM: Borys Malkin, D&L: J. Decelle, N. & J. Leleup, DHK: David H. Kavanaugh, DRM: David R. Maddison, DSS: Derek S. Sikes, EAM: E.A.Martinko, FCF: F.C. French, FGA: Fred G. Andrews, GEB: George E. Ball, HBL: Hugh B. Leech, HG: Henri Goulet, JA: Joachim Adis, JGE: J. Gordon Edwards, JWG: J.W. Green, KK: Kojun Kanda, KR: Keith Roney, KWC: Kenneth W. Cooper, LHH: Lee H. Herman, LRD: Lloyd R. Davis, Jr., MHH: Melville H. Hatch, PHA: Paul H. Arnaud, PJD: Philip J. Darlington, RDW: Robert D. Ward, RSA: Robert S. Anderson. **Abbreviations for killing substance**: EtOH: ethanol, EtOAc: ethyl acetate, AA: Acetic Acid, CN: cyanide. **Sources of information about killing and preserving methods**: If the initials are the same as under Collector, then the collector himself provided information via personal communication in 2013–2015. Otherwise, the following people provided information about specimens based upon personal experience with the collector: CMR: Rod Crawford, David McCorkle, Loren Russell (graduate students of H.B.Leech), GCoop: Geoff Cooper, son of K.W. Cooper, GCoul: Geoff Coulon, based in part on Leleup’s field notebooks; RLeech: Robin Leech, son of collector; RLesch: Richard Leschen, colleague of collector; TLE: Terry L. Erwin, colleague of collector; WM: Werner Marggi, colleague of collector. Treatment of Phil Darlington’s specimens was inferred from [26].

**Table 2:** Museum specimens that were assessed with a Qubit and Bioanalyzer but not Illumina sequenced, with details about specimen histories.

Taxon	Sample	Length (mm)	Year	Years death to extraction	Collector	Killing method	Storage before mounting	Relaxed before mounting?	Time from death to mounting	Source
*Bembidion subfusum*	2494	4.3	1945	64	PJD	probably 70% EtOH	?	?	?	PJD1971
*B*. *subfusum*	1955	4.7	1945	60	PJD	probably 70% EtOH	?	?	?	PJD1971
*Bembidarenas reicheellum*	3973	3.4	1962	52	PJD	possibly 70% EtOH	?	?	?	PJD1971
*Apteromimus platyderoides*	3959	4.4	1967	46	D&L	EtOH possibly 70%	?	?	>2 months	GCoul
*Pseudophilochthus nubigena*	3957	6.0	1967	46	D&L	EtOH possibly 70%	?	?	>2 months	GCoul
*Tachysbembix* sp.	3908	3.5	1974	39	LHH	70% EtOH	killing fluid	no	<1 year	LHH
*Moirainpa amazona*	3907	1.3	1976	37	JA	70% EtOH or picric acid	?	?	?	TLE
*B*. "Clearwater"	2907	5.3	1977	35	LRD	99% isopropanol	killing fluid	no	<1 year	LRD
*B*. *tencenti*	3286	3.9	1986	27	KR	EtOAc	dried	yes	< 1year	KR
*B*. "Arica"	3975	4.6	1993	21	RDW	75% EtOH, 5% EtOAc, .25% AA	killing fluid	no	4–6 months	RDW
*B*. sp. nr. *germainianum*	3976	5.4	1994	20	RDW	75% EtOH, 5% EtOAc, .25% AA	95% EtOH	no	<1 year	RDW
*B*. *(Asioperyphus)* sp.	4003	5.6	1996	18	PHA	EtOAc	dried	yes	< 1 year	PHA
*Chaetyllus* n. sp. 1	KK0280	4.2	1998	16	TLE	75% EtOH	-	-	-	TLE
*Chaetyllus* n. sp. 11	KK0278	4.5	2001	13	RSA	80% EtOH	80% EtOH	no	<4 months	RSA
*B*. *nesophilum*	3240	4.3	2004	9	RDW	75% EtOH, 5% EtOAc, .25% AA	80% EtOH	no	<2 months	RDW

All specimens were stored as dried specimens in museum drawers, except for the KK0278, which was stored in low-concentration ethanol (less than 75%). For abbreviations, see caption for Table 1.

**Table 3:** Museum specimens assessed with a Qubit but not with a Bioanalyzer or Illumina sequenced, with details about specimen histories.

Taxon	Sample	Length (mm)	Year	Years death to extraction	Collector	Killing method	Storage before mounting	Relaxed before mounting?	Time from death to mounting	Source
*Bembidion* "Kenosha Pass"	4004	4.6	1939	75	JWG	?	?	?	?	-
*B*. *sarpedon*	2463	6.2	1937	72	MHH	CN or 70% EtOH	?	?	?	CMR
*Lionepha casta*	4005	4.1	1952	62	FCF	?	?	?	?	
*B*. *(Notaphus)* "Sinaloa"	3971	4.6	1962	52	GEB	EtOAc	dried	yes	<4 months	GEB
*B*. "Talus"	4006	4.9	1963	51	JGE	CN	?	?	?	TLE
*B*. *(Notaphus)* "SLP"	3972	5.2	1965	49	GEB	EtOAc	dried	yes	<2 months	GEB
*Pseudophilochthus rufosuffusum*	3960	2.4	1967	46	D&L	EtOH possibly 70% EtOH	?	?	>2 months	GCoul
*B*. *rufinum*	4007	5.1	1970	44	DHK&HG	EtOAc	dried	yes	<1 year	DHK
*B*. "Red River"	4008	4.7	1971	43	DHK&AM	EtOAc	dried	yes	<1 year	DHK
*B*. *orion*	2826	3.5	1975	36	FGA	100% isopropanol	fresh 100% isopropanol < 24 hours later	?	?	FGA
*Chaetyllus* n. sp. 1	KK0285	4.4	1990	24	TLE	75% EtOH	<75% EtOH	no	<1 year	TLE

All specimens were stored as dried specimens in museum drawers. For abbreviations, see caption for Table 1.

We considered 35 specimens representing 30 species of carabids for HTS (Tables 1–3, S1 Table). We selected these specimens based primarily on their relevance to other projects on the phylogeny of the carabid supertribe Trechitae being conducted by DRM. All but one of the species are members of the subtribe Bembidiina; the exception is a member of *Bembidarenas*, a genus considered *incertae sedis* at the tribal level [27]. Some of the species we studied represent undescribed species or species whose names are not yet known; for these we use informal names.

The specimens we examined varied in size, from 1.3 to 9.7 mm in length (Tables 1–3). Body length of specimens was measured in Microvision's Cartograph software connected to a Leica Z6 lens and JVC KY-F75U camera.

All but one of the museum specimens had been previously mounted, dried, and stored in insect drawers. The exception was *Chaetyllus* n. sp. 1 KK0280, which was stored in lower-concentration ethanol (75% or less) until extraction. When multiple specimens of a taxon were available, we selected the cleanest specimen, with preference given to those with paler (light gray) eyes and paler legs, as this suggests substances such as fats that might darken the eyes have been cleaned out with a preservative such as ethanol. The museum specimens have varied histories (Tables 1–3). They were collected between 1929 and 2010, and their DNA was extracted between 4 and 84 years after death. Details about specimen treatment (including killing method and storage) were sought for most specimens by querying the collector, or, if the collector is no longer living, from a colleague of the collector, as described in Tables 1–3. The exceptions were specimens collected by P.J. Darlington, Jr.; for these specimens, details were inferred from Darlington [26].

Museums in which specimens have been stored include the following institutions (listed in alphabetical order of their codens).

BYU Monte L. Bean Life Science Museum, Brigham Young University, Provo
CAS California Academy of Sciences, San Francisco
CMNH Carnegie Museum of Natural History, Pittsburgh
DRM David R. Maddison collection, Corvallis, Oregon
EMEC Essig Museum Entomology Collection, University of California, Berkeley
KK Kojun Kanda collection, Corvallis, Oregon
KWC Kenneth W. Cooper collection, Riverside, California (now at California Department of Food and Agriculture, Sacramento)
MCZ Museum of Comparative Zoology, Harvard University, Cambridge
MRCA Musée Royal de l'Afrique Centrale, Tervuren
OSAC Oregon State Arthropod Collection, Oregon State University, Corvallis
SEMC Biodiversity Institute, University of Kansas, Lawrence
SMNS Stuttgart State Museum of Natural History
UAM University of Alaska, Museum of the North, Fairbanks
UAIC University of Arizona Insect Collection, Tucson
UASM University of Alberta, E.H. Strickland Entomological Museum, Edmonton
USNM National Museum of Natural History, Smithsonian Institution, Washington, DC

Vouchers will be deposited either within their original repository (see S1 Table), or in OSAC or KK. All material collected by the authors were legally acquired. To the best of our knowledge, this statement holds true for material borrowed from museums, though it is impossible to confirm this for all specimens.

### DNA extractions

We extracted all but four museum specimens in a clean room designed to minimize contamination from non-target DNA and PCR products; the exceptions are described below. The clean room is kept at a positive pressure to ensure only outward airflow, and is separated by a large room from the only room in the lab containing PCR products. Dedicated dissection and extraction supplies such as forceps, pipettes, microcentrifuge, and reagents are kept in the room, and all extractions were performed under a laminar flow hood, which was sterilized with UV before each use. Strict lab protocols limiting worker access to the extraction room were in place to further reduce the risk of PCR and fresh genomic DNA contamination in the clean room.

Prior to extraction, we removed specimens from their mounts. All carabids were glued to paper points or cards. Tenebrionids were either mounted on an insect pin, or glued to a paper point or card. We removed pointed and card-mounted specimens from their mounts by gently prying them loose from the dried glue or briefly soaking both the point and specimen in warm Qiagen ATL buffer. We removed pinned specimens by clipping the pin near the body of the specimen and gently wiggling it free. For most specimens, DNA was extracted from the entire body (S1 Table). All specimens were separated into two or more pieces (in general by separating the abdomen from the rest of the body) to allow for better penetration of reagents during extraction, but no specimens were ground, thus preserving exoskeletal structures.

We extracted DNA from non-museum specimens in a standard molecular lab outside the clean room. Four museum specimens (1955, 2463, 2494, and KK0280) were also extracted in this lab space. We extracted DNA from all specimens with DNeasy Blood and Tissue kits (Qiagen) following the manufacturer's specified protocol.

### Assessing DNA quality of museum and reference specimens

We assessed DNA quality using three measurements: (1) total DNA content, (2) distribution of DNA fragment lengths, and (3) success at PCR amplification.

We measured total DNA in each of the 41 extractions listed in Tables 4–6 using a Qubit Fluorometer (Life Technologies) with a Quant-iT dsDNA HS Assay Kit. We measured the fragment length distribution for 28 of the extractions (those museum specimens listed in Tables 4 and 5) with a 2100 Bioanalyzer (Agilent Technologies) using the High Sensitivity DNA Analysis Kit and 1 μl of sample. Extractions containing more than 10 ng / μl of DNA were diluted before bioanalysis. The 11 extractions listed in Table 6 were not bioanalyzed. These included mostly specimens with DNA concentrations that were too low to detect on the Qubit using 1 μl of sample; the only specimens with similarly low DNA that were analyzed were *Moirainpa amazona* 3908 and *Chaetyllus* n. sp. 1 KK0280, which we judged of enough importance to our work to warrant the expense. Five samples that did contain measurable DNA (those listed in Table 6 with total DNA greater than 1 ng) were also not analyzed further for financial reasons; however, we have no reason to believe that they contain poor-quality DNA.

**Table 4:** Quality and quantity of DNA for specimens sequenced using Illumina methods.

Taxon	Sample	Total DNA (ng)	Modal fragment (bases)	DNA quality score
Lagriinae n. gen.	KK0290	1700	60	1
*Bembidion subfusum*	3977	41.7	60	1
*Bembidion* sp. nr. *transversale*	3021	164	120	2
*Lionepha chintimini*	4002	246	220	3
*Bembidion lachnophoroides*	3022	9.9	80	1
*Bembidarenas*	3983	53.2	100	1
*Bembidion orion*	2831	83.9	200	3
*Bembidion* “Inuvik”	3285	168	50	2
*Bembidion lapponicum*	3974	749	250	4
*Bembidion* “Arica”	3242	539	150	4
*Bembidion* cf. “Desert Spotted”	3978	412	260	4
*Bembidion musae*	3239	3880	500	5
*Bembidion* “Inuvik”	3984	3300	>9,000	5
*Bembidion orion*	3079	146	-^1^	6
*Bembidion* sp. nr. *transversale*	3205	413	-^1^	6

**Total DNA**: calculated by multiplying concentrations measured with Qubit 2.0 Fluorometer (Life Technologies) with total volume of extraction. Extraction volume varied between specimens. **Modal fragment**: Most abundant fragment length in extractions as measured using a 2100 Bioanalyzer (Agilent). “-” indicates samples that were not run on the Bioanalyzer. **DNA quality score**: the score assigned based on total DNA and distribution of fragment lengths measured using a Bioanalyzer. See main text for an explanation of score values.

^1^ These two extractions were from specimens preserved specifically for DNA study so it was presumed that their modal fragment lengths would fall outside of the measurable range using the Bioanalyzer.

**Table 5:** Quality and quantity of DNA for specimens assessed with a Qubit and with a Bioanalyzer but not Illumina sequenced.

Taxon	Sample	Total DNA (ng)	Modal fragment (bases)	DNA quality score
*Bembidion subfusum*	2494	9	X	0
*Bembidion subfusum*	1955	199	X^1^	-^1^
*Bembidarenas reicheellum*	3973	22.4	120	1
*Apteromimus platyderoides*	3959	217	190	3
*Pseudophilochthus nubigena*	3957	745	190	4
*Tachysbembix* sp.	3908	42.7	140	2
*Moirainpa amazona*	3907	<0.045	X	0
*Bembidion* "Clearwater"	2907	437	>9,000	4
*Bembidion tencenti*	3286	14.1	X	0
*Bembidion* "Arica"	3975	131	120	2
*Bembidion* sp. nr. *germainianum*	3976	90.9	120	2
*Bembidion (Asioperyphus)* sp.	4003	169	160	2
*Chaetyllus* n. sp. 1	KK0280	<0.06	X	0
*Chaetyllus* n. sp. 11	KK0278	17.1	X	0
*Bembidion nesophilum*	3240	2270	5300	5

“X” indicates samples in which the modal fragment size could not be determined. For additional explanation, see caption for Table 4.

^1^ Extraction was run on a Bioanalyzer, but plot showed two distinct size peaks, one at 45 bases and another at 810 bases. We hypothesize that the larger peak may correspond to some fungal or bacterial contamination after the specimen had been mounted, however we did not sequence this extraction. A bioanalysis score was not assigned for this extraction.

**Table 6:** Quantity of DNA for specimens that assessed with a Qubit but not with a Bioanalyzer or Illumina sequenced.

Taxon	Sample	Total DNA (ng)
*Bembidion* "Kenosha Pass"	4004	<0.060
*Bembidion sarpedon*	2463	30.7
*Lionepha casta*	4005	<0.061
*Bembidion (Notaphus)* "Sinaloa"	3971	<0.049
*Bembidion* "Talus"	4006	104
*Bembidion (Notaphus)* "SLP"	3972	<0.049
*Pseudophilochthus rufosuffusum*	3960	<0.048
*Bembidion rufinum*	4007	224
*Bembidion* "Red River"	4008	<0.059
*Bembidion orion*	2826	26.1
*Chaetyllus* n. sp. 1	KK0285	137

For additional explanation, see caption for Table 4.

**Fig 4:**
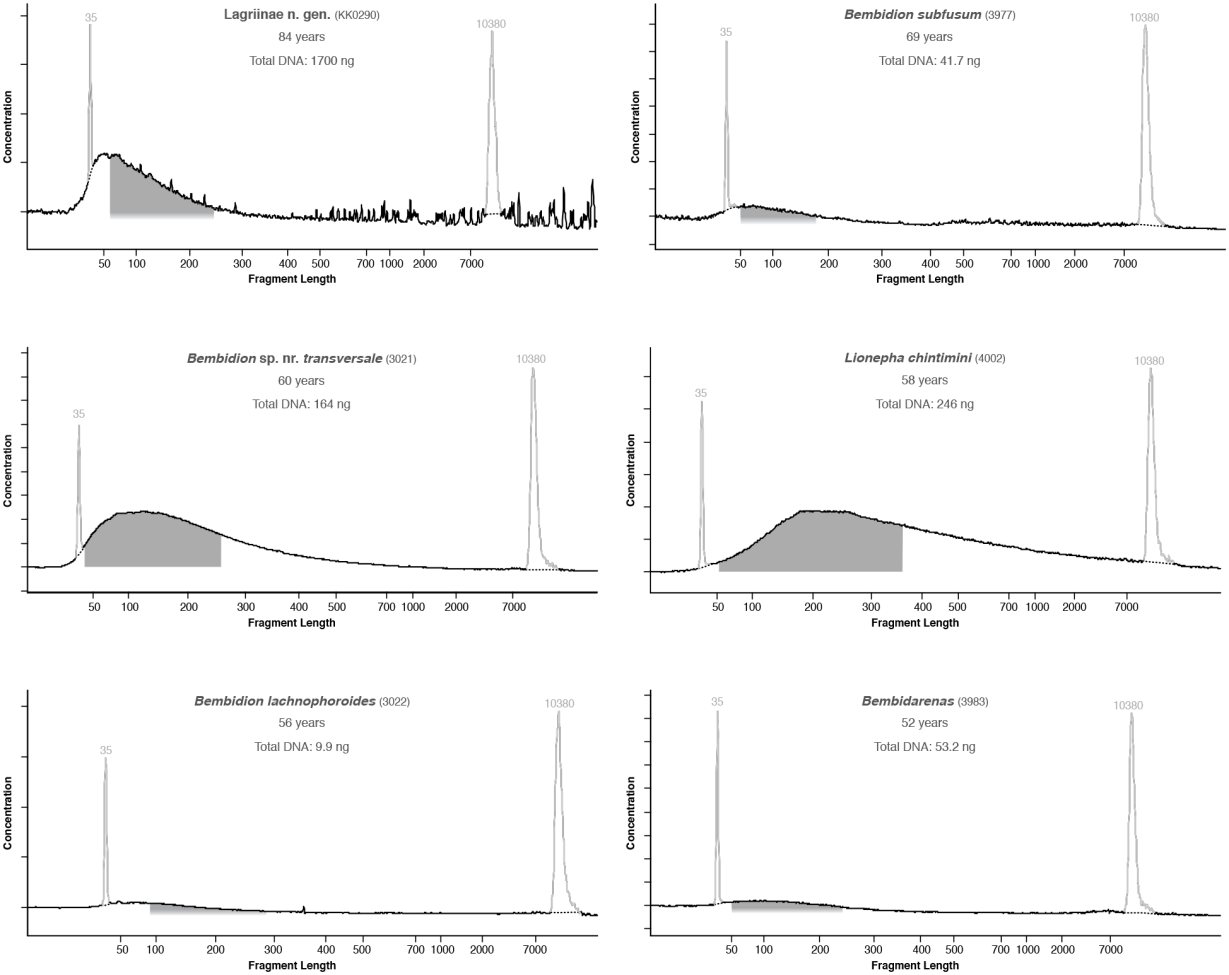
Electropherograms of DNA extracted from older museum specimens that were subsequently used in library preparation. Pale spikes at 35 and 10380 bases represent standards included in each analysis. Dark shaded regions, when present, correspond to range of fragments that were selected and sequenced on the HiSeq 2000.

**Fig 5:**
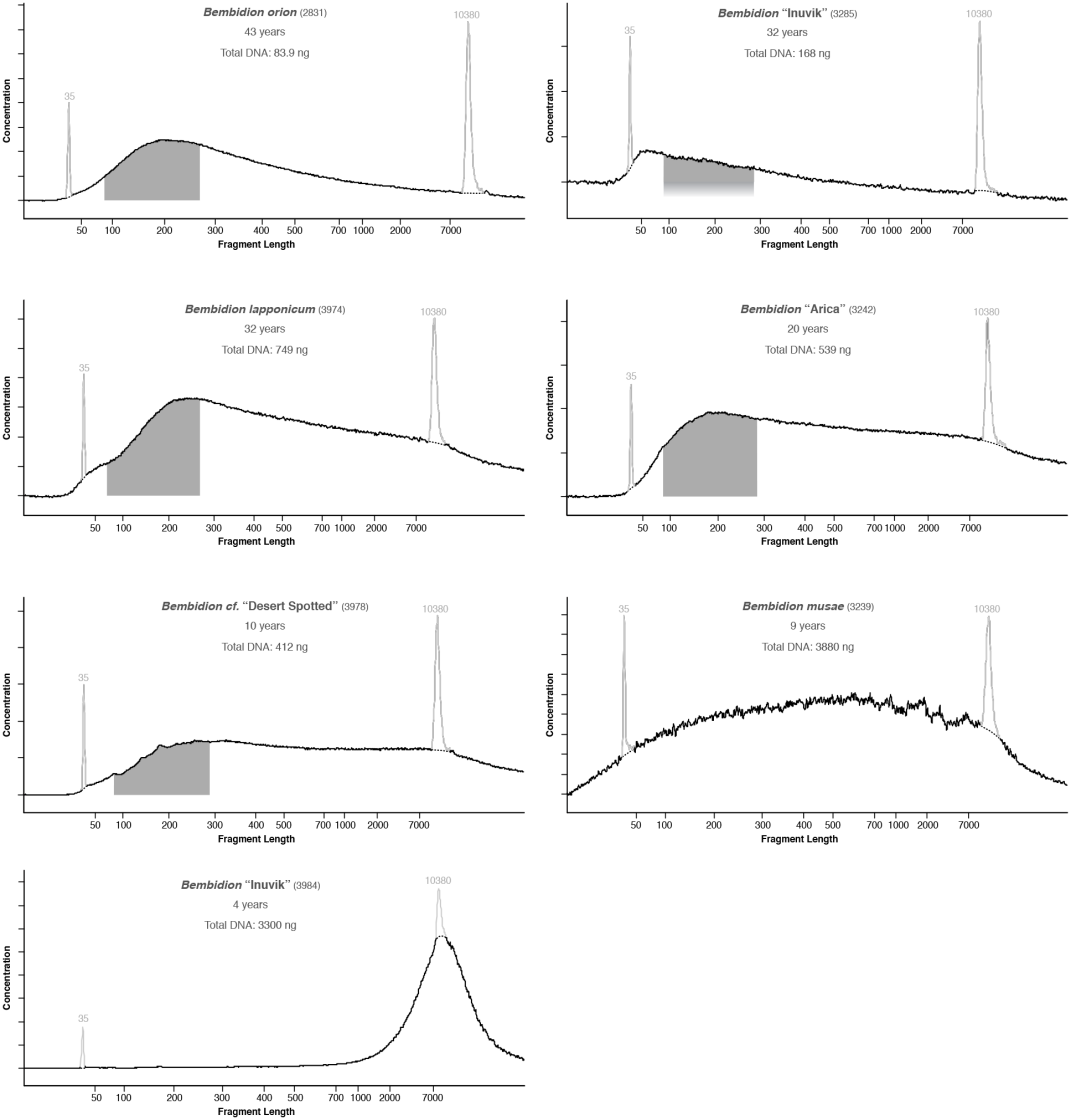
Electropherograms of DNA extracted from younger museum specimens that were subsequently used in library preparation. Pale spikes at 35 and 10380 bases represent standards included in each analysis. Dark shaded regions, when present, correspond to range of fragments that were selected and sequenced on the Illumina HiSeq 2000. Regions are not shown for *Bembidion musae* or *Bembidion* “Inuvik” 3984 as the DNA in those samples was sonicated prior to library preparation. For each specimen, age and total DNA in the extraction is also shown.

We created a synthetic measure of DNA quality by combining total DNA content values with the shape of the fragment-length distribution. We binned the 30 DNA extractions in Tables 4 and 5 into the following quality categories:

1. No measurable DNA in the Qubit (which means total DNA was less than about 0.06 ng) and no identifiable deviation from the baseline in the fragment-length distribution curve (e.g., *Bembidion subfusum* 2494, in S1 Fig).
2. With measurable DNA, modal fragment length below 100 bases, but no fragments longer than 400 bases (e.g., Lagriinae n. gen., KK0290, in Fig 4)
3. 40–220 ng total DNA, modal fragment length between 50 and 190 bases, with 3–10% of the fragments longer than 500 bases.
4. 80–250 ng total DNA, modal fragment length between 200 and 220 bases, with more than 15% of the fragments longer than 500 bases.
5. Between 400 and 550 ng of total DNA, modal fragment length around 200–300 bases, with some fragments greater than 1000 bases (e.g., *Bembidion* “Arica” 3242, in Fig 5).
6. More than 2000 ng of total DNA, modal fragment length greater than 500 bases with many fragments greater than 1,000 bases (e.g., *Bembidion nesophilum* 3240, in S1 Fig).
7. Material killed and preserved in 100% ethanol, with abdomen removed to allow ethanol penetration, replacement of ethanol, and storage at -20°C. Although we did not measure fragment length distributions for these samples, we assumed the DNA to be well-preserved [28,29].

*Bembidion subfusum* 1955 did not fall into any of these categories. Its fragment-length distribution showed two distinct peaks (S1 Fig), possibly indicating the presence of contamination by a saprophyte (perhaps fungal or bacterial) involved in the degradation of the specimen after death.

For the museum specimens selected for HTS (Table 1), we also attempted to amplify and sequence short fragments of genes commonly used in beetle systematics using PCR and Sanger sequencing methods, and used the success or failure as a measure of DNA quality. All PCRs were performed on an Eppendorf Mastercycler ProS using TaKaRa Ex Taq and manufacturer-recommended protocols. We targeted four fragments belonging to a total of three genes: (1) a 360–365 base fragment of 28S ribosomal DNA (**28S f1**), (2) a second 650–750 base fragment of 28S (**28S f2**), (3) a 450 base section of *wingless* (***wg***), and (4) a 650-base of cytochrome oxidase I (**COI**; this is the so-called “barcode” region, [30]). Details of primers and cycling conditions are provided in S1 Methods.

PCR products that showed a band when stained with SYBr Green and run on a 1% agarose gel were cleaned, quantified, and sequenced at the University of Arizona’s Genomic and Technology Core Facility using a 3730 XL Applied Biosystems automatic sequencer. Assembly of multiple chromatograms for each gene fragment and initial base calls were made with Phred [31] and Phrap [32] as orchestrated by Mesquite's Chromaseq package [33,34] with subsequent modifications by Chromaseq and manual inspection. Multiple peaks at a single position in multiple reads were coded using IUPAC ambiguity codes.

We considered PCR a success only if the sequenced product appeared to belong to the beetle itself rather than some contaminant. If the resulting sequence BLASTed to a non-beetle sequence in NCBI’s NR database (accessed October 2014), then the PCR was considered a failure. For example, the COI sequence from 4002 (*Lionepha chintimini*) matched with 100% identity GenBank sequences for *Homo sapiens*; two other sequences obtained through PCR BLASTed to bacteria. The remaining PCR-based sequences are all relatively similar to the Illumina-based results. In most cases, the PCR fragments are identical to the Illumina fragments, or the two differ only by one being an ambiguous superset of the other (S3 Table). For *B*. “Arica” and *B*. *musae*, however, there was at least one unambiguous difference between the PCR and Illumina fragments in 28S or *wingless*. For these, we conducted a single RAxML likelihood search of the matrices containing all taxa including the merged Illumina sequences (see below). The success of the PCR reaction was confirmed in *wingless* by a sister group relationship between the PCR fragments and the Illumina fragments of the same specimen, and in 28S by the PCR fragment of *B*. *musae* being in a clade with predicted relatives *B*. *parviceps*, and *B*. *anchonoderum*, and the Illumina *B*. *musae* sequence.

### Library preparation and sequencing

We selected 15 (13 museum specimens and two reference specimens, Table 1) for HTS based on their DNA quality and our desire to obtain sequences from them. Although we did not select any samples from category 0, we did include several samples with highly degraded DNA (category 1 and 2).

We used the DNA quality metrics outlined above to inform our library preparation procedures for each DNA extraction. Library preparation details for each sample are provided in Table 7, and details related to the protocols used are given in S1 Methods. Extractions from DNA quality categories five and six were first sheared using a Bioruptor^**®**^ Pico Sonication System (Diagenode). Other extractions were not sheared because their DNA was already fragmented. Samples containing short DNA fragments were manually prepared using TruSeq ChIP Sample Prep Kit (Illumina) as it is better optimized for shorter DNA fragments than the other kits that were available when we performed the library preparations. For extractions with longer DNA fragments we either manually prepared libraries using the TruSeq DNA Sample Prep Kit (Illumina) or automated preparations using the Apollo 324 NGS Prep System with the PrepX ILM DNA Library Kit (Wafergen).

**Table 7:** Library preparation details.

Taxon	Sample	Prep kit	DNA used (ng)	Min. insert size (bases)	Max. insert size (bases)
Lagriinae n. gen.	KK0290	ChIP	10	60	240
*Bembidion subfusum*	3977	ChIP	10	50	180
*Bembidion* sp. nr. *transversale*	3021	DNA	164	40	250
*Lionepha chintimini*	4002	PrepX	246	50	360
*Bembidion lachnophoroides*	3022	ChIP	8.25	80	280
*Bembidarenas*	3983	ChIP	10	50	240
*Bembidion orion*	2831	DNA	56	80	270
*Bembidion* "Inuvik"	3285	ChIP	10	80	280
*Bembidion lapponicum*	3974	ChIP	10	70	270
*Bembidion* "Arica"	3242	ChIP	10	80	280
*Bembidion cf*. "Desert Spotted"	3978	ChIP	10	80	280
*Bembidion musae*	3239	PrepX	300	60	740
*Bembidion* "Inuvik"	3984	PrepX	300	120	1480
*Bembidion orion*	3079	DNA	146	130	630
*Bembidion* sp. nr. *transversale*	3205	DNA	537	130	530

**Abbreviation for Prep kit**: ChIP: TruSeq ChIP Sample Prep Kit (Illumina), DNA: TruSeq DNA Sample Prep Kit (Illumina), PrepX: ILM DNA Library Kit (PrepX). **DNA used**: Amount used in the preparation of the sequenced library. **Min. insert size and Max. insert size**: measured by bioanalyzing the libraries. The first 11 samples were not sonicated; the last four (samples 3239 through 3205) were.

Libraries were run on an Illumina HiSeq 2000 maintained by the Oregon State University Center for Genome Research and Biocomputing. Each sample was given roughly 1/6 or 1/12 of a 100 base paired-end lane, with the exception of *B*. sp. nr. *transversale* 3205 which was run on a full lane. Samples run on 1/12 of a lane were done so not because of lack of library but because of financial considerations.

### *De novo* assembly

Demultiplexing and adaptor trimming was performed using CASAVA version 1.8 (Illumina). Paired-end reads were imported into CLC Genomic Workbench version 7.0.4 (CLC Bio), using default options except for the minimum and maximum paired-read distances, which we determined by analyzing a dilution of the enriched library on a Bioanalyzer 2100 (Agilent Technologies). Failed reads were removed during import. On average 0.9% of reads were discarded (0.26–2.4%). We used the “Trim Sequences” tool in CLC (with default parameters) to remove read ends with low quality or ambiguous base calls, and discard short reads. We generated *de novo* assemblies with the assembler in CLC using default parameters.

### Recovery of Core Eukaryotic Genes

Each *de novo* assembly was analyzed using CEGMA version 2.5 [24]. CEGMA searches sequences for a core set of highly conserved genes (CEGs). The relative abundance of these proteins provides a rough approximation of assembly quality. A gene is considered to be ‘complete’ if more than 70% of the CEG length is recovered, and ‘partial’ if less than 70% is recovered but the gene alignment exceeds a pre-computed minimum score [24].

### Recovery of 67 low-copy nuclear protein-coding gene fragments

We conducted a more thorough examination of low-copy nuclear protein-coding gene recovery by searching our HTS data for a set of 67 gene fragments previously used in arthropod phylogenetics [25]. The original 68 gene fragments in Regier et al. [25] included a fragment of the gene CAD; we excluded it from consideration, as we examined it more thoroughly in our seven focal gene study (see below). We explored *de novo* and reference-based assemblies of the HTS museum specimens to test for recovery of target regions.

#### Obtaining query sequences for 67 low-copy nuclear gene fragments

In order to test for recovery success of the 67 gene fragments from Regier *et al*. [25], it was first necessary to obtain query sequences for each gene with which to probe our assemblies. We generated these query sequences using the *de novo* assembly of reference specimen *B*. sp. nr. *transversale* 3205 for carabids and the *Tribolium castaneum* genome [35] for the Lagriinae n. gen. The methods we used to identify and extract orthologs of the 67 Regier *et al*. gene fragments are provided in S1 Methods. We were not able to recover orthologs for two of the gene fragments from *T*. *castaneum*.

#### Measuring recovery of 67 low-copy nuclear gene fragments in museum specimens

To test for recovery of the 67-gene set from *de novo* assemblies we created a BLAST database of contigs from each HTS specimen’s assembly, which we then queried using BLASTn (e-score cutoff: 1e-30; Word Size: 11; Scoring Match Mismatch: 2–3; Gap Cost: 5 2). All carabids were queried using the 67-gene set from *B*. sp. nr. *transversale* 3205 and Lagriinae n. gen was queried using the 65-gene set from *T*. *castaneum*. The BLAST searches often resulted in multiple contigs matching the query. To select orthologs, we examined the amino acid translation of the hits and first eliminated any sequences that contained any stop codons. If multiple hits were still retained but did not overlap or only overlapped by at most 30 bases, the union of the bases in each sequence was taken. If there was greater overlap, the search was assessed as having failed to recover the loci.

For carabids, we also performed reference-based assembly [36–39] in CLC using the *B*. sp. nr. *transversale* 3205 query sequences as the reference. We did not perform reference-based assembly for Lagriinae n. gen. because genomic resources for a sufficiently close relative were not available.

For each specimen, the 67 assembled fragments from both *de novo* and reference-based assemblies were examined for the percentage of the total fragment length that was recovered. This yielded four measures of success: NPDN50 (percentage of the 67 nuclear protein-coding gene fragments represented by at least 50% of the fragment length in the *de novo* assembly), NPDN80 (same, but represented by at least 80% of the fragment length), NPRef50 (percentage of the 67 nuclear protein-coding gene fragments represented by at least 50% of the fragment length in the reference-based assembly), NPRef80 (same, but represented by at least 80% of the fragment length).

Coverage for each specimen was calculated by averaging the coverage values produced by CLC for each of the 67 fragments with one adjustment: the length of the *recovered* portion was used to calculate coverage instead of the total length of the reference fragment.

### Recovery of seven focal genes

As a more rigorous test of gene recovery, we extracted seven additional genes (all distinct from the 67 gene set) from our HTS data, including four low-copy nuclear protein-coding genes that KK and DRM have sequenced throughout tenebrionids and carabids. These genes were chosen because available sequence data from related taxa would allow us to test the accuracy of our HTS data using phylogenetic analyses. The gene fragments we targeted are: **18S** or 18S rDNA: approximately 2000 bases of 18S nuclear ribosomal DNA; **28S** or 28S rDNA: approximately 1000–1100 bases of 28S nuclear ribosomal DNA; **COI**: between 650 and 1500 bases of the mitochondrial gene cytochrome oxidase I; **CAD**: approximately 2600 (tenebrionids) or 730 (carabids) bases of the carbamoyl phosphate synthetase domain of the *rudimentary* gene; **ArgK**: approximately 815 bases of arginine kinase; **Topo**: approximately 890 bases of topoisomerase I; ***wg***: 450 to 540 bases of *wingless*.

#### Obtaining query and reference sequences for focal genes

In order to test for recovery of the seven focal genes, it was first necessary to obtain query or reference sequences for each gene with which to probe or produce our assemblies. We generated these sequences using the partially sequenced genome of *B*. sp. nr. *transversale* 3205 for carabids and the *T*. *castaneum* genome for the Lagriinae n. gen. The methods we used to identify and extract orthologs of the focal genes are provided in S1 Methods.

#### Measuring recovery of focal genes

To extract the focal genes from our *de novo* assemblies, we queried BLAST databases for each museum specimens using sequences from either *B*. sp. nr. *transversal*e or *T*. *castaneum*. For CAD, the entire query region was used in analyses of Lagriinae; in contrast, only approximately 730 bases (between primers CD806F and CD1098R2 in [27]) were used as a query sequence for carabids.

BLAST searches often resulted in multiple contigs matching the query (S4 Table). To select potential orthologs among these contigs we first BLASTed all contigs found against GenBank’s nucleotide database, and all contigs for which there was at least one non-insect sequence within the first 50 hits were immediately discarded. Contigs that overlapped with the analyzed region by less than 30 bases were also excluded. The remaining contigs were judged as potential orthologs.

For some analyses, we sought for each sample a single sequence representing the ortholog of the target gene. We first discarded contigs for any protein-coding genes that showed internal stop codons. If there were two or more potential orthologs for a specimen, we attempted to select a single sequence as follows. If one contig was 90% or more of the length of the analyzed fragment, and the remainder were all less than 70% of the length of analyzed fragment and were fully contained within the span of the longest contig, then the longest contig was chosen. If that rule did not apply, and all contigs only partly overlap (i.e., no contig is contained within another contig’s span), and the overlap is less than 25 bases, then the union of the aligned contigs was used as the primary sequence (with any conflicting bases converted to IUPAC ambiguity codes). If that rule did not apply, no primary *de novo* sequence was chosen, and the *de novo* assembly was viewed as a failure for that gene. There were two exceptions to these rules, one intentional, and one not. The single *de novo* contig produced for *Bembidion* sp. nr. *transversale* 3021 had a stop codon (TGA) where 18 other specimens of this species [40] have a TGT. However, this 296-base *de novo* sequence was identical to that from the near-reference-based assembly except for that single nucleotide. We decided to include the *de novo* sequence in downstream analyses to see if the *de novo* sequence fell where predicted. The other exception to the rules was the accidental exclusion of a *de novo* CAD sequence that met the criteria from *Bembidion* “Inuvik” 3984. This 616-base sequence differed by only one nucleotide from its ortholog in the far-referenced-based assembly. This exclusion was discovered after all analyses were completed; we believe that inclusion of this sequence with a single nucleotide difference would not have appreciably affected the results for this specimen.

In addition to the *de novo* assemblies, we conducted reference-based assemblies for the carabids sequenced with HTS, taking advantage of available DNA sequences of the seven focal gene fragments and current understanding of relationships within the supertribe Trechitae [27]. For each HTS specimen, we used two or three references so that we could explore whether the reference sequence used biased the results. For all carabids, we chose a distantly related species, *Asaphidion yukonense*, as a “far reference”. It is expected that *Asaphidion yukonense* will be equally distantly related to all museum carabids studied except for *Bembidarenas* and *Lionepha*, to which it is expected to be somewhat more distantly related [27]. As a counterpart to this far reference, we chose a “near-reference” that varied among HTS specimens. This near reference belonged to a different species than the HTS specimen, but a species that was presumed closely related to the HTS specimen. Details of which far and near references were used for museum specimens are provided in S1 Methods.

We performed assemblies using the “Map Reads to Reference” tool in CLC Genomics Workbench version 7.0.4. Default parameters were used with two modifications: the length and similarity fractions were increased to 0.9 and 0.8, respectively, to reduce the chance of spurious read mappings.

For most carabid samples, there were four or more sequences for each of the focal genes: the *de novo* sequence (“DeNovo”), the reference-based assembly sequence from a distantly related species (“FarRef”), and the reference-based assembly sequence from a closely related species (“NearRef”). We also formed a single sequence (IlluminaMerged) for each gene fragment by taking the union of the FarRef, NearRef, and DeNovo sequences. Any conflict between those sequences was represented by an IUPAC ambiguity code.

### Tests of accuracy of Illumina results

A comparison of the Illumina results from museum specimens with sequence data obtained from fresh specimens from the same species using traditional PCR and Sanger sequencing would provide a measure both sequencing error and sequence changes through DNA degradation in the museum specimens. We made the comparison where possible; however, for most of the species sequenced there do not exist specimens preserved using methods that ensure the maintenance of high-quality DNA. For this reason, in general we took an alternative approach, using phylogenetic analysis, to help verify the Illumina results.

We combined sequences generated from HTS of museum specimens with sequence data of fresh specimens generated through PCR and Sanger sequencing and conducted phylogenetic analyses. We predicted the smallest clade likely to contain each museum specimen using previously obtained morphological and molecular evidence. If the HTS sequences fell in the expected phylogenetic position, they were judged to have passed this test regarding their accuracy.

Some of the PCR/Sanger sequences that formed the basis of the matrix into which the HTS sequences were included have been previously published, but some we acquired for this study using PCR and Sanger sequencing. Alignment and phylogenetic inference methods are described in S1 Methods.

#### Tenebrionidae: Taxon sampling and matrix acquisition for the phylogenetic test

For tenebrionids, there are currently no sufficiently extensive published matrices of DNA sequences to assess the phylogenetic placement of Lagriinae n. gen., and we therefore assembled a matrix of sequences for taxa that could provide the context with which to judge the HTS sequences. A few tenebrionid sequences were retrieved from GenBank but the majority were newly sequenced for this study. PCR and Sanger sequencing was conducted using protocols described in S1 Methods for 28S, 18S, COI, ArgK, CAD, and *wg*. We sampled an additional 29 lagriines representing all nine currently recognized tribes [41] and seven Tenebrionidae from other subfamilies (S10 Table) to infer the phylogeny of Lagriinae and examine the placement of the museum specimen. Collection information for all newly sequenced specimens can be found in S5 Table.

#### Tenebrionidae: Phylogenetic predictions

Among the taxa sampled, we predicted that Lagriinae n. gen., *Chaetyllus* and Lagriinae n. gen. 2 would form a clade. These taxa share numerous morphological similarities. However, as no analysis has been conducted regarding which of these states are derived and which are ancestral within lagriines, our prediction is based on the close similarity of these three taxa rather than an explicit phylogenetic analysis.

#### Tenebrionidae: Alignment and phylogenetic analysis

Detailed discussion of alignment and phylogenetic analyses is provided in the supplementary materials S1 Methods. In brief, we conducted sequence alignments for genes containing indels using MAFFT [42,43]. For 28S and 18S, poorly aligned regions were masked using the server version of GBlocks [44,45] with all options for less stringent block selection chosen. Nucleotide substitution models for these two genes were selected using jModelTest 2.0 [46,47]. For protein-coding genes, optimal data partitions and nucleotide substitution models were chosen using PartitionFinder v1.1.1 [48] from initial partition schemes based on codon positions. We also used PartitionFinder for model and partition selection of the concatenated dataset starting from an initial partition scheme based on gene and codon position. Maximum Likelihood (ML) tree search and bootstrap analyses were conducted using RAxML [49] on all single gene alignments and the concatenated alignment.

#### Carabidae: Taxon sampling and matrix acquisition for the phylogenetic test

For the phylogenetic tests within carabids, we used a subset of 146 species from [27] as the base data set, supplemented by three *B*. *(Chilioperyphus)* species from [50], and *Bembidion orion* (specimen 3079) from [21]. The matrices of the seven genes were then slightly modified, in part because of problems in voucher identifications in those previous papers (see S1 Methods for details). To this base data matrix we added four additional taxa which were newly sequenced for this study (S11 Table): *Bembidarenas reicheellum #*2 (specimen 1450), *Lionepha chintimini* (specimen 4059; this specimen is stored at the University of Alaska Museum, voucher number UAM:Ento:170452), *B*. “Desert Spotted” (specimen 2786), and *Bembidion approximatum* (2141). The base matrices with which the HTS sequences were analyzed thus contain 154 taxa.

One nomenclatural action is needed: The specimens of *Lionepha chintimini* Erwin and Kavanaugh [51] sequenced here would have traditionally been called *Lionepha lummi* Erwin and Kavanaugh, given their geographic origins. However, *L*. *chintimini* and *L*. *lummi* are here considered synonyms based upon examination of male genitalia, other morphological features, and DNA sequence data from specimens from multiple localities (Maddison, unpublished). As first reviser (International Code of Zoological Nomenclature, Article 24.2), DRM choses *L*. *chintimini* as having precedence, and therefore the valid name.

To study the quality of the HTS DNA sequences of the museum specimens of *Bembidion* sp. nr. *transversale* in more detail, we compared them to sequences from other individuals of that and related species. For this, we extracted DNA and sequenced 28S, COI, CAD, and Topo using PCR/Sanger sequencing from 29 specimens preserved in ethanol (S11 Table). We examined these sequences in the context of data from [27,40].

#### Carabidae: Phylogenetic predictions

As with tenebrionids, there have been few explicit phylogenetic analyses of morphological characters in the subtribe Bembidiina and related carabids; thus, predictions regarding the phylogenetic placements of the museum specimens are for the most part based upon overall morphological similarity to previously sequenced taxa (Table 8). The two exceptions are *Bembidion lachnophoroides* and *Bembidion* “Arica”, which have patterns of known derived and ancestral characters that allow placement with greater confidence.

**Table 8:** Predictions about phylogenetic placement of museum specimens.

Taxon	Sample	Prediction	Evidence
Lagriinae n. gen.	KK0290	In clade with *Chaetyllus* n. sp. 5, Lagriinae n. gen 2	Morphologically similar to those two species
*Bembidion subfusum*	3977	In the subgenus *Odontium*	Morphologically very similar to *B*. *(Odontium) paraenulum*
*Bembidion* sp. nr. *transversale*	3021	Sister to *B*. sp.nr. *transversale* 3205	Morphological data suggests they are the same species
*Lionepha chintimini*	4002	In the *Lionepha erasa* species group	Morphologically very similar to members of this species group.
*Bembidion lachnophoroides*	3022	In the *Princidium* Complex of *Bembidion*	Shares the derived, punctate head and other features characteristic of the *Princidium* Complex.
*Bembidarenas*	3983	In a clade with *Bembidarenas reicheellum* #1 and #2	Morphologically very similar to those two species
*Bembidion orion*	2831	Sister to *B*. *orion* 3079	Morphological data suggests they are the same species
*Bembidion* "Inuvik"	3285	In the *B*. *dentellum* species group	Morphologically very similar to *Bembidion immaturum* in this species group
*Bembidion lapponicum*	3974	Sister to *B*. *lapponicum* 1604	Morphological data suggests they are the same species
*Bembidion* "Arica"	3242	Sister to the remaining species of subgenus *Chilioperyphus*	Shares the derived male genitalia characteristic of subgenus *Chilioperyphus* [50], but lacks the derived, convergent frontal furrows of other members of the group
*Bembidion cf* "Desert Spotted"	3978	Sister to *B*. "Desert Spotted" 2786	Morphological data suggests they are likely the same species
*Bembidion musae*	3239	In the *Ananotaphus* Complex of *Bembidion*	Morphological similarities to other members of the complex (see [52])
*Bembidion* "Inuvik"	3984	In the *B*. *dentellum* species group	Morphologically very similar to *Bembidion immaturum* in this species group

#### Carabidae: Matrix assembly and phylogenetic analysis

For each of the seven genes (28S, 18S, COI, ArgK, CAD, Topo, and *wg*) we created three matrices of carabid sequences. All matrices contained the base set of 154 taxa sequenced using PCR/Sanger methods (see above). The first (“All Contigs”) matrix included all contigs from the *de novo* assembly that BLASTed to query sequences from *Bembidion* sp. nr. *transversale* (see S4 Table). The second (“Three Separate”) matrix included the FarRef and NearRef sequences, plus the DeNovo sequence for those specimens for which the procedure described above, under “Measuring recovery of focal genes”, yielded a single sequence. The third (“Illumina Merged”) matrix included only IlluminaMerged sequences for the HTS specimens.

Alignment and phylogenetic analyses were similar to those used for the tenebrionid phylogenetic test, and are documented in S1 Methods.

#### Carabidae: Comparisons between PCR and Illumina sequences within species

For any museum specimens for which PCR of short DNA fragments was successful, we compared the PCR-based DNA sequence to the merged Illumina sequence from the same specimen. We aligned the PCR-based sequenced to Illumina sequence in MAFFT (using the L-INS-i algorithm) and visualized the alignments in Mesquite, and recorded the number of ambiguous and unambiguous differences for each comparison.

For eight museum specimens (*Bembidarenas* 3983, *Bembidion* “Inuvik” 3285, *B*. “Inuvik” 3985, *B*. *cf*. “Desert Spotted” 3978, *B*. *lapponicum* 3974, *B*. *orion* 2831, *B*. sp. nr. *transversale* 3021, and *Lionepha chintimini* 4002) and two reference specimens (*B*. *orion* 3079 and *B*. sp. nr. *transversale* 3205) we were able to compare Illumina sequences to PCR-based sequences of our focal genes from conspecific (or likely conspecific in the case of *Bembidarenas*) specimens that had been preserved for DNA. For each comparison, we aligned merged Illumina sequences of museum specimens to PCR-based sequences of conspecific specimens in MAFFT. We then visualized the alignments in Mesquite and counted the number of unambiguous base differences between conspecific sequences for each gene. For protein-coding genes, we also recorded whether base differences resulted in a non-synonymous substitution.

To further assess the accuracy of Illumina sequences obtained from museum specimens, we conducted species-level phylogenetic analyses of the *B*. *transversale* species group. This species group is a complex of several closely related taxa for which we already had complete taxon sampling of four genes (28S, COI, CAD, and Topo) of Sanger-sequenced data. We extracted the four gene regions from each of the DeNovo, NearRef, FarRef, and IlluminaMerged assemblies of museum specimen *B*. sp. nr. *transversale* 3021 and combined them with sequences of the remaining members of the species group. We also included sequences from the assemblies of our reference specimen of *B*. sp. nr. *transversale* 3205 in the analysis. The four single gene matrices were analyzed separately using RAxML, after alignment in MAFFT, using the same methods as documented in S1 Methods for other phylogenetic analyses (except that only 100 searches for the maximum likelihood tree were conducted).

### Factors affecting success of gene recovery

In order to examine the factors that might contribute to the variation we observed in our measures of sequencing success, we conducted an exploratory linear regression analysis using R version 3.1.2 [53]. Four measures of success were examined (NPDN50, NPDN80, NPRef50, and NPRef80), with ten potential explanatory variables (data provided in S9 Table):

1. Age: number of years between death of specimen and DNA extraction
2. DNA Quantity: total mass of DNA in extraction
3. DNA Quality Score: DNA quality score, as measured by DNA content and distribution of fragment lengths (see section Assessing DNA quality of museum and reference specimens)
4. Killing Chemical: method of killing of specimen, either by (2) immersion in 95%-100% ethanol, (1) immersion in lower-concentration ethanol, (0) some other killing method
5. Body Length: body length of specimen, from anterior edge of clypeus to posterior edge of elytra.
6. Modal Fragment Length: the most common fragment length in the DNA extraction
7. PCR Success: success at amplifying any of the short fragments of 28S, COI, or wingless
8. PCR 28S Success: success at amplifying either of the short fragments of 28S
9. PCR COI Success: success at amplifying the region of COI
10. Reads: the total number of Illumina reads obtained

We used an iterative approach to accommodate the potential for some explanatory variables to predict success only after controlling for predictive power of other explanatory variables. First we performed univariate linear regression, using each of the ten explanatory variables and each of the four success measures ('lm' function in R; 40 total univariate analyses). For any analysis between a success measure and an explanatory variable that showed a significant correlation (p<0.05), a secondary, bivariate regression was conducted on each of the remaining nine explanatory variables, thus controlling for the original explanatory variable, to see if any additional variables may predict success.

### Data deposition

Raw reads for all museum and reference specimens are submitted to NCBI Sequence Read Archive (accessions SRR2939013– SRR2939027).

Focal gene fragments recovered from the *de novo* assembly of Lagriinae n. gen. and those that were newly sequenced for the phylogeny of Lagriinae are deposited in GenBank (accessions KU233685-KU234083). Focal gene fragments from PCR/Sanger sequencing and the IlluminaMerged sequences of carabids are also deposited in GenBank (accessions KU233685-KU234083).

The *Tribolium castaneum* and *Bembidion* sp. nr *transversale* query sequences used to probe our museum specimens for the 67 nuclear protein-coding gene fragments and all alignments used in phylogenetic analyses (including the DeNovo, FarRef, and NearRef sequences), as well as trees from the phylogenetic tests, are deposited in Dryad (data available from the Dryad Digital Repository: http://doi.org/10.5061/dryad.q7m07).

### Nomenclatural acts

The electronic edition of this article conforms to the requirements of the amended International Code of Zoological Nomenclature, and hence the new names contained herein are available under that Code from the electronic edition of this article. This published work has been registered in ZooBank, the online registration system for the ICZN. The ZooBank LSID (Life Science Identifier) can be resolved and the associated information viewed through any standard web browser by appending the LSID to the prefix “http://zoobank.org/”. The LSID for this publication is: urn:lsid:zoobank.org:pub:EC22080B-7DB3-49A5-A89C-C5AFB6F681EB. The electronic edition of this work was published in a journal with an ISSN, and has been archived and is available from the following digital repositories: PubMed Central, LOCKSS.

## Results

### DNA quantity and quality

The amount of total DNA extracted from the 39 museum specimens examined ranged from being undetectable (i.e., < 0.61 ng) to over 3 μg. Total DNA was undetectable in eight specimens, 19 specimens had between 9 and 200 ng, and 12 specimens had greater than 200 ng of total DNA (Tables 4–6).

Of the 28 specimens that we bioanalyzed, modal fragment size could not be determined for six specimens as the fragment length distribution was essentially flat (S1 and S2 Figs). Modal fragment size ranged from 50–200 bases for 15 specimens, and was greater than 200 bases for seven specimens. The 28 specimens fell into DNA quality score categories 1–5 with the exception of one specimen (*Bembidion subfusum*, 1955), which did not fit any defined category due to a secondary peak in the bioanalysis curve.

Among the 15 specimens selected for HTS, total DNA extracted ranged from 9.9 ng to over 3 μg and modal fragment size of specimens ranged from 50 bases to more than 9000 bases (Figs 4 and 5). DNA quality score categories 1–5 were each represented by at least two specimens. Categories 0 and 6 were not represented in the museum specimens chosen for HTS. In general, specimens killed in the last 30 years tended to have longer fragment lengths and higher overall DNA quality scores. DNA quality metrics for each specimen are provided in Table 4.

We were unsuccessful at amplifying any of the four short gene fragments in six of the 14 museum specimens in the study (Table 9). PCR amplification was successful for the smallest fragment (28S f1) in three of the remaining specimens, and successful for two or more longer fragments in five specimens. In general, PCR amplification was less successful in older specimens (killed more than 32 years before extraction) and more successful in younger specimens (killed less than 32 years before extraction).

**Table 9:** Summary of success of PCR of four gene fragments.

Taxon	Sample	28S f1	28S f2	wg	COI
Lagriinae n. gen.	KK0290	-	no	no	no
*Bembidion subfusum*	3977	no	no	no	no
*Bembidion* sp. nr. *transversale*	3021	no	no	no	no
*Lionepha chintimini*	4002	**yes**	no	no	no
*Bembidion lachnophoroides*	3022	**yes**	no	no	no
*Bembidarenas*	3983	no	no	no	no
*Bembidion orion*	2831	no	no	no	no
*Bembidion* "Inuvik"	3285	no	no	no	no
*Bembidion lapponicum*	3974	**yes**	no	no	no
*Bembidion* "Arica"	3242	**yes**	**yes**	**yes**	**yes**
*Bembidion cf*. "Desert Spotted"	3978	**yes**	**yes**	no	no
*Bembidion musae*	3239	**yes**	no	**yes**	no
*Bembidion* "Inuvik"	3984	**yes**	**yes**	**yes**	**yes**
*Bembidion orion*	3079	**yes**	**yes**	-	**yes**
*Bembidion* sp. nr. *transversale*	3205	**yes**	**yes**	**yes**	**yes**

“no” indicates PCR failure, “yes” indicates PCR and sequencing success, “-” indicates that we did not attempt PCR.

All museum specimens for which we attempted library construction produced sequenceable libraries, even for samples with very small amounts of fragmented DNA.

### Assembly statistics

N50 ranged from 280 to 700 for 12 of the 13 museum specimens (Table 10), with *Bembidion* “Inuvik” 3984 having an N50 of 1,355. The two reference specimens preserved in 100% ethanol, *B*. *orion* 3079 and *B*. sp. nr. *transversale* 3205, had N50 of 3,625 and 1,983 respectively. Assembly length ranged from approximately 3–152 Mb in museum specimens compared to approximately 174 and 266 Mb in the reference specimens. Although the assembly of the museum specimen of *B*. *orion* 2831 was created with a comparable number of reads as the reference specimen *Bembidion orion* 3079, the N50, assembly length, and maximum contig length were all lower in the museum specimen (N50 = 673 versus 3,625, assembly length = 134Mb versus 173.4Mb).

**Table 10:** *De novo* assembly statistics.

Taxon	Sample	Reads used (millions)	N50	Assembly length (Mb)
Lagriinae n. gen.	KK0290	60	306	29.9
*Bembidion subfusum*	*3977* ^1^	*24*.*7*	*287*	*6*.*1*
*Bembidion* sp. nr. *transversale*	3021	62.7	280	36.9
*Lionepha chintimini*	4002	71.9	630	152.3
*Bembidion lachnophoroides*	3022	63.7	445	141.5
*Bembidarenas*	*3983* ^1^	*22*.*8*	*293*	*21*.*8*
*Bembidion orion*	2831	64.8	673	134
*Bembidion* "Inuvik"	*3285* ^1^	*33*.*9*	*325*	*3*
*Bembidion lapponicum*	3974	76.5	369	29.9
*Bembidion* "Arica"	3242	70.2	447	71.7
*Bembidion cf*. "Desert Spotted"	*3978* ^1^	*26*.*8*	*354*	*22*.*7*
*Bembidion musae*	3239	75.6	445	109.2
*Bembidion* "Inuvik"	3984	71.4	1355	139.8
*Bembidion orion*	3079	61.3	3625	173.6
*Bembidion* sp. nr. *transversale*	3205^2^	351.4	1983	265.7

^1^ When multiplexing libraries we aimed for 1/12 of a HiSeq2000 lane for these samples.

^2^ Run on an entire HiSeq2000 lane.

### CEGMA results

Assembly quality as judged by CEGMA is shown in Table 11. Assemblies from the two reference specimens (*Bembidion* sp. nr. *transversale* 3205 and *B*. *orion* 3079) had the most complete CEGs, with 73–74% of CEGs recovered, and both specimens had over 90% of CEGs partially recovered. We did not recover any CEGs, complete or partial, from museum specimens that had fewer than 34 million reads. Complete CEGs in the remaining nine samples ranged between 0% to 33.9%, with five of those samples yielding complete fragments of 6.9–12.9% CEGs. *Bembidion* “Inuvik” 3984, a specimen killed in high-concentration ethanol and then kept dried in a museum drawer for four years, yielded the highest percentage of CEGs of all the museum specimens (33.9% complete and 61.7% partial). The oldest specimen, Lagriinae n. gen., had only nine of the 248 CEGs partially present (3.63%). Although we expected samples with more degraded DNA to produce fewer matches than higher quality DNA, this was not always the case. *Bembidion lachnophoroides* (DNA quality score of 1) had matches to parts of more CEGs (41.1%) than *Bembidion lapponicum* (21.4%) and *Bembidion musae* (22.2%), both of which were preserved much more recently than *B*. *lachnophoroides*, and contained more and longer fragments of DNA (DNA quality scores of 4 and 5).

**Table 11:** Results from CEGMA analyses between contigs from *de novo* assemblies and 248 core Eukaryotic genes (CEGs).

Taxon	Sample	Complete (%)	Partial (%)
Lagriinae n. gen.	KK0290	0	3.63
*Bembidion subfusum*	*3977* ^1^	*0*	*0*
*Bembidion* sp. nr. *transversale*	3021	0.4	6.85
*Lionepha chintimini*	4002	8.47	36.3
*Bembidion lachnophoroides*	3022	10.9	41.1
*Bembidarenas*	*3983* ^1^	*0*	*0*
*Bembidion orion*	2831	22.6	56.5
*Bembidion* "Inuvik"	*3285* ^1^	*0*	*0*
*Bembidion lapponicum*	3974	11.3	21.4
*Bembidion* "Arica"	3242	6.85	37.1
*Bembidion cf*. "Desert Spotted"	*3978* ^1^	*0*	*0*
*Bembidion musae*	3239	12.9	22.2
*Bembidion* "Inuvik"	3984	33.9	61.7
*Bembidion orion*	3079	73.8	93.2
*Bembidion* sp. nr. *transversale*	3205	73	94.4

Percentage of the 248 CEGMA core eukaryotic genes (CEGs) recovered. A gene is considered to be ‘complete’ if more than 70% of the CEG length is recovered, and ‘partial’ if less than 70% is recovered but the gene alignment score exceeds a pre-computed minimum [24].

^1^ Samples for which less than 34 million reads were obtained.

The most direct comparison can be made between the two specimens of *Bembidion orion* that were sequenced, as their assemblies were based upon similar numbers of reads and the specimens had presumably similar genome sizes. The assembly for the museum specimen (2831, killed and dried 43 years before extraction) contained 22.6% complete CEGs, in contrast to the reference specimen (3079, preserved in 100% ethanol two years before extraction), whose assembly contained 73.8% complete CEGs. The percentages of partial CEGs found in the assemblies were slightly closer: 56.5% for the museum specimen, and 93.2% for the reference specimen.

### Recovery of 67 low-copy nuclear protein-coding gene fragments

**Fig 6:**
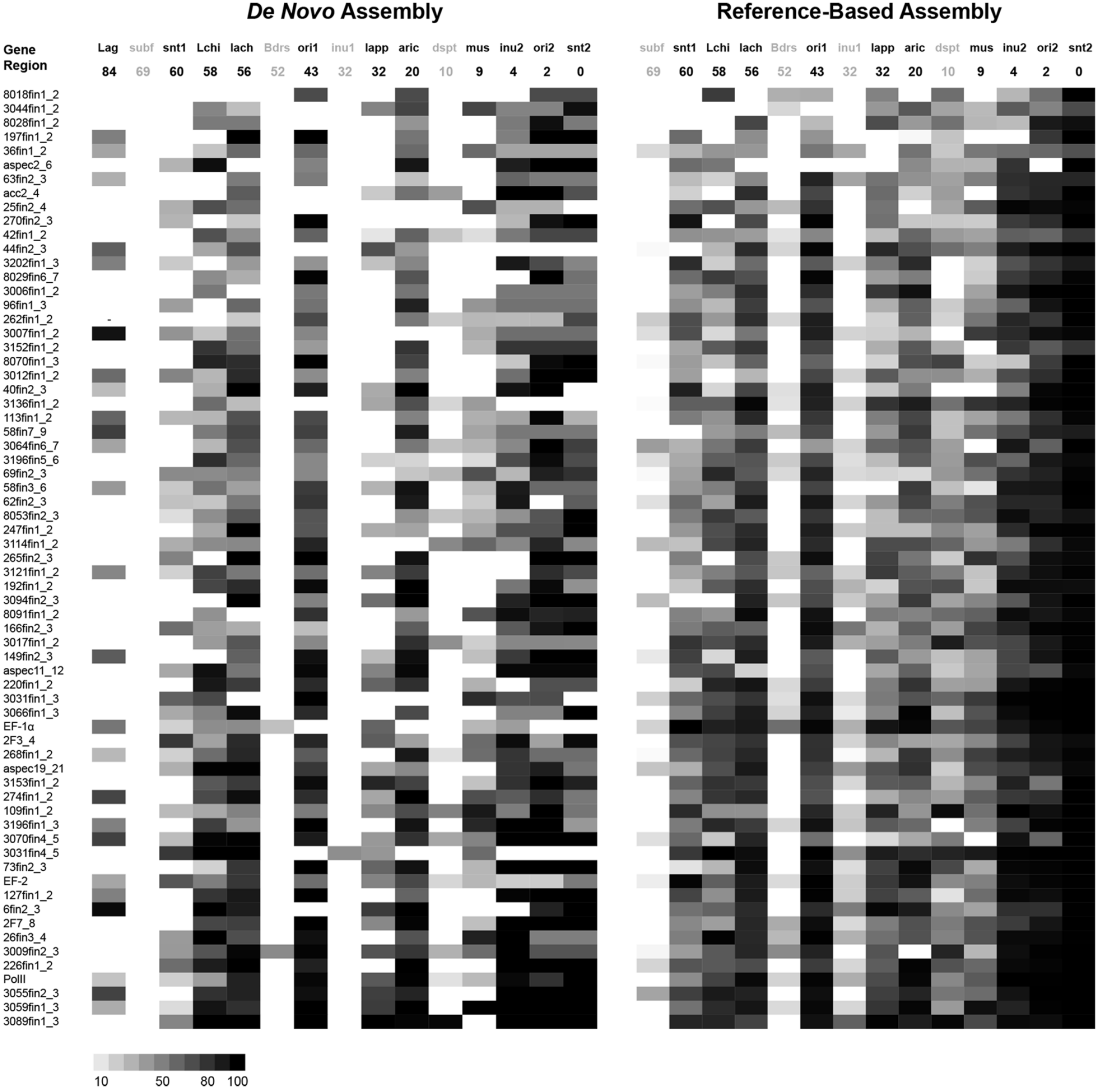
Recovery success of 67 low-copy nuclear protein-coding gene fragments in HTS museum specimens. Darkness of cell corresponds to percentage of the length of that fragment that was recovered, with black cells corresponding to 100% recovery. Gene fragments are ordered by average recovery as measured across both *de novo* and reference-based assemblies. Gene abbreviations are those used in Regier *et al*. [25]. Specimen abbreviations: Lag: Lagriinae n. gen. KK0290, subf: *Bembidion subfusum* 3977, snt1: *B*. sp. nr. *transversale* 3021, Lchi: *Lionepha chintimini* 4002, lach: *B*. *lachnophoroides* 3022, Bdrs: *Bembidarenas* 3983, ori1: *B*. *orion* 2831, inu1: *B*. "Inuvik" 3285, lapp: *B*. *lapponicum* 3974, aric: *B*. "Arica" 3242, dspt: *B*. *cf*. "Desert Spotted" 3978, mus: *B*. *musae* 3239, inu2: *B*. "Inuvik" 3984, ori2: *B*. *orion* 3079, snt2: *B*. sp. nr. *transversale* 3205. Four specimens with less than 34 million reads have specimen abbreviation and age shown in gray. Numbers under the specimen abbreviations are years between death and extraction.

Recovery success of the 67 nuclear protein-coding gene fragments from Regier *et al*. [25] is summarized in Fig 6 and Table 12, with numerical values provided in S7 and S8 Tables. In general, reference-based assembly recovered more and longer gene fragments from the set of 67 gene fragments than *de novo* assembly, with an average increase in recovered bases across all gene fragments and all specimens of 14%. The four specimens with reduced reads performed worse than the remaining specimens, and failed to recover even partial fragments of most target genes in *de novo* assemblies, although recovery improved for those specimens in reference-based assemblies (Fig 6, S8 Table). Of the twelve carabid museum specimens, all but one showed an increase in the average recovery in the reference-based assembly relative to the *de novo* assembly, with increases in additional bases recovered ranging from a low of 5% in *Bembidion subfusum* to a high of 31% in *Bembidion* sp. nr. *transversale* and 34% in *Bembidion cf*. “Desert Spotted” (S10 Fig). The one exception was *Bembidion* “Arica”, which showed a decrease in recovery in the reference-based assembly, having 3% fewer bases recovered on average across the gene fragments. Within the museum carabids, there were no apparent patterns with respect to the age of specimens and recovery success, nor were there many gene fragments that were equally recovered across specimens (Fig 6).

**Table 12:** Comparison of 67-gene set recovery between *de novo* assemblies and reference-based assemblies.

		*De Novo* Assembly				Reference-Based Assembly				
Taxon	Sample	% total bases	% genes >10% bases	% genes >50% bases [NPDN50]	% genes >80% bases [NPDN80]	% total bases	% genes >10% bases	% genes >50% bases [NPRef50]	% genes >80% bases [NPRef80]	Depth of Coverage
Lagriinae n. gen.	KK0290	22	37	21	3	-	-	-	-	-
*B*. *subfusum*	*3977* ^1^	*0*	*0*	*0*	*0*	*5*	*21*	*0*	*0*	*0*.*44*
*B*. sp. nr. *transversale*	3021	19	49	9	0	52	91	52	10	4.64
*Lionepha chintimini*	4002	49	84	49	21	53	88	58	15	2.44
*B*. *lachnophoroides*	3022	65	93	72	34	75	96	84	49	3.45
*Bembidarenas*	*3983* ^1^	*1*	*3*	*0*	*0*	*9*	*37*	*1*	*0*	*0*.*66*
*B*. *orion*	2831	68	87	72	43	80	97	90	55	4.25
*B*. "Inuvik"	*3285* ^1^	*0* ^2^	*1*	*0*	*0*	*14*	*51*	*4*	*0*	*1*.*12*
*B*. *lapponicum*	3974	26	48	19	3	51	94	55	10	2.20
*B*. "Arica"	3242	66	90	72	37	65	94	72	24	3.28
*B*. *cf*. "Desert Spotted"	*3978* ^1^	*8*	*24*	*1*	*1*	*42*	*91*	*34*	*7*	*1*.*74*
*B*. *musae*	3239	29	60	24	7	48	90	51	6	2.08
*B*. "Inuvik"	3984	62	87	64	34	80	99	93	58	3.97
*B*. *orion*	3079	71	93	81	55	88	99	97	79	7.16
*B*. sp. nr. *transversale*	3205	68	90	79	42	98	100	100	94	34.69

**% bases**: Percentage of total bases of the 67-gene set that were recovered. **% genes > X% bases**: percentage of genes in which more than X% of the query or reference length was recovered.

^1^ Samples for which less than 34 million reads were obtained.

^2^ One very short gene fragment was recovered, but the percentage was rounded down to 0.

Average depth of coverage across all 67 gene targets ranged from 0.44X to 4.64X for museum specimens (Table 12). Average coverage depth for the reference specimen *B*. *orion* 3079 was 7.16X, compared to 4.25X coverage in the museum specimen *B*. *orion* 2831 which had slightly more reads than the reference. The other reference specimen, *B*. sp. nr. *transversale* 3205, had 34.69X average coverage of targets, compared to 4.64X coverage of the museum specimen *B*. sp. nr. *transversale* 3021, although the museum specimen had had 5.6 times fewer reads than the reference. Depth of coverage was lowest for the four museum specimens with reduced reads, ranging from 0.44X to 1.74X, whereas all other museum specimens had average coverage of at least 2X. We should note that it was not possible to verify all 67 gene sequences for each taxon using Sanger sequencing or phylogenetic tests, and thus the accuracy of the recovered sequences is unknown.

### Recovery of the seven focal genes

**Fig 7:**
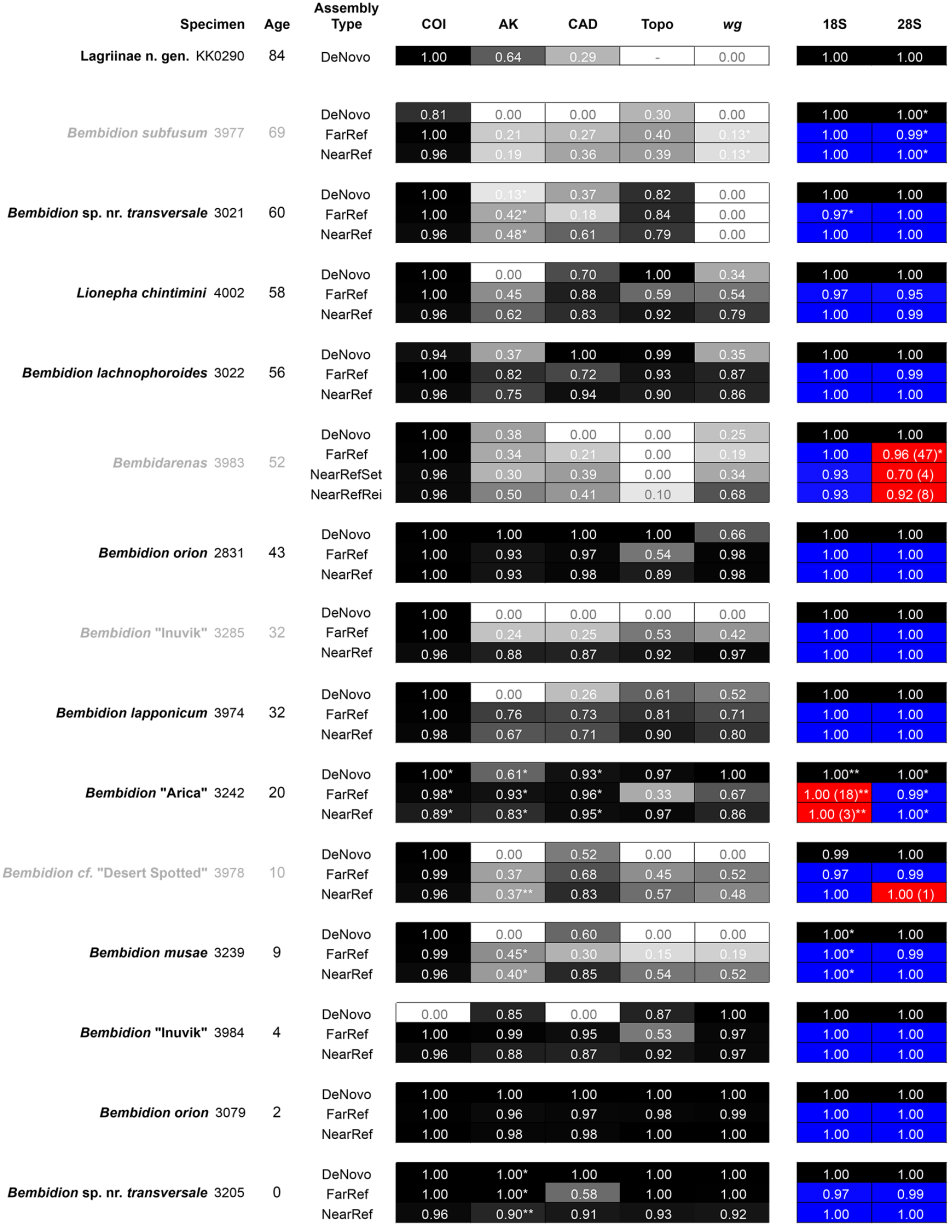
Recovery success of seven focal genes, with comparison of *de novo* and reference-based assemblies. For protein-coding genes, values in cells are the fractional recovery of the query sequence (for *de novo* assemblies) or reference sequence (for reference-based assemblies). Cells are shaded in a gray-scale ramp with black recovery of 100% of the fragment length and white 0%. For ribosomal genes, values in cells are the fractional recovery of the query sequence (for *de novo* assemblies), and for reference-based assemblies, values in cells represent the percentage recovery of the assembly relative to the *de novo* assembly (as opposed to the query or reference sequence). Values less than 1.0 indicate that some bases were missing from the reference-based assembly. A comparison of the *de novo* assembly sequence to the reference sequence shows that those missing regions are very different between the museum sample and the reference, and thus that region of the reference-based assembly failed. If there are no base differences between the reference-based and the *de novo* assemblies, the cell is colored using a blue ramp, with pure blue indicating 100% recovery. If there are base differences, the cell is colored red, with the number of base differences shown in parentheses. An asterisk (*) indicates that the sequence so marked is not in the predicted place in the maximum likelihood tree including the DeNovo, FarRef, and NearRef sequences; two asterisks (**) indicates that this placement failure is supported by a bootstrap value of over 50%. “-”indicates that no attempt was made to find this fragment in the assemblies. Four specimens with less than 34 million reads have specimen abbreviation and age shown in gray.

We recovered the entire target region of 18S and 28S sequences from all *de novo* assemblies except for *Bembidion cf*. “Desert Spotted” 3978, which was missing 12 bases at the start of the sequence (Fig 7). Many of the *de novo* assemblies contained multiple contigs that were returned in BLAST searches as matches for 18S or 28S (S4 Table), but the single contig chosen by our selection procedure in all instances passed our phylogenetic test for accuracy (see below). Reference-based assemblies performed slightly worse than *de novo* assemblies at recovering ribosomal genes in a few specimens, especially in regions of the genes with extensive insertions and deletions. However, with very few exceptions more than 90% of the length of the ribosomal gene fragments was recovered.

We recovered the entire barcoding region of COI from the *de novo* assemblies of all samples except *Bembidion* “Inuvik” 3984, for which our selection procedure did not choose a single sequence. This may have been caused by the presence of nuclear copies of COI, which has been previously documented in *Bembidion* [27,54]. As with the ribosomal genes, a number of the BLAST searches recovered multiple matching contigs. However, except for *B*. “Inuvik” 3984, all but one contig was notably shorter or had internal stop codons. The reference-based assemblies successfully recovered COI sequences for all specimens.

For focal nuclear protein-coding genes from *de novo* assemblies, we recovered complete sequences from all genes in both reference specimens (*Bembidion* sp. nr. *transversale* 3205 and *Bembidion orion* 3079), with museum specimens showing complete or partial recovery for most genes (Fig 7). *Bembidion* “Inuvik” 3285 was the only specimen in which recovery failed in *de novo* assembly of all four nuclear protein-coding genes. In general we recovered less data from *de novo* assemblies built from fewer reads (22–34 million, as opposed to more than 60 million), and failure to recover even partial gene fragments was common. For these specimens, reference-based assemblies increased data recovery. Although we recovered none of the four nuclear protein-coding genes from the *de novo* assembly of *B*. “Inuvik” 3285, we did recover a significant portion of the fragment in reference-based assemblies; especially when using the near reference. In contrast, there were a few reference-based assemblies, especially using the far reference *Asaphidion yukonense*, that recovered less data than corresponding *de novo* assemblies (for example, the far reference assembly of *B*. *lachnophoroides* for CAD) (Fig 7).

Because of the potential for cross-contamination between samples multiplexed in the same Illumina lane [55], we examined the phylogenetic placement of contigs for signs of within-lane contamination. Of all of the sequences obtained, only 10 of the small, discarded, *de novo* contigs showed any potential to be contaminants (that is, only 10 were similar to sequences expected from other samples present in the same lane). None of the reference-based sequences or *de novo* contigs chosen by our selection process appeared to be from cross-contamination between samples. All 10 of these discarded sequences were from samples run on one Illumina HiSeq 2000 lane, a lane with a higher-than-ideal cluster density of 932k/mm^2^. One of these 10 contigs is a 113-base piece of 18S (*Lionepha chintimini* 4002 contig 155118, S5 Fig) which is identical to that found in another sample in the same lane (a sample not included in this study). The other nine are small pieces of COI from *Bembidion orion* 2831 and *Bembidion* sp. nr. *transversale* 3021, which appear as paired terminals in the COI tree (S5 Fig); three of these sequences exactly match sequences of *Bembidion castor* (S5 Fig), a specimen which was also run in the same lane. Thus, although there are 10 contigs that may be within-lane cross-contaminants, all of these were discarded by the selection process we described above, which was based upon other criteria.

### Accuracy of sequences of the seven focal genes

#### Tenebrionidae: Phylogenetic tests

**Fig 8:**
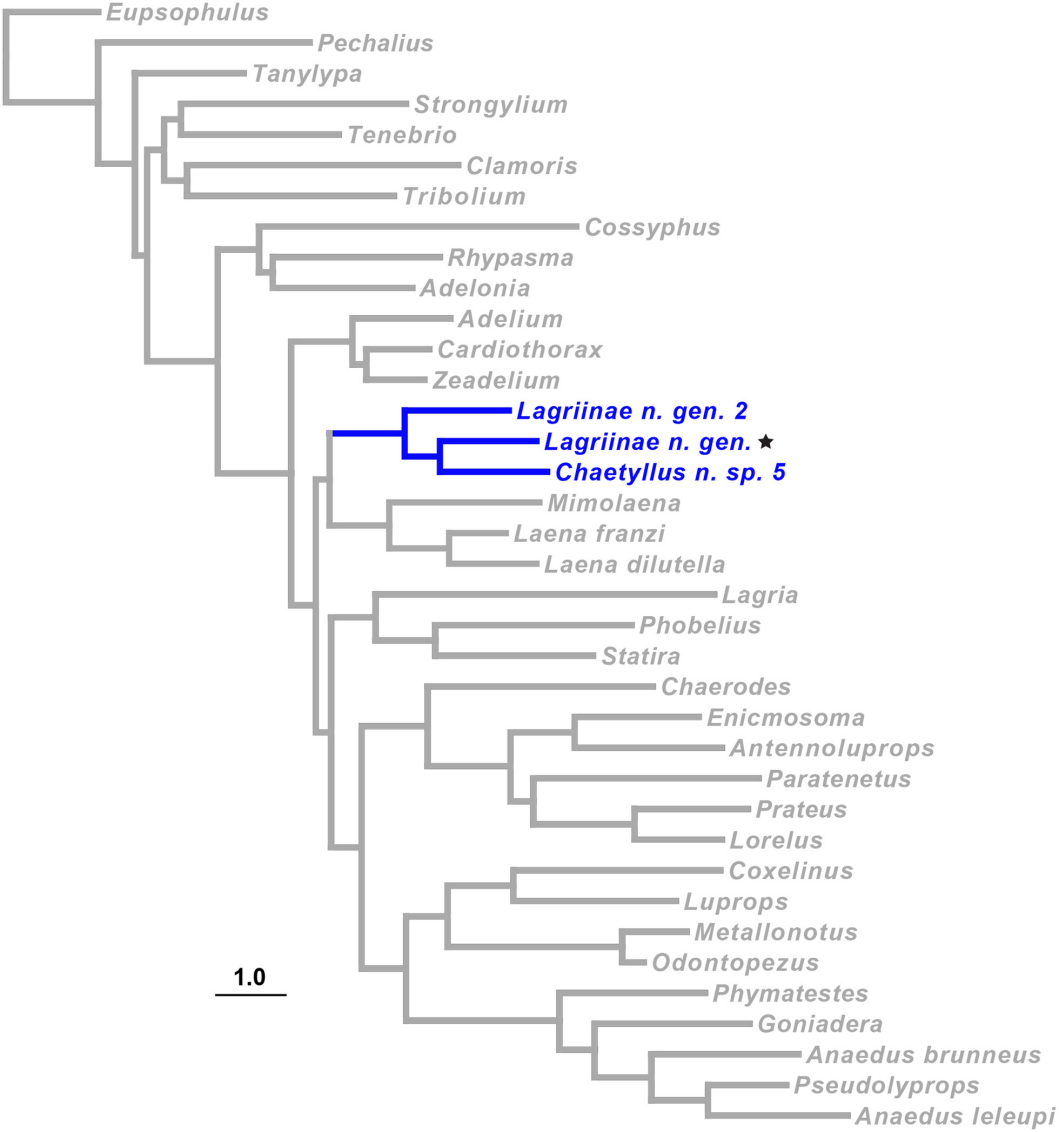
A maximum likelihood tree for the six-gene concatenated dataset of Lagriinae. The museum specimen is marked with a star symbol. The branches and taxon names of Lagriinae n. gen. and its predicted closest relatives (based on morphological characters) are colored in blue.

Lagriinae n. gen. was recovered in a clade with *Chaetyllus* n. sp. 5 and Lagriinae n. gen. 2, as predicted based on morphological characters, in ML analyses of the concatenated dataset (Fig 8), with strong support (bootstrap support = 100%; S4 Fig). These three taxa are also recovered in a clade in single gene analyses of CAD, and 28S (S3 Fig), though bootstrap support for the clade in the CAD analyses is low (61%; S3 and S4 Figs). We did not have ArgK data for *Chaetyllus* n. sp.5, but Lagriinae n. gen. is placed (as predicted) as sister to Lagriinae n. gen. 2, with strong support (bootstrap support = 96%).

#### Carabidae: Phylogenetic distribution of all contigs

**Fig 9:**
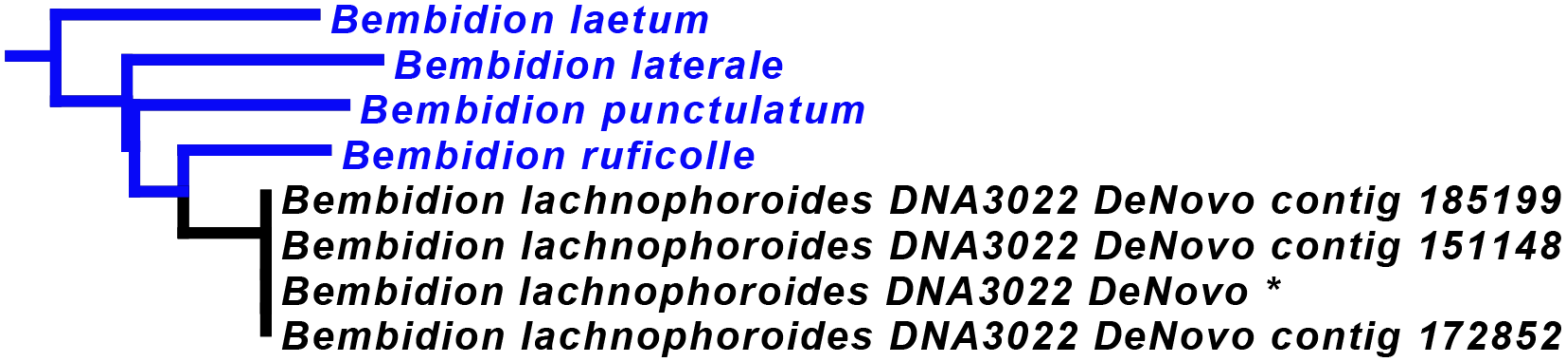
A portion of the maximum likelihood tree of carabids for Topo with all *de novo* assembly contigs included. An example in which our BLAST searches for target genes within HTS museum specimen assemblies returned multiple contigs, all of which were nearly identical and within the prediction group. The prediction group is shown in blue. The contig chosen by our filtering criteria is marked by an asterisk.

**Fig 10:**
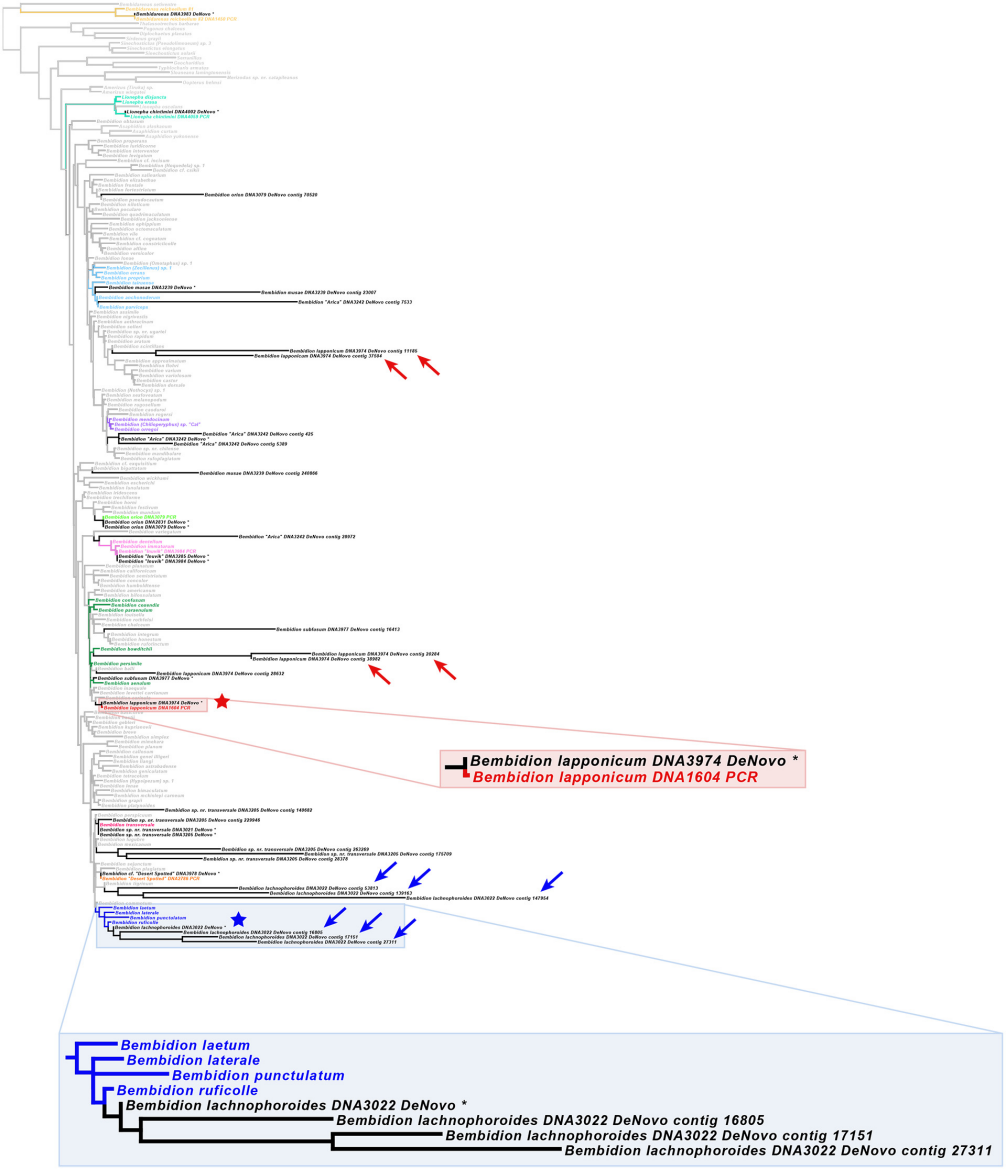
A maximum likelihood tree of carabids for 28S with all contigs included from the *de novo* assembly. An example in which our BLAST searches for target genes within HTS museum specimen assemblies returned multiple contigs, many of which were placed on long branches at unexpected positions across the tree. The behavior of the multiple contigs is highlighted using two museum specimens, *Bembidion lapponicum* 3974 and *Bembidion lachnophoroides* 3022. In both cases, our filtering criteria appear to select the best of the multiple contigs. Red star: chosen contig for *Bembidion lapponicum* 3974. Red arrows: other contigs from *B*. *lapponicum* 3974. Blue star: chosen contig for *Bembidion lachnophoroides* 3022; blue arrows: other contigs from *B*. *lachnophoroides* 3022.

**Fig 11:**
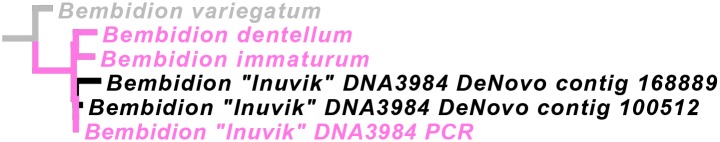
A portion of the maximum likelihood tree of carabids for CAD with all contigs included from the *de novo* assembly. An example in which our BLAST searches for target genes within HTS museum specimen assemblies returned multiple contigs, however our filtering criteria failed to accept a best contig, despite two contigs falling in the prediction group (shown in pink), and being nearly identical to the PCR-based sequence of a conspecific specimen (*Bembidion* “Inuvik” 3984).

**Fig 12:**
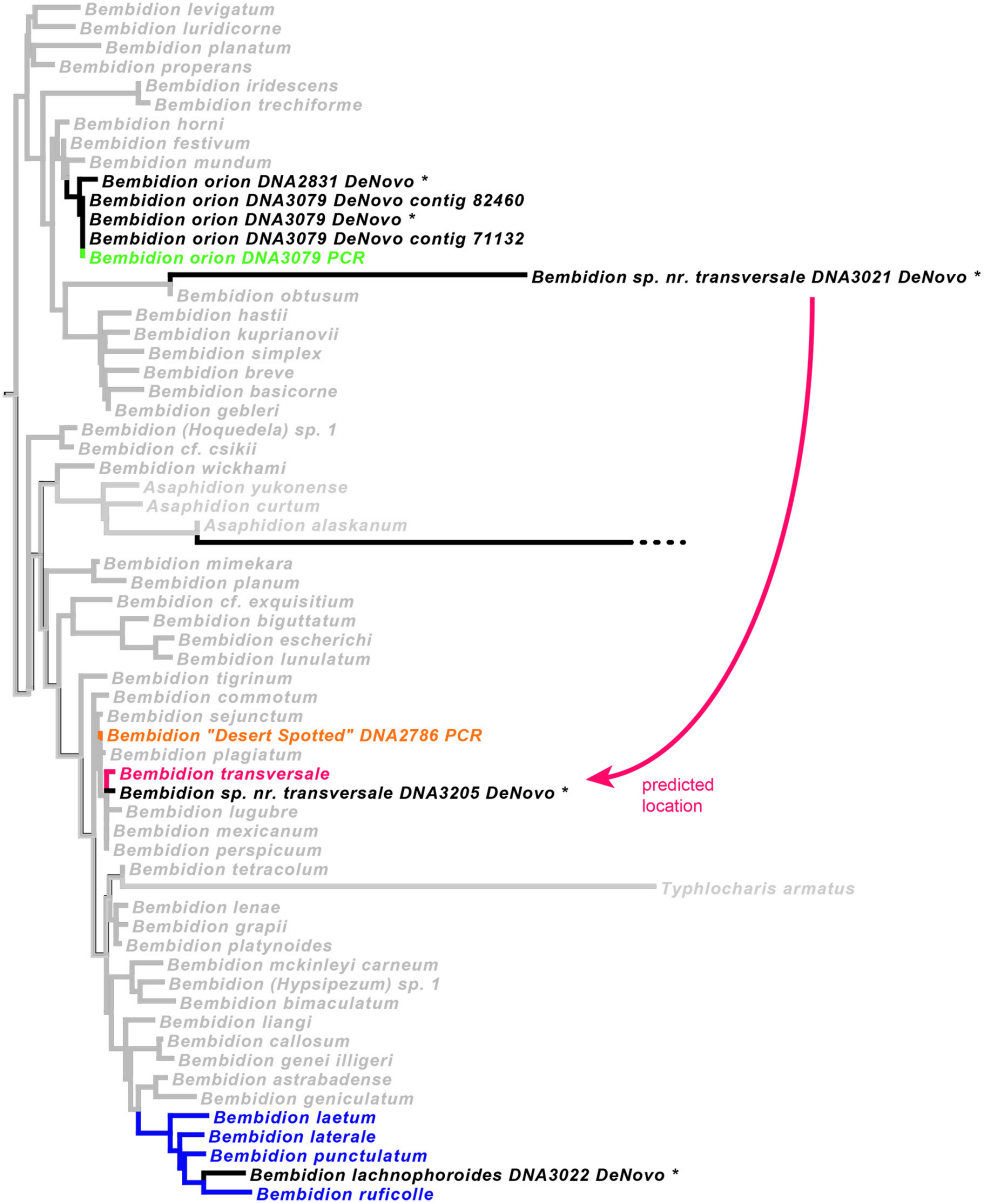
A portion of the maximum likelihood tree of carabids for ArgK with all contigs included from the *de novo* assembly. An example in which our BLAST searches for target genes within HTS museum specimen assemblies returned multiple contigs, however our criteria for choosing the best among multiple contigs selected a contig which was not inferred to be where predicted in the phylogeny.

As noted above, for some carabid samples, *de novo* assemblies contained multiple contigs that BLASTed only to beetles and that contained no stop codons (S4 Table). The phylogenetic analyses containing all contigs (S5 Fig) show various patterns of relationships between the multiple contigs in a sample. In many cases in which there was more than one contig for a sample, the different contigs formed a clade in the maximum likelihood tree (e.g., *Bembidion lachnophoroides* 3022 for Topo, Fig 9). Some had contigs scattered around the tree, but with the contig chosen by our selection process falling where predicted (e.g., *Bembidion lapponicum* 3974 and *B*. *lachnophoroides* 3022 for 28S, Fig 10). For some samples, some of the contigs that were not chosen were not where predicted and were extremely divergent (see, for example, the *B*. *lachnophoroides* COI contigs in S5 Fig). A third pattern is shown by *Bembidion* “Inuvik” 3984 for CAD (Fig 11): the two contigs appeared in the tree exactly where predicted, but our selection process failed to choose one over the other, and thus *de novo* assembly for this sample for CAD was judged to be a failure. In a very few cases none of the contigs were inferred to be where predicted in the phylogeny, including the single chosen contig (see, for example, *Bembidion* sp. nr. *transversale* 3205 in ArgK, Fig 12). In most cases, however, the chosen contig was inferred to fall where predicted in the phylogeny, or at least not strongly supported to fall in a contradictory place (S7 Fig).

#### Carabidae: Consistency and accuracy of DeNovo, NearRef, and FarRef sequences

**Fig 13:**
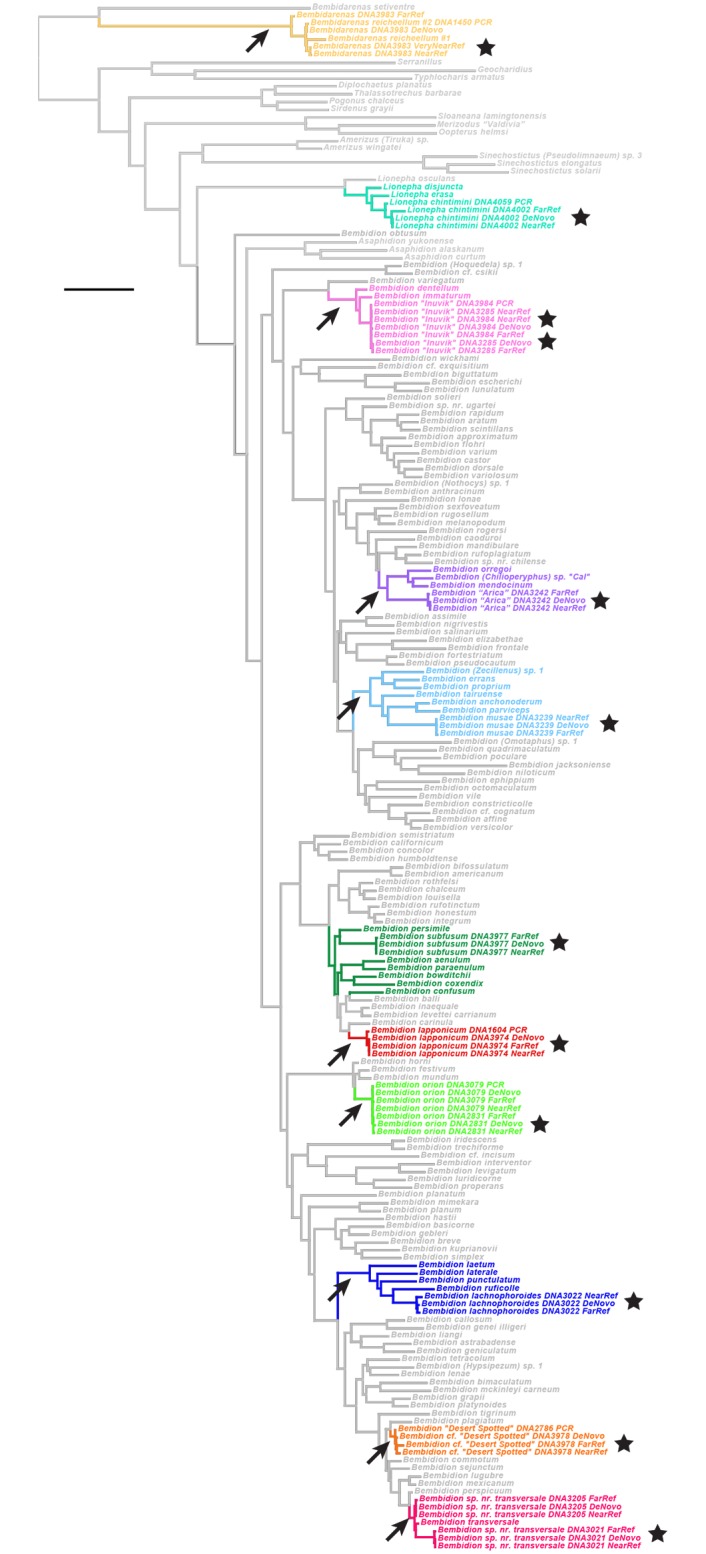
A maximum likelihood tree of carabids from seven focal genes and “Three Separate” assembly sequences. The placement of the DeNovo, NearRef, and FarRef sequences is shown relative to their prediction groups in a concatenated analysis of seven focal genes. Each prediction group is marked by a black arrow, and a unique color for branches and taxon names of all specimens in the prediction group. The placement of the three assembly sequences is indicated with a black star.

In the phylogenetic analysis of the seven-gene concatenated matrix, the concatenated, multi-gene DeNovo, NearRef, and FarRef sequences for all museum specimens were inferred in positions (Fig 13; S6 and S7 Figs) consistent with our predictions (Table 8). In addition to being inferred with their predicted group, DeNovo, NearRef, and FarRef sequences formed a clade for nine of 12 museum specimens (see also Table 13); seven of these clades were strongly supported, with bootstrap support over 90%. For the four specimens in which DeNovo, NearRef, and FarRef sequences did not form a clade, an interfering sequence from a conspecific specimen or very closely related species disrupted the clade. For *Bembidarenas* 3983, the FarRef sequence fell outside of a moderately supported clade (bootstrap support = 82%) that included the DeNovo and NearRef sequences, as well as the PCR-based sequences from two other *Bembidarenas* specimens in the matrix (*Bembidarenas reicheellum* #1 and #2). For *B*. “Inuvik” 3285 and B. “Inuvik” 3984, the NearRef sequence fell outside of a poorly supported clade (bootstrap support = 56%) that included the DeNovo and FarRef sequences of *B*. “Inuvik” 3285 and *B*. “Inuvik” 3984, as well as the PCR-based sequence from *B*. “Inuvik” 3984. For *B*. *orion* 3079, the DeNovo sequence fell outside of a poorly supported clade (bootstrap support = 53%) that included the NearRef and FarRef sequences of *B*. *orion* 3079 and *B*. *orion* 2831, as well as the PCR-based sequence from *B*. *orion* 3079.

**Table 13:** Support for or against *de novo* and reference-based sequences of each museum specimen forming a clade.

Taxon	Sample	7 Genes	18S	28S	COI	ArgK	CAD	Topo	*wg*
*Bembidion subfusum*	3977	100	100	92	100	83	98	100	89
*Bembidion* sp. nr. *transversale*	3021	100	-53/-	-87/94	85	-	-/69	82	
*Lionepha chintimini*	4002	84	-100	-86/64	-99/91	95	61	58	83
*Bembidion lachnophoroides*	3022	100	99	100	99	100	99	100	92
*Bembidarenas*	3983	-82/100	-100/100	-100/100	98	-56/100	-79/100		-94/69
*Bembidion orion*	2831	92	-99/100	-96/100	62	-/69	-65/100	-99/98	-56/94
*Bembidion* "Inuvik"	3285	-59/100	-98/100	-97/96	-83/100	-98/74	-/69	-69/95	-88/96
*Bembidion lapponicum*	3974	100	-	61	99	89	72	-76/100	-66/97
*Bembidion* "Arica"	3242	100	98	100	100	100	100	100	100
*Bembidion cf*. "Desert Spotted"	3978	78	59	-73/74	-96/100	-51	53	58	-/57
*Bembidion musae*	3239	100	100	100	100	99	100	94	96
*Bembidion* "Inuvik"	3984	-59/100	-98/100	-97/96	-83/100	-98/74	-/69	-69/95	-88/96
*Bembidion orion*	3079	-60/100	-99/100	-96/100	-	56	-60/100	-99/98	-56/94
*Bembidion* sp. nr. *transversale*	3205	91	-53/-	-87/94	69	-51	-/69	-	65

Bootstrap support for phylogenetic placement of museum specimen sequences in concatenated (7 Genes) and single gene analyses matrices with DeNovo, NearRef, and FarRef sequences. Single positive numbers indicate that there is that amount of maximum-likelihood bootstrap support for the far reference, near reference, and *de novo* sequence (if present) forming a clade exclusive of all other sequences. Cells with two numbers separated by a slash show, first, bootstrap support against the three sequences for each specimen forming a clade, and second, the bootstrap support for the three sequences forming a clade with members of the prediction group. Single negative numbers indicate bootstrap support against the three sequences forming a clade and support against them all being in the predicted group. Blank cells indicate that the sequence was not recovered by at least one assembly method; cells with only “-”indicate that the relationships of the three sequences were unresolved in the 50% majority rule bootstrap tree.

In single-gene analyses, DeNovo, NearRef, and FarRef sequences for each museum specimen appeared in an exclusive clade in the maximum likelihood trees in most cases, or at least with other sequences from the same species or closely related species (S6 Fig). In single-gene bootstrap analyses (S7 Fig), the placement of the three sequences from a specimen formed a clade in 43 of the possible 82 cases (Table 13). (There are 82 total cases considering 12 museum specimens and seven genes each, minus two because of the lack of relevant sequence of Topo for *Bembidarenas* and *wg* for *B*. sp. nr. *transversale* 3021.) In 26 of the remaining cases, the three sequences were not an exclusive clade, but they appeared in a well-supported, exclusive clade with conspecifics. In six cases (18S, 28S, ArgK, and *wg* for *Bembidarenas*, 28S and COI for *Bembidion* sp. nr. transversale 3021) the variation in the DeNovo, NearRef, and FarRef resulted in at least one of the sequences falling out with sequences from a different but very closely related species (*Bembidarenas* sp. #1 and *Bembidion transversale*, respectively) in the bootstrap analysis. In the bootstrap tree for *wg*, the various assembly sequences for *B*. “Inuvik” 3285 and *B*. “Inuvik” 3984 were intermingled with each other and with the PCR-based sequences for other members of the same subgenus (S7 Fig). In two additional cases, the relevant relationships are unresolved in the bootstrap tree.

In 16 of the 82 cases, at least one of the DeNovo, NearRef, or FarRef sequences fell outside the predicted groups, suggesting some level of inaccuracy. For example, even though the DeNovo, NearRef, and FarRef 28S sequences of *B*. “Arica” 3242 form a clade, the clade is not placed where predicted in the phylogeny. In 18S, the FarRef sequence for *L*. *chintimini* 4002 fell out as sister to the rest of the genus *Lionepha*, and thus outside the predicted group, with bootstrap support of 100. Six of the failures occur with ArgK; for example, the NearRef sequences of *B*. *cf*. “Desert Spotted” 3978 and *B*. sp. nr. *transversale* 3205 were not placed with sequences from other assemblies of the same specimens, but were instead sister to each other in the bootstrap tree (S7 Fig). Similarly, the reference-based assembly sequences of ArgK in *B*. sp. nr. *transversale* 3021 are not close to the DeNovo sequence in the maximum likelihood tree (S6 Fig).

#### Carabidae: Prediction outcomes in “Illumina Merged” analyses

**Fig 14:**
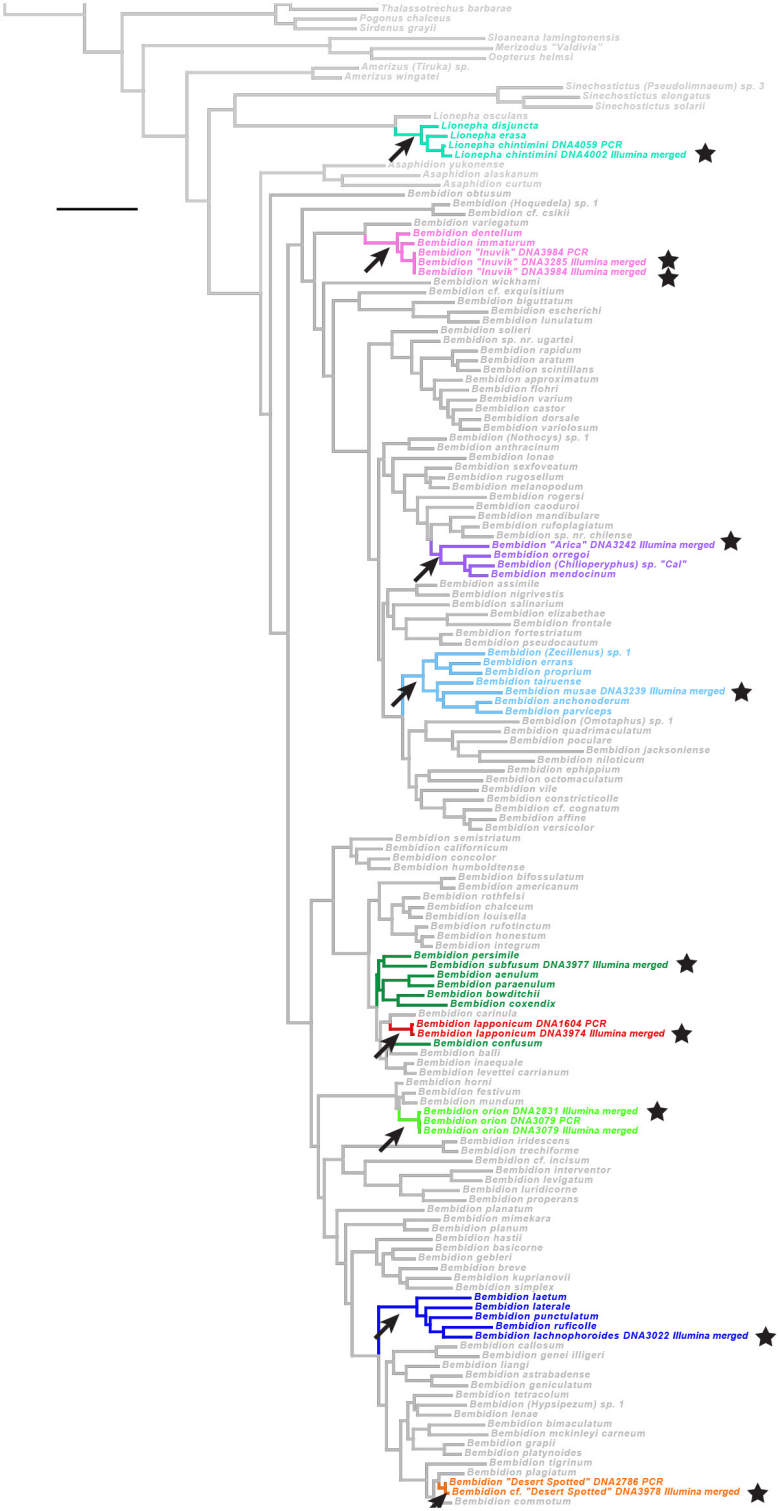
A maximum likelihood tree of carabids from seven focal genes and IlluminaMerged sequences. The placement of the IlluminaMerged sequences is shown relative to their prediction groups in a concatenated analysis of seven focal genes. Each prediction group is marked by a black arrow, and with a unique color for branches and taxon names of all specimens in the prediction group. The placement of each IlluminaMerged sequences is indicated with a black star.

In the seven-gene concatenated matrix, all IlluminaMerged sequences from carabid museum specimens were inferred in their predicted groups (Table 8) with high nodal support (bootstrap support range = 91–100%) (Fig 14, Table 14, S8 and S9 Figs), except for *B*. *subfusum*. *B*. *subfusum* did appear in the maximum likelihood tree in its predicted group (Fig 14), but without bootstrap support for the placement.

**Table 14:** Support for IlluminaMerged sequence being in predicted location in phylogeny.

Taxon	Sample	7 Genes	18S	28S	COI	ArgK	CAD	Topo	*wg*
Lagriinae n. gen.	KK0290	100	(-)	82	(-)	96	61	NA	–
*Bembidion subfusum*	3977	yes	(-)	(-)	yes	-59	yes	yes	**x**
*Bembidion* sp. nr. *transversale*	3021	100	**x**	99	100	-81	69	**x**	–
*Lionepha chintimini*	4002	100	51	100	100	96	99	98	97
*Bembidion lachnophoroides*	3022	100	71	64	57	84	96	76	95
*Bembidarenas*	3983	100	100	100	100	100	100	100	100
*Bembidion orion*	2831	100	100	100	100	95	100	100	99
*Bembidion* “Inuvik”	3285	100	100	98	100	78	98	94	97
*Bembidion lapponicum*	3974	100	**x**	99	100	85	100	100	100
*Bembidion* “Arica”	3242	91	-54	**x**	**x**	**x**	**x**	92	55
*Bembidion cf*. “Desert Spotted”	3978	100	NA	100	100	-61	99	91	61
*Bembidion musae*	3239	100	**x**	58	yes	**x**	100	yes	yes
*Bembidion* “Inuvik”	3984	100	100	98	100	78	98	94	97
*Bembidion orion*	3079	100	100	100	100	95	100	100	99
*Bembidion* sp. nr. *transversale*	3205	100	**x**	99	100	**x**	69	**x**	yes

Bootstrap support for phylogenetic placement of museum specimens in concatenated (7 Genes) and single-gene analyses of “Illumina Merged” matrices. Positive number indicate bootstrap support (expressed as a percentage) for museum specimen recovered in its predictive group with support value greater than 50. Negative values represent bootstrap support for museum specimen recovered with taxa not in the predictive group with support value greater than 50. “-”indicates sequence was not recovered; “yes” indicates taxa is recovered with predicted clade in ML tree but the placement is unresolved in the bootstrap tree; “x” indicates taxa is not recovered with predicted clade in either ML or bootstrap tree; “(-)” indicates predicted group exclusive of museum specimen is not present in either ML or bootstrap tree. “NA” indicates that missing data, either for the museum specimen (Lagriinae n gen. KK0290 for Topo), or the members of the prediction group (*Bembidion cf*. “Desert Spotted” 3978 for 18S) prevented testing of placement with the prediction group.

In single gene analyses, the placement of IlluminaMerged sequences from these museum specimens fell in the predicted group in 56 of the 84 cases, 50 of which were supported by bootstrap values greater than 50 (Table 14, S8 and S9 Figs). Of the remaining 28 cases, 14 represent true failures of the prediction, four of which are supported by bootstrap values of 54 to 81 (negative values in Table 14), and the remaining 10 without such support (“x” in Table 14).

#### Comparisons between PCR and Illumina sequences within species

For most museum specimens in which PCR of COI, *wg*, or one of the two fragments of 28S was successful, the PCR-based sequence matches that of the merged Illumina sequence (S3 Table). Ambiguous discrepancies were more commonly due to the presence of an ambiguous base in the PCR-based sequence rather than due to obvious assembly or sequencing errors. In some cases, the ambiguity in the PCR sequence was a result of poor sequencing reactions for both forward and reverse sequences. In other cases, the ambiguous bases in the PCR sequences occurred in clean sequences and are almost certainly heterozygous sites (for example, two of the ambiguities in *wg* for *B*. “Inuvik” 3984). Two museum specimens showed a total of four unambiguous discrepancies between PCR-derived sequences and those obtained from HTS.

A comparison of museum specimen merged Illumina sequence to PCR-based sequences from conspecific specimens (or probably conspecific specimens in the case of *Bembidarenas*) showed that the compared sequences were identical in more than half of the comparisons (Table 15). There were very few apparent discrepancies in the ribosomal markers (Table 15); COI sequences appeared quite accurate, with the few differences likely reflecting intraspecific variation. HTS museum sequences that were not identical to their conspecific counterpart showed unambiguous differences ranging from 1–6 bases per gene with two exceptions: *Lionepha chintimini* 4002 showed 22 unambiguous differences in ArgK, and *Bembidion* sp. nr. *transversale* 3021 showed 60 different bases in ArgK. For *L*. *chintimini* 4002, all but two of the 22 discrepancies were concentrated in one short fragment present in the reference-based assembly. In contrast, for *B*. sp. nr. *transversale* 3021, the discrepancies were scattered throughout the gene. We suspect that this represents HTS sequencing of an alternative copy or pseudogene, at least in part.

**Table 15:** Comparison of the IlluminaMerged sequences of museum specimens to sequences from conspecific specimens.

			Comparison Sequencing Method	Differences between Samples						
Taxon	Museum Sample	Comparison Sample		18S	28S	COI	ArgK	CAD	Topo	*wg*
*Bembidarenas*	3983	1450	PCR/Sanger	-	0	**5/0**	0	**6/4** *	-	**4/0** *
*Bembidion* "Inuvik"	3285	3984	PCR/Sanger	-	0	0	0	0	0	0
*B*. "Inuvik"	3984	3984	PCR/Sanger	-	0	0	0	0	0	0
*B*. *cf*. "Desert Spotted"	3978	2786	PCR/Sanger	-	0	0	**5/2** *	**1/1**	**2/2**	0
*B*. *lapponicum*	3974	1604	PCR/Sanger	**3**	**1**	**4/0**	**1**	**4/1**	**2/0**	0
*B*. *orion*	2831	3079	PCR/Sanger	0	0	**1/0**	**2/1**	0	0	0
*B*. sp. nr. *transversale*	3021	3205	PCR/Sanger	-	0	**4/1**	-	0	**2/1** *	-
*B*. sp. nr. *transversale*	3021	3205	Illumina	0	0	**4/1**	**60/3**	0	**2/1** *	-
*Lionepha chintimini*	4002	4059	PCR/Sanger	-	0	0	**22/10** *	**1/0**	**1/0**	**2/0**

All comparisons are between the IlluminaMerged sequences of museum specimens and sequences of likely conspecific specimens. Comparison samples were sequenced using PCR/Sanger sequencing except for the second comparison between sample 3021 and comparison sample 3205; for that comparison, the comparison sequences are from a *de novo* assembly. Under “Differences between Samples”, the number before the "/" is the number of unambiguous differences between the sequences; the number after the "/" is the number of differences that resulted in a non-synonymous substitution of the amino acid.

* indicates one or more substitutions are near the end of a portion of the museum sequence.

**Fig 15:**
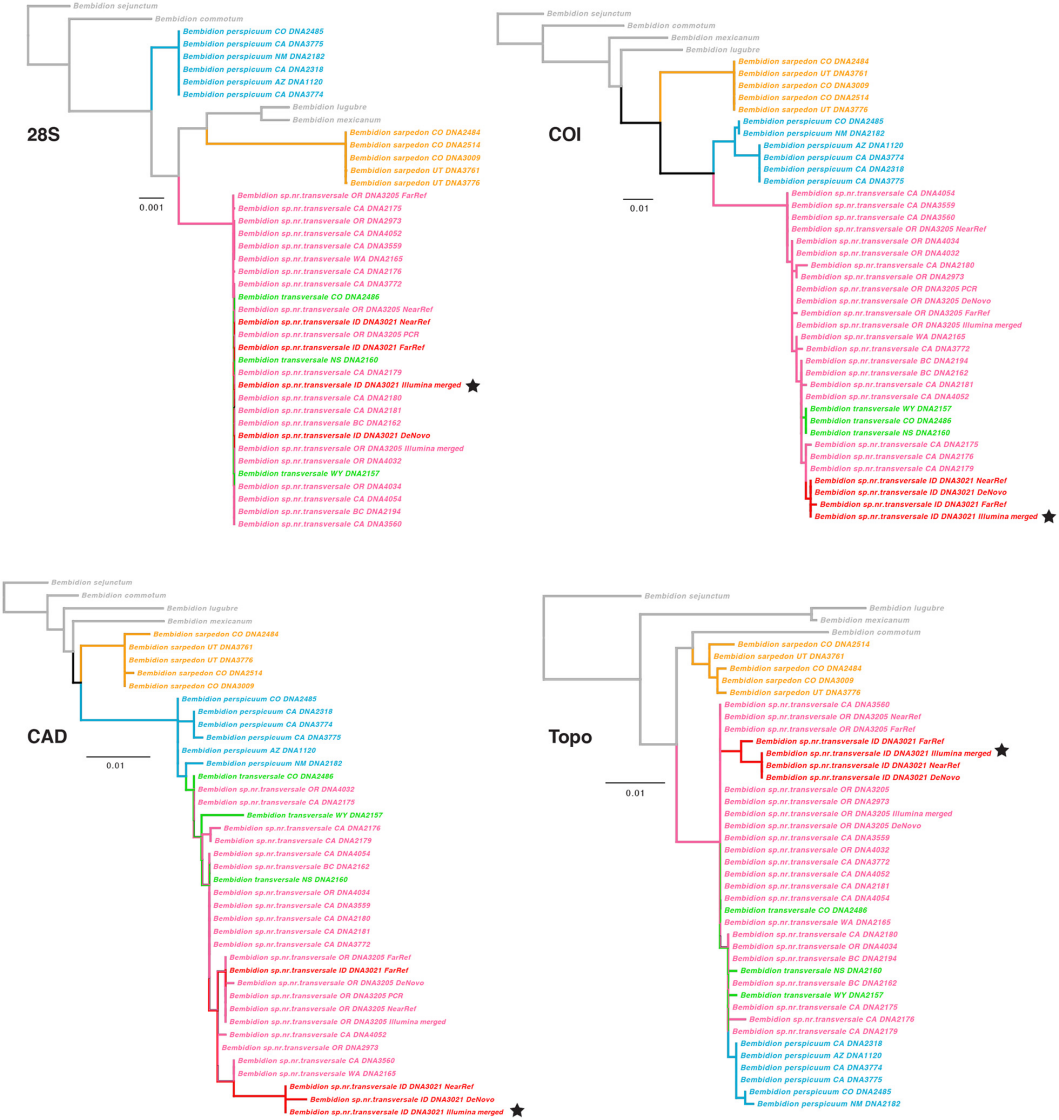
Four maximum likelihood gene trees for the *Bembidion transversale* group. The placement of DeNovo, NearRef, FarRef, and IlluminaMerged sequences of museum specimen *Bembidion* sp. nr. *transversale* 3021 is shown in a matrix of conspecific specimens and close relatives in the *transversale* species group. The museum specimen assembly sequences are shown in red with the IlluminaMerged sequence marked with a black star. All other specimens are colored by species.

In the single-gene analyses of the *transversale* species group, museum specimen *B*. sp. nr. *transversale* 3021 was recovered with other members of its species in all four genes available (28S, COI, CAD, and Topo; Fig 15). In 28S, the DeNovo, FarRef, NearRef, and IlluminaMerged assembly sequences from the museum specimen were identical to sequences generated by Sanger sequencing of extractions from conspecific specimens preserved in high-percentage ethanol. In COI all four assembly sequences from the museum specimen form a clade that is nested within other *B*. sp. nr. *transversale* specimens. It appears the formation of this clade is due to a single position at which the HTS sequences have a unique, synonymous base difference. In CAD, the FarRef sequence fell out in a clade with sequences from the reference specimen *B*. sp. nr. *transversale* 3205, while the other sequences fell out as a clade that was notably differentiated (having unique bases at four positions). These four bases are flanking a region for which the Illumina sequences have missing data. In Topo, all sequences from the museum specimen assemblies were inferred as a clade nested within other *B*. sp. nr. *transversale* specimens, however this clade was notably differentiated from the other *B*. sp. nr. *transversale* sequences in the matrix. Four positions have character states that were unique in one or more Illumina assembly sequences. Two bases flank a region of missing data as in CAD, one base is unique to all Illumina sequences but not near missing data, and the remaining base was only unique in the DeNovo sequence.

### Factors affecting success of gene recovery

**Fig 16:**
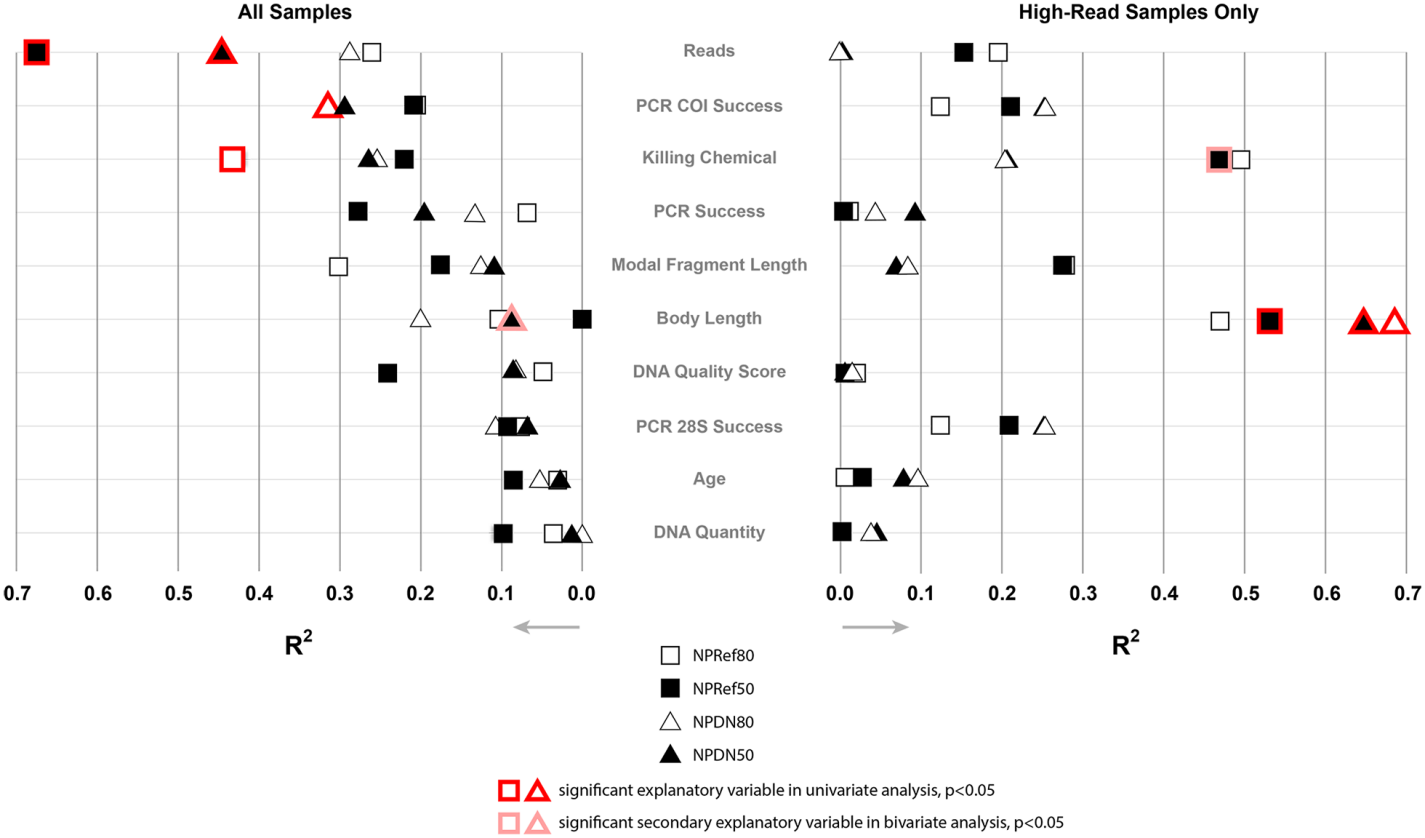
Squared correlation coefficients from univariate linear regression analyses between success measures and potential explanatory variables. Measures of success of acquiring protein-coding gene fragments are NPDN50 (*de novo* assembly, percent of gene fragments for which at least 50% of the bases were recovered), NPDN80 (same, but at least 80% of the bases), NPRef50 (reference-based assembly, percent of gene fragments for which at least 50% of the bases were recovered), and NPRef80 (same, but at least 80% of the bases). On the left are analysis with all samples included; on the right are analyses with only samples with more than 60 million reads included. Symbols outlined in red indicate that the correlation is significant in a single-variable analysis; symbols outlined in pale pink indicate that the correlation is significant as a secondary variable in a bivariate analysis. Note x-axis orientations are mirrored in the two graphs.

In univariate analyses of all samples, the number of reads, PCR COI success, and killing chemical all showed significant correlation with at least one measure of success (Fig 16); in some bivariate analyses, body length was significant as a secondary explanatory variable. In the analysis restricted to samples with large numbers of reads, body length was the only significant explanatory variable in univariate analyses for three of the four measures of success, with killing chemical being an additional significant explanatory factor for NPRef50 in bivariate analyses. In particular, high success was correlated with high number of reads, success at COI PCR, being killed in high concentrations of ethanol, and small body size. Curiously, two variables one might have presumed to be relevant to success at sequencing, age of specimen and total quantity of DNA, showed the weakest correlations (Fig 16).

## Discussion

Modern HTS methods are designed to sequence small DNA fragments, and for this reason they have revolutionized sequencing of the fragmented DNA that results from non-optimal storage conditions [16,56]. Archaeological or paleontological specimens that are thousands of years old are especially challenging, as they have highly fragmented DNA and have been exposed to potential DNA contamination from the environment. Obtaining sequences from these specimens (especially non-mitochondrial elements) often requires an extensive sequencing investment (perhaps billions of reads) [17,19], reference genomes of closely related extant species [57,58], or the development of hybrid capture probes to enrich target regions [59–61]. Reference-based assembly and targeted enrichment allow for efficient use of the limited DNA fragments present in the sample, as well as aid in avoiding DNA from contaminants. Similar techniques have been used on museum specimens that were not specifically preserved for DNA study [7,16,22,62–64]. The protected environment of museums presumably lessens the risk of contamination relative to ancient samples found in nature, and thus the main advantages of reference-based assembly and targeted enrichment are in their ability to enhance the signal.

For many groups of organisms, sequencing ancient or old specimens cannot rely on the signal enhancement enabled by reference-based assembly or targeted enrichment, as the relevant genomic resources are lacking. For example, in carabid beetles related to *Bembidion*, there are sufficient genomic resources for reference-based assembly, but not for design of a probe set for targeted enrichment; for other carabids, and most tenebrionid beetles, neither is possible with currently available resources. Even without reference-based assembly or targeted enrichment, loci of interest may still be recovered using *de novo* assemblies of low coverage genome sequencing projects (sometimes called “genome skimming”). This has been successfully demonstrated in few recent studies of museum specimens stored in vertebrate collections and herbaria [5,65–67]. In this study we have demonstrated the utility of genome skimming from low coverage HTS data in recovering low-copy nuclear protein-coding genes and multi-copy loci from small-bodied arthropods, which represent the vast majority of diversity stored in many museum collections.

### Success at sequencing museum insects

The extent of our success in recovering many low-copy nuclear protein-coding targets for all samples in this study was unexpected. We were hopeful that we would recover mitochondrial and ribosomal genes for all specimens we sequenced, especially given previous success with high-copy genes [20,22], but we expected that few low-copy genes would be recovered for older, smaller specimens with minimal, low quality DNA. Our unexpected sequencing success is best illustrated by *B*. *lachnophoroides* 3022, a 56-year-old specimen, 4.4 mm in length, with 9.9 ng of total DNA in the extraction and a modal fragment length of less than 100 bases (Fig 4). This old, small specimen performed comparatively well in the CEGMA analysis (Table 11), in recovery of the 67 Regier *et al*. nuclear protein-coding gene fragments (Figs 6 and 7), and in accurate recovery of the seven focal genes (Tables 13 and 14). For this specimen, our *de novo* assembly recovered about 26,400 of the approximately 41,900 nucleotides in the 67 Regier *et al*. gene fragments (S7 Table), or approximately 63% of all nucleotides; recovery in the reference-based assembly increased to 70% of all nucleotides (S8 Table).

Our finding that *de novo* assemblies allowed for partial recovery of many target loci for almost all museum specimens with greater than 60 million reads (Figs 6 and 7) demonstrates the application of this approach for specimens that lack genomic resources required for reference-based assembly. In groups such as beetles, we have limited sequenced genomes or transcriptomes to serve as scaffolds, in contrast to the rich selection of genomic resources that have been used to sequence ancient DNA of groups such as vertebrates. For many non-model, non-vertebrate taxa, *de novo* assembly will be the only viable option, as it was with our museum specimen *Lagriinae* n. gen. KK0290, for which no genomic data exist for closely related taxa.

We found reference-based assembly to be effective at recovering targets for specimens for which we had relatively few reads [68,69]. The specimens that failed to recover low-copy nuclear protein-coding genes in *de novo* assembly all showed at least partial recovery for many genes in the reference-based assembly (Figs 6 and 7, S8 Table). The increase in success of reference-based assembly over *de novo* assembly was especially notable for the two specimens (*B*. sp. nr. *transversale* 3021 and *B*. *cf*. “Desert Spotted” 3978) that were most closely related to the reference (*B*. sp. nr. *transversale* 3205) (S8 Table, S10 Fig). Although reference-based assembly generally performed better than *de novo* assembly, it performed slightly worse in the nuclear ribosomal genes (Fig 7). The reduced recovery occurs in the highly variable regions of these two genes, which contain numerous indels across the sampled taxa. It is possible that a more closely related reference, with fewer indels relative to the sequenced taxon, would have yielded more accurate reference-based data for the nuclear ribosomal genes.

Our success is presumably influenced by the genome sizes of these beetles. Although the genome sizes of carabids related to *Bembidion*, *Lionepha*, and *Bembidarenas* have never been measured, distantly related carabids that have been measured have genome sizes between 660 Mb and 1.0 Gb [70]. Similarly, the most closely related genus to Lagriinae n. gen. whose genome has been measured is *Cossyphus*, with a genome size of 479–558 Mb [71]. Museum specimens with larger genomes will require a larger number of sequencing reads to be as successfully sequenced as those with smaller genomes. Most *Bembidion* species have very similar karyotypes [72], consistent with similar genome sizes throughout the genus; thus, the variation we do see in HTS success in *Bembidion* is not likely a result of large variation in genome size.

### Accuracy of sequences from museum specimens

Sequences obtained from HTS of museum specimens might be inaccurate for several reasons. DNA in the specimens might have undergone degradation over the years, with resulting base changes [7,73–75]. Contaminant DNA might also be present in the sample, which might be sequenced instead of the original specimen’s DNA [20,76–78]. Although museum specimens are less likely to be subject to the same barrage of potential environmental contaminants as those present in nature, saprophytes (e.g., fungi or bacteria) are a possible source of contaminant DNA if they grew within the organism as it decayed before drying. Organisms that feed on museum specimens (e.g., dermestid beetles) might also provide DNA contaminants to the sample. The degradation of DNA into small pieces may lead to assembly problems with HTS, as some target regions may not be well represented by fragments of a sufficient length for sequencing, resulting in reduced coverage of those regions and thus poor assemblies [79]. And finally, HTS in general can have inaccuracies [80], even for fresh specimens, if low coverage fails to expose sequencing errors that would be recognized if coverage were higher, or leads to calling a site homozygous that is actually heterozygous [81–83]. For these reasons, tests of the accuracy of museum specimen sequences are of value.

We relied on indirect means to measure the accuracy of our HTS sequences. An ideal method of measuring errors in sequences from museum specimens would use samples that were divided into two pieces, with one piece preserved to ensure maintenance of high-quality DNA, and the other piece subject to treatments similar to those experienced by dried museum specimens. A comparison of results from the split sample would enable detection of degradation and sequencing errors. As we do not have available samples of small insects treated in this way, we relied on three complimentary methods to determine accuracy: (1) calculation of base differences between HTS sequences and PCR/Sanger sequences from the same museum specimen, (2) calculation of base differences between HTS sequences of a museum specimen and sequences from fresh, conspecific specimens, (3) phylogenetic tests that examine the placement and branch lengths of the HTS sequences in the context of related species.

#### Base differences from PCR/Sanger sequences of the same specimen

In our exploration of PCR/Sanger sequencing of the same museum specimens subject to HTS, we found most HTS sequences exactly matched the PCR sequences, providing some evidence for the accuracy of the HTS data. When discrepancies were present, they were most often a result of an ambiguous base in the PCR sequence, in contrast to an unambiguous base in the Illumina data (S3 Table). In cases in which the PCR/Sanger data showed clear evidence of heterozygosity, the discrepancy may be explained by our HTS assemblies not having sufficient depth of coverage to detect both bases at those sites. The four bases in the HTS sequences that were unambiguously different from the PCR sequences may have resulted from changes during library amplification, or sequencing errors, or from some other cause. In general, examination of discrepancies between HTS sequences and PCR/Sanger sequences from the same museum specimen can provide only a partial test of the accuracy of the Illumina approach: it can detect problems specifically associated with low-coverage HTS data, but it cannot detect whether any general degradation has occurred to which PCR/Sanger sequencing would also be sensitive.

#### Base differences from conspecifics

PCR/Sanger sequences of well-preserved conspecific specimens would not be subject to the degradation of an old museum specimen, and thus provide a potentially better comparator for judging HTS museum sequence accuracy. Ribosomal genes and COI appeared quite accurate by this measure (Table 15). Of the few nuclear protein-coding genes that showed discrepancies between HTS sequences and those from PCR/Sanger of conspecifics, the most divergent had differing results in the phylogenetic tests: the museum sequence of *Lionepha chintimini* 4002 ArgK was inferred where expected in the phylogenetic analysis (S6 Fig), but *B*. sp. nr. *transversale* 3021 ArgK was not (Fig 12). We conducted additional assemblies (not shown) using more thoroughly trimmed reads (by removing at least 10 bases from each end of each read, in addition to the default trimming based upon quality scores) to see if more conservative sequences would remove these discrepancies [38,73]. Although the stricter trimming regime reduced the length of some of the fragments (e.g., CAD for *Bembidarenas* 3983 was reduced from 362 bases to 237 bases), almost all of the discrepancies remained. The greatest changes were in ArgK, with the sequence for *B*. *cf*. “Desert Spotted” 3978 being reduced by 27 bases, with the consequent removal of four of the five discrepancies, and the sequence for *Lionepha chintimini* 4002, which after stricter trimming showed only 15 discrepancies (eight of which were non-synonymous), seven less than with the original trimming.

#### Phylogenetic tests of accuracy

Our examination of HTS sequences in the phylogenetic context of other specimens of the same and closely related species allows a more nuanced understanding of possible sequence errors than simple comparison of base differences. Without a phylogenetic context, it is difficult to judge the importance of discrepancies between a museum specimen and a conspecific, fresh specimen; any difference might simply be the result of intraspecific variation. In a phylogenetic context, with museum specimen sequences analyzed with very closely related sequences including several from putative conspecifics, inaccuracy of museum sequences can be easier to detected based upon outliers in branching pattern and branch lengths. Our finding that assembly sequences from museum specimen *B*. sp. nr. *transversale* 3021 were inferred with conspecifics in all four genes analyzed (Fig 15) is evidence for the general quality of the data. However, CAD and Topo showed unusual branch lengths relative to conspecific sequences (Fig 15), due to the presence of unique bases flanking regions of missing data in the HTS museum specimen data. Later examination showed that differences in CAD are a result of a less-than-optimal MAFFT alignment that could have been corrected through hand-curating. For Topo, the differentiating bases may represent sequencing errors near the ends of reads that persisted in the final assemblies due to low coverage.

If no well-preserved conspecifics are available, phylogeny inference to test for placement within predicted groups is useful as an additional means of testing the accuracy of sequences obtained from museum specimens. For the museum specimens we sequenced, morphological evidence provided predictions regarding taxa to which the specimens are closely related, and for the most part these predictions were upheld. Our analyses show that the sequences are generally accurate enough to place the sequences in their prediction group with branch lengths that are consistent with what would be expected from accurate, non-contaminant sequences.

Although the potential errors we did detect were not sufficient to produce placement failures for most sequences in our phylogenetic testing, they nonetheless expose potential minor quality issues with low-coverage museum specimen sequences that should be anticipated during data analysis. The instances of sequence errors we described above are expected to decrease as the number of reads increases for a given specimen [82,83].

Congruence between phylogenetic results from different gene regions can be used to test for accuracy for those specimens without sufficiently detailed predictions about their phylogenetic placement. For example, even if there were no predictions about the placement of *Bembidion lachnophoroides* 3022, the consistent placement of HTS sequences as sister to *Bembidion ruficolle* in all seven genes (S7 Fig) itself is an indication of accuracy, as such consistency would be unlikely if the sequences were error-ridden. Congruence between genes cannot rule out contamination from another taxon’s DNA, however, and that possibility may need to be explored if one is relying on congruence to test for accuracy.

#### Depth of coverage

A low-coverage sequencing approach, in which the number of reads relative to genome size leads to low depth of coverage at a given locus, reduces the per-specimen sequencing cost. However, it also raises concern over the accuracy of assemblies due to sequencing errors in low coverage regions being incorporated into final assemblies [81,82]. In the present study, the depth of coverage of nuclear protein-coding targets in our focal genes was less than 6X for all museum specimens that we sequenced. Although this coverage depth would be considered insufficient coverage for genomic applications of fresh specimens [69,79], this coverage depth is comparable to other studies sequencing museum specimens [5,16,23,84], and our tests of phylogenetic placement suggest that generally accurate sequences can be obtained from museum sequences even at this coverage depth. The balance between low cost per specimen and the depth of coverage will be dictated by the data quality required by the planned application of the data. We emphasize that museum specimens will likely require more reads than well-preserved specimens to achieve similar coverage depth. This conclusion is supported by our comparison of coverage depth between museum and reference specimens (Table 12), and other studies [16,22,84], which all showed roughly half the depth of coverage as would have been expected in fresh specimens.

#### Choosing the *de novo* assembly sequence

For many of our museum specimens and genes, multiple contigs were returned in our BLAST probing of the *de novo* assemblies, requiring us to develop a protocol for selecting the sequence most likely to be the accurate ortholog. Multiple contigs in a *de novo* assembly were far more prevalent in nuclear ribosomal genes and COI than nuclear protein-coding genes, and were often highly varied, leading to unexpected phylogenetic placement in several museum specimens (S5 Fig). The multiple contigs in nuclear protein-coding genes were often nearly identical sequences; however, there were instances where selecting the wrong contig would lead to inaccurate data as judged by our phylogenetic tests (for example ArgK in *L*. *chintimini*, or CAD in *B*. “Arica”). In general, our criteria for selecting a single likely orthologous sequence was critical in COI and the nuclear ribosomal genes for improving accuracy. Our subsequent phylogenetic analysis of only the chosen sequences provides good evidence that the criteria we used in filtering out multiple contigs were effective.

#### Effects of method of assembly

For most museum carabids and most genes, the two reference-based sequences fell in a clade with the *de novo* sequence, or in a clade with the *de novo* sequence and other conspecific sequences, suggesting that our reference-based assemblies are not generally affected by serious assembly biases. However, there were 16 examples in which at least one of the assemblies produced a sequence that failed the phylogenetic test; for four of these (all for the gene 18S), one of the other assemblies produced a sequence that did pass the phylogenetic test. These failed placements highlight the value of conducting multiple assemblies in order to verify consistent placement of sequences from both *de novo* and reference-based approaches and more readily identify potential issues with sequence quality.

The age of the split between a sequenced taxon and its reference varied, and the consequent variation in divergences might be expected to affect assemblies. We estimate that the ancestors of *Asaphidion yukonense*, our far reference, diverged from those of the *Bembidion* museum samples at least 34–49 million years before present, based upon the presence of *Bembidion* in Baltic amber [85], and the estimated age of Baltic amber [86,87]. The *Lionepha*–*Asaphidion* and *Bembidarenas—Asaphidion* divergences would be older, but of unestimated ages. The near references were all much more closely related to the sequenced taxon, with the expected divergence times being much less. However, as noted, in general we detected only slight differences in the assemblies as a function of the reference used. This is consistent with the results from other comparisons of multiple references of varying evolutionary distances [88].

We found value in merging our three assemblies for each gene into a single sequence, as did Marchant [89]. This allowed us to objectively retain more data than if we had chosen to accept sequences from a single assembly approach for all targets, while still retaining any variation between assemblies. The analyses that included the IlluminaMerged sequence suggest that our approach was in general successful (Table 14, S8 and S9 Figs), especially for the concatenated data (Fig 14). However, of the 16 failures (among the 82 cases) in which at least one of the three assemblies of single genes failed to pass the phylogenetic test, only two failures were alleviated by forming the merged Illumina sequence. Nonetheless, we recommend this approach if both reference-based and *de novo* assemblies can be conducted.

### Predicting sequencing success of candidate specimens

As resources may limit the number of library preparations and high-throughput sequencing that can be performed, having some means to predict which museum specimens would most likely yield desired DNA sequences would be ideal. For ancient DNA, qPCR [90,91], measurement of amino acid racemization [92], and histological examination of tissue [93] have been used to predict DNA sequence recovery success. However, these methods may not be practical in a museum setting where dozens or even hundreds of specimens are at hand. For these situations, it would be desirable if we could predict sequencing success using simpler methods.

The prediction should be based upon some easily measured property of a specimen (such as age, size, preservation method, or success of PCR amplification; [94]). Some properties that might be relevant predictors will not be known for all specimens. For example, genome size is expected to be correlated with success, with organisms with larger genomes expected to have lower success under the same conditions. However, as genome size is still unknown from many taxa (including the taxa included in this study), it may not be available as a predictor.

One value we expected to be a predictor of success was total DNA contained within the specimen. The low cost of DNA extraction, availability of non-destructive extraction protocols [11,95], and simple DNA quantification methods make this a viable exploratory measure for most specimens. In the present study, we did not attempt library preparation for specimens with less than 9.9 ng of total DNA. However, all of our samples with this amount or more of DNA, and for which we sequenced at least 60 million reads, were generally successful at yielding numerous genes through HTS. Against expectation, our regression analyses did not reveal total DNA to be a significant predictor of sequencing success.

We also did not find a statistical correlation between sequencing success and the DNA quality metric we used to categorize the museum specimens. For example, *B*. *lachnophoroides* 3022 scored in the lowest DNA quality category (Category 1), yet performed better in gene recovery than many samples with more DNA that was less fragmented (Fig 7, Tables 4 and 12).

If sufficient DNA is present for library construction, our statistical analysis found the most consistent factor affecting gene recovery success to be number of reads. While this is not an intrinsic property of the organism, it is a factor that can be adjusted by allocating additional reads for a specimen during initial sequencing, or adding more reads in future sequencing after the first round of sequencing has been evaluated. We note that many methods of library preparation generate sufficient library to undergo multiple sequencing reactions, and in the present study sufficient library remains for all samples to be re-sequenced many times.

In addition to number of reads, PCR COI success and killing chemical showed some correlation with sequencing success, suggesting that these factors may be used as predictors of success when considering candidate specimens (Fig 16). However, for our analysis restricted to those samples with more than 60 million reads, there was a significant correlation between small body size and sequencing success. A similar correlation between PCR/Sanger sequencing success and small size of ethanol-preserved spiders was observed by [94]. For the beetles we studied, it is possible this may be a result of small-bodied specimens drying out faster after mounting, leading to faster stabilization of DNA than specimens with a longer drying time. We emphasize, however, that because of low sample sizes, and non-random sampling of specimens, our results should be viewed as tentative, rather than as definitive evidence of any particular metric predicting success.

## Conclusion

In this study we demonstrate the utility of low-coverage HTS in recovering low-copy nuclear protein-coding genes from dry-mounted museum insects with diverse preservation histories and a range of DNA quantity and quality. We were able to confirm general accuracy of the acquired data, with some exceptions. The ability to recover low-copy number genes opens the door for many research questions that require data from nuclear protein-coding genes, and will provide a vital complement to high-copy number (ribosomal and mitochondrial) genes previously recovered from insect specimens in museums [20,22]. Our success has implications for molecular studies on small-bodied arthropods across many fields for which obtaining new material is challenging or costly, or for which obtaining sequence data from rare specimens is desirable.

We used standard DNA extraction kits and standard shotgun sequencing. Use of extraction kits geared for lower quantities of degraded DNA would likely improve success. In addition, sequencing approaches that start with targeted enrichment have proven successful with ancient DNA [60,61,63,64], and they will likely also be valuable with small-bodied museum specimens. The advantage for museum specimens will be increased depth of coverage per locus, rather than avoiding contamination, which is a greater concern for ancient DNA in the environment than for a specimen in a protected museum environment. The greater depth of coverage of targeted genes for a given total number of reads will allow for higher quality sequences at much lower cost per specimen, especially if the organism has a large genome.

The overall accuracy of the sequences we obtained from museum specimens suggests that HTS and the assembly methods we used can be trusted to generate data sufficiently accurate for many research purposes. Inaccuracies may affect a research study’s results, but to differing levels depending upon the nature of the study. For phylogenetic research, the results of our tests suggest that the sequences can be accurate enough to infer the correct relationships of the specimens, at least if multiple genes are included in the analysis. Nonetheless, we recommend that tests of accuracy be conducted when possible.

Use of HTS to acquire DNA sequences from museum specimens will be the only option for extinct taxa, or may alleviate the need for costly fieldwork. Recovering fresh material for some of the taxa included in this study would have required a significant monetary and logistical investment, with a real possibility of failure to collect the target taxa in the end. For example, DRM has attempted to collect live *Bembidion lachnophoroides* specimens on four separate expeditions, and failed each time; our success at obtaining extensive genomic data from a 56-year-old specimen lessens the need for additional attempts. That said, some research questions will not be answerable with only the material currently available in museums, and new fieldwork will be necessary.

Future studies should explore factors that affect sequencing of museum specimens using specimens with more accurately known histories and a larger sample size than was available to us in our “found experiment”. If easily measured properties of the specimens could be shown to be accurate predictors of sequencing success, then genetic resources available in museum collections could be studied more efficiently. To enable this, it would be valuable for museums to gather data about the history of their specimens, to help guide DNA research. For example, collectors who have contributed material to a museum could be queried about their field methods, including the chemicals they used to kill and preserve specimens. A study of the effects of various museum practices would also be valuable, as it would help museums determine best practices to preserve the valuable DNA sequences in specimens. For example, having air conditioning, which would reduce the ambient temperature in collections, may be important in warmer climates, as there is an expectation that degradation of DNA will be more rapid with higher temperatures [15]. Some museum practices would surely damage most DNA irreparably (such as removal of soft tissue with potassium hydroxide), and the potential to damage DNA of unique or rare specimens should be considered before such practices are employed; the effect of other practices (such as short-term exposure to hot water to relax specimens) on DNA are not well known, and should be studied.

As DNA sequencing methods improve, costs continue to drop, and library preparation methods are further optimized to handle smaller amounts of starting DNA, accessing the DNA in museum specimens will become increasingly easier, further enhancing the value of natural history collections as an indispensable resource for genomic data.

## Supporting Information

S1 Fig Electropherograms of DNA extracted from older museum specimens that did not undergo library preparation. Pale spikes at 35 and 10380 bases represent standards included in each analysis.
(PDF)

S2 Fig Electropherograms of DNA extracted from younger museum specimens that did not undergo library preparation. Pale spikes at 35 and 10380 bases represent standards included in each analysis.
(PDF)

S3 Fig Maximum likelihood trees for individual gene datasets of Lagriinae. The museum specimen is marked with a star symbol. The branches and taxon names of Lagriinae n. gen and its predicted closest relatives (based on morphological characters) are colored in blue. No sequences for *wg* were recovered from Lagriinae n. gen.
(PDF)

S4 Fig Majority rules consensus trees of maximum likelihood bootstrap analyses for concatenated and single gene datasets of Lagriinae. Bootstrap support values given below the branch at nodes with at bootstrap values of at least 50. The museum specimen is marked with a star symbol. The branches and taxon names of Lagriinae n. gen and its predicted closest relatives (based on morphological characters) are colored in blue. No sequences for *wg* were recovered from Lagriinae n. gen
(PDF)

S5 Fig Maximum likelihood gene trees of carabids from seven focal genes with all contigs included from the *de novo* assembly. Each tree includes all contigs returned from our BLAST searches for target genes within HTS museum specimen assemblies, prior to our selecting the chosen contig for that specimen. The contig that was chosen through our criteria outlined in the text is marked with a star symbol.
(PDF)

S6 Fig Maximum likelihood gene trees of carabids from seven focal genes and “Three Separate” assembly sequences. The placement of the DeNovo, NearRef, and FarRef sequences is shown relative to their prediction groups. Each prediction group is indicated with a unique color for branches and taxon names of all specimens in the prediction group.
(PDF)

S7 Fig Majority rule consensus trees with bootstrap values of carabids from seven focal genes and “Three Separate” assembly sequences. The placement of the DeNovo, NearRef, and FarRef sequences is shown relative to their prediction groups. Branches and taxon names of all specimens in the prediction group are indicated with a unique color.
(PDF)

S8 Fig Maximum likelihood trees of carabids from seven focal genes and IlluminaMerged sequences. The placement of the IlluminaMerged sequences is shown relative to their prediction groups. Branches and taxon names of all specimens in the prediction group are indicated with a unique color.
(PDF)

S9 Fig Majority rule consensus trees with bootstrap values for carabids from seven focal genes and IlluminaMerged sequences. The placement of the IlluminaMerged sequences is shown relative to their prediction groups. Branches and taxon names of all specimens in the prediction group are indicated with a unique color. Bootstrap support values are given under nodes for branches that are supported with a bootstrap value greater than or equal to 50.
(PDF)

S10 Fig Lengths of reference-based assembly relative to *de novo* assembly of 67 low-copy nuclear protein-coding gene fragments in HTS museum specimens. Values shown are the percent difference of the length of reference-based contig minus the length of the corresponding *de novo* contig. Positive values (blue) indicate the reference-base contig was longer, and negative values (red) indicate the de novo contig was longer. Gene fragments are ordered by average recovery as measured across both *de novo* and reference-based assemblies. Gene abbreviations are those used in Regier *et al*. [25]. Specimen abbreviations: Lag: Lagriinae n. gen. KK0290, subf: *Bembidion subfusum* 3977, snt1: *B*. sp. nr. *transversale* 3021, Lchi: *Lionepha chintimini* 4002, lach: *B*. *lachnophoroides* 3022, Bdrs: *Bembidarenas* 3983, ori1: *B*. *orion* 2831, inu1: *B*. "Inuvik" 3285, lapp: *B*. *lapponicum* 3974, aric: *B*. "Arica" 3242, dspt: *B*. *cf*. "Desert Spotted" 3978, mus: *B*. *musae* 3239, inu2: *B*. "Inuvik" 3984, ori2: *B*. *orion* 3079, snt2: *B*. sp. nr. *transversale* 3205. Four specimens with less than 34 million reads have specimen abbreviation and age shown in gray.
(PDF)

S1 Methods Details of methods used in this study. (DOCX)

S1 Table Additional details of specimen provenance and extracted tissue. (DOCX)

S2 Table PCR primers used in this study. (DOCX)

S3 Table Comparison of Sanger sequenced fragments and corresponding Illumina sequenced region for museum specimens, gene. (DOCX)

S4 Table Number of candidate contigs for each gene in the *de novo* assemblies chosen for subsequent analyses. (DOCX)

S5 Table Preservation and collection data for Tenebrionidae sampled for PCR and Sanger sequencing. (DOCX)

S6 Table Thermocycler profiles used in PCR amplification of focal genes. (DOCX)

S7 Table Proportion of recovered bases from 67-gene set: *De novo* assemblies. (DOCX)

S8 Table Proportion of recovered bases from 67-gene set: reference-based assemblies. (DOCX)

S9 Table Data used in regression study (DOCX)

S10 Table Gene fragments sampled for the phylogenetic analyses of Lagriinae. (DOCX)

S11 Table Ethanol-killed specimens sequenced using PCR amplification and Sanger sequencing. (DOCX)
