# Correction: Successful Recovery of Nuclear Protein-Coding Genes from Small Insects in Museums Using Illumina Sequencing

**DOI:** 10.1371/journal.pone.0151124

**Published:** 2016-03-07

**Authors:** The PLOS ONE Staff

The Dryad URL provided in the data availability statement is incorrect. The correct data availability statement is as follows: Raw reads for all museum and reference specimens are submitted to NCBI Sequence Read Archive (accessions SRR2939013– SRR2939027). Focal gene fragments recovered from the de novo assembly of Lagriinae n. gen. and those that were newly sequenced for the phylogeny of Lagriinae are deposited in GenBank (accessions KU233685-KU234083). Focal gene fragments from PCR/Sanger sequencing and the IlluminaMerged sequences of carabids are also deposited in GenBank (accessions KU233685-KU234083). The Tribolium castaneum and Bembidion sp. nr transversale query sequences used to probe our museum specimens for the 67 nuclear protein-coding gene fragments and all alignments used in phylogenetic analyses (including the DeNovo, FarRef, and NearRef sequences), as well as trees from the phylogenetic tests, are deposited in Dryad (data available from the Dryad Digital Repository: http://doi.org/10.5061/dryad.q7m07).

The numbering for the list that appears below the third paragraph of the “Assessing DNA quality of museum and reference specimens” section of the Materials and Methods is incorrect. The correct list is as follows:

0: No measurable DNA in the Qubit (which means total DNA was less than about 0.06 ng) and no identifiable deviation from the baseline in the fragment-length distribution curve (e.g., *Bembidion subfusum* 2494, in S1 Fig).
1: With measurable DNA, modal fragment length below 100 bases, but no fragments longer than 400 bases (e.g., Lagriinae n. gen., KK0290, in Fig 4).
2: 40–220 ng total DNA, modal fragment length between 50 and 190 bases, with 3–10% of the fragments longer than 500 bases.
3: 80–250 ng total DNA, modal fragment length between 200 and 220 bases, with more than 15% of the fragments longer than 500 bases.
4: Between 400 and 550 ng of total DNA, modal fragment length around 200–300 bases, with some fragments greater than 1000 bases (e.g., *Bembidion* “Arica” 3242, in Fig 5).
5: More than 2000 ng of total DNA, modal fragment length greater than 500 bases with many fragments greater than 1,000 bases (e.g., *Bembidion nesophilum* 3240, in S1 Fig).
6: Material killed and preserved in 100% ethanol, with abdomen removed to allow ethanol penetration, replacement of ethanol, and storage at -20°C. Although we did not measure fragment length distributions for these samples, we assumed the DNA to be well-preserved [28,29].

The image for Fig 2 is incorrect. Please see the corrected Fig 2 here.

**Fig 2:**
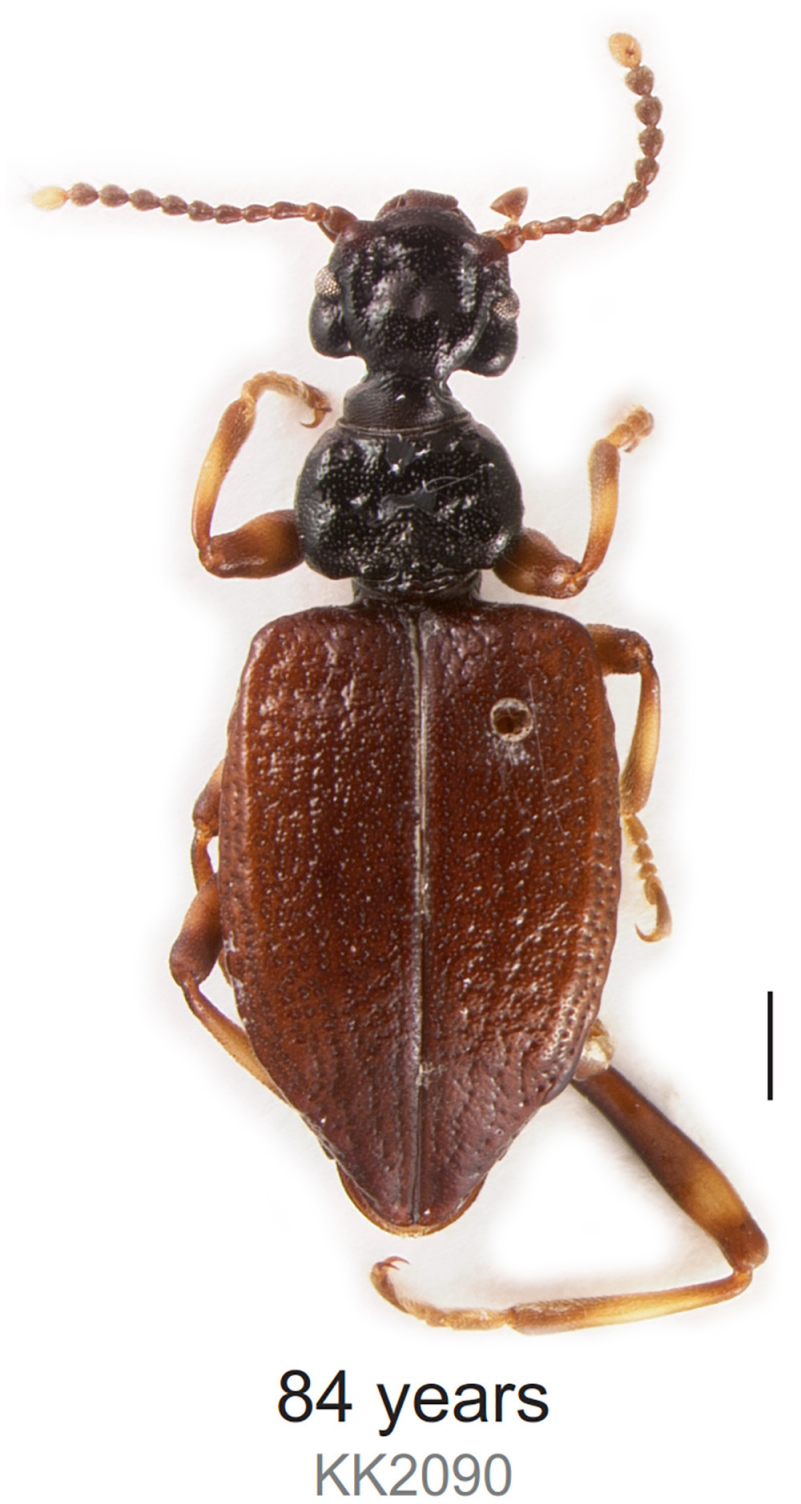
Habitus of Lagriinae n. gen. KK0290. Image taken after DNA extraction. Scale bar is 1 mm.

The image for Fig 14 is incorrect. Please see the corrected Fig 14 here.

**Fig 14:**
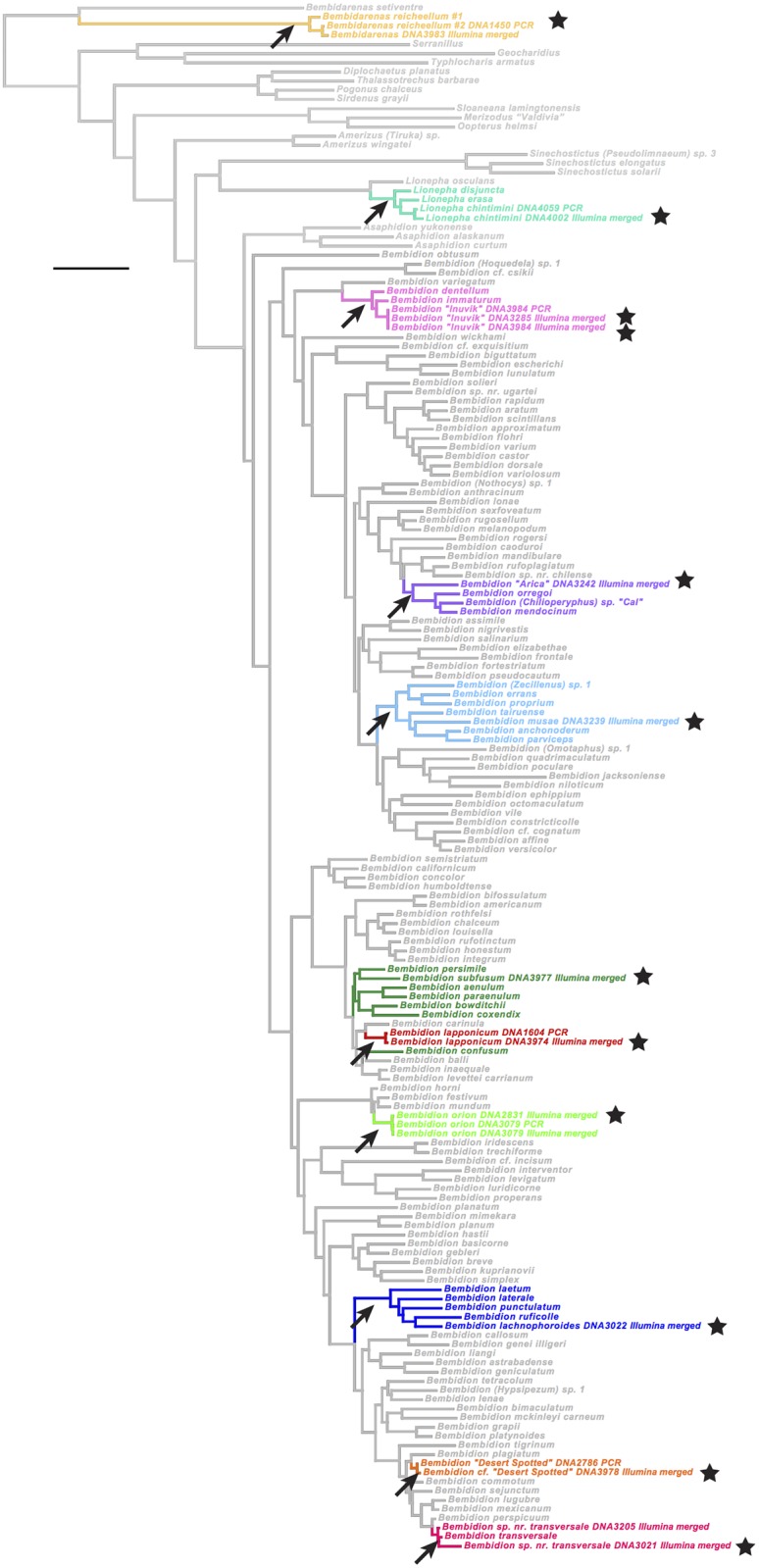
A maximum likelihood tree of carabids from seven focal genes and IlluminaMerged sequences. The placement of the IlluminaMerged sequences is shown relative to their prediction groups in a concatenated analysis of seven focal genes. Each prediction group is marked by a black arrow, and with a unique color for branches and taxon names of all specimens in the prediction group. The placement of each IlluminaMerged sequences is indicated with a black star.

The publisher apologizes for the errors.
