# Supplementary material for: Successful Recovery of Nuclear Protein-Coding Genes from Small Insects in Museums Using Illumina Sequencing
Source: PLoS One. 2015 Dec 30;10(12):e0143929. doi: 10.1371/journal.pone.0143929 (PMC4696846; doi:10.1371/journal.pone.0143929)
Supplement: S1 Fig — Pale spikes at 35 and 10380 bases represent standards included in each analysis. (PDF) [file pone.0143929.s001.pdf]

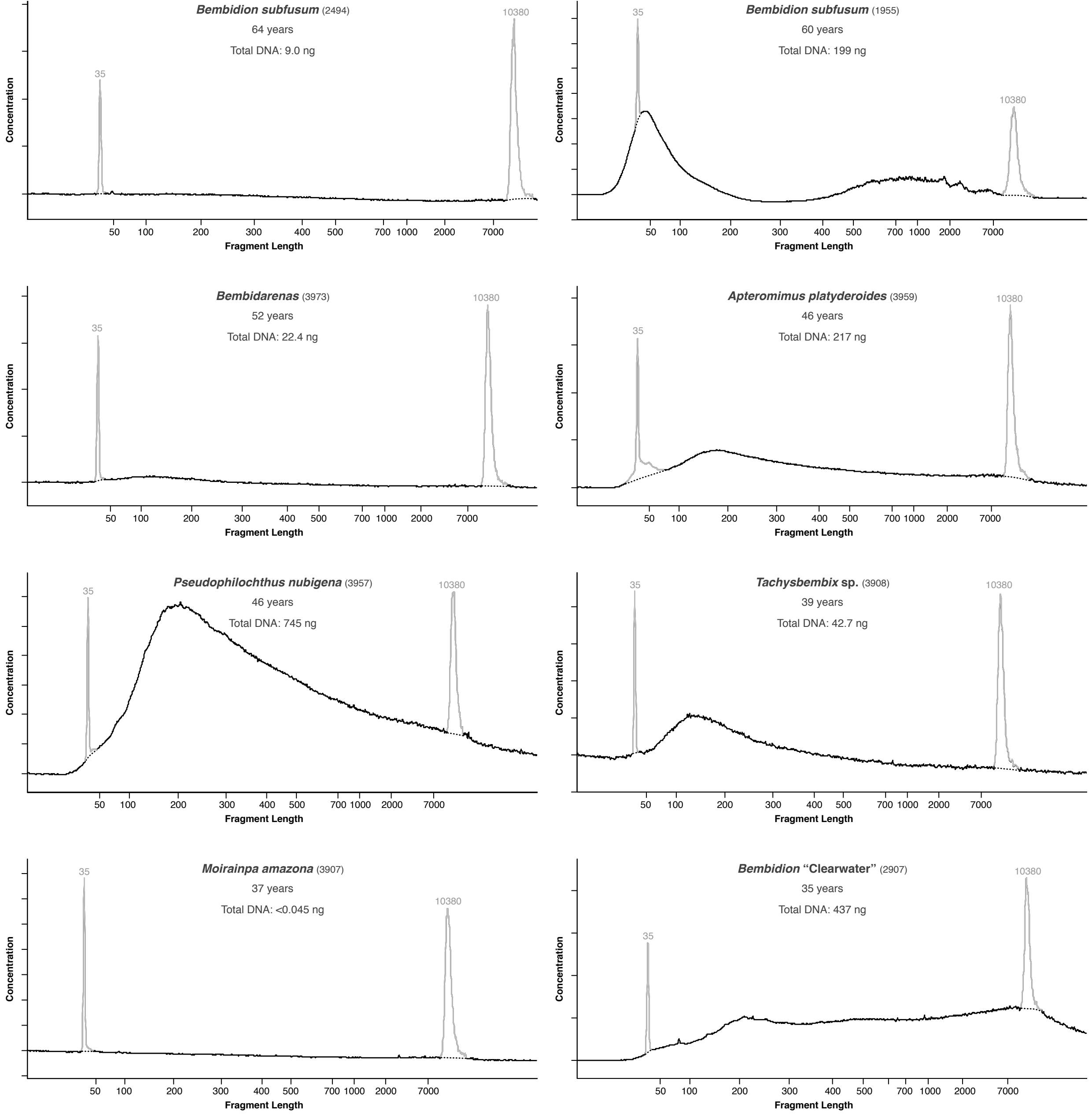

Bembidion subfusum (2494)
64 years
Total DNA: 9.0 ng
35
10380
Concentration
50 100 200 300 400 500 700 1000 2000 7000
Fragment Length
Bembidion subfusum (1955)
60 years
Total DNA: 199 ng
35
10380
Concentration
50 100 200 300 400 500 700 1000 2000 7000
Fragment Length
Bembidarenas (3973)
52 years
Total DNA: 22.4 ng
35
10380
Concentration
50 100 200 300 400 500 700 1000 2000 7000
Fragment Length
Apteromimus platyderoides (3959)
46 years
Total DNA: 217 ng
35
10380
Concentration
50 100 200 300 400 500 700 1000 2000 7000
Fragment Length
Pseudophilochthus nubigena (3957)
46 years
Total DNA: 745 ng
35
10380
Concentration
50 100 200 300 400 500 700 1000 2000 7000
Fragment Length
Tachysbembix sp. (3908)
39 years
Total DNA: 42.7 ng
35
10380
Concentration
50 100 200 300 400 500 700 1000 2000 7000
Fragment Length
Moirainpa amazona (3907)
37 years
Total DNA: <0.045 ng
35
10380
Concentration
50 100 200 300 400 500 700 1000 2000 7000
Fragment Length
Bembidion "Clearwater" (2907)
35 years
Total DNA: 437 ng
35
10380
Concentration
50 100 200 300 400 500 700 1000 2000 7000
Fragment Length
