# Supplementary figures and images for: Successful Recovery of Nuclear Protein-Coding Genes from Small Insects in Museums Using Illumina Sequencing
Source: PLoS One. 2015 Dec 30;10(12):e0143929. doi: 10.1371/journal.pone.0143929 (PMC4696846; doi:10.1371/journal.pone.0143929)

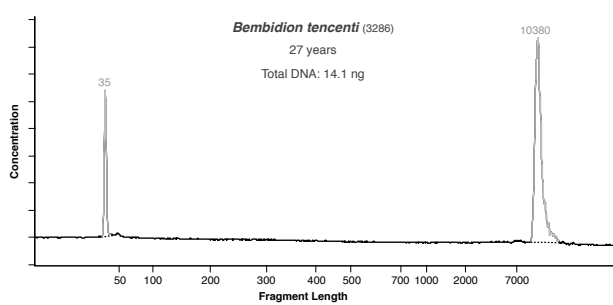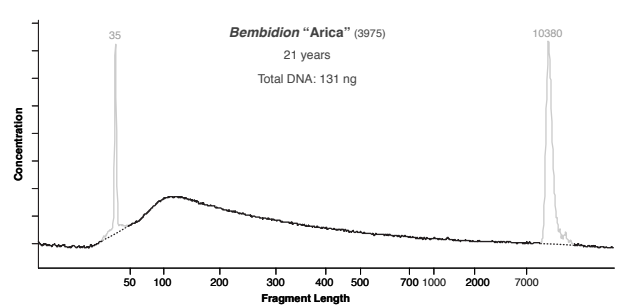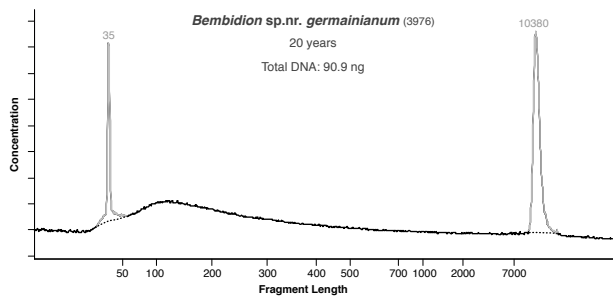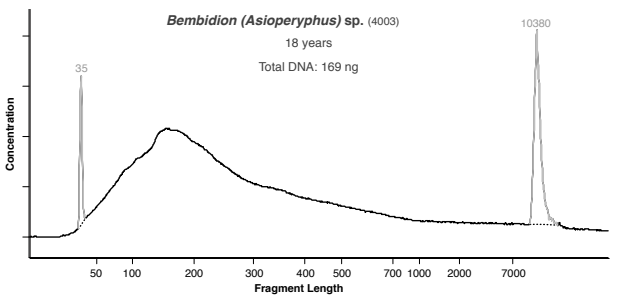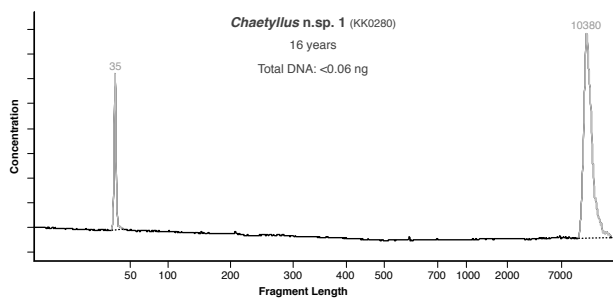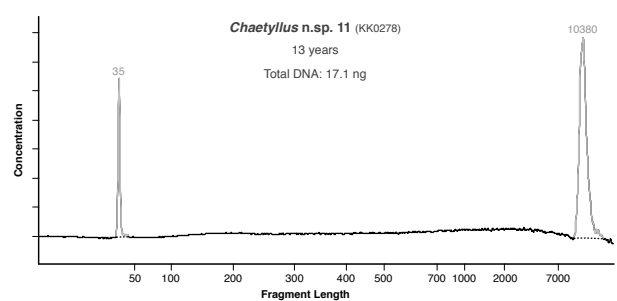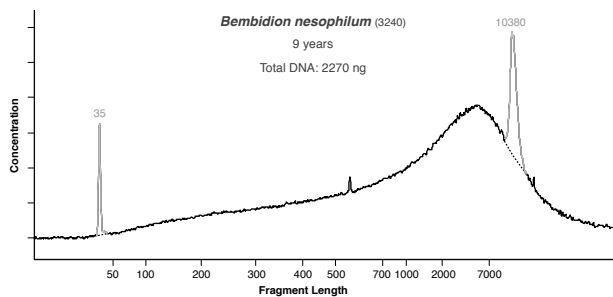

Supplement: S2 Fig — Pale spikes at 35 and 10380 bases represent standards included in each analysis. (PDF) [file pone.0143929.s002.pdf]

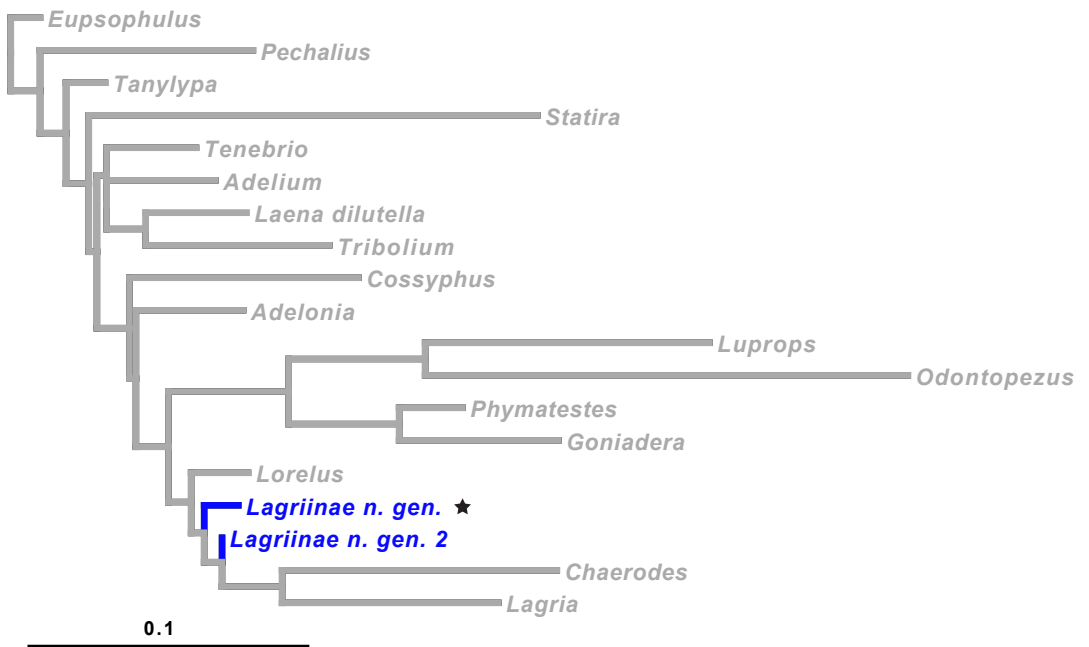

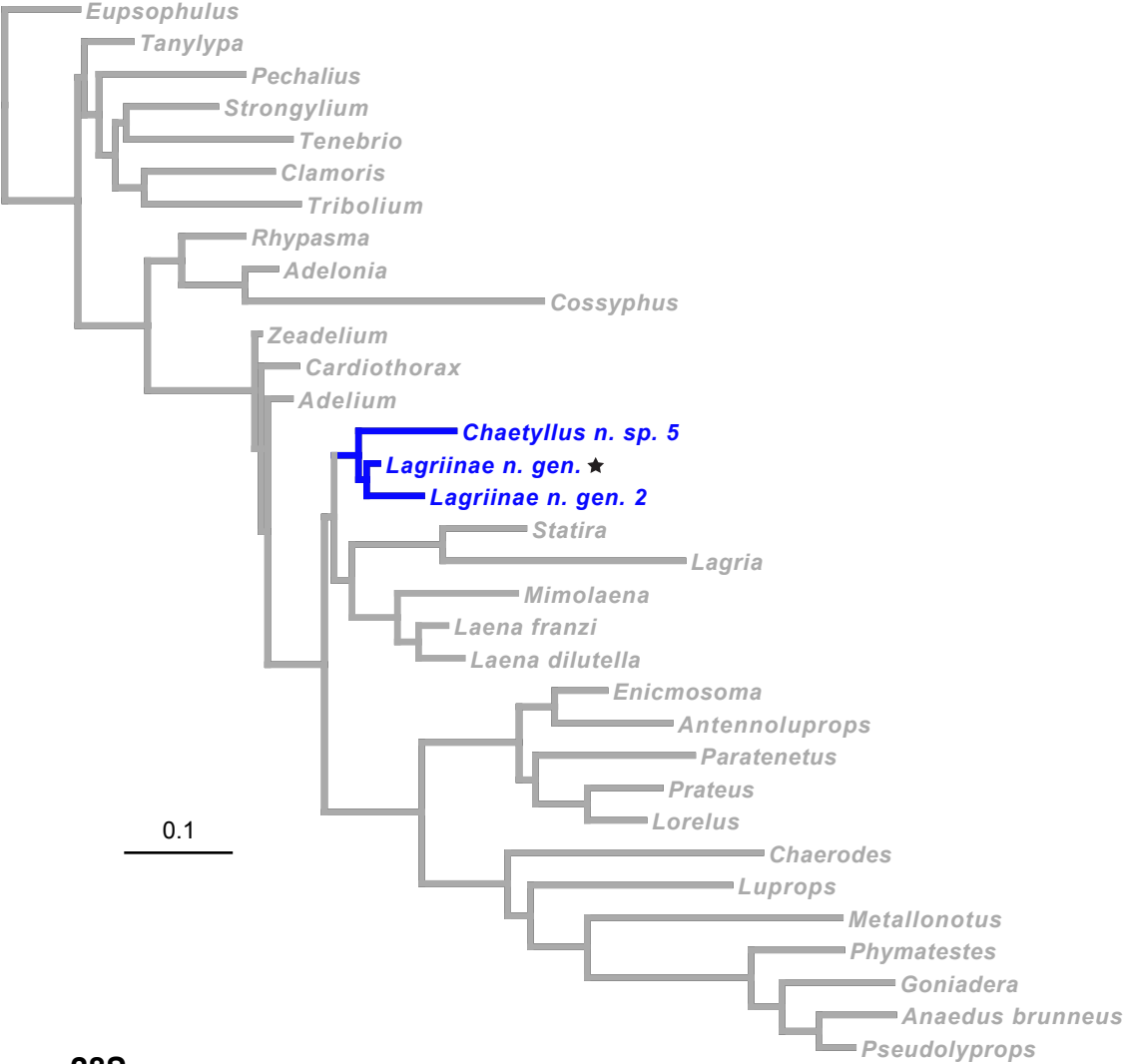

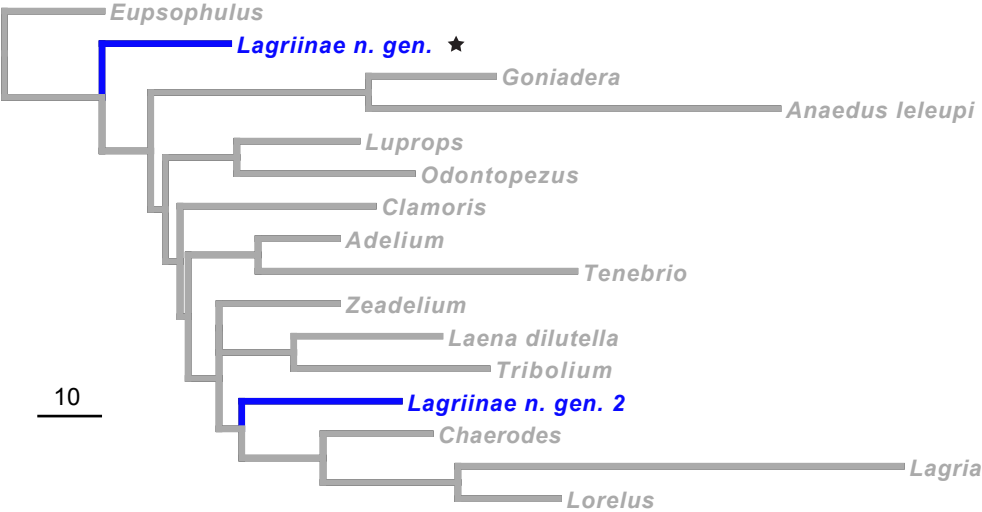

COI

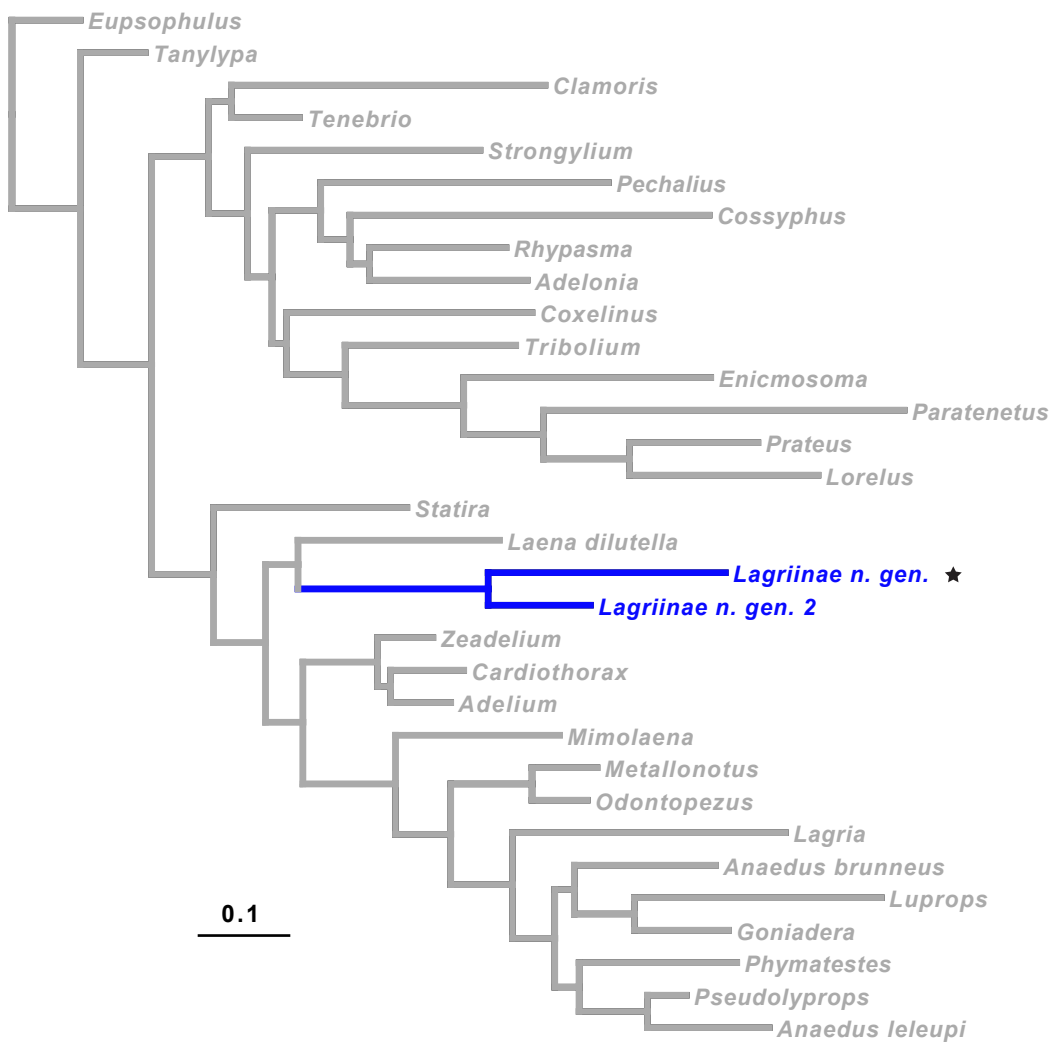

ArgK

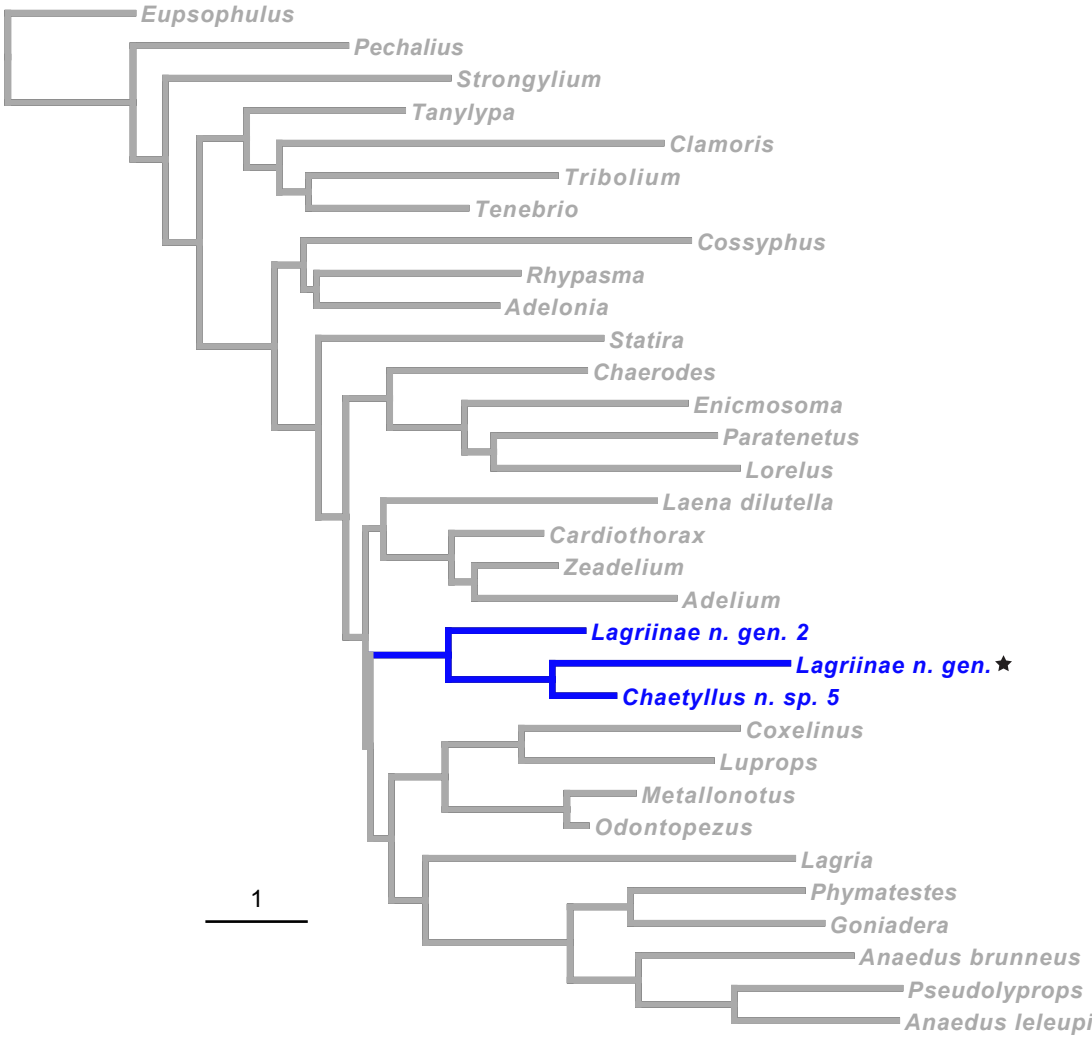

CAD

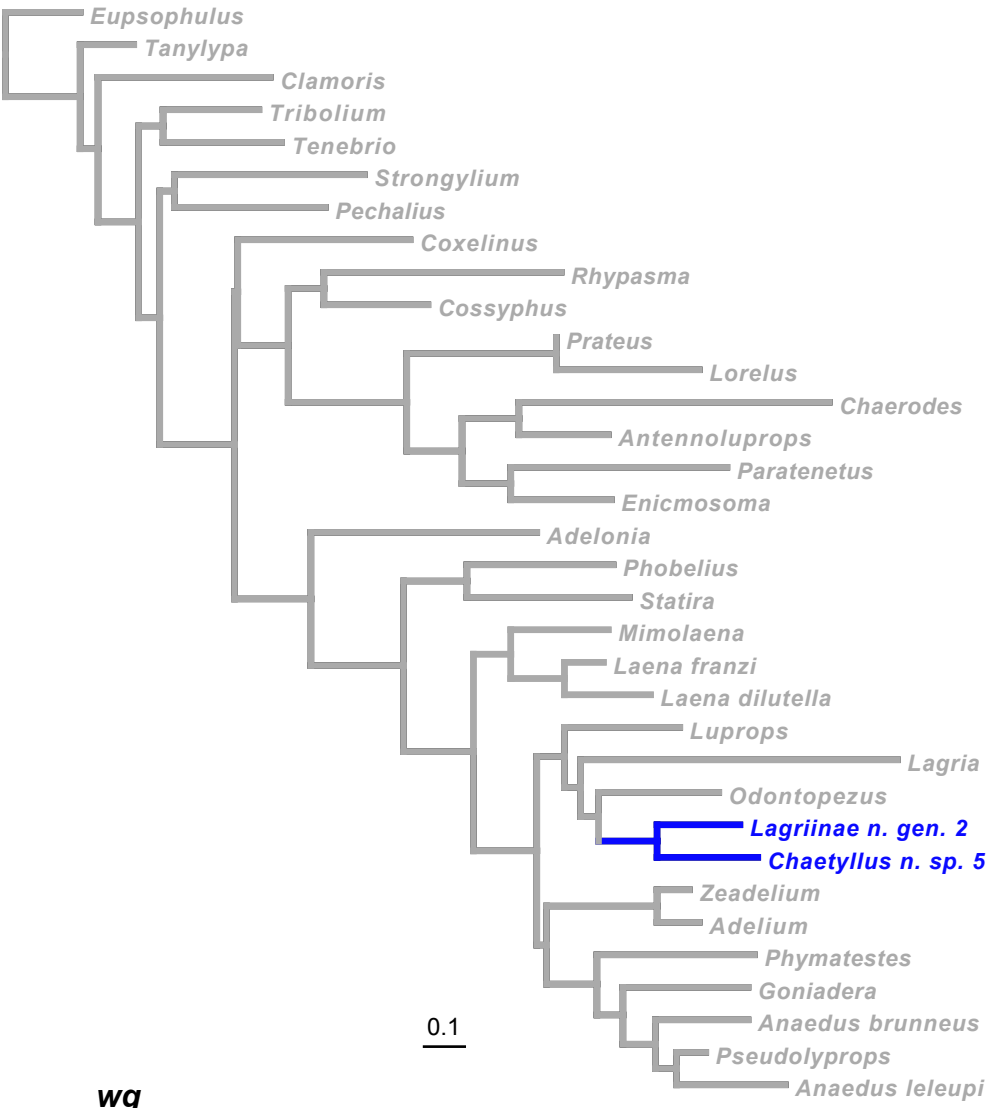

wg

Supplement: S3 Fig — The museum specimen is marked with a star symbol. The branches and taxon names of Lagriinae n. gen and its predicted closest relatives (based on morphological characters) are colored in blue. No sequences for wg were recovered from Lagriinae n. gen. (PDF) [file pone.0143929.s003.pdf]

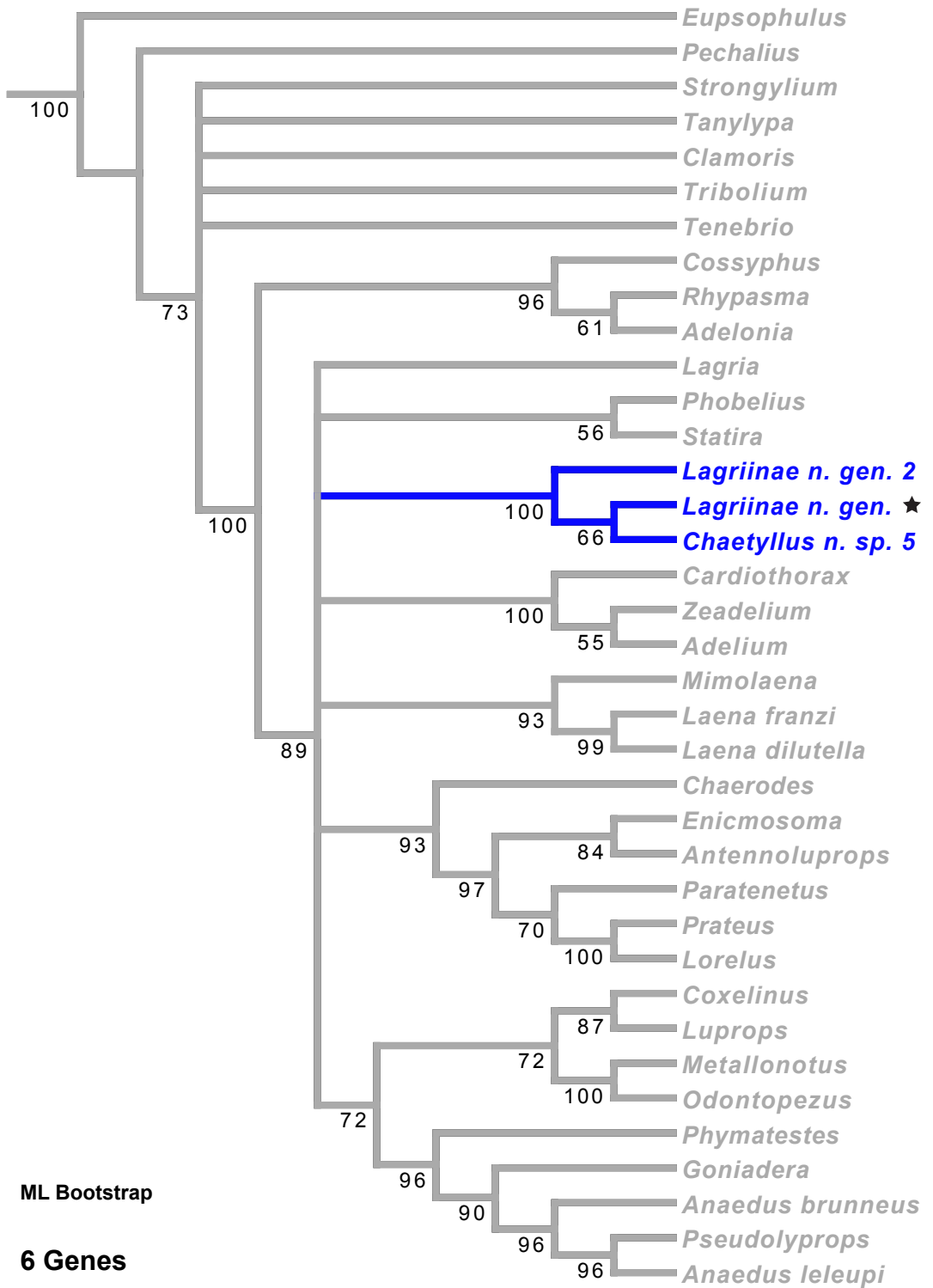

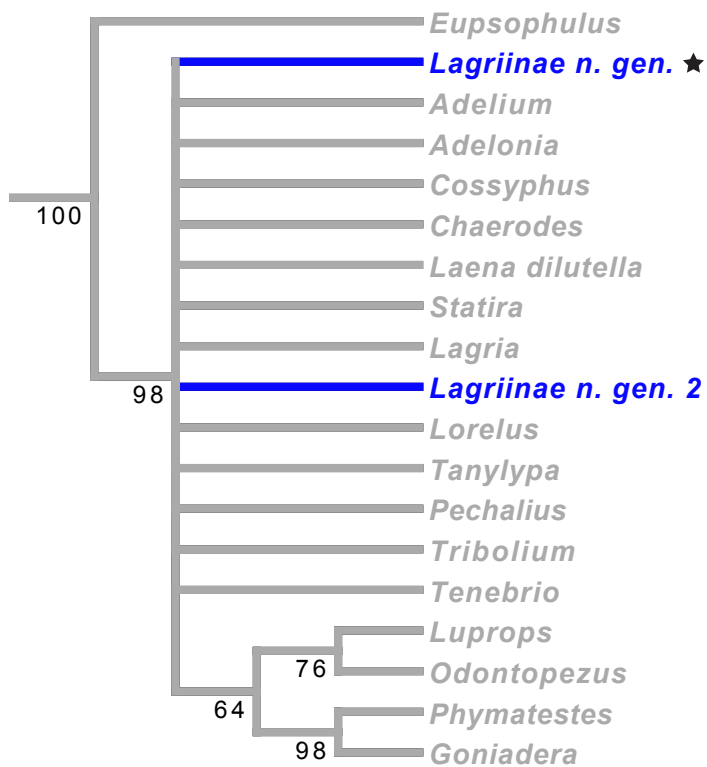

ML Bootstrap

18S

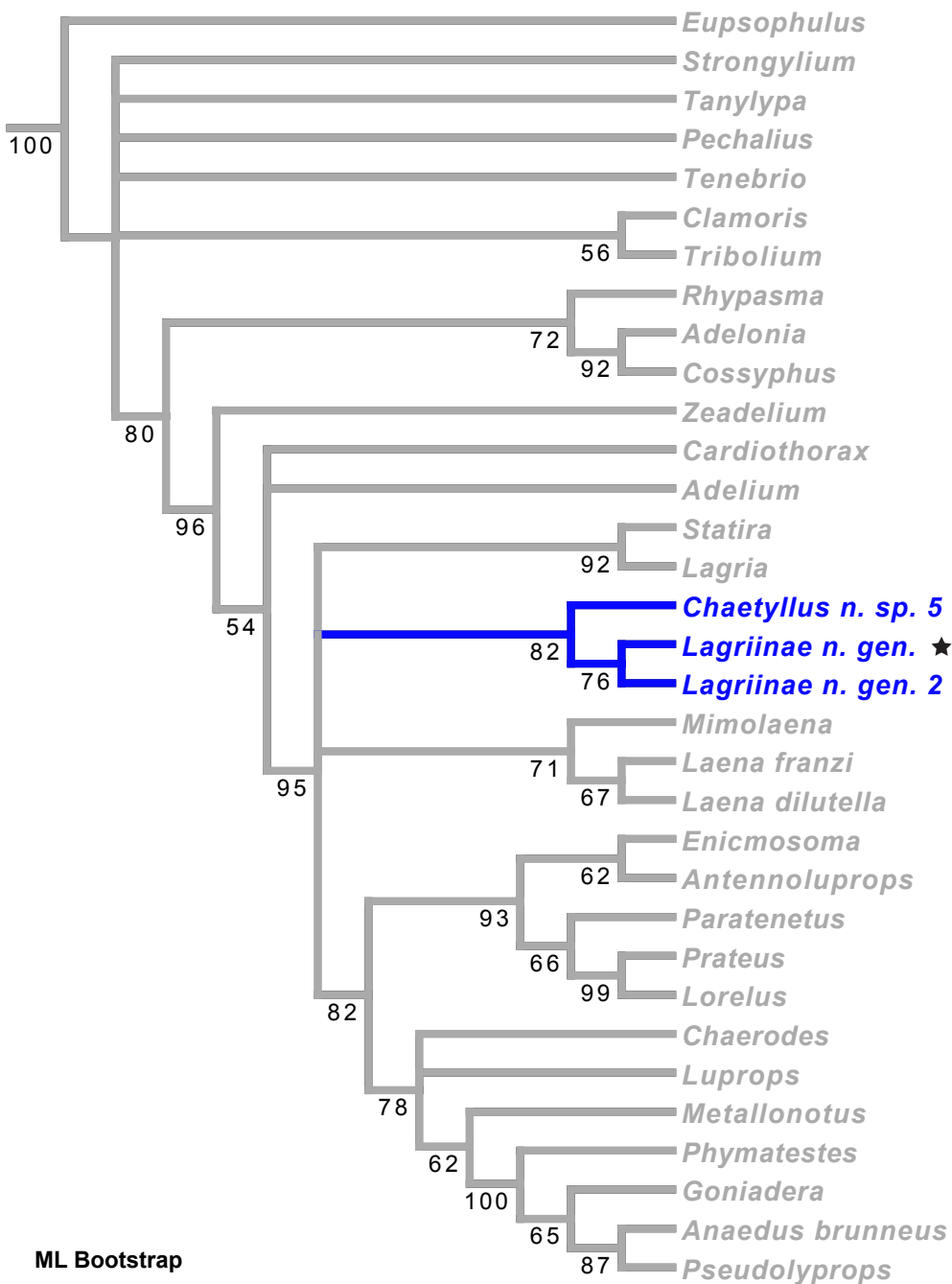

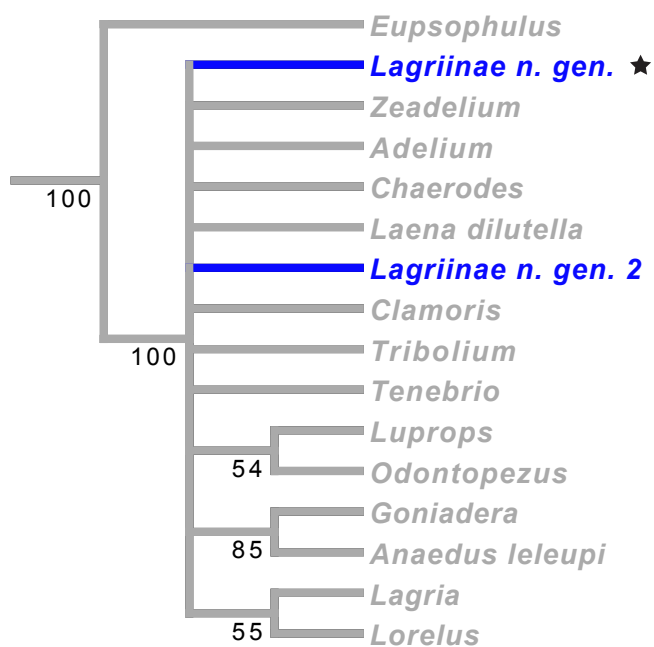

ML Bootstrap

COI

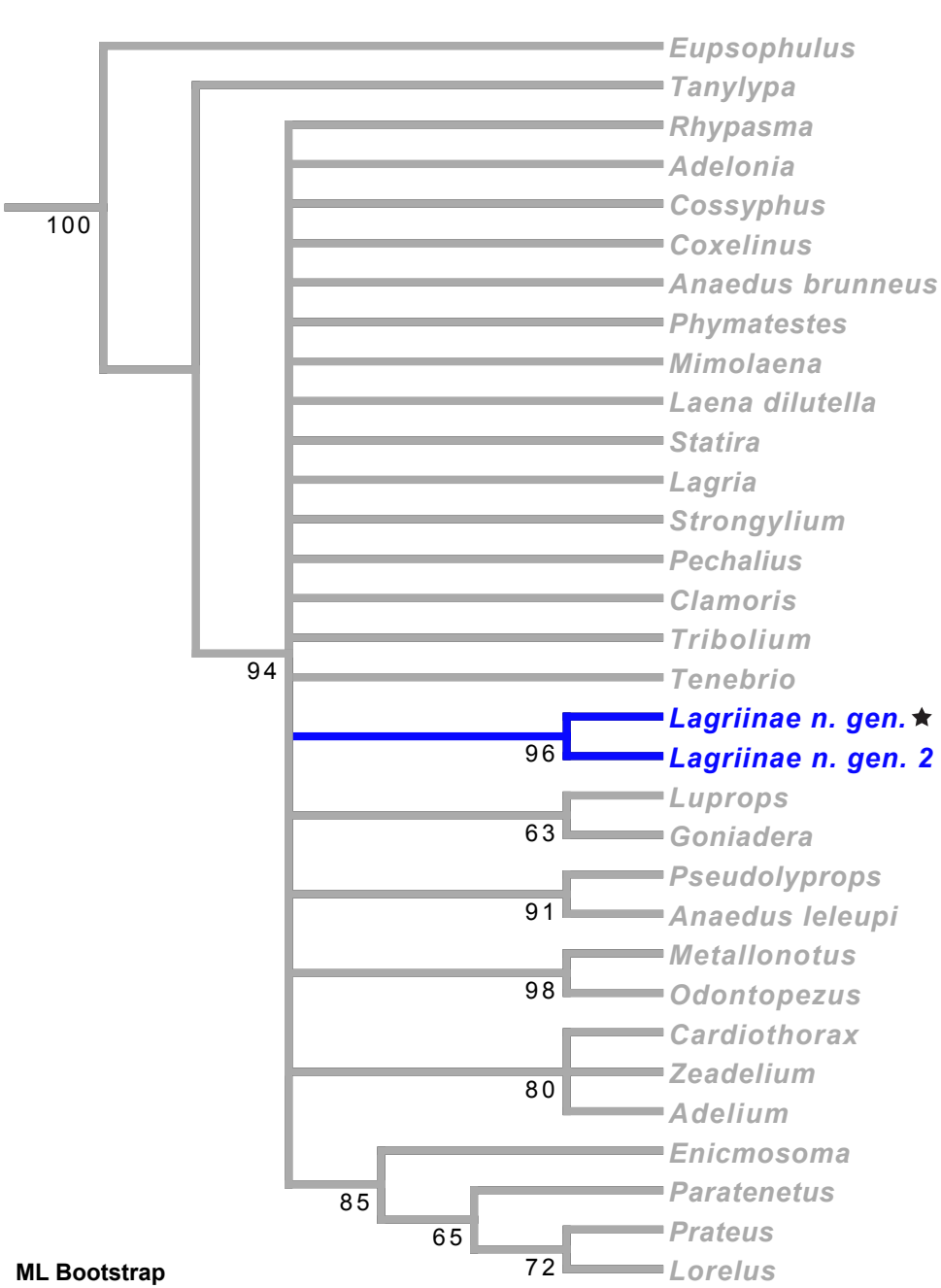

ML Bootstrap

ArgK

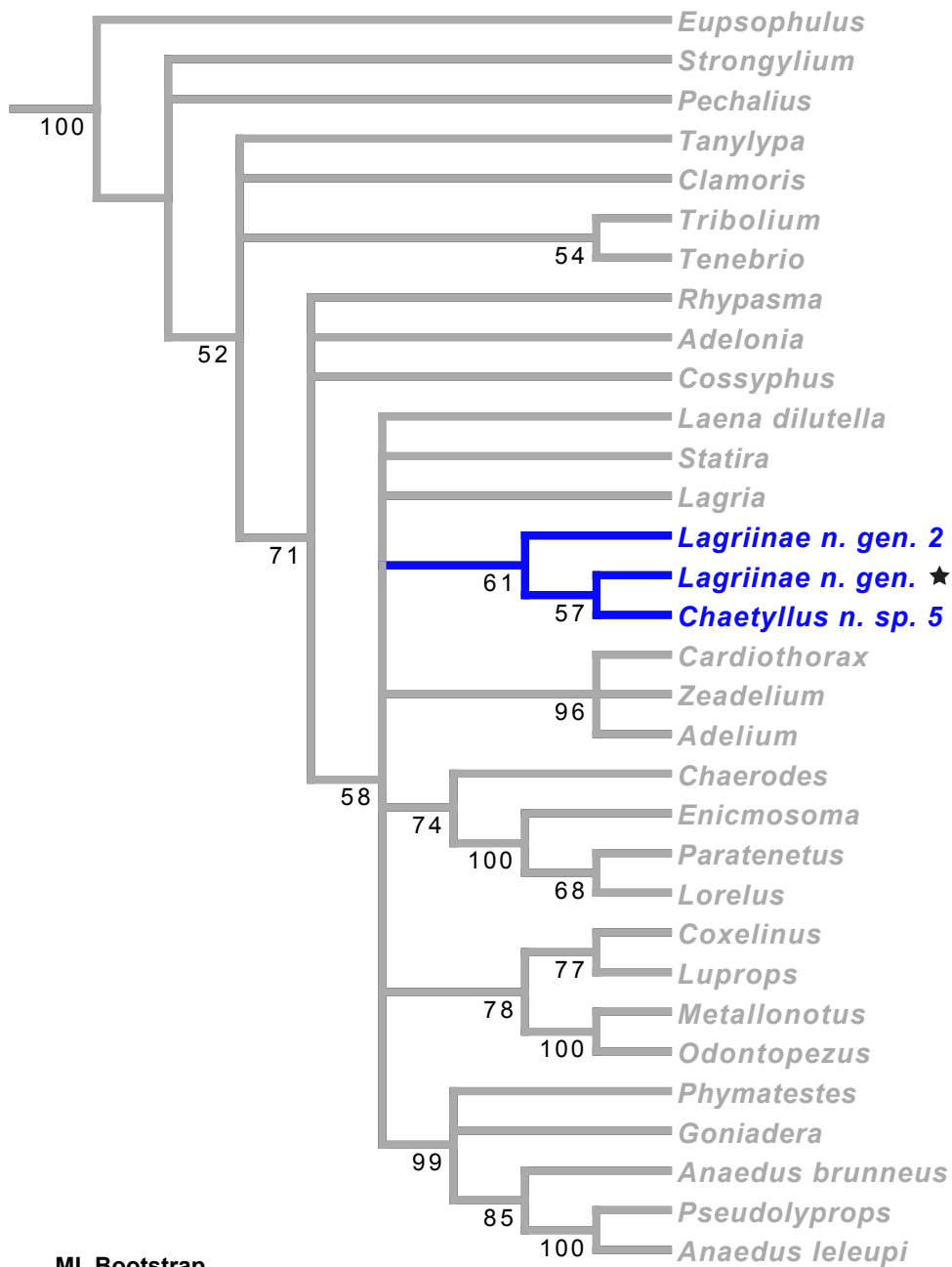

ML Bootstrap

CAD

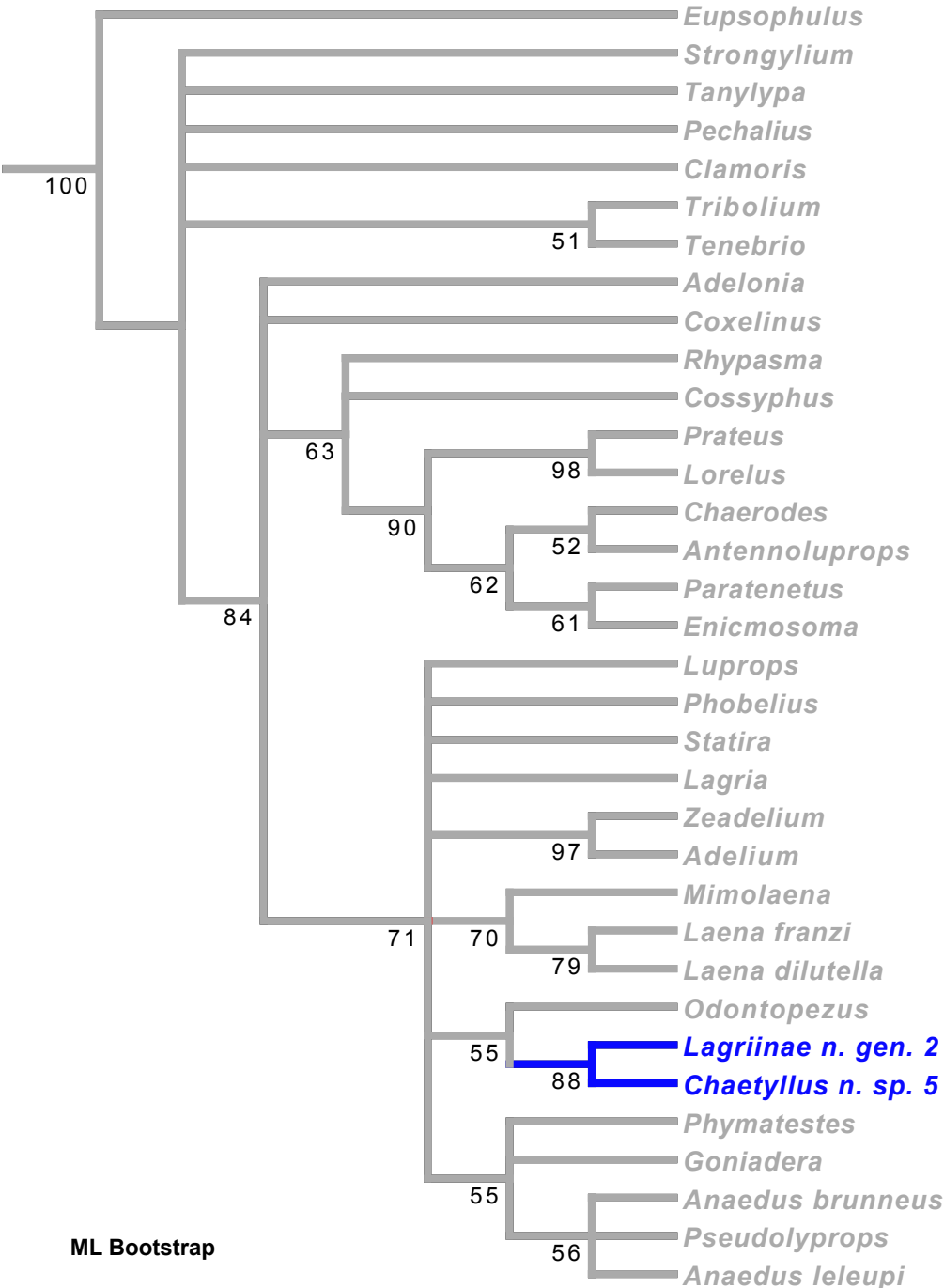

ML Bootstrap

wg

Supplement: S4 Fig — Bootstrap support values given below the branch at nodes with at bootstrap values of at least 50. The museum specimen is marked with a star symbol. The branches and taxon names of Lagriinae n. gen and its predicted closest relatives (based on morphological characters) are colored in blue. No sequences for wg were recovered from Lagriinae n. gen (PDF) [file pone.0143929.s004.pdf]
