# Supplementary material for: Successful Recovery of Nuclear Protein-Coding Genes from Small Insects in Museums Using Illumina Sequencing
Source: PLoS One. 2015 Dec 30;10(12):e0143929. doi: 10.1371/journal.pone.0143929 (PMC4696846; doi:10.1371/journal.pone.0143929)
Supplement: S5 Fig — Each tree includes all contigs returned from our BLAST searches for target genes within HTS museum specimen assemblies, prior to our selecting the chosen contig for that specimen. The contig that was chosen through our criteria outlined in the text is marked with a star symbol. (PDF) [file pone.0143929.s005.pdf]

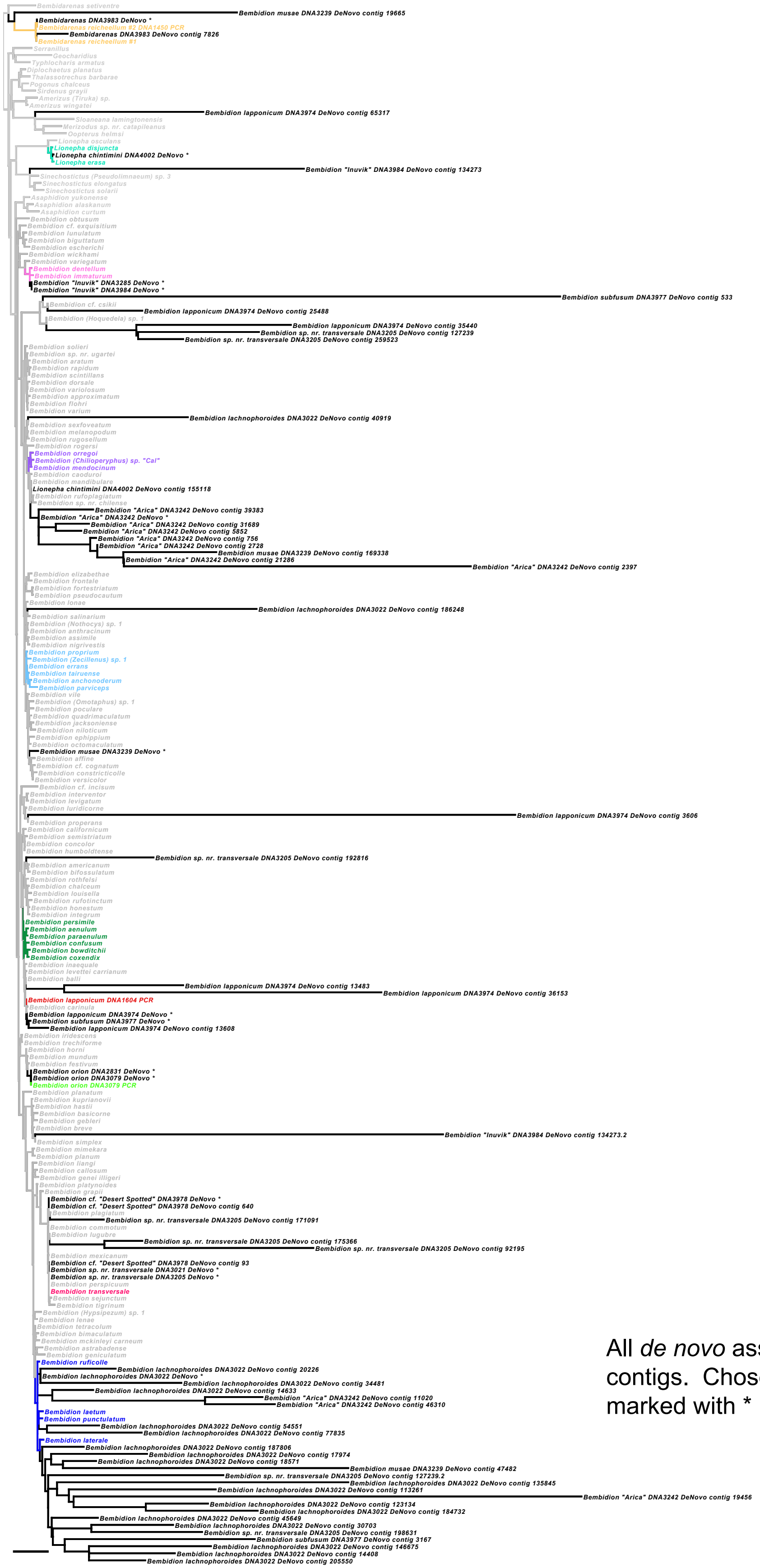

All *de novo* assembly contigs. Chosen *de novo* marked with *

18S

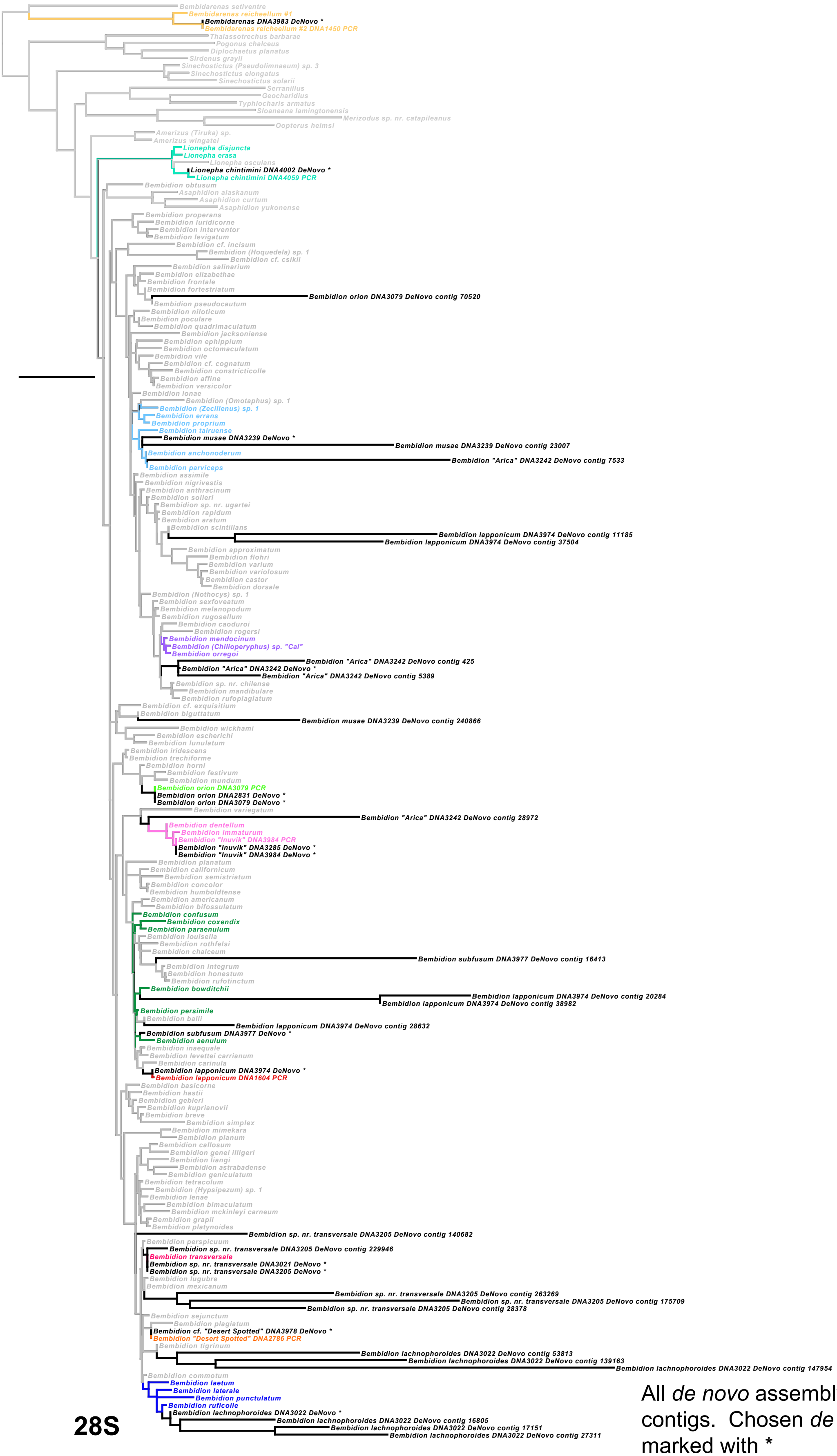

All *de novo* assembly contigs. Chosen *de novo* marked with *

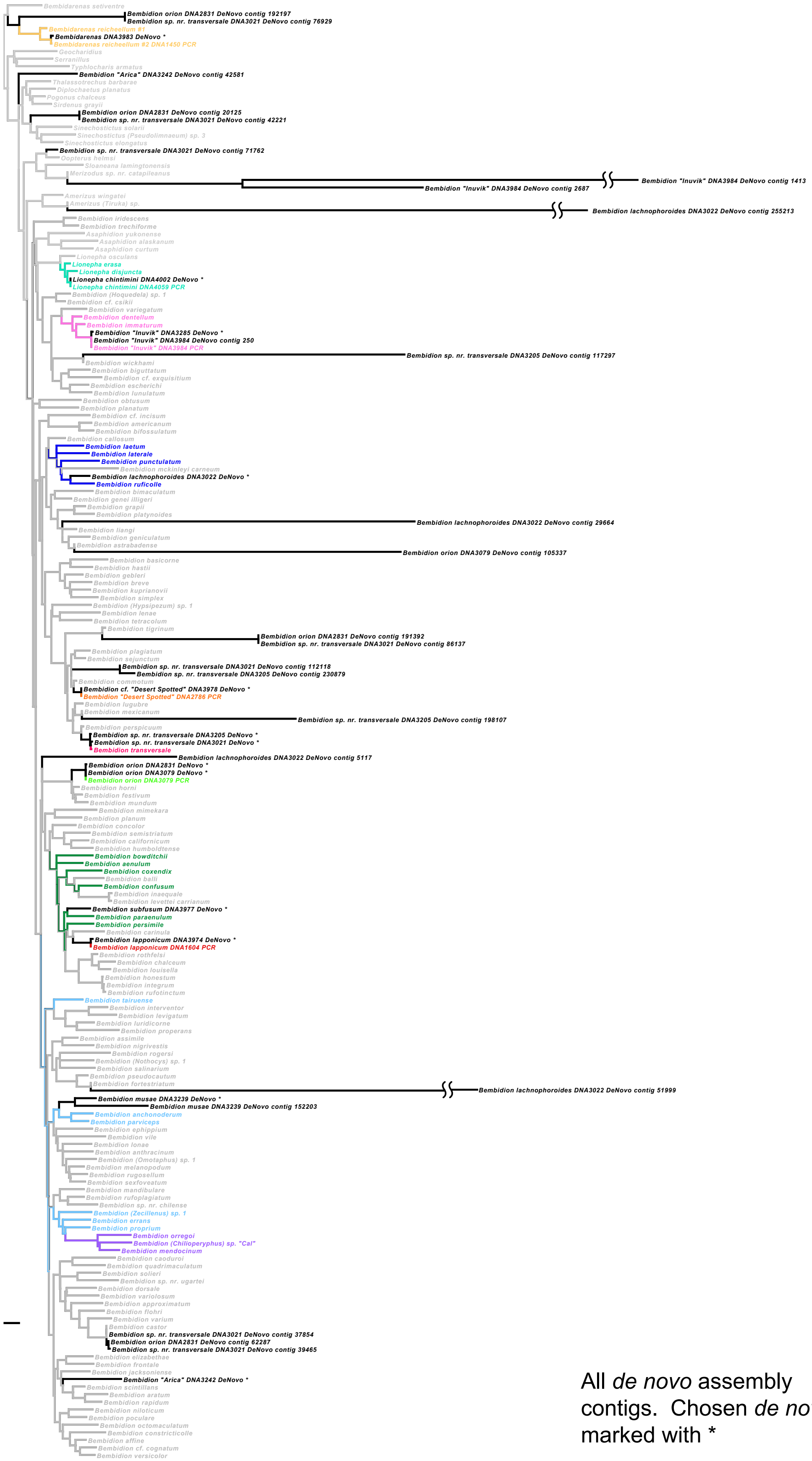

COI

All *de novo* assembly contigs. Chosen *de novo* marked with *

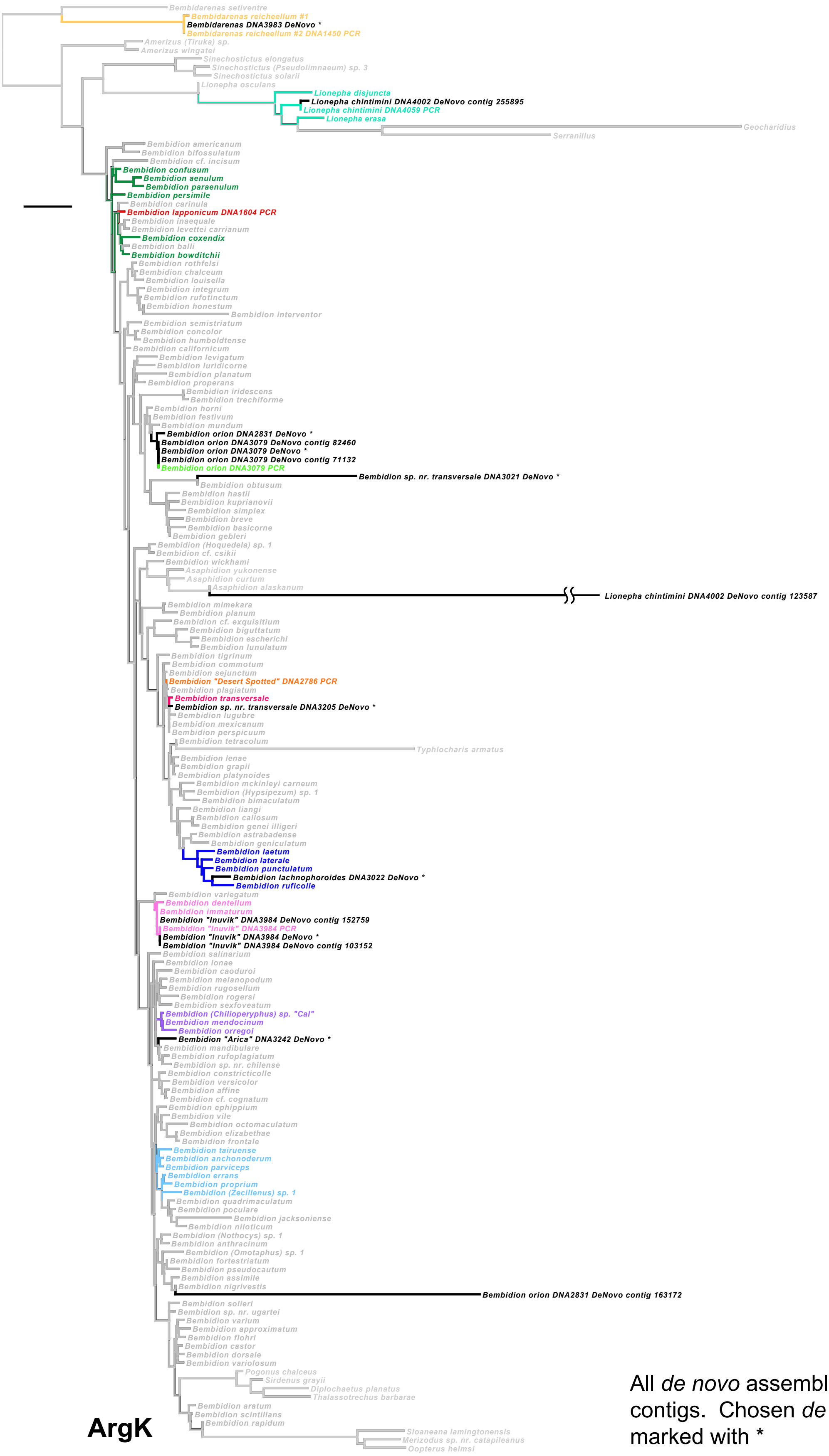

All *de novo* assembly contigs. Chosen *de novo* marked with *

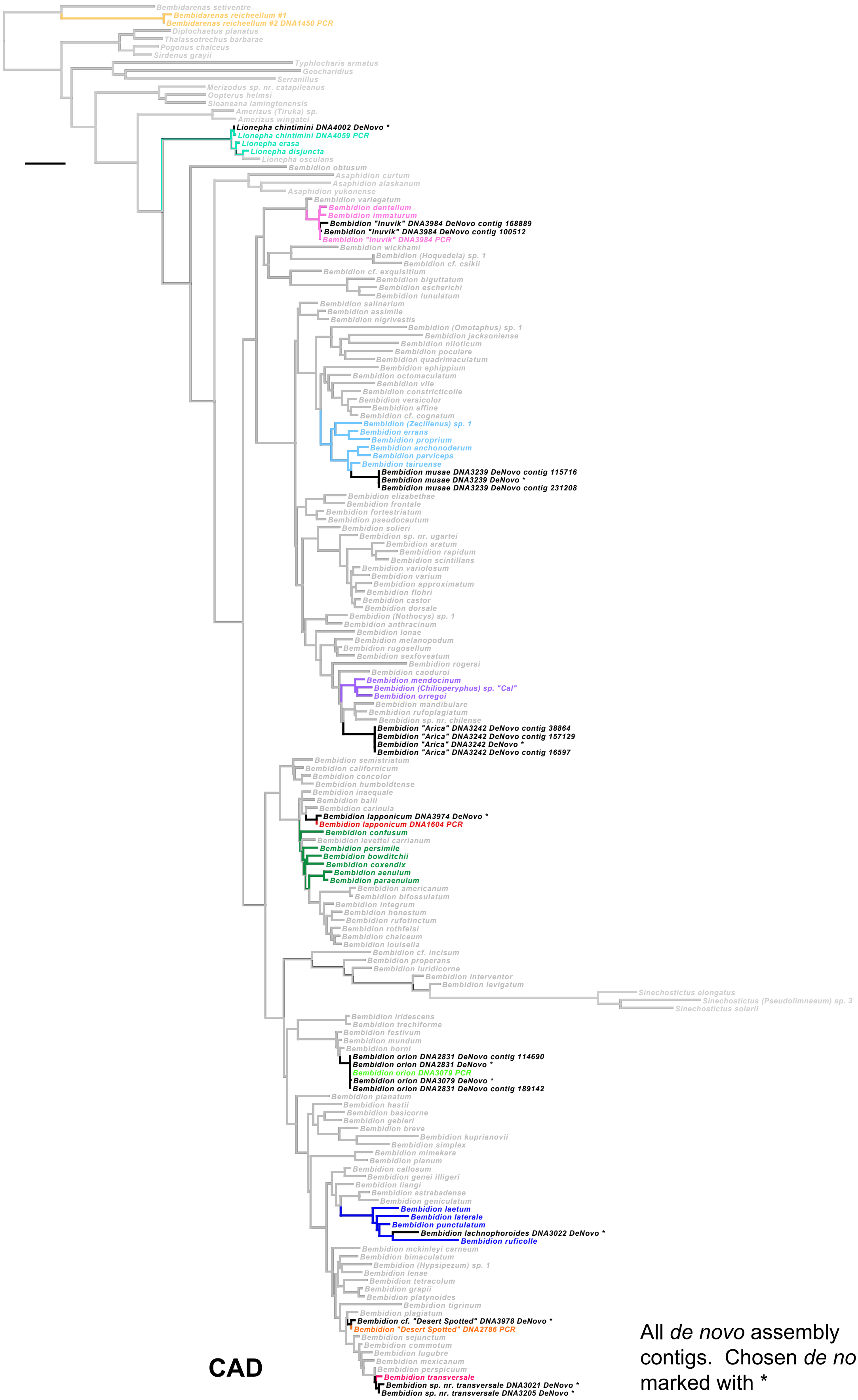

All *de novo* assembly contigs. Chosen *de novo* marked with *

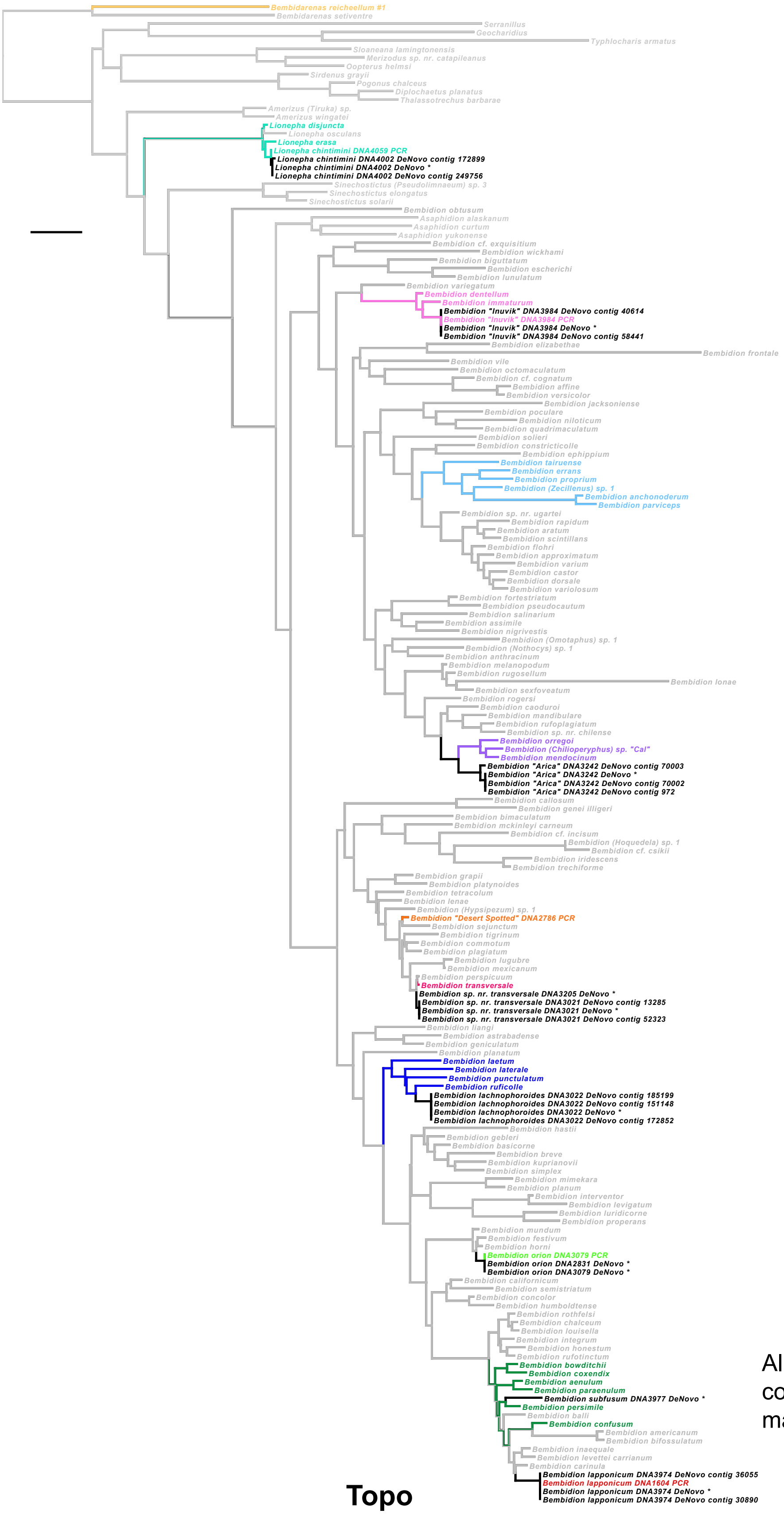

All *de novo* assembly contigs. Chosen *de novo* marked with *

Topo

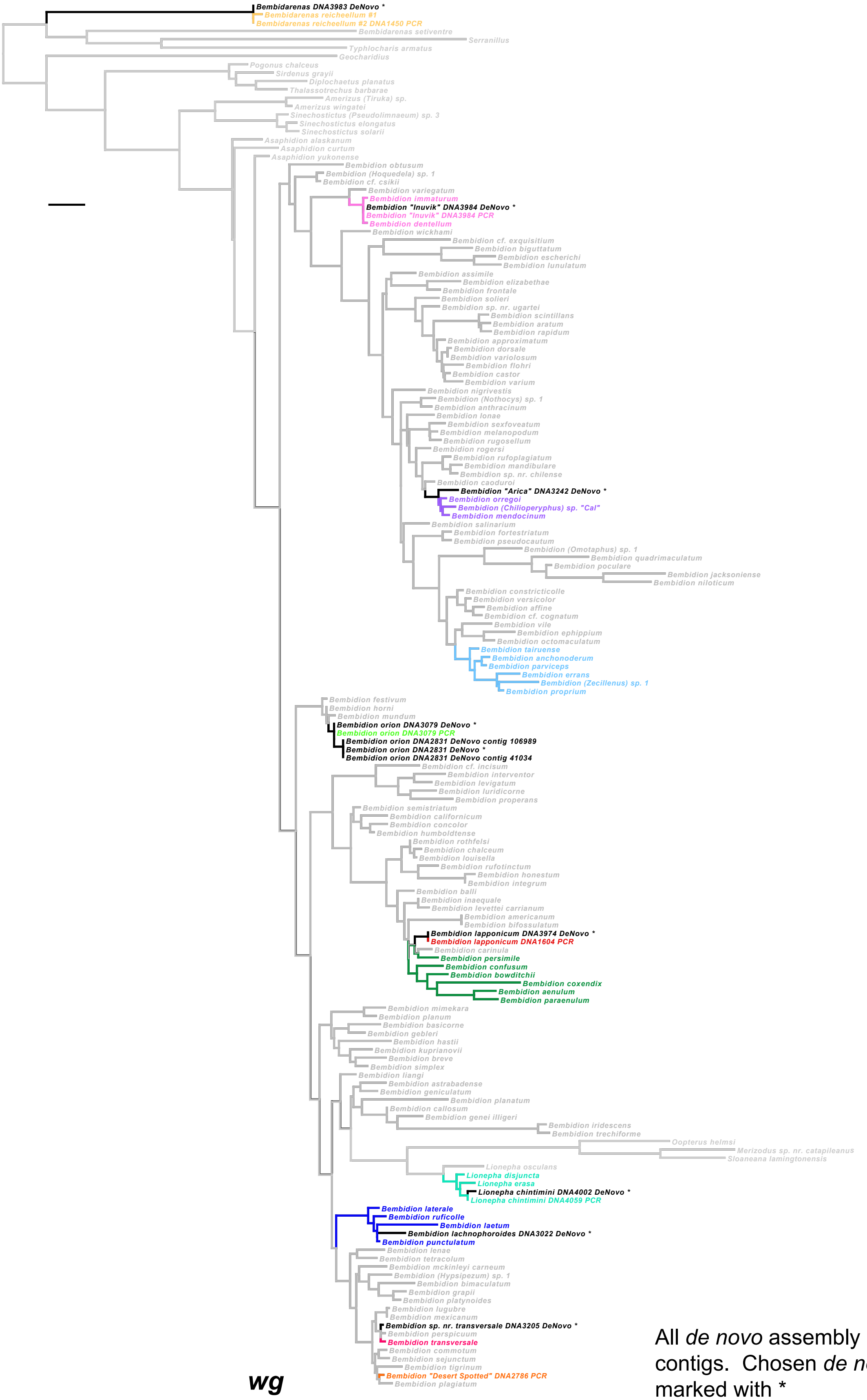

All *de novo* assembly contigs. Chosen *de novo* marked with *
