# Supplementary material for: Successful Recovery of Nuclear Protein-Coding Genes from Small Insects in Museums Using Illumina Sequencing
Source: PLoS One. 2015 Dec 30;10(12):e0143929. doi: 10.1371/journal.pone.0143929 (PMC4696846; doi:10.1371/journal.pone.0143929)
Supplement: S6 Fig — The placement of the DeNovo, NearRef, and FarRef sequences is shown relative to their prediction groups. Each prediction group is indicated with a unique color for branches and taxon names of all specimens in the prediction group. (PDF) [file pone.0143929.s006.pdf]

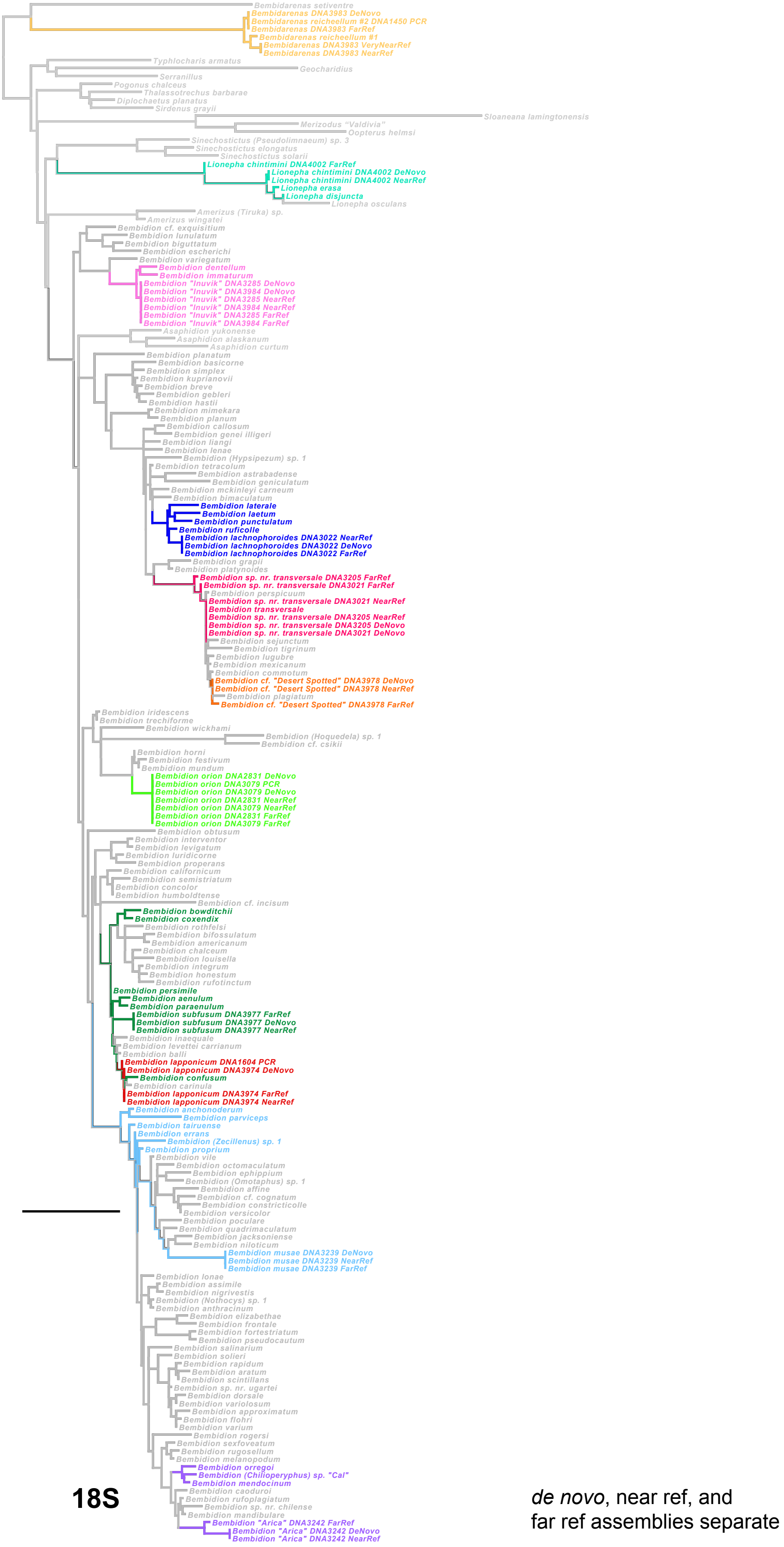
Bembidarenas setiventre
Bembidarenas DNA3983 DeNovo
Bembidarenas reicheellum #2 DNA1450 PCR
Bembidarenas DNA3983 FarRef
Bembidarenas reicheellum #1
Bembidarenas DNA3983 VeryNearRef
Bembidarenas DNA3983 NearRef
Typhlocharis armatus
Geocharidius
Serranillus
Pogonus chalceus
Thalassotrechus barbarae
Diplochaetus planatus
Sirdenus grayii
Sloaneana lamingtonensis
Merizodus "Valdivia"
Oopterus helmsi
Sinechostictus (Pseudolimnaeum) sp. 3
Sinechostictus elongatus
Sinechostictus solarii
Lionepha chintimini DNA4002 FarRef
Lionepha chintimini DNA4002 DeNovo
Lionepha chintimini DNA4002 NearRef
Lionepha erasa
Lionepha disjuncta
Lionepha osculans
Amerizus (Tiruka) sp.
Amerizus wingatei
Bembidion cf. exquisitium
Bembidion lunulatum
Bembidion biguttatum
Bembidion escherichi
Bembidion variegatum
Bembidion dentellum
Bembidion immaturum
Bembidion "Inuvik" DNA3285 DeNovo
Bembidion "Inuvik" DNA3984 DeNovo
Bembidion "Inuvik" DNA3285 NearRef
Bembidion "Inuvik" DNA3984 NearRef
Bembidion "Inuvik" DNA3285 FarRef
Bembidion "Inuvik" DNA3984 FarRef
Asaphidion yukonense
Asaphidion alaskanum
Asaphidion curtum
Bembidion planatum
Bembidion basicorne
Bembidion simplex
Bembidion kuprianovii
Bembidion breve
Bembidion gebleri
Bembidion hastii
Bembidion mimekara
Bembidion planum
Bembidion callosum
Bembidion genei illigeri
Bembidion liangi
Bembidion lenae
Bembidion (Hypsipezum) sp. 1
Bembidion tetracolum
Bembidion astrabadense
Bembidion geniculatum
Bembidion mckinleyi carneum
Bembidion bimaculatum
Bembidion laterale
Bembidion laetum
Bembidion punctulatum
Bembidion ruficolle
Bembidion lachnophoroides DNA3022 NearRef
Bembidion lachnophoroides DNA3022 DeNovo
Bembidion lachnophoroides DNA3022 FarRef
Bembidion grapii
Bembidion platynoides
Bembidion sp. nr. transversale DNA3205 FarRef
Bembidion sp. nr. transversale DNA3021 FarRef
Bembidion perspicuum
Bembidion sp. nr. transversale DNA3021 NearRef
Bembidion transversale
Bembidion sp. nr. transversale DNA3205 NearRef
Bembidion sp. nr. transversale DNA3205 DeNovo
Bembidion sp. nr. transversale DNA3021 DeNovo
Bembidion sejunctum
Bembidion tigrinum
Bembidion lugubre
Bembidion mexicanum
Bembidion commotum
Bembidion cf. "Desert Spotted" DNA3978 DeNovo
Bembidion cf. "Desert Spotted" DNA3978 NearRef
Bembidion plagiatum
Bembidion cf. "Desert Spotted" DNA3978 FarRef
Bembidion iridescens
Bembidion trechiforme
Bembidion wickhami
Bembidion (Hoquedela) sp. 1
Bembidion cf. csikii
Bembidion horni
Bembidion festivum
Bembidion mundum
Bembidion orion DNA2831 DeNovo
Bembidion orion DNA3079 PCR
Bembidion orion DNA3079 DeNovo
Bembidion orion DNA2831 NearRef
Bembidion orion DNA3079 NearRef
Bembidion orion DNA2831 FarRef
Bembidion orion DNA3079 FarRef
Bembidion obtusum
Bembidion interventor
Bembidion levigatum
Bembidion luridicorne
Bembidion properans
Bembidion californicum
Bembidion semistriatum
Bembidion concolor
Bembidion humboldtense
Bembidion cf. incisum
Bembidion bowditchii
Bembidion coxendix
Bembidion rothfelsi
Bembidion bifossulatum
Bembidion americanum
Bembidion chalceum
Bembidion louisella
Bembidion integrum
Bembidion honestum
Bembidion rufotinctum
Bembidion persimile
Bembidion aenulum
Bembidion paraenulum
Bembidion subfusum DNA3977 FarRef
Bembidion subfusum DNA3977 DeNovo
Bembidion subfusum DNA3977 NearRef
Bembidion inaequale
Bembidion levettei carrianum
Bembidion balli
Bembidion lapponicum DNA1604 PCR
Bembidion lapponicum DNA3974 DeNovo
Bembidion confusum
Bembidion carinula
Bembidion lapponicum DNA3974 FarRef
Bembidion lapponicum DNA3974 NearRef
Bembidion anchonoderum
Bembidion parviceps
Bembidion tairuense
Bembidion errans
Bembidion (Zecillenus) sp. 1
Bembidion proprium
Bembidion vile
Bembidion octomaculatum
Bembidion ephippium
Bembidion (Omotaphus) sp. 1
Bembidion affine
Bembidion cf. cognatum
Bembidion constricticolle
Bembidion versicolor
Bembidion poculare
Bembidion quadrimaculatum
Bembidion jacksoniense
Bembidion niloticum
Bembidion musae DNA3239 DeNovo
Bembidion musae DNA3239 NearRef
Bembidion musae DNA3239 FarRef
Bembidion lonae
Bembidion assimile
Bembidion nigrivestis
Bembidion (Nothocys) sp. 1
Bembidion anthracinum
Bembidion elizabethae
Bembidion frontale
Bembidion fortestriatum
Bembidion pseudocautum
Bembidion salinarium
Bembidion solieri
Bembidion rapidum
Bembidion aratum
Bembidion scintillans
Bembidion sp. nr. ugartei
Bembidion dorsale
Bembidion variolosum
Bembidion approximatum
Bembidion flohri
Bembidion varium
Bembidion rogersi
Bembidion sexfoveatum
Bembidion rugosellum
Bembidion melanopodum
Bembidion orregoi
Bembidion (Chilioperyphus) sp. "Cal"
Bembidion mendocinum
Bembidion caoduroi
Bembidion rufoplagiatum
Bembidion sp. nr. chilense
Bembidion mandibulare
Bembidion "Arica" DNA3242 FarRef
Bembidion "Arica" DNA3242 DeNovo
Bembidion "Arica" DNA3242 NearRef
18S
de novo, near ref, and
far ref assemblies separate

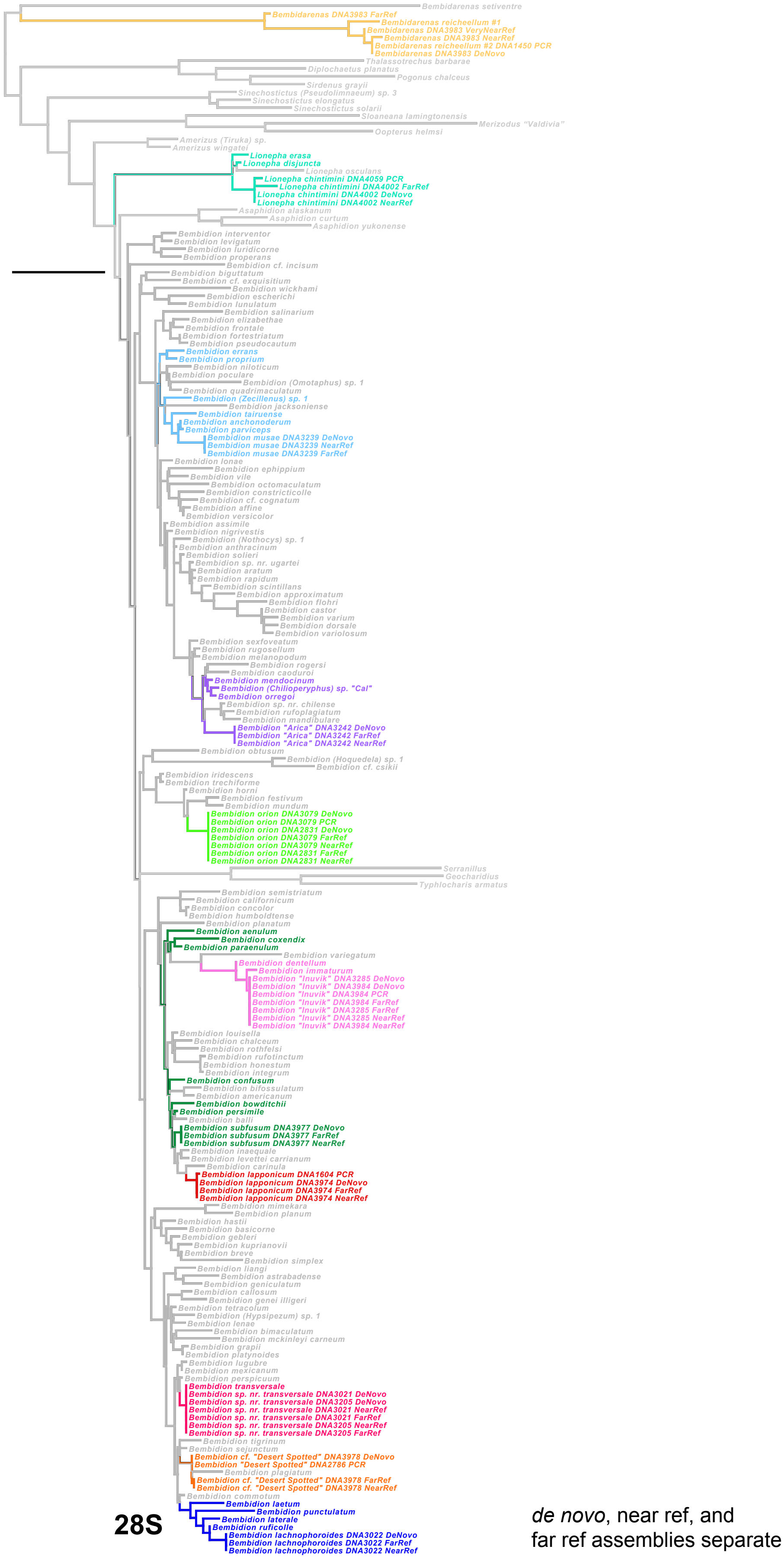

Bembidarenas setiventre
Bembidarenas DNA3983 FarRef
Bembidarenas reicheellum #1
Bembidarenas DNA3983 VeryNearRef
Bembidarenas DNA3983 NearRef
Bembidarenas reicheellum #2 DNA1450 PCR
Bembidarenas DNA3983 DeNovo
Thalassotrechus barbarae
Diplochaetus planatus
Pogonus chalceus
Sirdenus grayii
Sinechostictus (Pseudolimnaeum) sp. 3
Sinechostictus elongatus
Sinechostictus solarii
Sloaneana lamingtonensis
Merizodus "Valdivia"
Oopterus helmsi
Amerizus (Tiruka) sp.
Amerizus wingatei
Lionepha erasa
Lionepha disjuncta
Lionepha osculans
Lionepha chintimini DNA4059 PCR
Lionepha chintimini DNA4002 FarRef
Lionepha chintimini DNA4002 DeNovo
Lionepha chintimini DNA4002 NearRef
Asaphidion alaskanum
Asaphidion curtum
Asaphidion yukonense
Bembidion interventor
Bembidion levigatum
Bembidion luridicorne
Bembidion properans
Bembidion cf. incisum
Bembidion biguttatum
Bembidion cf. exquisitium
Bembidion wickhami
Bembidion escherichi
Bembidion lunulatum
Bembidion salinarium
Bembidion elizabethae
Bembidion frontale
Bembidion fortestriatum
Bembidion pseudocautum
Bembidion errans
Bembidion proprium
Bembidion niloticum
Bembidion poculare
Bembidion (Omotaphus) sp. 1
Bembidion quadrimaculatum
Bembidion (Zecillenus) sp. 1
Bembidion jacksoniense
Bembidion tairuense
Bembidion anchonoderum
Bembidion parviceps
Bembidion musae DNA3239 DeNovo
Bembidion musae DNA3239 NearRef
Bembidion musae DNA3239 FarRef
Bembidion lonae
Bembidion ephippium
Bembidion vile
Bembidion octomaculatum
Bembidion constricticolle
Bembidion cf. cognatum
Bembidion affine
Bembidion versicolor
Bembidion assimile
Bembidion nigrivestis
Bembidion (Nothocys) sp. 1
Bembidion anthracinum
Bembidion solieri
Bembidion sp. nr. ugartei
Bembidion aratum
Bembidion rapidum
Bembidion scintillans
Bembidion approximatum
Bembidion flohri
Bembidion castor
Bembidion varium
Bembidion dorsale
Bembidion variolosum
Bembidion sexfoveatum
Bembidion rugosellum
Bembidion melanopodum
Bembidion rogersi
Bembidion caoduroi
Bembidion mendocinum
Bembidion (Chilioperyphus) sp. "Cal"
Bembidion orregoi
Bembidion sp. nr. chilense
Bembidion rufoplagiatum
Bembidion mandibulare
Bembidion "Arica" DNA3242 DeNovo
Bembidion "Arica" DNA3242 FarRef
Bembidion "Arica" DNA3242 NearRef
Bembidion obtusum
Bembidion (Hoquedela) sp. 1
Bembidion cf. csikii
Bembidion iridescens
Bembidion trechiforme
Bembidion horni
Bembidion festivum
Bembidion mundum
Bembidion orion DNA3079 DeNovo
Bembidion orion DNA3079 PCR
Bembidion orion DNA2831 DeNovo
Bembidion orion DNA3079 FarRef
Bembidion orion DNA3079 NearRef
Bembidion orion DNA2831 FarRef
Bembidion orion DNA2831 NearRef
Serranillus
Geocharidius
Typhlocharis armatus
Bembidion semistriatum
Bembidion californicum
Bembidion concolor
Bembidion humboldtense
Bembidion planatum
Bembidion aenulum
Bembidion coxendix
Bembidion paraenulum
Bembidion variegatum
Bembidion dentellum
Bembidion immaturum
Bembidion "Inuvik" DNA3285 DeNovo
Bembidion "Inuvik" DNA3984 DeNovo
Bembidion "Inuvik" DNA3984 PCR
Bembidion "Inuvik" DNA3984 FarRef
Bembidion "Inuvik" DNA3285 FarRef
Bembidion "Inuvik" DNA3285 NearRef
Bembidion "Inuvik" DNA3984 NearRef
Bembidion louisella
Bembidion chalceum
Bembidion rothfelsi
Bembidion rufotinctum
Bembidion honestum
Bembidion integrum
Bembidion confusum
Bembidion bifossulatum
Bembidion americanum
Bembidion bowditchii
Bembidion persimile
Bembidion balli
Bembidion subfusum DNA3977 DeNovo
Bembidion subfusum DNA3977 FarRef
Bembidion subfusum DNA3977 NearRef
Bembidion inaequale
Bembidion levettei carrianum
Bembidion carinula
Bembidion lapponicum DNA1604 PCR
Bembidion lapponicum DNA3974 DeNovo
Bembidion lapponicum DNA3974 FarRef
Bembidion lapponicum DNA3974 NearRef
Bembidion mimekara
Bembidion planum
Bembidion hastii
Bembidion basicorne
Bembidion gebleri
Bembidion kuprianovii
Bembidion breve
Bembidion simplex
Bembidion liangi
Bembidion astrabadense
Bembidion geniculatum
Bembidion callosum
Bembidion genei illigeri
Bembidion tetracolum
Bembidion (Hypsipezum) sp. 1
Bembidion lenae
Bembidion bimaculatum
Bembidion mckinleyi carneum
Bembidion grapii
Bembidion platynoides
Bembidion lugubre
Bembidion mexicanum
Bembidion perspicuum
Bembidion transversale
Bembidion sp. nr. transversale DNA3021 DeNovo
Bembidion sp. nr. transversale DNA3205 DeNovo
Bembidion sp. nr. transversale DNA3021 NearRef
Bembidion sp. nr. transversale DNA3021 FarRef
Bembidion sp. nr. transversale DNA3205 NearRef
Bembidion sp. nr. transversale DNA3205 FarRef
Bembidion tigrinum
Bembidion sejunctum
Bembidion cf. "Desert Spotted" DNA3978 DeNovo
Bembidion "Desert Spotted" DNA2786 PCR
Bembidion plagiatum
Bembidion cf. "Desert Spotted" DNA3978 FarRef
Bembidion cf. "Desert Spotted" DNA3978 NearRef
Bembidion commotum
Bembidion laetum
Bembidion punctulatum
Bembidion laterale
Bembidion ruficolle
Bembidion lachnophoroides DNA3022 DeNovo
Bembidion lachnophoroides DNA3022 FarRef
Bembidion lachnophoroides DNA3022 NearRef
28S
de novo, near ref, and far ref assemblies separate

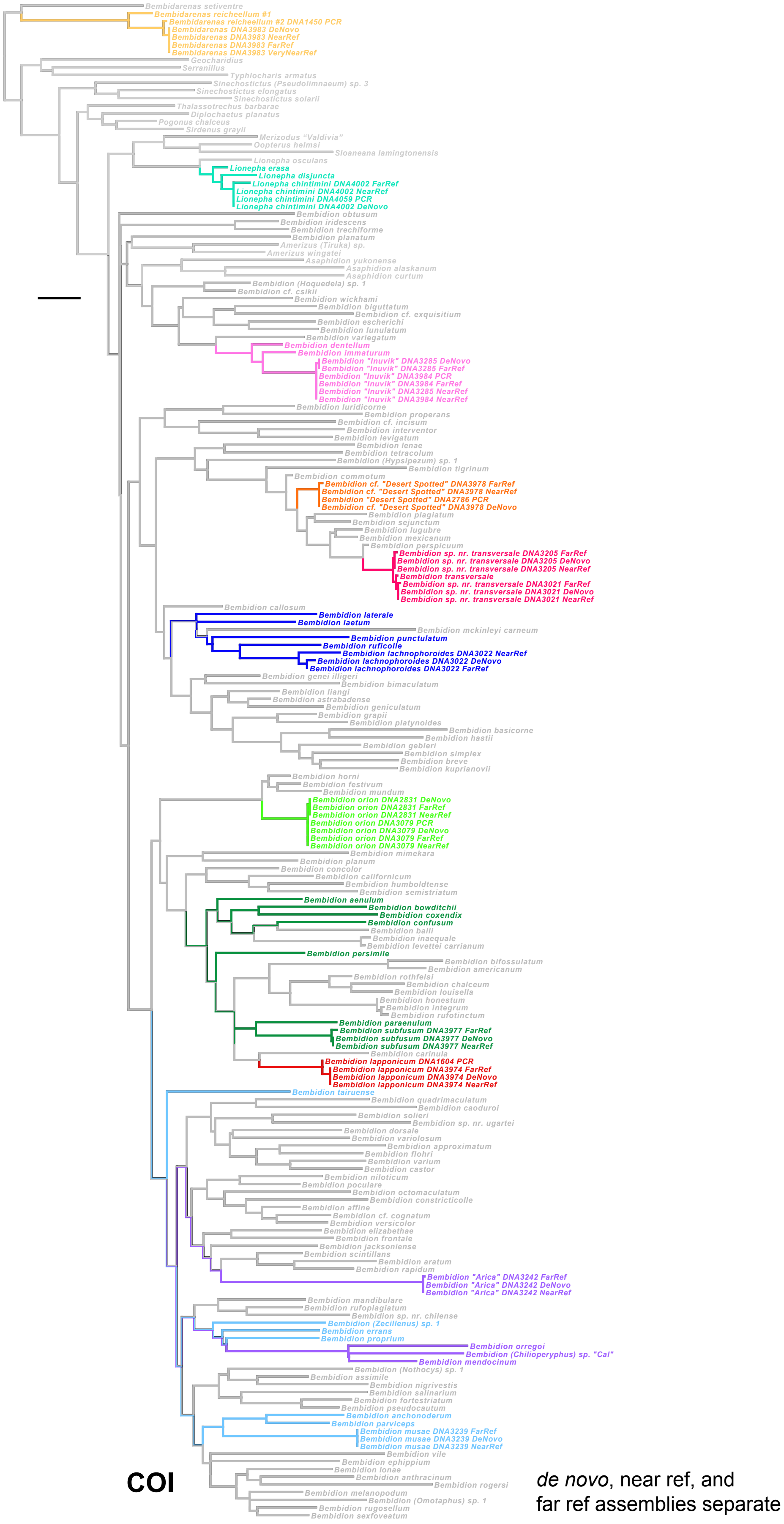
Bembidarenas setiventre
Bembidarenas reicheellum #1
Bembidarenas reicheellum #2 DNA1450 PCR
Bembidarenas DNA3983 DeNovo
Bembidarenas DNA3983 NearRef
Bembidarenas DNA3983 FarRef
Bembidarenas DNA3983 VeryNearRef
Geocharidius
Serranillus
Typhlocharis armatus
Sinechostictus (Pseudolimnaeum) sp. 3
Sinechostictus elongatus
Sinechostictus solarii
Thalassotrechus barbarae
Diplochaetus planatus
Pogonus chalceus
Sirdenus grayii
Merizodus "Valdivia"
Oopterus helmsi
Sloaneana lamingtonensis
Lionepha osculans
Lionepha erasa
Lionepha disjuncta
Lionepha chintimini DNA4002 FarRef
Lionepha chintimini DNA4002 NearRef
Lionepha chintimini DNA4059 PCR
Lionepha chintimini DNA4002 DeNovo
Bembidion obtusum
Bembidion iridescens
Bembidion trechiforme
Bembidion planatum
Amerizus (Tiruka) sp.
Amerizus wingatei
Asaphidion yukonense
Asaphidion alaskanum
Asaphidion curtum
Bembidion (Hoquedela) sp. 1
Bembidion cf. csikii
Bembidion wickhami
Bembidion biguttatum
Bembidion cf. exquisitium
Bembidion escherichi
Bembidion lunulatum
Bembidion variegatum
Bembidion dentellum
Bembidion immaturum
Bembidion "Inuvik" DNA3285 DeNovo
Bembidion "Inuvik" DNA3285 FarRef
Bembidion "Inuvik" DNA3984 PCR
Bembidion "Inuvik" DNA3984 FarRef
Bembidion "Inuvik" DNA3285 NearRef
Bembidion "Inuvik" DNA3984 NearRef
Bembidion luridicorne
Bembidion properans
Bembidion cf. incisum
Bembidion interventor
Bembidion levigatum
Bembidion lenae
Bembidion tetracolum
Bembidion (Hypsipezum) sp. 1
Bembidion tigrinum
Bembidion commotum
Bembidion cf. "Desert Spotted" DNA3978 FarRef
Bembidion cf. "Desert Spotted" DNA3978 NearRef
Bembidion "Desert Spotted" DNA2786 PCR
Bembidion cf. "Desert Spotted" DNA3978 DeNovo
Bembidion plagiatum
Bembidion sejunctum
Bembidion lugubre
Bembidion mexicanum
Bembidion perspicuum
Bembidion sp. nr. transversale DNA3205 FarRef
Bembidion sp. nr. transversale DNA3205 DeNovo
Bembidion sp. nr. transversale DNA3205 NearRef
Bembidion transversale
Bembidion sp. nr. transversale DNA3021 FarRef
Bembidion sp. nr. transversale DNA3021 DeNovo
Bembidion sp. nr. transversale DNA3021 NearRef
Bembidion callosum
Bembidion laterale
Bembidion laetum
Bembidion mckinleyi carneum
Bembidion punctulatum
Bembidion ruficolle
Bembidion lachnophoroides DNA3022 NearRef
Bembidion lachnophoroides DNA3022 DeNovo
Bembidion lachnophoroides DNA3022 FarRef
Bembidion genei illigeri
Bembidion bimaculatum
Bembidion liangi
Bembidion astrabadense
Bembidion geniculatum
Bembidion grapii
Bembidion platynoides
Bembidion basicorne
Bembidion hastii
Bembidion gebleri
Bembidion simplex
Bembidion breve
Bembidion kuprianovii
Bembidion horni
Bembidion festivum
Bembidion mundum
Bembidion orion DNA2831 DeNovo
Bembidion orion DNA2831 FarRef
Bembidion orion DNA2831 NearRef
Bembidion orion DNA3079 PCR
Bembidion orion DNA3079 DeNovo
Bembidion orion DNA3079 FarRef
Bembidion orion DNA3079 NearRef
Bembidion mimekara
Bembidion planum
Bembidion concolor
Bembidion californicum
Bembidion humboldtense
Bembidion semistriatum
Bembidion aenulum
Bembidion bowditchii
Bembidion coxendix
Bembidion confusum
Bembidion balli
Bembidion inaequale
Bembidion levettei carrianum
Bembidion persimile
Bembidion bifossulatum
Bembidion americanum
Bembidion rothfelsi
Bembidion chalceum
Bembidion louisella
Bembidion honestum
Bembidion integrum
Bembidion rufotinctum
Bembidion paraenulum
Bembidion subfusum DNA3977 FarRef
Bembidion subfusum DNA3977 DeNovo
Bembidion subfusum DNA3977 NearRef
Bembidion carinula
Bembidion lapponicum DNA1604 PCR
Bembidion lapponicum DNA3974 FarRef
Bembidion lapponicum DNA3974 DeNovo
Bembidion lapponicum DNA3974 NearRef
Bembidion tairuense
Bembidion quadrimaculatum
Bembidion caoduroi
Bembidion solieri
Bembidion sp. nr. ugartei
Bembidion dorsale
Bembidion variolosum
Bembidion approximatum
Bembidion flohri
Bembidion varium
Bembidion castor
Bembidion niloticum
Bembidion poculare
Bembidion octomaculatum
Bembidion constricticolle
Bembidion affine
Bembidion cf. cognatum
Bembidion versicolor
Bembidion elizabethae
Bembidion frontale
Bembidion jacksoniense
Bembidion scintillans
Bembidion aratum
Bembidion rapidum
Bembidion "Arica" DNA3242 FarRef
Bembidion "Arica" DNA3242 DeNovo
Bembidion "Arica" DNA3242 NearRef
Bembidion mandibulare
Bembidion rufoplagiatum
Bembidion sp. nr. chilense
Bembidion (Zecillenus) sp. 1
Bembidion errans
Bembidion proprium
Bembidion orregoi
Bembidion (Chilioperyphus) sp. "Cal"
Bembidion mendocinum
Bembidion (Nothocys) sp. 1
Bembidion assimile
Bembidion nigrivestis
Bembidion salinarium
Bembidion fortestriatum
Bembidion pseudocautum
Bembidion anchonoderum
Bembidion parviceps
Bembidion musae DNA3239 FarRef
Bembidion musae DNA3239 DeNovo
Bembidion musae DNA3239 NearRef
Bembidion vile
Bembidion ephippium
Bembidion ionae
Bembidion anthracinum
Bembidion rogersi
Bembidion melanopodum
Bembidion (Omotaphus) sp. 1
Bembidion rugosellum
Bembidion sexfoveatum
COI
de novo, near ref, and
far ref assemblies separate

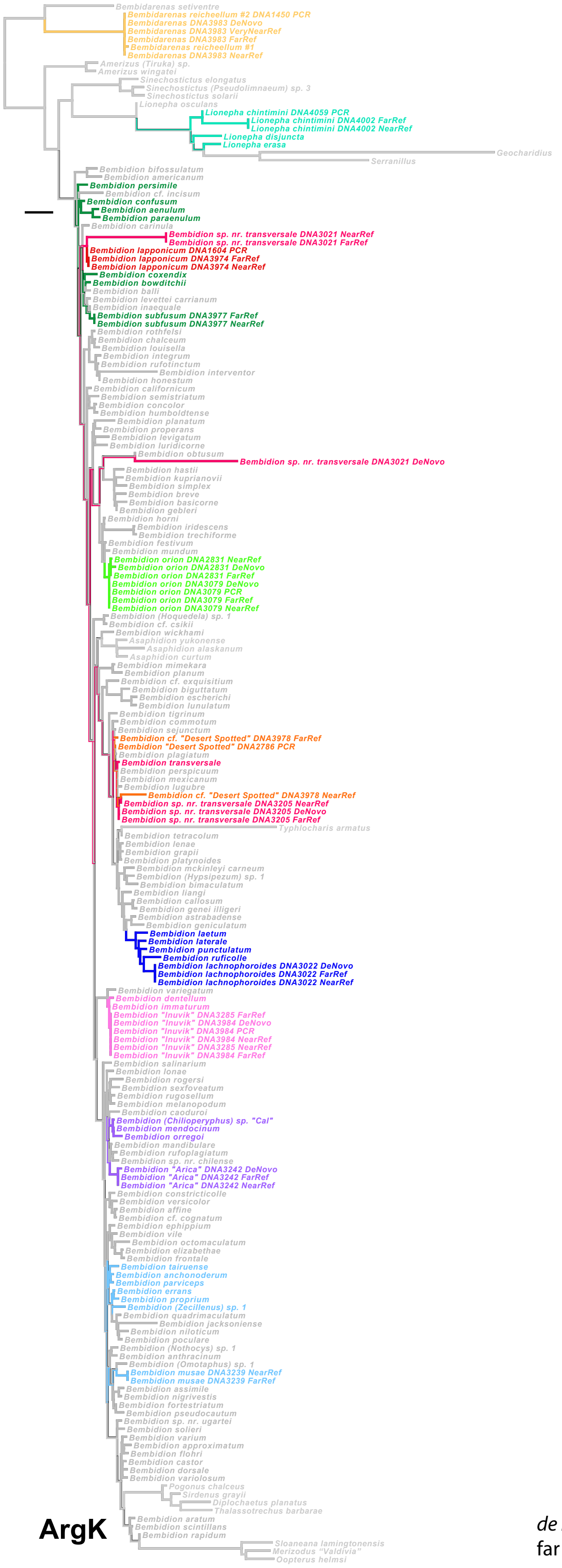

de novo, near ref, and far ref assemblies separated

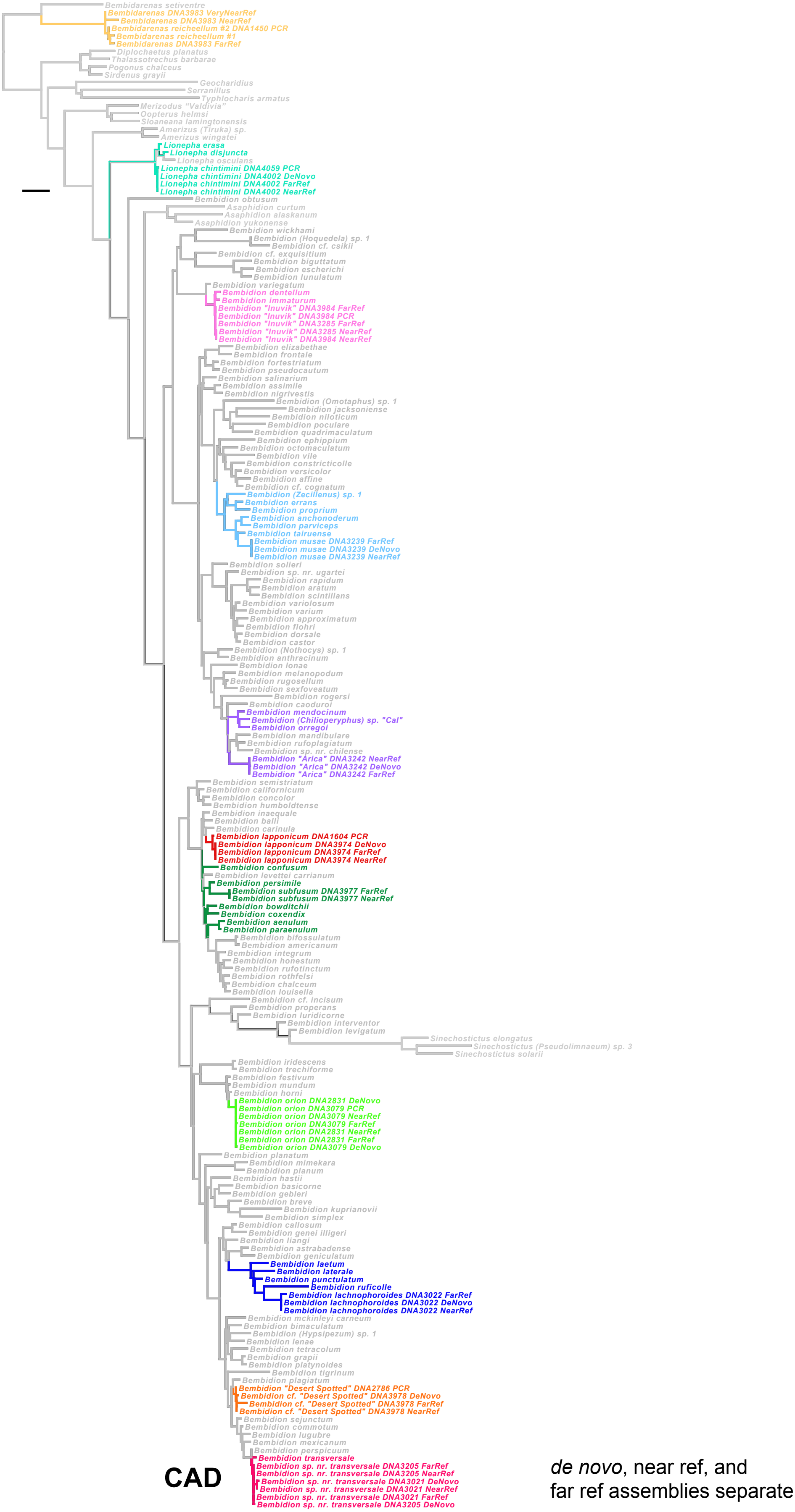
Bembidarenas setiventre
Bembidarenas DNA3983 VeryNearRef
Bembidarenas DNA3983 NearRef
Bembidarenas reicheellum #2 DNA1450 PCR
Bembidarenas reicheellum #1
Bembidarenas DNA3983 FarRef
Diplochaetus planatus
Thalassotrechus barbarae
Pogonus chalceus
Sirdenus grayii
Geocharidius
Serranillus
Typhlocharis armatus
Merizodus "Valdivia"
Oopterus helmsi
Sloaneana lamingtonensis
Amerizus (Tiruka) sp.
Amerizus wingatei
Lionepha erasa
Lionepha disjuncta
Lionepha osculans
Lionepha chintimini DNA4059 PCR
Lionepha chintimini DNA4002 DeNovo
Lionepha chintimini DNA4002 FarRef
Lionepha chintimini DNA4002 NearRef
Bembidion obtusum
Asaphidion curtum
Asaphidion alaskanum
Asaphidion yukonense
Bembidion wickhami
Bembidion (Hoquedela) sp. 1
Bembidion cf. csikii
Bembidion cf. exquisitium
Bembidion biguttatum
Bembidion escherichi
Bembidion lunulatum
Bembidion variegatum
Bembidion dentellum
Bembidion immaturum
Bembidion "Inuvik" DNA3984 FarRef
Bembidion "Inuvik" DNA3984 PCR
Bembidion "Inuvik" DNA3285 FarRef
Bembidion "Inuvik" DNA3285 NearRef
Bembidion "Inuvik" DNA3984 NearRef
Bembidion elizabethae
Bembidion frontale
Bembidion fortestriatum
Bembidion pseudocautum
Bembidion salinarium
Bembidion assimile
Bembidion nigrivestis
Bembidion (Omotaphus) sp. 1
Bembidion jacksoniense
Bembidion niloticum
Bembidion poculare
Bembidion quadrimaculatum
Bembidion ephippium
Bembidion octomaculatum
Bembidion vile
Bembidion constricticolle
Bembidion versicolor
Bembidion affine
Bembidion cf. cognatum
Bembidion (Zecillenus) sp. 1
Bembidion errans
Bembidion proprium
Bembidion anchonoderum
Bembidion parviceps
Bembidion tairuense
Bembidion musae DNA3239 FarRef
Bembidion musae DNA3239 DeNovo
Bembidion musae DNA3239 NearRef
Bembidion solieri
Bembidion sp. nr. ugartei
Bembidion rapidum
Bembidion aratum
Bembidion scintillans
Bembidion variolosum
Bembidion varium
Bembidion approximatum
Bembidion flohri
Bembidion dorsale
Bembidion castor
Bembidion (Nothocys) sp. 1
Bembidion anthracinum
Bembidion lonae
Bembidion melanopodum
Bembidion rugosellum
Bembidion sexfoveatum
Bembidion rogersi
Bembidion caoduroi
Bembidion mendocinum
Bembidion (Chilioperyphus) sp. "Cal"
Bembidion orregoi
Bembidion mandibulare
Bembidion rufoplagiatum
Bembidion sp. nr. chilense
Bembidion "Arica" DNA3242 NearRef
Bembidion "Arica" DNA3242 DeNovo
Bembidion "Arica" DNA3242 FarRef
Bembidion semistriatum
Bembidion californicum
Bembidion concolor
Bembidion humboldtense
Bembidion inaequale
Bembidion balli
Bembidion carinula
Bembidion lapponicum DNA1604 PCR
Bembidion lapponicum DNA3974 DeNovo
Bembidion lapponicum DNA3974 FarRef
Bembidion lapponicum DNA3974 NearRef
Bembidion confusum
Bembidion levettei carrianum
Bembidion persimile
Bembidion subfusum DNA3977 FarRef
Bembidion subfusum DNA3977 NearRef
Bembidion bowditchii
Bembidion coxendix
Bembidion aenulum
Bembidion paraenulum
Bembidion bifossulatum
Bembidion americanum
Bembidion integrum
Bembidion honestum
Bembidion rufotinctum
Bembidion rothfelsi
Bembidion chalceum
Bembidion louisella
Bembidion cf. incisum
Bembidion properans
Bembidion luridicorne
Bembidion interventor
Bembidion levigatum
Sinechostictus elongatus
Sinechostictus (Pseudolimnaeum) sp. 3
Sinechostictus solarii
Bembidion iridescens
Bembidion trechiforme
Bembidion festivum
Bembidion mundum
Bembidion horni
Bembidion orion DNA2831 DeNovo
Bembidion orion DNA3079 PCR
Bembidion orion DNA3079 NearRef
Bembidion orion DNA3079 FarRef
Bembidion orion DNA2831 NearRef
Bembidion orion DNA2831 FarRef
Bembidion orion DNA3079 DeNovo
Bembidion planatum
Bembidion mimekara
Bembidion planum
Bembidion hastii
Bembidion basicorne
Bembidion gebleri
Bembidion breve
Bembidion kuprianovii
Bembidion simplex
Bembidion callosum
Bembidion genei illigeri
Bembidion liangi
Bembidion astrabadense
Bembidion geniculatum
Bembidion laetum
Bembidion laterale
Bembidion punctulatum
Bembidion ruficolle
Bembidion lachnophoroides DNA3022 FarRef
Bembidion lachnophoroides DNA3022 DeNovo
Bembidion lachnophoroides DNA3022 NearRef
Bembidion mckinleyi carneum
Bembidion bimaculatum
Bembidion (Hypsipezum) sp. 1
Bembidion lenae
Bembidion tetracolum
Bembidion grapii
Bembidion platynoides
Bembidion tigrinum
Bembidion plagiatum
Bembidion "Desert Spotted" DNA2786 PCR
Bembidion cf. "Desert Spotted" DNA3978 DeNovo
Bembidion cf. "Desert Spotted" DNA3978 FarRef
Bembidion cf. "Desert Spotted" DNA3978 NearRef
Bembidion sejunctum
Bembidion commotum
Bembidion lugubre
Bembidion mexicanum
Bembidion perspicuum
Bembidion transversale
Bembidion sp. nr. transversale DNA3205 FarRef
Bembidion sp. nr. transversale DNA3205 NearRef
Bembidion sp. nr. transversale DNA3021 DeNovo
Bembidion sp. nr. transversale DNA3021 NearRef
Bembidion sp. nr. transversale DNA3021 FarRef
Bembidion sp. nr. transversale DNA3205 DeNovo
CAD
de novo, near ref, and
far ref assemblies separate

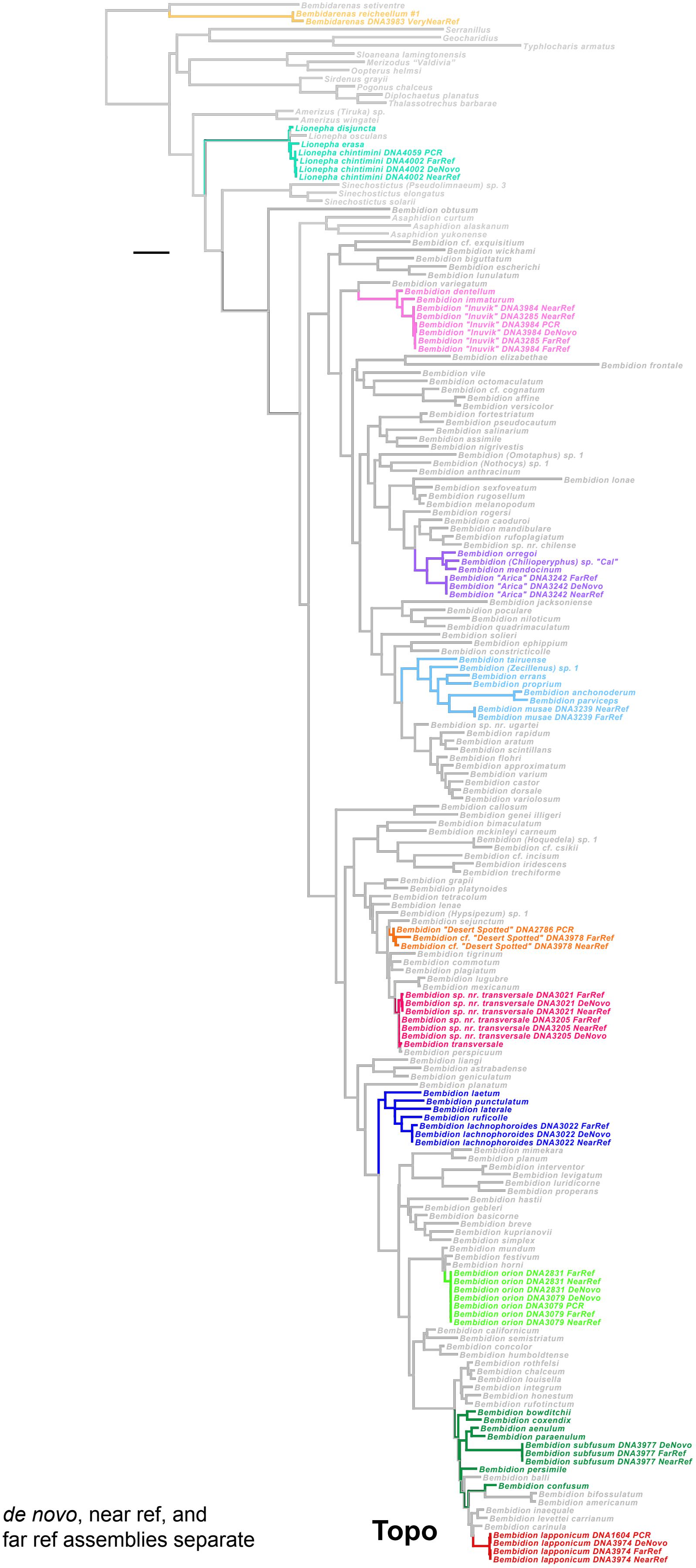
Bembidarenas setiventre
Bembidarenas reicheellum #1
Bembidarenas DNA3983 VeryNearRef
Serranillus
Geocharidius
Typhlocharis armatus
Sloaneana lamingtonensis
Merizodus "Valdivia"
Oopterus helmsi
Sirdenus grayii
Pogonus chalceus
Diplochaetus planatus
Thalassotrechus barbarae
Amerizus (Tiruka) sp.
Amerizus wingatei
Lionepha disjuncta
Lionepha osculans
Lionepha erasa
Lionepha chintimini DNA4059 PCR
Lionepha chintimini DNA4002 FarRef
Lionepha chintimini DNA4002 DeNovo
Lionepha chintimini DNA4002 NearRef
Sinechostictus (Pseudolimnaeum) sp. 3
Sinechostictus elongatus
Sinechostictus solarii
Bembidion obtusum
Asaphidion curtum
Asaphidion alaskanum
Asaphidion yukonense
Bembidion cf. exquisitium
Bembidion wickhami
Bembidion biguttatum
Bembidion escherichi
Bembidion lunulatum
Bembidion variegatum
Bembidion dentellum
Bembidion immaturum
Bembidion "Inuvik" DNA3984 NearRef
Bembidion "Inuvik" DNA3285 NearRef
Bembidion "Inuvik" DNA3984 PCR
Bembidion "Inuvik" DNA3984 DeNovo
Bembidion "Inuvik" DNA3285 FarRef
Bembidion "Inuvik" DNA3984 FarRef
Bembidion elizabethae
Bembidion frontale
Bembidion vile
Bembidion octomaculatum
Bembidion cf. cognatum
Bembidion affine
Bembidion versicolor
Bembidion fortestriatum
Bembidion pseudocautum
Bembidion salinarium
Bembidion assimile
Bembidion nigrivestis
Bembidion (Omotaphus) sp. 1
Bembidion (Nothocys) sp. 1
Bembidion anthracinum
Bembidion lonae
Bembidion sexfoveatum
Bembidion rugosellum
Bembidion melanopodum
Bembidion rogersi
Bembidion caoduroi
Bembidion mandibulare
Bembidion rufoplagiatum
Bembidion sp. nr. chilense
Bembidion orregoi
Bembidion (Chilioperyphus) sp. "Cal"
Bembidion mendocinum
Bembidion "Arica" DNA3242 FarRef
Bembidion "Arica" DNA3242 DeNovo
Bembidion "Arica" DNA3242 NearRef
Bembidion jacksoniense
Bembidion poculare
Bembidion niloticum
Bembidion quadrimaculatum
Bembidion solieri
Bembidion ephippium
Bembidion constricticolle
Bembidion tairuense
Bembidion (Zecillenus) sp. 1
Bembidion errans
Bembidion proprium
Bembidion anchonoderum
Bembidion parviceps
Bembidion musae DNA3239 NearRef
Bembidion musae DNA3239 FarRef
Bembidion sp. nr. ugartei
Bembidion rapidum
Bembidion aratum
Bembidion scintillans
Bembidion flohri
Bembidion approximatum
Bembidion varium
Bembidion castor
Bembidion dorsale
Bembidion variolosum
Bembidion callosum
Bembidion genei illigeri
Bembidion bimaculatum
Bembidion mckinleyi carneum
Bembidion (Hoquedela) sp. 1
Bembidion cf. csikii
Bembidion cf. incisum
Bembidion iridescens
Bembidion trechiforme
Bembidion grapii
Bembidion platynoides
Bembidion tetracolum
Bembidion lenae
Bembidion (Hypsipezum) sp. 1
Bembidion sejunctum
Bembidion "Desert Spotted" DNA2786 PCR
Bembidion cf. "Desert Spotted" DNA3978 FarRef
Bembidion cf. "Desert Spotted" DNA3978 NearRef
Bembidion tigrinum
Bembidion commotum
Bembidion plagiatum
Bembidion lugubre
Bembidion mexicanum
Bembidion sp. nr. transversale DNA3021 FarRef
Bembidion sp. nr. transversale DNA3021 DeNovo
Bembidion sp. nr. transversale DNA3021 NearRef
Bembidion sp. nr. transversale DNA3205 FarRef
Bembidion sp. nr. transversale DNA3205 NearRef
Bembidion sp. nr. transversale DNA3205 DeNovo
Bembidion transversale
Bembidion perspicuum
Bembidion liangi
Bembidion astrabadense
Bembidion geniculatum
Bembidion planatum
Bembidion laetum
Bembidion punctulatum
Bembidion laterale
Bembidion ruficolle
Bembidion lachnophoroides DNA3022 FarRef
Bembidion lachnophoroides DNA3022 DeNovo
Bembidion lachnophoroides DNA3022 NearRef
Bembidion mimekara
Bembidion planum
Bembidion interventor
Bembidion levigatum
Bembidion luridicorne
Bembidion properans
Bembidion hastii
Bembidion gebleri
Bembidion basicorne
Bembidion breve
Bembidion kuprianovii
Bembidion simplex
Bembidion mundum
Bembidion festivum
Bembidion horni
Bembidion orion DNA2831 FarRef
Bembidion orion DNA2831 NearRef
Bembidion orion DNA2831 DeNovo
Bembidion orion DNA3079 DeNovo
Bembidion orion DNA3079 PCR
Bembidion orion DNA3079 FarRef
Bembidion orion DNA3079 NearRef
Bembidion californicum
Bembidion semistriatum
Bembidion concolor
Bembidion humboldtense
Bembidion rothfelsi
Bembidion chalceum
Bembidion louisella
Bembidion integrum
Bembidion honestum
Bembidion rufotinctum
Bembidion bowditchii
Bembidion coxendix
Bembidion aenulum
Bembidion paraenulum
Bembidion subfusum DNA3977 DeNovo
Bembidion subfusum DNA3977 FarRef
Bembidion subfusum DNA3977 NearRef
Bembidion persimile
Bembidion balli
Bembidion confusum
Bembidion bifossulatum
Bembidion americanum
Bembidion inaequale
Bembidion levettei carrianum
Bembidion carinula
Bembidion lapponicum DNA1604 PCR
Bembidion lapponicum DNA3974 DeNovo
Bembidion lapponicum DNA3974 FarRef
Bembidion lapponicum DNA3974 NearRef
de novo, near ref, and
far ref assemblies separate
Topo

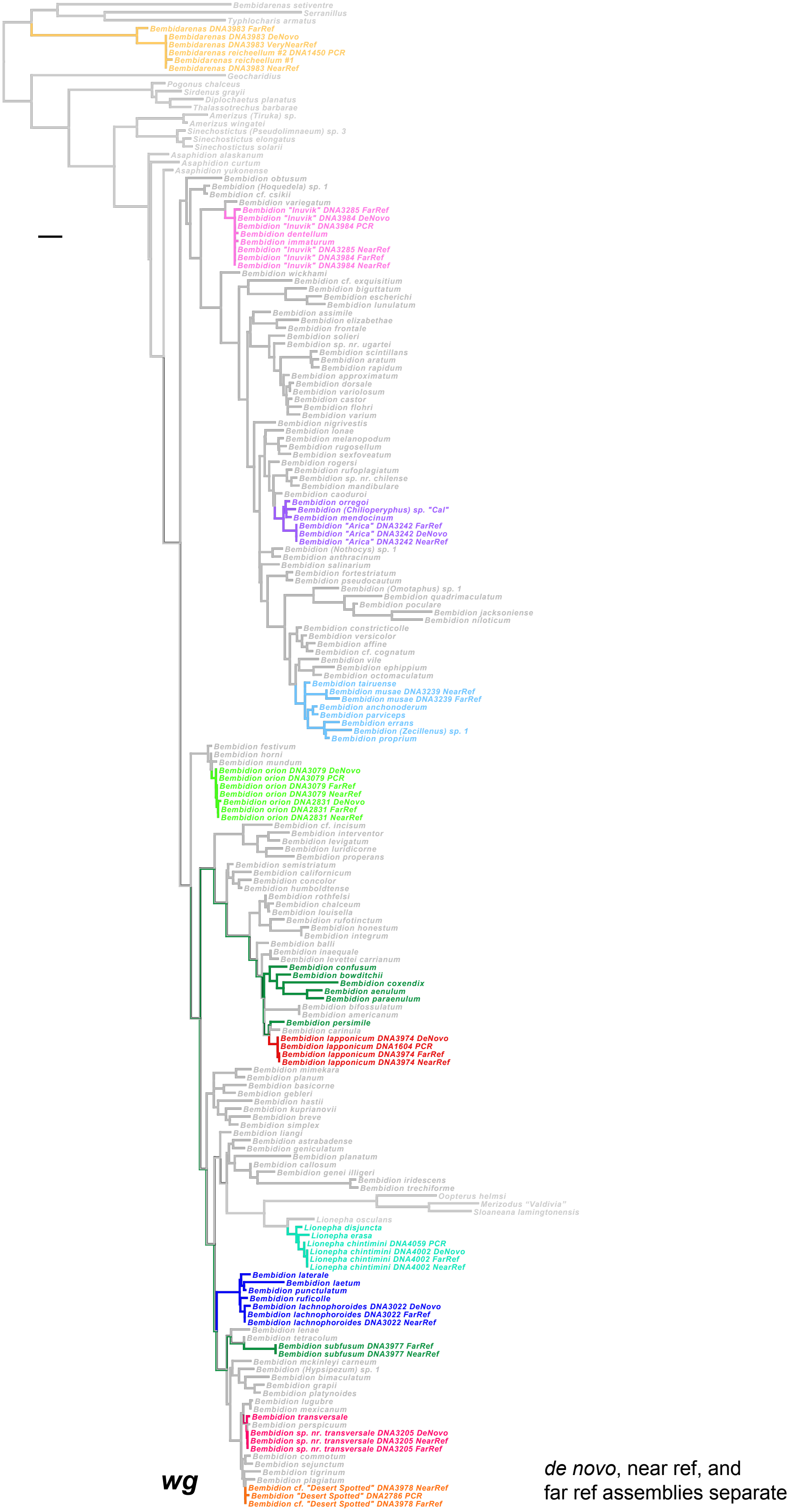

Bembidarenas setiventre
Serranillus
Typhlocharis armatus
Bembidarenas DNA3983 FarRef
Bembidarenas DNA3983 DeNovo
Bembidarenas DNA3983 VeryNearRef
Bembidarenas reicheellum #2 DNA1450 PCR
Bembidarenas reicheellum #1
Bembidarenas DNA3983 NearRef
Geocharidius
Pogonus chalceus
Sirdenus grayii
Diplochaetus planatus
Thalassotrechus barbarae
Amerizus (Tiruka) sp.
Amerizus wingatei
Sinechostictus (Pseudolimnaeum) sp. 3
Sinechostictus elongatus
Sinechostictus solarii
Asaphidion alaskanum
Asaphidion curtum
Asaphidion yukonense
Bembidion obtusum
Bembidion (Hoquedela) sp. 1
Bembidion cf. csikii
Bembidion variegatum
Bembidion "Inuvik" DNA3285 FarRef
Bembidion "Inuvik" DNA3984 DeNovo
Bembidion "Inuvik" DNA3984 PCR
Bembidion dentellum
Bembidion immaturum
Bembidion "Inuvik" DNA3285 NearRef
Bembidion "Inuvik" DNA3984 FarRef
Bembidion "Inuvik" DNA3984 NearRef
Bembidion wickhami
Bembidion cf. exquisitium
Bembidion biguttatum
Bembidion escherichi
Bembidion lunulatum
Bembidion assimile
Bembidion elizabethae
Bembidion frontale
Bembidion solieri
Bembidion sp. nr. ugartei
Bembidion scintillans
Bembidion aratum
Bembidion rapidum
Bembidion approximatum
Bembidion dorsale
Bembidion variolosum
Bembidion castor
Bembidion flohri
Bembidion varium
Bembidion nigrivestis
Bembidion lonae
Bembidion melanopodum
Bembidion rugosellum
Bembidion sexfoveatum
Bembidion rogersi
Bembidion rufoplagiatum
Bembidion sp. nr. chilense
Bembidion mandibulare
Bembidion caoduroi
Bembidion orregoi
Bembidion (Chilioperyphus) sp. "Cal"
Bembidion mendocinum
Bembidion "Arica" DNA3242 FarRef
Bembidion "Arica" DNA3242 DeNovo
Bembidion "Arica" DNA3242 NearRef
Bembidion (Nothocys) sp. 1
Bembidion anthracinum
Bembidion salinarium
Bembidion fortestriatum
Bembidion pseudocautum
Bembidion (Omotaphus) sp. 1
Bembidion quadrimaculatum
Bembidion poculare
Bembidion jacksoniense
Bembidion niloticum
Bembidion constricticolle
Bembidion versicolor
Bembidion affine
Bembidion cf. cognatum
Bembidion vile
Bembidion ephippium
Bembidion octomaculatum
Bembidion tairuense
Bembidion musae DNA3239 NearRef
Bembidion musae DNA3239 FarRef
Bembidion anchonoderum
Bembidion parviceps
Bembidion errans
Bembidion (Zecillenus) sp. 1
Bembidion proprium
Bembidion festivum
Bembidion horni
Bembidion mundum
Bembidion orion DNA3079 DeNovo
Bembidion orion DNA3079 PCR
Bembidion orion DNA3079 FarRef
Bembidion orion DNA3079 NearRef
Bembidion orion DNA2831 DeNovo
Bembidion orion DNA2831 FarRef
Bembidion orion DNA2831 NearRef
Bembidion cf. incisum
Bembidion interventor
Bembidion levigatum
Bembidion luridicorne
Bembidion properans
Bembidion semistriatum
Bembidion californicum
Bembidion concolor
Bembidion humboldtense
Bembidion rothfelsi
Bembidion chalceum
Bembidion louisella
Bembidion rufotinctum
Bembidion honestum
Bembidion integrum
Bembidion balli
Bembidion inaequale
Bembidion levettei carrianum
Bembidion confusum
Bembidion bowditchii
Bembidion coxendix
Bembidion aenulum
Bembidion paraenulum
Bembidion bifossulatum
Bembidion americanum
Bembidion persimile
Bembidion carinula
Bembidion lapponicum DNA3974 DeNovo
Bembidion lapponicum DNA1604 PCR
Bembidion lapponicum DNA3974 FarRef
Bembidion lapponicum DNA3974 NearRef
Bembidion mimekara
Bembidion planum
Bembidion basicorne
Bembidion gebleri
Bembidion hastii
Bembidion kuprianovii
Bembidion breve
Bembidion simplex
Bembidion liangi
Bembidion astrabadense
Bembidion geniculatum
Bembidion planatum
Bembidion callosum
Bembidion genei illigeri
Bembidion iridescens
Bembidion trechiforme
Oopterus helmsi
Merizodus “Valdivia”
Sloaneana lamingtonensis
Lionepha osculans
Lionepha disjuncta
Lionepha erasa
Lionepha chintimini DNA4059 PCR
Lionepha chintimini DNA4002 DeNovo
Lionepha chintimini DNA4002 FarRef
Lionepha chintimini DNA4002 NearRef
Bembidion laterale
Bembidion laetum
Bembidion punctulatum
Bembidion ruficolle
Bembidion lachnophoroides DNA3022 DeNovo
Bembidion lachnophoroides DNA3022 FarRef
Bembidion lachnophoroides DNA3022 NearRef
Bembidion lenae
Bembidion tetracolum
Bembidion subfusum DNA3977 FarRef
Bembidion subfusum DNA3977 NearRef
Bembidion mckinleyi carneum
Bembidion (Hypsipezum) sp. 1
Bembidion bimaculatum
Bembidion grapii
Bembidion platynoides
Bembidion lugubre
Bembidion mexicanum
Bembidion transversale
Bembidion perspicuum
Bembidion sp. nr. transversale DNA3205 DeNovo
Bembidion sp. nr. transversale DNA3205 NearRef
Bembidion sp. nr. transversale DNA3205 FarRef
Bembidion commotum
Bembidion sejunctum
Bembidion tigrinum
Bembidion plagiatum
Bembidion cf. "Desert Spotted" DNA3978 NearRef
Bembidion "Desert Spotted" DNA2786 PCR
Bembidion cf. "Desert Spotted" DNA3978 FarRef
wg
de novo, near ref, and
far ref assemblies separate
