# Supplementary material for: Successful Recovery of Nuclear Protein-Coding Genes from Small Insects in Museums Using Illumina Sequencing
Source: PLoS One. 2015 Dec 30;10(12):e0143929. doi: 10.1371/journal.pone.0143929 (PMC4696846; doi:10.1371/journal.pone.0143929)
Supplement: S7 Fig — The placement of the DeNovo, NearRef, and FarRef sequences is shown relative to their prediction groups. Branches and taxon names of all specimens in the prediction group are indicated with a unique color. (PDF) [file pone.0143929.s007.pdf]

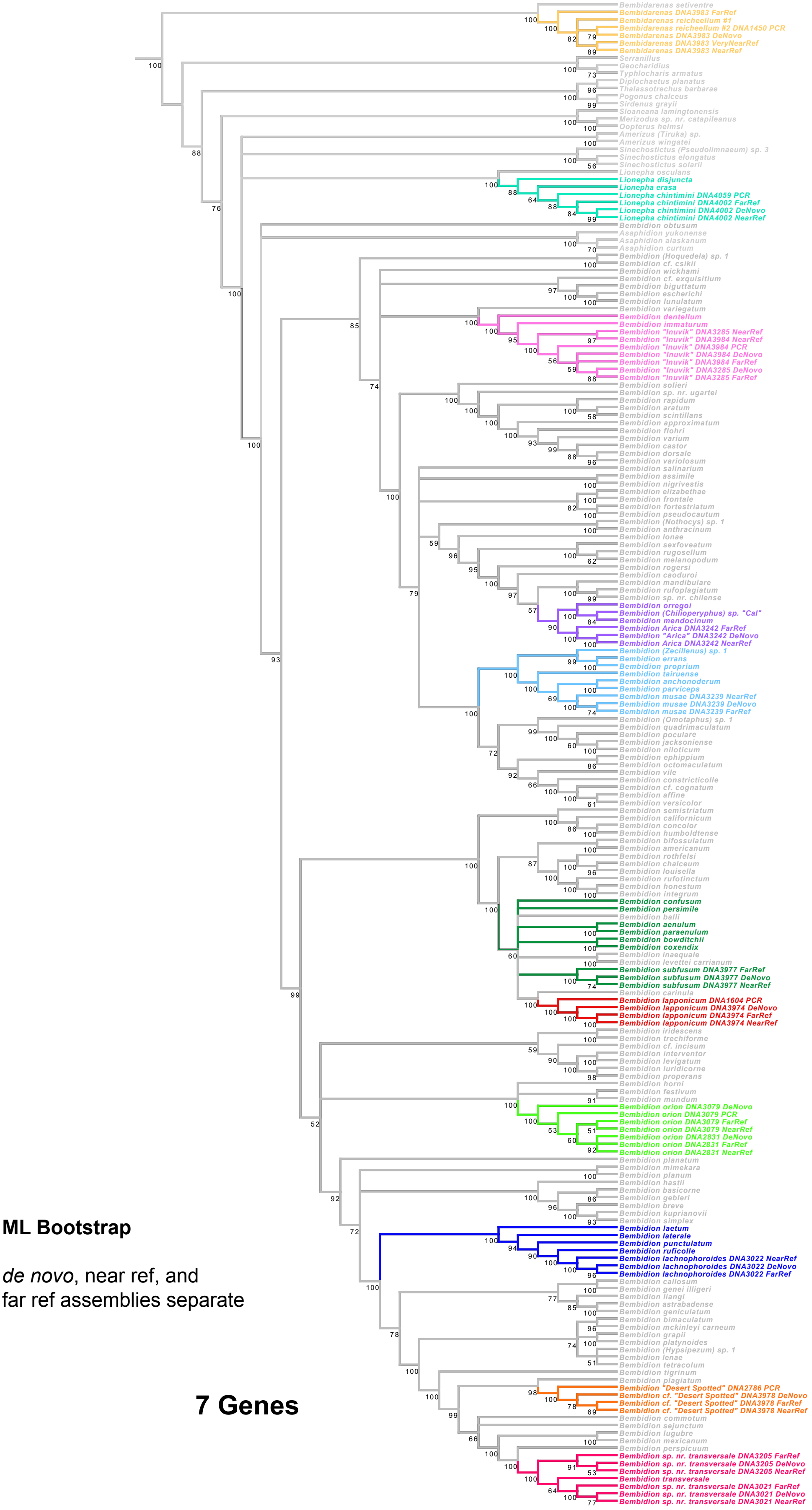
ML Bootstrap
de novo, near ref, and far ref assemblies separate
7 Genes
Bembidarenas setiventre
Bembidarenas DNA3983 FarRef
Bembidarenas reicheellum #1
Bembidarenas reicheellum #2 DNA1450 PCR
Bembidarenas DNA3983 DeNovo
Bembidarenas DNA3983 VeryNearRef
Bembidarenas DNA3983 NearRef
Serranillus
Geocharidius
Typhlocharis armatus
Diplochaetus planatus
Thalassotrechus barbarae
Pogonus chalceus
Sirdenus grayii
Sloaneana tamingtonensis
Merizodus sp. nr. catapileanus
Oopterus helmsi
Amerizus (Tiruka) sp.
Amerizus wingatei
Sinechostictus (Pseudolimnaeum) sp. 3
Sinechostictus elongatus
Sinechostictus solarii
Lionepha osculans
Lionepha disjuncta
Lionepha erasa
Lionepha chintimini DNA4059 PCR
Lionepha chintimini DNA4002 FarRef
Lionepha chintimini DNA4002 DeNovo
Lionepha chintimini DNA4002 NearRef
Bembidion obtusum
Asaphidion yukonense
Asaphidion alaskanum
Asaphidion curtum
Bembidion (Hoquedela) sp. 1
Bembidion cf. csikii
Bembidion wickhami
Bembidion cf. exquisitum
Bembidion biguttatum
Bembidion escherichi
Bembidion lunulatum
Bembidion variegatum
Bembidion dentellum
Bembidion immaturum
Bembidion "Inuvik" DNA3285 NearRef
Bembidion "Inuvik" DNA3984 NearRef
Bembidion "Inuvik" DNA3984 PCR
Bembidion "Inuvik" DNA3984 DeNovo
Bembidion "Inuvik" DNA3984 FarRef
Bembidion "Inuvik" DNA3285 DeNovo
Bembidion "Inuvik" DNA3285 FarRef
Bembidion solieri
Bembidion sp. nr. ugartei
Bembidion rapidum
Bembidion aratum
Bembidion scintillans
Bembidion approximatum
Bembidion flohri
Bembidion varium
Bembidion castor
Bembidion dorsale
Bembidion variolosum
Bembidion salinarium
Bembidion assimile
Bembidion nigrivestis
Bembidion elizabethae
Bembidion frontale
Bembidion fortestriatum
Bembidion pseudocautum
Bembidion (Nothocys) sp. 1
Bembidion anthracinum
Bembidion lonae
Bembidion sexfoveatum
Bembidion rugosellum
Bembidion melanopodum
Bembidion rogersi
Bembidion caoduroi
Bembidion mandibulare
Bembidion rufoplagiatum
Bembidion sp. nr. chilense
Bembidion orregoi
Bembidion (Chilioperyphus) sp. "Cal"
Bembidion mendocinum
Bembidion Arica DNA3242 FarRef
Bembidion "Arica" DNA3242 DeNovo
Bembidion Arica DNA3242 NearRef
Bembidion (Zecillenus) sp. 1
Bembidion errans
Bembidion proprium
Bembidion tairuense
Bembidion anchonoderum
Bembidion parviceps
Bembidion musae DNA3239 NearRef
Bembidion musae DNA3239 DeNovo
Bembidion musae DNA3239 FarRef
Bembidion (Omotaphus) sp. 1
Bembidion quadrimaculatum
Bembidion poculare
Bembidion jacksoniense
Bembidion niloticum
Bembidion ephippium
Bembidion octomaculatum
Bembidion vile
Bembidion constricticolle
Bembidion cf. cognatum
Bembidion affine
Bembidion versicolor
Bembidion semistriatum
Bembidion californicum
Bembidion concolor
Bembidion humboldtense
Bembidion bifossulatum
Bembidion americanum
Bembidion rothfelsi
Bembidion chalceum
Bembidion louisella
Bembidion rufotinctum
Bembidion honestum
Bembidion integrum
Bembidion confusum
Bembidion persimile
Bembidion balli
Bembidion aenulum
Bembidion paraenulum
Bembidion bowditchii
Bembidion coxendix
Bembidion inaequale
Bembidion levettei carrianum
Bembidion subfusum DNA3977 FarRef
Bembidion subfusum DNA3977 DeNovo
Bembidion subfusum DNA3977 NearRef
Bembidion carinula
Bembidion lapponicum DNA1604 PCR
Bembidion lapponicum DNA3974 DeNovo
Bembidion lapponicum DNA3974 FarRef
Bembidion lapponicum DNA3974 NearRef
Bembidion iridescens
Bembidion trechiforme
Bembidion cf. incisum
Bembidion interventor
Bembidion levigatum
Bembidion luridicorne
Bembidion properans
Bembidion horni
Bembidion festivum
Bembidion mundum
Bembidion orion DNA3079 DeNovo
Bembidion orion DNA3079 PCR
Bembidion orion DNA3079 FarRef
Bembidion orion DNA3079 NearRef
Bembidion orion DNA2831 DeNovo
Bembidion orion DNA2831 FarRef
Bembidion orion DNA2831 NearRef
Bembidion planatum
Bembidion mimekara
Bembidion planum
Bembidion hastii
Bembidion basicorne
Bembidion gebleri
Bembidion breve
Bembidion kuprianovii
Bembidion simplex
Bembidion laetum
Bembidion laterale
Bembidion punctulatum
Bembidion ruficolle
Bembidion lachnophoroides DNA3022 NearRef
Bembidion lachnophoroides DNA3022 DeNovo
Bembidion lachnophoroides DNA3022 FarRef
Bembidion callosum
Bembidion genei illigeri
Bembidion liangi
Bembidion astrabadense
Bembidion geniculatum
Bembidion bimaculatum
Bembidion mckinleyi carneum
Bembidion grapii
Bembidion platynoides
Bembidion (Hypsipezum) sp. 1
Bembidion lenae
Bembidion tetracolum
Bembidion tigrinum
Bembidion plagiatum
Bembidion "Desert Spotted" DNA2786 PCR
Bembidion cf. "Desert Spotted" DNA3978 DeNovo
Bembidion cf. "Desert Spotted" DNA3978 FarRef
Bembidion cf. "Desert Spotted" DNA3978 NearRef
Bembidion commotum
Bembidion sejunctum
Bembidion lugubre
Bembidion mexicanum
Bembidion perspicuum
Bembidion sp. nr. transversale DNA3205 FarRef
Bembidion sp. nr. transversale DNA3205 DeNovo
Bembidion sp. nr. transversale DNA3205 NearRef
Bembidion transversale
Bembidion sp. nr. transversale DNA3021 FarRef
Bembidion sp. nr. transversale DNA3021 DeNovo
Bembidion sp. nr. transversale DNA3021 NearRef

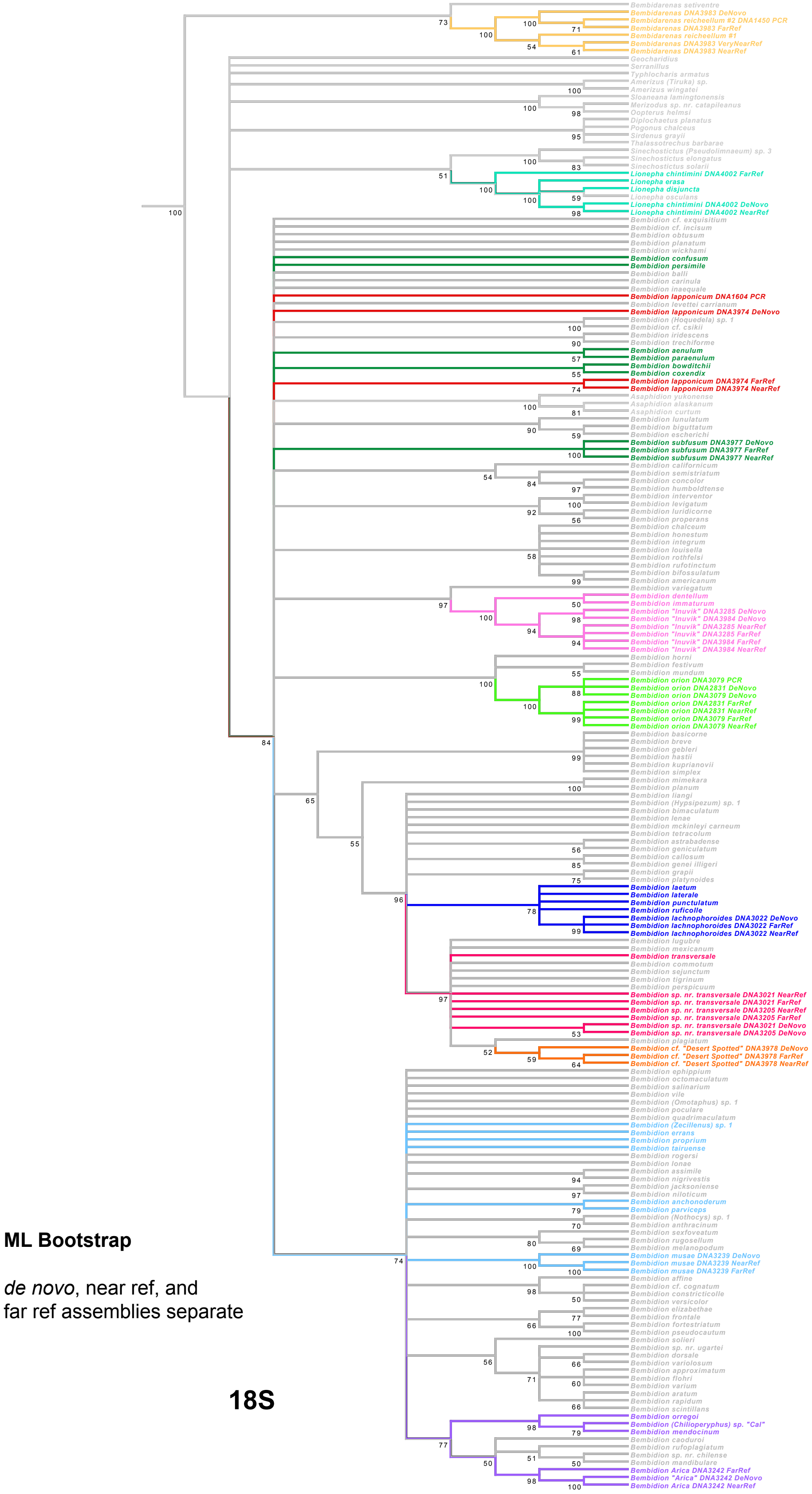

Bembidarenas setiventre
Bembidarenas DNA3983 DeNovo
Bembidarenas reicheellum #2 DNA1450 PCR
Bembidarenas DNA3983 FarRef
Bembidarenas reicheellum #1
Bembidarenas DNA3983 VeryNearRef
Bembidarenas DNA3983 NearRef
Geocharidius
Serranillus
Typhlocharis armatus
Amerizus (Tiruka) sp.
Amerizus wingatei
Sloaneana lamingtonensis
Merizodus sp. nr. catapileanus
Oopterus helmsi
Diplochaetus planatus
Pogonus chalceus
Sirdenus grayii
Thalassotrechus barbarae
Sinechostictus (Pseudolimnaeum) sp. 3
Sinechostictus elongatus
Sinechostictus solarii
Lionepha chintimini DNA4002 FarRef
Lionepha erasa
Lionepha disjuncta
Lionepha osculans
Lionepha chintimini DNA4002 DeNovo
Lionepha chintimini DNA4002 NearRef
Bembidion cf. exquisitium
Bembidion cf. incisum
Bembidion obtusum
Bembidion planatum
Bembidion wickhami
Bembidion confusum
Bembidion persimile
Bembidion balli
Bembidion carinula
Bembidion inaequale
Bembidion lapponicum DNA1604 PCR
Bembidion levettei carrianum
Bembidion lapponicum DNA3974 DeNovo
Bembidion (Hoquedela) sp. 1
Bembidion cf. csikii
Bembidion iridescens
Bembidion trechiforme
Bembidion aenulum
Bembidion paraenulum
Bembidion bowditchii
Bembidion coxendix
Bembidion lapponicum DNA3974 FarRef
Bembidion lapponicum DNA3974 NearRef
Asaphidion yukonense
Asaphidion alaskanum
Asaphidion curtum
Bembidion lunulatum
Bembidion biguttatum
Bembidion escherichi
Bembidion subfusum DNA3977 DeNovo
Bembidion subfusum DNA3977 FarRef
Bembidion subfusum DNA3977 NearRef
Bembidion californicum
Bembidion semistriatum
Bembidion concolor
Bembidion humboldtense
Bembidion interventor
Bembidion levigatum
Bembidion luridicorne
Bembidion properans
Bembidion chalceum
Bembidion honestum
Bembidion integrum
Bembidion louisella
Bembidion rothfelsi
Bembidion rufotinctum
Bembidion bifossulatum
Bembidion americanum
Bembidion variegatum
Bembidion dentellum
Bembidion immaturum
Bembidion "Inuvik" DNA3285 DeNovo
Bembidion "Inuvik" DNA3984 DeNovo
Bembidion "Inuvik" DNA3285 NearRef
Bembidion "Inuvik" DNA3285 FarRef
Bembidion "Inuvik" DNA3984 FarRef
Bembidion "Inuvik" DNA3984 NearRef
Bembidion horni
Bembidion festivum
Bembidion mundum
Bembidion orion DNA3079 PCR
Bembidion orion DNA2831 DeNovo
Bembidion orion DNA3079 DeNovo
Bembidion orion DNA2831 FarRef
Bembidion orion DNA2831 NearRef
Bembidion orion DNA3079 FarRef
Bembidion orion DNA3079 NearRef
Bembidion basicorne
Bembidion breve
Bembidion gebleri
Bembidion hastii
Bembidion kuprianovii
Bembidion simplex
Bembidion mimekara
Bembidion planum
Bembidion liangi
Bembidion (Hypsipezum) sp. 1
Bembidion bimaculatum
Bembidion lenae
Bembidion mckinleyi carneum
Bembidion tetracolum
Bembidion astrabadense
Bembidion geniculatum
Bembidion callosum
Bembidion genei illigeri
Bembidion grapii
Bembidion platynoides
Bembidion laetum
Bembidion laterale
Bembidion punctulatum
Bembidion ruficolle
Bembidion lachnophoroides DNA3022 DeNovo
Bembidion lachnophoroides DNA3022 FarRef
Bembidion lachnophoroides DNA3022 NearRef
Bembidion lugubre
Bembidion mexicanum
Bembidion transversale
Bembidion commotum
Bembidion sejunctum
Bembidion tigrinum
Bembidion perspicuum
Bembidion sp. nr. transversale DNA3021 NearRef
Bembidion sp. nr. transversale DNA3021 FarRef
Bembidion sp. nr. transversale DNA3205 NearRef
Bembidion sp. nr. transversale DNA3205 FarRef
Bembidion sp. nr. transversale DNA3021 DeNovo
Bembidion sp. nr. transversale DNA3205 DeNovo
Bembidion plagiatum
Bembidion cf. "Desert Spotted" DNA3978 DeNovo
Bembidion cf. "Desert Spotted" DNA3978 FarRef
Bembidion cf. "Desert Spotted" DNA3978 NearRef
Bembidion ephippium
Bembidion octomaculatum
Bembidion salinarium
Bembidion vile
Bembidion (Omotaphus) sp. 1
Bembidion poculare
Bembidion quadrimaculatum
Bembidion (Zecillenus) sp. 1
Bembidion errans
Bembidion proprium
Bembidion tairuense
Bembidion rogersi
Bembidion lonae
Bembidion assimile
Bembidion nigrivestis
Bembidion jacksoniense
Bembidion niloticum
Bembidion anchonoderum
Bembidion parviceps
Bembidion (Nothocys) sp. 1
Bembidion anthracinum
Bembidion sexfoveatum
Bembidion rugosellum
Bembidion melanopodum
Bembidion musae DNA3239 DeNovo
Bembidion musae DNA3239 NearRef
Bembidion musae DNA3239 FarRef
Bembidion affine
Bembidion cf. cognatum
Bembidion constricticolle
Bembidion versicolor
Bembidion elizabethae
Bembidion frontale
Bembidion fortestriatum
Bembidion pseudocautum
Bembidion solieri
Bembidion sp. nr. ugartei
Bembidion dorsale
Bembidion variolosum
Bembidion approximatum
Bembidion flohri
Bembidion varium
Bembidion aratum
Bembidion rapidum
Bembidion scintillans
Bembidion orregoi
Bembidion (Chilioperyphus) sp. "Cal"
Bembidion mendocinum
Bembidion caoduroi
Bembidion rufoplagiatum
Bembidion sp. nr. chilense
Bembidion mandibulare
Bembidion Arica DNA3242 FarRef
Bembidion "Arica" DNA3242 DeNovo
Bembidion Arica DNA3242 NearRef
ML Bootstrap
de novo, near ref, and
far ref assemblies separate
18S

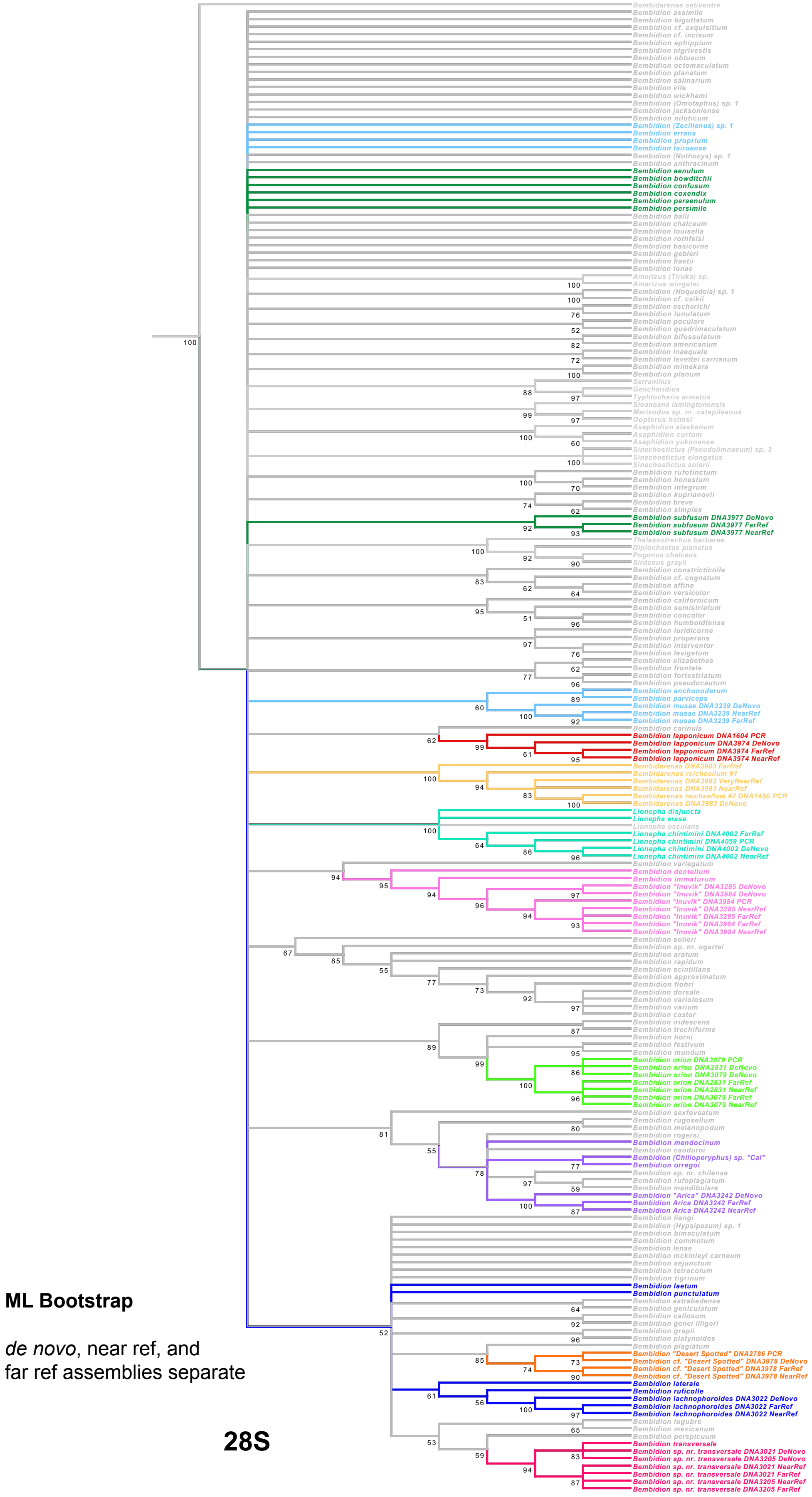
Bembidarenas setiventre
Bembidion assimile
Bembidion biguttatum
Bembidion cf. exquisitium
Bembidion cf. incisum
Bembidion ephippium
Bembidion nigrivestis
Bembidion obtusum
Bembidion octomaculatum
Bembidion planatum
Bembidion salinarium
Bembidion vile
Bembidion wickhami
Bembidion (Omotaphus) sp. 1
Bembidion jacksoniense
Bembidion niloticum
Bembidion (Zecillenus) sp. 1
Bembidion errans
Bembidion proprium
Bembidion tairuense
Bembidion (Nothocys) sp. 1
Bembidion anthracinum
Bembidion aenulum
Bembidion bowditchii
Bembidion confusum
Bembidion coxendix
Bembidion paraenulum
Bembidion persimile
Bembidion balli
Bembidion chalceum
Bembidion louisella
Bembidion rothfelsi
Bembidion basicorne
Bembidion gebleri
Bembidion hastii
Bembidion lonae
Amerizus (Tiruka) sp.
Amerizus wingatei
Bembidion (Hoquedela) sp. 1
Bembidion cf. csikii
Bembidion escherichi
Bembidion lunulatum
Bembidion poculare
Bembidion quadrimaculatum
Bembidion bifossulatum
Bembidion americanum
Bembidion inaequale
Bembidion levettei carrianum
Bembidion mimekara
Bembidion planum
Serranillus
Geocharidius
Typhlocharis armatus
Sloaneana lamingtonensis
Merizodus sp. nr. catapileanus
Oopterus helmsi
Asaphidion alaskanum
Asaphidion curtum
Asaphidion yukonense
Sinechostictus (Pseudolimnaeum) sp. 3
Sinechostictus elongatus
Sinechostictus solarii
Bembidion rufotinctum
Bembidion honestum
Bembidion integrum
Bembidion kuprianovii
Bembidion breve
Bembidion simplex
Bembidion subfusum DNA3977 DeNovo
Bembidion subfusum DNA3977 FarRef
Bembidion subfusum DNA3977 NearRef
Thalassotrechus barbarae
Diplochaetus planatus
Pogonus chalceus
Sirdenus grayii
Bembidion constricticolle
Bembidion cf. cognatum
Bembidion affine
Bembidion versicolor
Bembidion californicum
Bembidion semistriatum
Bembidion concolor
Bembidion humboldtense
Bembidion luridicorne
Bembidion properans
Bembidion interventor
Bembidion levigatum
Bembidion elizabethae
Bembidion frontale
Bembidion fortestriatum
Bembidion pseudocautum
Bembidion anchonoderum
Bembidion parviceps
Bembidion musae DNA3239 DeNovo
Bembidion musae DNA3239 NearRef
Bembidion musae DNA3239 FarRef
Bembidion carinula
Bembidion lapponicum DNA1604 PCR
Bembidion lapponicum DNA3974 DeNovo
Bembidion lapponicum DNA3974 FarRef
Bembidion lapponicum DNA3974 NearRef
Bembidarenas DNA3983 FarRef
Bembidarenas reicheellum #1
Bembidarenas DNA3983 VeryNearRef
Bembidarenas DNA3983 NearRef
Bembidarenas reicheellum #2 DNA1450 PCR
Bembidarenas DNA3983 DeNovo
Lionepha disjuncta
Lionepha erasa
Lionepha osculans
Lionepha chintimini DNA4002 FarRef
Lionepha chintimini DNA4059 PCR
Lionepha chintimini DNA4002 DeNovo
Lionepha chintimini DNA4002 NearRef
Bembidion variegatum
Bembidion dentellum
Bembidion immaturum
Bembidion "Inuvik" DNA3285 DeNovo
Bembidion "Inuvik" DNA3984 DeNovo
Bembidion "Inuvik" DNA3984 PCR
Bembidion "Inuvik" DNA3285 NearRef
Bembidion "Inuvik" DNA3285 FarRef
Bembidion "Inuvik" DNA3984 FarRef
Bembidion "Inuvik" DNA3984 NearRef
Bembidion solieri
Bembidion sp. nr. ugartei
Bembidion aratum
Bembidion rapidum
Bembidion scintillans
Bembidion approximatum
Bembidion flohri
Bembidion dorsale
Bembidion variolosum
Bembidion varium
Bembidion castor
Bembidion iridescens
Bembidion trechiforme
Bembidion horni
Bembidion festivum
Bembidion mundum
Bembidion orion DNA3079 PCR
Bembidion orion DNA2831 DeNovo
Bembidion orion DNA3079 DeNovo
Bembidion orion DNA2831 FarRef
Bembidion orion DNA2831 NearRef
Bembidion orion DNA3079 FarRef
Bembidion orion DNA3079 NearRef
Bembidion sexfoveatum
Bembidion rugosellum
Bembidion melanopodum
Bembidion rogersi
Bembidion mendocinum
Bembidion caoduroi
Bembidion (Chilioperyphus) sp. "Cal"
Bembidion orregoi
Bembidion sp. nr. chilense
Bembidion rufoplagiatum
Bembidion mandibulare
Bembidion "Arica" DNA3242 DeNovo
Bembidion Arica DNA3242 FarRef
Bembidion Arica DNA3242 NearRef
Bembidion liangi
Bembidion (Hypsipezum) sp. 1
Bembidion bimaculatum
Bembidion commotum
Bembidion lenae
Bembidion mckinleyi carneum
Bembidion sejunctum
Bembidion tetracolum
Bembidion tigrinum
Bembidion laetum
Bembidion punctulatum
Bembidion astrabadense
Bembidion geniculatum
Bembidion callosum
Bembidion genei illigeri
Bembidion grapii
Bembidion platynoides
Bembidion plagiatum
Bembidion "Desert Spotted" DNA2786 PCR
Bembidion cf. "Desert Spotted" DNA3978 DeNovo
Bembidion cf. "Desert Spotted" DNA3978 FarRef
Bembidion cf. "Desert Spotted" DNA3978 NearRef
Bembidion laterale
Bembidion ruficolle
Bembidion lachnophoroides DNA3022 DeNovo
Bembidion lachnophoroides DNA3022 FarRef
Bembidion lachnophoroides DNA3022 NearRef
Bembidion lugubre
Bembidion mexicanum
Bembidion perspicuum
Bembidion transversale
Bembidion sp. nr. transversale DNA3021 DeNovo
Bembidion sp. nr. transversale DNA3205 DeNovo
Bembidion sp. nr. transversale DNA3021 NearRef
Bembidion sp. nr. transversale DNA3021 FarRef
Bembidion sp. nr. transversale DNA3205 NearRef
Bembidion sp. nr. transversale DNA3205 FarRef
ML Bootstrap
de novo, near ref, and far ref assemblies separate
28S

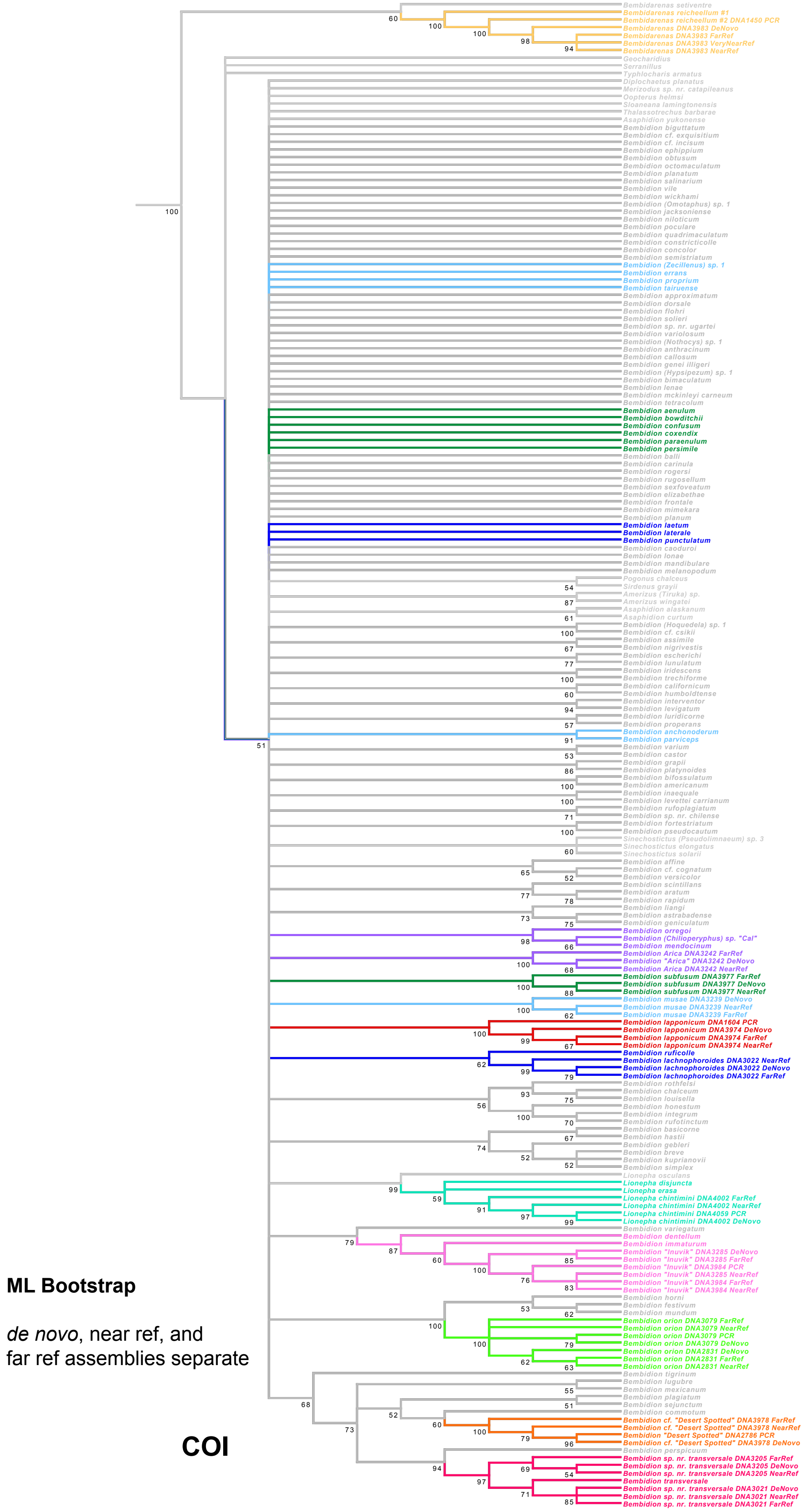

Bembidarenas setiventre
Bembidarenas reicheellum #1
Bembidarenas reicheellum #2 DNA1450 PCR
Bembidarenas DNA3983 DeNovo
Bembidarenas DNA3983 FarRef
Bembidarenas DNA3983 VeryNearRef
Bembidarenas DNA3983 NearRef
Geocharidius
Serranillus
Typhlocharis armatus
Diplochaetus planatus
Merizodus sp. nr. catapileanus
Oopterus helmsi
Sloaneana lamingtonensis
Thalassotrechus barbarae
Asaphidion yukonense
Bembidion biguttatum
Bembidion cf. exquisitum
Bembidion cf. incisum
Bembidion ephippium
Bembidion obtusum
Bembidion octomaculatum
Bembidion planatum
Bembidion salinarium
Bembidion vile
Bembidion wickhami
Bembidion (Omotaphus) sp. 1
Bembidion jacksoniense
Bembidion niloticum
Bembidion poculare
Bembidion quadrimaculatum
Bembidion constricticolle
Bembidion concolor
Bembidion semistriatum
Bembidion (Zecillenus) sp. 1
Bembidion errans
Bembidion proprium
Bembidion tairuense
Bembidion approximatum
Bembidion dorsale
Bembidion flohri
Bembidion solieri
Bembidion sp. nr. ugartei
Bembidion variolosum
Bembidion (Nothocys) sp. 1
Bembidion anthracinum
Bembidion callosum
Bembidion genei illigeri
Bembidion (Hypsipezum) sp. 1
Bembidion bimaculatum
Bembidion lenae
Bembidion mckinleyi carneum
Bembidion tetracolum
Bembidion aenulum
Bembidion bowditchii
Bembidion confusum
Bembidion coxendix
Bembidion paraenulum
Bembidion persimile
Bembidion balli
Bembidion carinula
Bembidion rogersi
Bembidion rugosellum
Bembidion sexfoveatum
Bembidion elizabethae
Bembidion frontale
Bembidion mimekara
Bembidion planum
Bembidion laetum
Bembidion laterale
Bembidion punctulatum
Bembidion caoduroi
Bembidion lonae
Bembidion mandibulare
Bembidion melanopodum
Pogonus chalceus
Sirdenus grayii
Amerizus (Tiruka) sp.
Amerizus wingatei
Asaphidion alaskanum
Asaphidion curtum
Bembidion (Hoquedela) sp. 1
Bembidion cf. csikii
Bembidion assimile
Bembidion nigrivestis
Bembidion escherichi
Bembidion lunulatum
Bembidion iridescens
Bembidion trechiforme
Bembidion californicum
Bembidion humboldtense
Bembidion interventor
Bembidion levigatum
Bembidion luridicorne
Bembidion properans
Bembidion anchonoderum
Bembidion parviceps
Bembidion varium
Bembidion castor
Bembidion grapii
Bembidion platynoides
Bembidion bifossulatum
Bembidion americanum
Bembidion inaequale
Bembidion levettei carrianum
Bembidion rufoplagiatum
Bembidion sp. nr. chilense
Bembidion fortestriatum
Bembidion pseudocautum
Sinechostictus (Pseudolimnaeum) sp. 3
Sinechostictus elongatus
Sinechostictus solarii
Bembidion affine
Bembidion cf. cognatum
Bembidion versicolor
Bembidion scintillans
Bembidion aratum
Bembidion rapidum
Bembidion liangi
Bembidion astrabadense
Bembidion geniculatum
Bembidion orregoi
Bembidion (Chilioperyphus) sp. "Cal"
Bembidion mendocinum
Bembidion Arica DNA3242 FarRef
Bembidion "Arica" DNA3242 DeNovo
Bembidion Arica DNA3242 NearRef
Bembidion subfusum DNA3977 FarRef
Bembidion subfusum DNA3977 DeNovo
Bembidion subfusum DNA3977 NearRef
Bembidion musae DNA3239 DeNovo
Bembidion musae DNA3239 NearRef
Bembidion musae DNA3239 FarRef
Bembidion lapponicum DNA1604 PCR
Bembidion lapponicum DNA3974 DeNovo
Bembidion lapponicum DNA3974 FarRef
Bembidion lapponicum DNA3974 NearRef
Bembidion ruficolle
Bembidion lachnophoroides DNA3022 NearRef
Bembidion lachnophoroides DNA3022 DeNovo
Bembidion lachnophoroides DNA3022 FarRef
Bembidion rothfelsi
Bembidion chalceum
Bembidion louisella
Bembidion honestum
Bembidion integrum
Bembidion rufotinctum
Bembidion basicorne
Bembidion hastii
Bembidion gebleri
Bembidion breve
Bembidion kuprianovii
Bembidion simplex
Lionepha osculans
Lionepha disjuncta
Lionepha erasa
Lionepha chintimini DNA4002 FarRef
Lionepha chintimini DNA4002 NearRef
Lionepha chintimini DNA4059 PCR
Lionepha chintimini DNA4002 DeNovo
Bembidion variegatum
Bembidion dentellum
Bembidion immaturum
Bembidion "Inuvik" DNA3285 DeNovo
Bembidion "Inuvik" DNA3285 FarRef
Bembidion "Inuvik" DNA3984 PCR
Bembidion "Inuvik" DNA3285 NearRef
Bembidion "Inuvik" DNA3984 FarRef
Bembidion "Inuvik" DNA3984 NearRef
Bembidion horni
Bembidion festivum
Bembidion mundum
Bembidion orion DNA3079 FarRef
Bembidion orion DNA3079 NearRef
Bembidion orion DNA3079 PCR
Bembidion orion DNA3079 DeNovo
Bembidion orion DNA2831 DeNovo
Bembidion orion DNA2831 FarRef
Bembidion orion DNA2831 NearRef
Bembidion tigrinum
Bembidion lugubre
Bembidion mexicanum
Bembidion plagiatum
Bembidion sejunctum
Bembidion commotum
Bembidion cf. "Desert Spotted" DNA3978 FarRef
Bembidion cf. "Desert Spotted" DNA3978 NearRef
Bembidion "Desert Spotted" DNA2786 PCR
Bembidion cf. "Desert Spotted" DNA3978 DeNovo
Bembidion perspicuum
Bembidion sp. nr. transversale DNA3205 FarRef
Bembidion sp. nr. transversale DNA3205 DeNovo
Bembidion sp. nr. transversale DNA3205 NearRef
Bembidion transversale
Bembidion sp. nr. transversale DNA3021 DeNovo
Bembidion sp. nr. transversale DNA3021 NearRef
Bembidion sp. nr. transversale DNA3021 FarRef
ML Bootstrap
de novo, near ref, and
far ref assemblies separate
COI

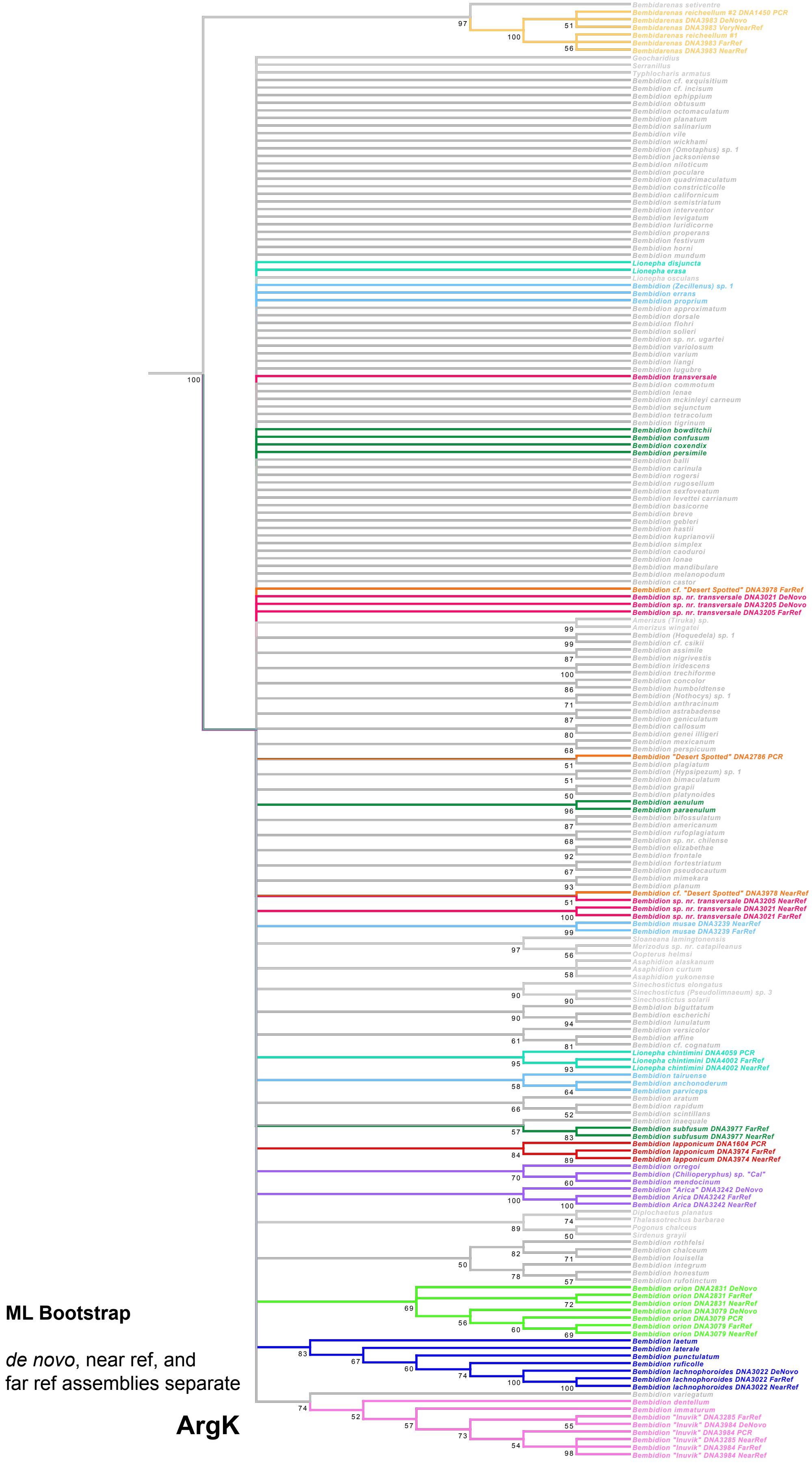
Bembidarenas setiventre
Bembidarenas reicheellum #2 DNA1450 PCR
Bembidarenas DNA3983 DeNovo
Bembidarenas DNA3983 VeryNearRef
Bembidarenas reicheellum #1
Bembidarenas DNA3983 FarRef
Bembidarenas DNA3983 NearRef
Geocharidius
Serranillus
Typhlocharis armatus
Bembidion cf. exquisitium
Bembidion cf. incisum
Bembidion ephippium
Bembidion obtusum
Bembidion octomaculatum
Bembidion planatum
Bembidion salinarium
Bembidion vile
Bembidion wickhami
Bembidion (Omotaphus) sp. 1
Bembidion jacksoniense
Bembidion niloticum
Bembidion poculare
Bembidion quadrimaculatum
Bembidion constricticolle
Bembidion californicum
Bembidion semistriatum
Bembidion interventor
Bembidion levigatum
Bembidion luridicorne
Bembidion properans
Bembidion festivum
Bembidion horni
Bembidion mundum
Lionepha disjuncta
Lionepha erasa
Lionepha osculans
Bembidion (Zecillenus) sp. 1
Bembidion errans
Bembidion proprium
Bembidion approximatum
Bembidion dorsale
Bembidion flohri
Bembidion solieri
Bembidion sp. nr. ugartei
Bembidion variolosum
Bembidion varium
Bembidion liangi
Bembidion lugubre
Bembidion transversale
Bembidion commotum
Bembidion lenae
Bembidion mckinleyi carneum
Bembidion sejunctum
Bembidion tetracolum
Bembidion tigrinum
Bembidion bowditchii
Bembidion confusum
Bembidion coxendix
Bembidion persimile
Bembidion balli
Bembidion carinula
Bembidion rogersi
Bembidion rugosellum
Bembidion sexfoveatum
Bembidion levettei carrianum
Bembidion basicorne
Bembidion breve
Bembidion gebleri
Bembidion hastii
Bembidion kuprianovii
Bembidion simplex
Bembidion caoduroi
Bembidion lonae
Bembidion mandibulare
Bembidion melanopodum
Bembidion castor
Bembidion cf. "Desert Spotted" DNA3978 FarRef
Bembidion sp. nr. transversale DNA3021 DeNovo
Bembidion sp. nr. transversale DNA3205 DeNovo
Bembidion sp. nr. transversale DNA3205 FarRef
Amerizus (Tiruka) sp.
Amerizus wingatei
Bembidion (Hoquedela) sp. 1
Bembidion cf. csikii
Bembidion assimile
Bembidion nigrivestis
Bembidion iridescens
Bembidion trechiforme
Bembidion concolor
Bembidion humboldtense
Bembidion (Nothocys) sp. 1
Bembidion anthracinum
Bembidion astrabadense
Bembidion geniculatum
Bembidion callosum
Bembidion genei illigeri
Bembidion mexicanum
Bembidion perspicuum
Bembidion "Desert Spotted" DNA2786 PCR
Bembidion plagiatum
Bembidion (Hypsipezum) sp. 1
Bembidion bimaculatum
Bembidion grapii
Bembidion platynoides
Bembidion aenulum
Bembidion paraenulum
Bembidion bifossulatum
Bembidion americanum
Bembidion rufoplagiatum
Bembidion sp. nr. chilense
Bembidion elizabethae
Bembidion frontale
Bembidion fortestriatum
Bembidion pseudocautum
Bembidion mimekara
Bembidion planum
Bembidion cf. "Desert Spotted" DNA3978 NearRef
Bembidion sp. nr. transversale DNA3205 NearRef
Bembidion sp. nr. transversale DNA3021 NearRef
Bembidion sp. nr. transversale DNA3021 FarRef
Bembidion musae DNA3239 NearRef
Bembidion musae DNA3239 FarRef
Sloaneana lamingtonensis
Merizodus sp. nr. catapileanus
Oopterus helmsi
Asaphidion alaskanum
Asaphidion curtum
Asaphidion yukonense
Sinechostictus elongatus
Sinechostictus (Pseudolimnaeum) sp. 3
Sinechostictus solarii
Bembidion biguttatum
Bembidion escherichi
Bembidion lunulatum
Bembidion versicolor
Bembidion affine
Bembidion cf. cognatum
Lionepha chintimini DNA4059 PCR
Lionepha chintimini DNA4002 FarRef
Lionepha chintimini DNA4002 NearRef
Bembidion tairuense
Bembidion anchonoderum
Bembidion parviceps
Bembidion aratum
Bembidion rapidum
Bembidion scintillans
Bembidion inaequale
Bembidion subfusum DNA3977 FarRef
Bembidion subfusum DNA3977 NearRef
Bembidion lapponicum DNA1604 PCR
Bembidion lapponicum DNA3974 FarRef
Bembidion lapponicum DNA3974 NearRef
Bembidion orregoi
Bembidion (Chilioperyphus) sp. "Cal"
Bembidion mendocinum
Bembidion "Arica" DNA3242 DeNovo
Bembidion Arica DNA3242 FarRef
Bembidion Arica DNA3242 NearRef
Diplochaetus planatus
Thalassotrechus barbarae
Pogonus chalceus
Sirdenus grayii
Bembidion rothfelsi
Bembidion chalceum
Bembidion louisella
Bembidion integrum
Bembidion honestum
Bembidion rufotinctum
Bembidion orion DNA2831 DeNovo
Bembidion orion DNA2831 FarRef
Bembidion orion DNA2831 NearRef
Bembidion orion DNA3079 DeNovo
Bembidion orion DNA3079 PCR
Bembidion orion DNA3079 FarRef
Bembidion orion DNA3079 NearRef
Bembidion laetum
Bembidion laterale
Bembidion punctulatum
Bembidion ruficolle
Bembidion lachnophoroides DNA3022 DeNovo
Bembidion lachnophoroides DNA3022 FarRef
Bembidion lachnophoroides DNA3022 NearRef
Bembidion variegatum
Bembidion dentellum
Bembidion immaturum
Bembidion "Inuvik" DNA3285 FarRef
Bembidion "Inuvik" DNA3984 DeNovo
Bembidion "Inuvik" DNA3984 PCR
Bembidion "Inuvik" DNA3285 NearRef
Bembidion "Inuvik" DNA3984 FarRef
Bembidion "Inuvik" DNA3984 NearRef
ML Bootstrap
de novo, near ref, and
far ref assemblies separate
ArgK

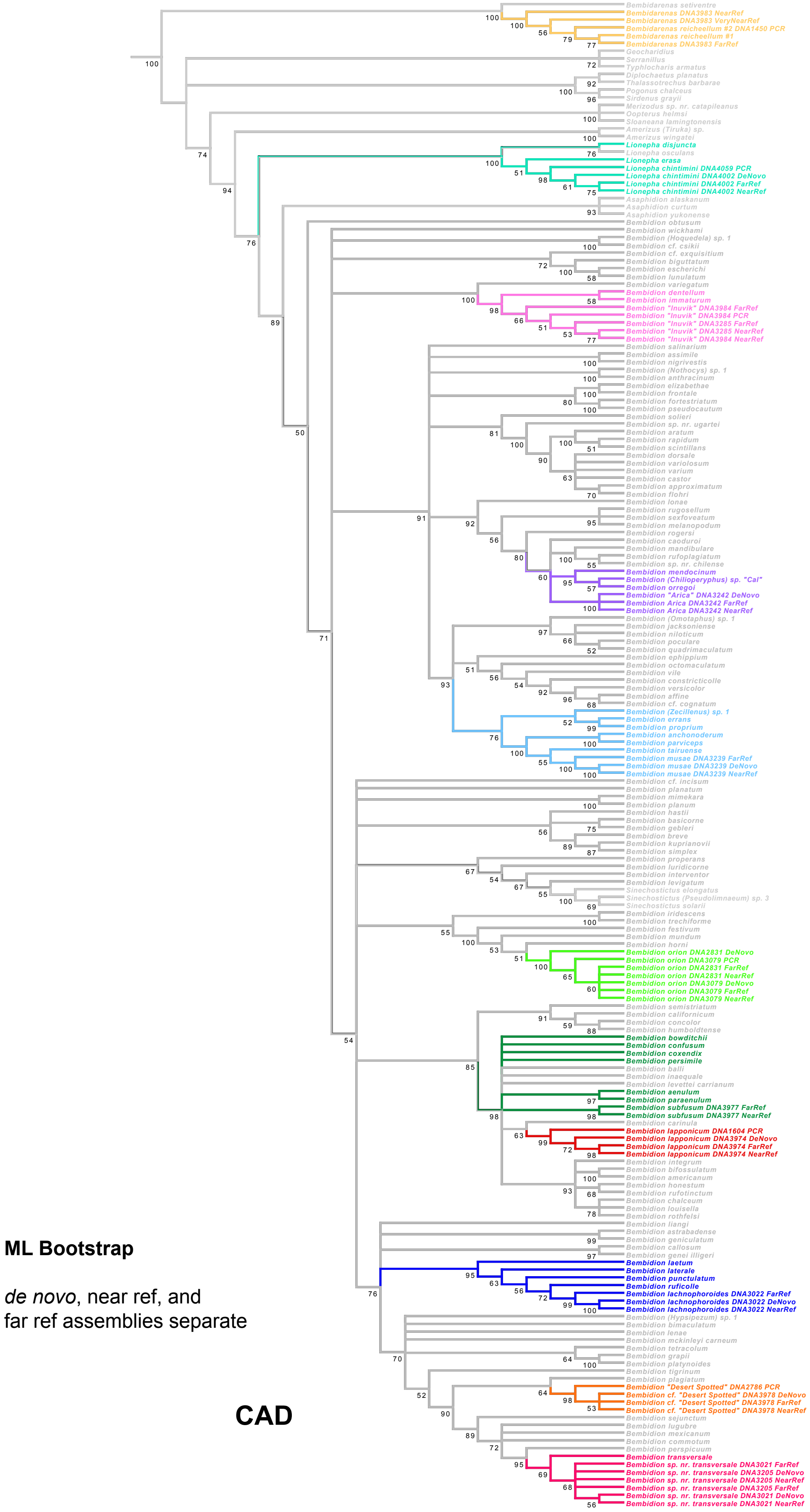

Bembidarenas setiventre
Bembidarenas DNA3983 NearRef
Bembidarenas DNA3983 VeryNearRef
Bembidarenas reicheellum #2 DNA1450 PCR
Bembidarenas reicheellum #1
Bembidarenas DNA3983 FarRef
Geocharidius
Serranillus
Typhlocharis armatus
Diplochaetus planatus
Thalassotrechus barbarae
Pogonus chalceus
Sirdenus grayii
Merizodus sp. nr. catapileanus
Oopterus helmsi
Sloaneana tamingtonensis
Amerizus (Tiruka) sp.
Amerizus wingatei
Lionepha disjuncta
Lionepha osculans
Lionepha erasa
Lionepha chintimini DNA4059 PCR
Lionepha chintimini DNA4002 DeNovo
Lionepha chintimini DNA4002 FarRef
Lionepha chintimini DNA4002 NearRef
Asaphidion alaskanum
Asaphidion curtum
Asaphidion yukonense
Bembidion obtusum
Bembidion wickhami
Bembidion (Hoquedela) sp. 1
Bembidion cf. csikii
Bembidion cf. exquisitium
Bembidion biguttatum
Bembidion escherichi
Bembidion lunulatum
Bembidion variegatum
Bembidion dentellum
Bembidion immaturum
Bembidion "Inuvik" DNA3984 FarRef
Bembidion "Inuvik" DNA3984 PCR
Bembidion "Inuvik" DNA3285 FarRef
Bembidion "Inuvik" DNA3285 NearRef
Bembidion "Inuvik" DNA3984 NearRef
Bembidion salinarium
Bembidion assimile
Bembidion nigrivestis
Bembidion (Nothocys) sp. 1
Bembidion anthracinum
Bembidion elizabethae
Bembidion frontale
Bembidion fortestriatum
Bembidion pseudocautum
Bembidion solieri
Bembidion sp. nr. ugartei
Bembidion aratum
Bembidion rapidum
Bembidion scintillans
Bembidion dorsale
Bembidion variolosum
Bembidion varium
Bembidion castor
Bembidion approximatum
Bembidion flohri
Bembidion lonae
Bembidion rugosellum
Bembidion sexfoveatum
Bembidion melanopodum
Bembidion rogersi
Bembidion caoduroi
Bembidion mandibulare
Bembidion rufoplagiatum
Bembidion sp. nr. chilense
Bembidion mendocinum
Bembidion (Chilioperyphus) sp. "Cal"
Bembidion orregoi
Bembidion "Arica" DNA3242 DeNovo
Bembidion Arica DNA3242 FarRef
Bembidion Arica DNA3242 NearRef
Bembidion (Omotaphus) sp. 1
Bembidion jacksoniense
Bembidion niloticum
Bembidion poculare
Bembidion quadrimaculatum
Bembidion ephippium
Bembidion octomaculatum
Bembidion vile
Bembidion constricticolle
Bembidion versicolor
Bembidion affine
Bembidion cf. cognatum
Bembidion (Zecillenus) sp. 1
Bembidion errans
Bembidion proprium
Bembidion anchonoderum
Bembidion parviceps
Bembidion tairuense
Bembidion musae DNA3239 FarRef
Bembidion musae DNA3239 DeNovo
Bembidion musae DNA3239 NearRef
Bembidion cf. incisum
Bembidion planatum
Bembidion mimekara
Bembidion planum
Bembidion hastii
Bembidion basicorne
Bembidion gebleri
Bembidion breve
Bembidion kuprianovii
Bembidion simplex
Bembidion properans
Bembidion luridicorne
Bembidion interventor
Bembidion levigatum
Sinechostictus elongatus
Sinechostictus (Pseudolimnaeum) sp. 3
Sinechostictus solarii
Bembidion iridescens
Bembidion trechiforme
Bembidion festivum
Bembidion mundum
Bembidion horni
Bembidion orion DNA2831 DeNovo
Bembidion orion DNA3079 PCR
Bembidion orion DNA2831 FarRef
Bembidion orion DNA2831 NearRef
Bembidion orion DNA3079 DeNovo
Bembidion orion DNA3079 FarRef
Bembidion orion DNA3079 NearRef
Bembidion semistriatum
Bembidion californicum
Bembidion concolor
Bembidion humboldtense
Bembidion bowditchii
Bembidion confusum
Bembidion coxendix
Bembidion persimile
Bembidion balli
Bembidion inaequale
Bembidion levettei carrianum
Bembidion aenulum
Bembidion paraenulum
Bembidion subfusum DNA3977 FarRef
Bembidion subfusum DNA3977 NearRef
Bembidion carinula
Bembidion lapponicum DNA1604 PCR
Bembidion lapponicum DNA3974 DeNovo
Bembidion lapponicum DNA3974 FarRef
Bembidion lapponicum DNA3974 NearRef
Bembidion integrum
Bembidion bifossulatum
Bembidion americanum
Bembidion honestum
Bembidion rufotinctum
Bembidion chalceum
Bembidion louisella
Bembidion rothfelsi
Bembidion liangi
Bembidion astrabadense
Bembidion geniculatum
Bembidion callosum
Bembidion genei illigeri
Bembidion laetum
Bembidion laterale
Bembidion punctulatum
Bembidion ruficolle
Bembidion lachnophoroides DNA3022 FarRef
Bembidion lachnophoroides DNA3022 DeNovo
Bembidion lachnophoroides DNA3022 NearRef
Bembidion (Hypsipezum) sp. 1
Bembidion bimaculatum
Bembidion lenae
Bembidion mckinleyi carneum
Bembidion tetracolum
Bembidion grapii
Bembidion platynoides
Bembidion tigrinum
Bembidion plagiatum
Bembidion "Desert Spotted" DNA2786 PCR
Bembidion cf. "Desert Spotted" DNA3978 DeNovo
Bembidion cf. "Desert Spotted" DNA3978 FarRef
Bembidion cf. "Desert Spotted" DNA3978 NearRef
Bembidion sejunctum
Bembidion lugubre
Bembidion mexicanum
Bembidion commotum
Bembidion perspicuum
Bembidion transversale
Bembidion sp. nr. transversale DNA3021 FarRef
Bembidion sp. nr. transversale DNA3205 DeNovo
Bembidion sp. nr. transversale DNA3205 NearRef
Bembidion sp. nr. transversale DNA3205 FarRef
Bembidion sp. nr. transversale DNA3021 DeNovo
Bembidion sp. nr. transversale DNA3021 NearRef
ML Bootstrap
de novo, near ref, and
far ref assemblies separate
CAD

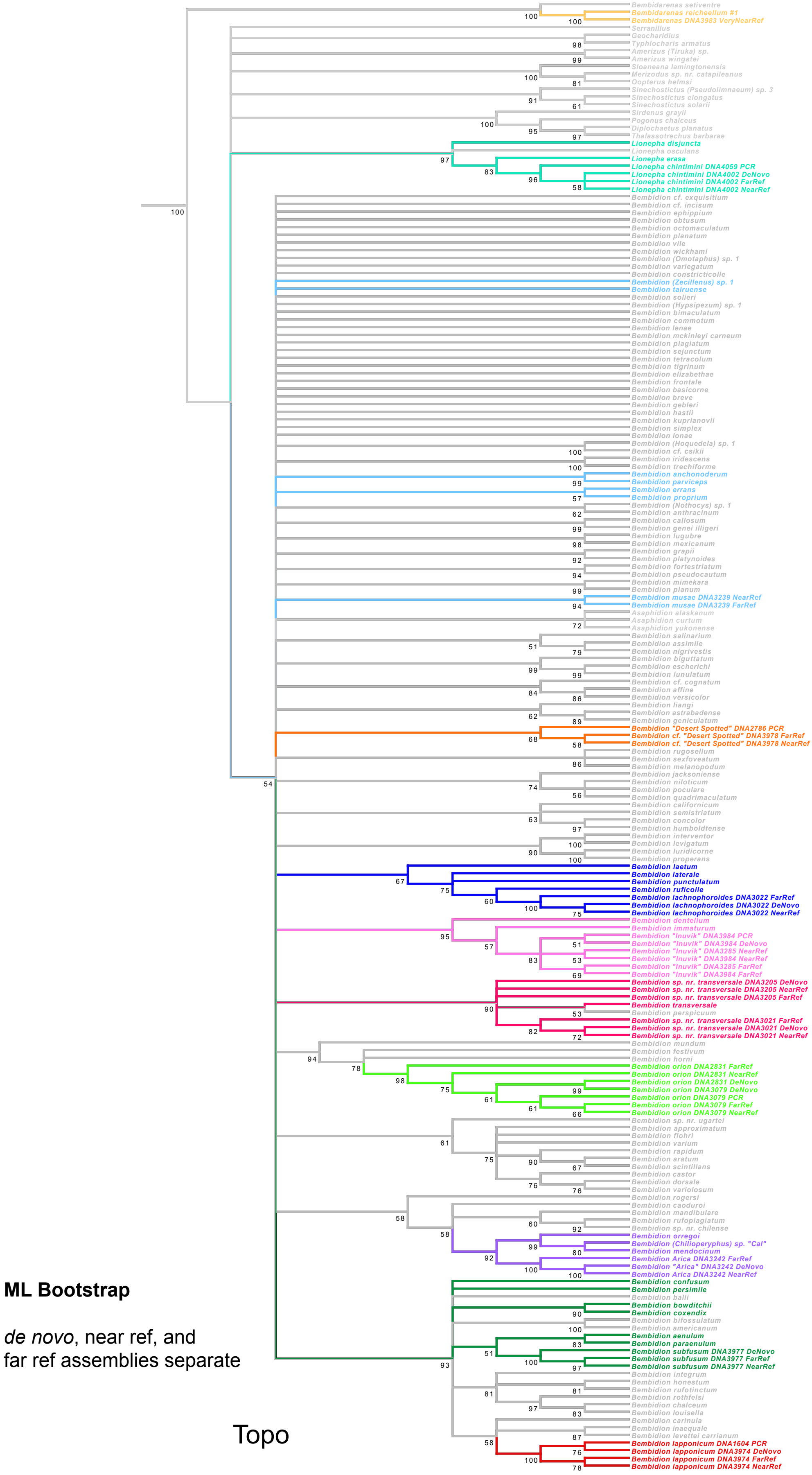
Bembidarenas setiventre
Bembidarenas reicheellum #1
Bembidarenas DNA3983 VeryNearRef
Serranillus
Geocharidius
Typhlocharis armatus
Amerizus (Tiruka) sp.
Amerizus wingatei
Sloaneana lamingtonensis
Merizodus sp. nr. catapileanus
Oopterus helmsi
Sinechostictus (Pseudolimnaeum) sp. 3
Sinechostictus elongatus
Sinechostictus solarii
Sirdenus grayii
Pogonus chalceus
Diplochaetus planatus
Thalassotrechus barbarae
Lionepha disjuncta
Lionepha osculans
Lionepha erasa
Lionepha chintimini DNA4059 PCR
Lionepha chintimini DNA4002 DeNovo
Lionepha chintimini DNA4002 FarRef
Lionepha chintimini DNA4002 NearRef
Bembidion cf. exquisitium
Bembidion cf. incisum
Bembidion ephippium
Bembidion obtusum
Bembidion octomaculatum
Bembidion planatum
Bembidion vile
Bembidion wickhami
Bembidion (Omotaphus) sp. 1
Bembidion variegatum
Bembidion constricticolle
Bembidion (Zecillenus) sp. 1
Bembidion tairuense
Bembidion solieri
Bembidion (Hypsipezum) sp. 1
Bembidion bimaculatum
Bembidion commotum
Bembidion lenae
Bembidion mckinleyi carneum
Bembidion plagiatum
Bembidion sejunctum
Bembidion tetracolum
Bembidion tigrinum
Bembidion elizabethae
Bembidion frontale
Bembidion basicorne
Bembidion breve
Bembidion gebleri
Bembidion hastii
Bembidion kuprianovii
Bembidion simplex
Bembidion lonae
Bembidion (Hoquedela) sp. 1
Bembidion cf. csikii
Bembidion iridescens
Bembidion trechiforme
Bembidion anchonoderum
Bembidion parviceps
Bembidion errans
Bembidion proprium
Bembidion (Nothocys) sp. 1
Bembidion anthracinum
Bembidion callosum
Bembidion genei illigeri
Bembidion lugubre
Bembidion mexicanum
Bembidion grapii
Bembidion platynoides
Bembidion fortestriatum
Bembidion pseudocautum
Bembidion mimekara
Bembidion planum
Bembidion musae DNA3239 NearRef
Bembidion musae DNA3239 FarRef
Asaphidion alaskanum
Asaphidion curtum
Asaphidion yukonense
Bembidion salinarium
Bembidion assimile
Bembidion nigrivestis
Bembidion biguttatum
Bembidion escherichi
Bembidion lunulatum
Bembidion cf. cognatum
Bembidion affine
Bembidion versicolor
Bembidion liangi
Bembidion astrabadense
Bembidion geniculatum
Bembidion "Desert Spotted" DNA2786 PCR
Bembidion cf. "Desert Spotted" DNA3978 FarRef
Bembidion cf. "Desert Spotted" DNA3978 NearRef
Bembidion rugosellum
Bembidion sexfoveatum
Bembidion melanopodum
Bembidion jacksoniense
Bembidion niloticum
Bembidion poculare
Bembidion quadrimaculatum
Bembidion californicum
Bembidion semistriatum
Bembidion concolor
Bembidion humboldtense
Bembidion interventor
Bembidion levigatum
Bembidion luridicorne
Bembidion properans
Bembidion laetum
Bembidion laterale
Bembidion punctulatum
Bembidion ruficolle
Bembidion lachnophoroides DNA3022 FarRef
Bembidion lachnophoroides DNA3022 DeNovo
Bembidion lachnophoroides DNA3022 NearRef
Bembidion dentellum
Bembidion immaturum
Bembidion "Inuvik" DNA3984 PCR
Bembidion "Inuvik" DNA3984 DeNovo
Bembidion "Inuvik" DNA3285 NearRef
Bembidion "Inuvik" DNA3984 NearRef
Bembidion "Inuvik" DNA3285 FarRef
Bembidion "Inuvik" DNA3984 FarRef
Bembidion sp. nr. transversale DNA3205 DeNovo
Bembidion sp. nr. transversale DNA3205 NearRef
Bembidion sp. nr. transversale DNA3205 FarRef
Bembidion transversale
Bembidion perspicuum
Bembidion sp. nr. transversale DNA3021 FarRef
Bembidion sp. nr. transversale DNA3021 DeNovo
Bembidion sp. nr. transversale DNA3021 NearRef
Bembidion mundum
Bembidion festivum
Bembidion horni
Bembidion orion DNA2831 FarRef
Bembidion orion DNA2831 NearRef
Bembidion orion DNA2831 DeNovo
Bembidion orion DNA3079 DeNovo
Bembidion orion DNA3079 PCR
Bembidion orion DNA3079 FarRef
Bembidion orion DNA3079 NearRef
Bembidion sp. nr. ugartei
Bembidion approximatum
Bembidion flohri
Bembidion varium
Bembidion rapidum
Bembidion aratum
Bembidion scintillans
Bembidion castor
Bembidion dorsale
Bembidion variolosum
Bembidion rogersi
Bembidion caoduroi
Bembidion mandibulare
Bembidion rufoplagiatum
Bembidion sp. nr. chilense
Bembidion orregoi
Bembidion (Chilioperyphus) sp. "Cal"
Bembidion mendocinum
Bembidion Arica DNA3242 FarRef
Bembidion "Arica" DNA3242 DeNovo
Bembidion Arica DNA3242 NearRef
Bembidion confusum
Bembidion persimile
Bembidion balli
Bembidion bowditchii
Bembidion coxendix
Bembidion bifossulatum
Bembidion americanum
Bembidion aenulum
Bembidion paraenulum
Bembidion subfusum DNA3977 DeNovo
Bembidion subfusum DNA3977 FarRef
Bembidion subfusum DNA3977 NearRef
Bembidion integrum
Bembidion honestum
Bembidion rufotinctum
Bembidion rothfelsi
Bembidion chalceum
Bembidion louisella
Bembidion carinula
Bembidion inaequale
Bembidion levettei carrianum
Bembidion lapponicum DNA1604 PCR
Bembidion lapponicum DNA3974 DeNovo
Bembidion lapponicum DNA3974 FarRef
Bembidion lapponicum DNA3974 NearRef
ML Bootstrap
de novo, near ref, and
far ref assemblies separate
Topo

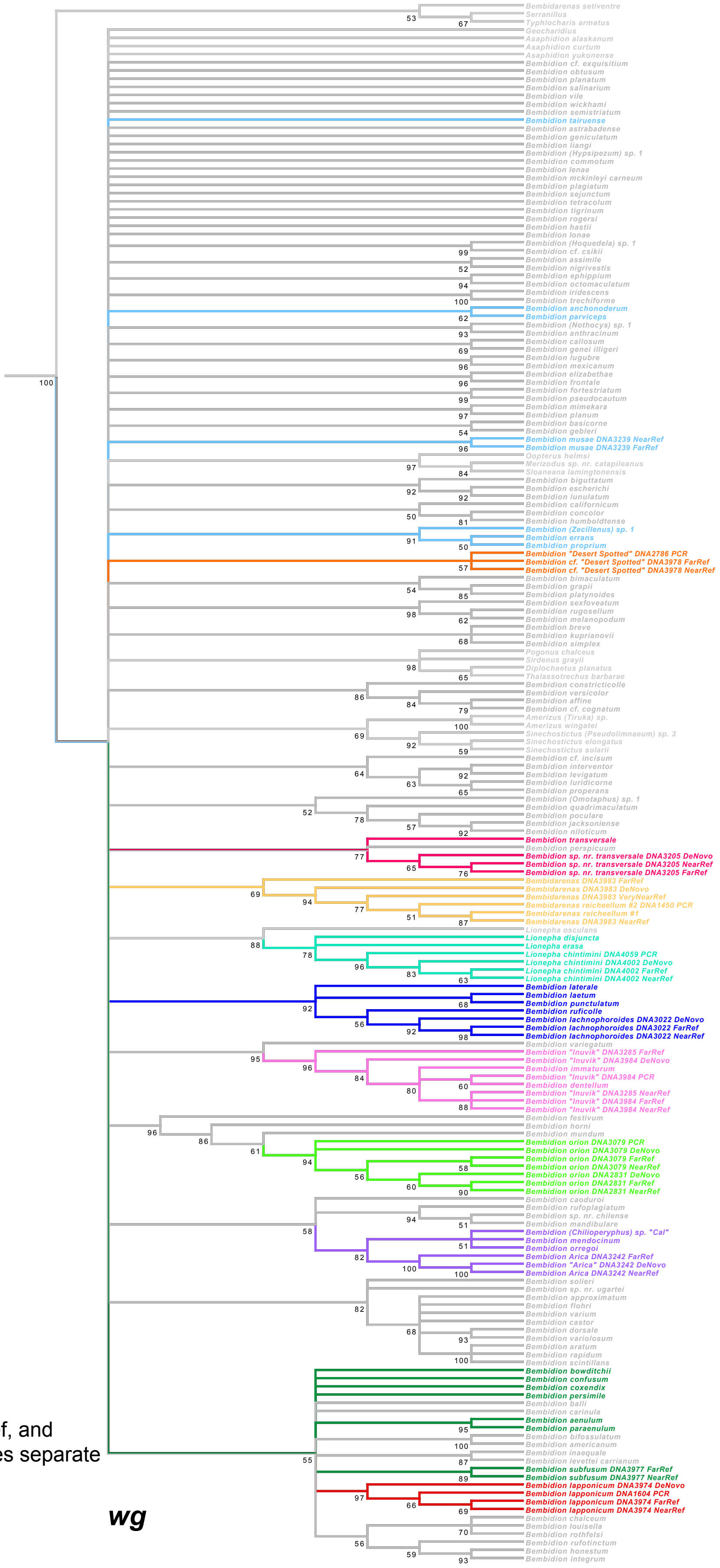

ML Bootstrap

de novo, near ref, and far ref assemblies separate

wg
