# Supplementary material for: Successful Recovery of Nuclear Protein-Coding Genes from Small Insects in Museums Using Illumina Sequencing
Source: PLoS One. 2015 Dec 30;10(12):e0143929. doi: 10.1371/journal.pone.0143929 (PMC4696846; doi:10.1371/journal.pone.0143929)
Supplement: S8 Fig — The placement of the IlluminaMerged sequences is shown relative to their prediction groups. Branches and taxon names of all specimens in the prediction group are indicated with a unique color. (PDF) [file pone.0143929.s008.pdf]

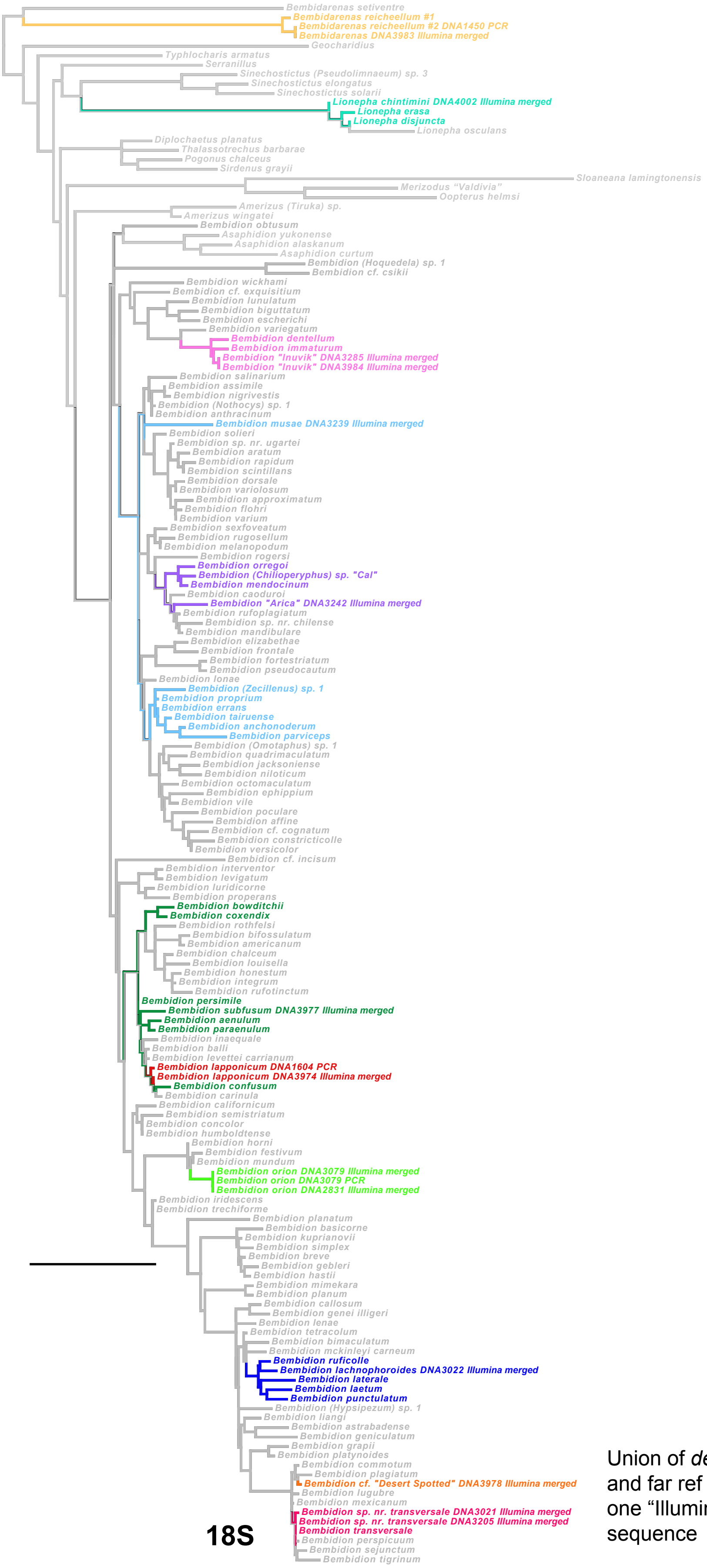

Union of *de novo*, near ref, and far ref assemblies form one "Illumina merged" sequence

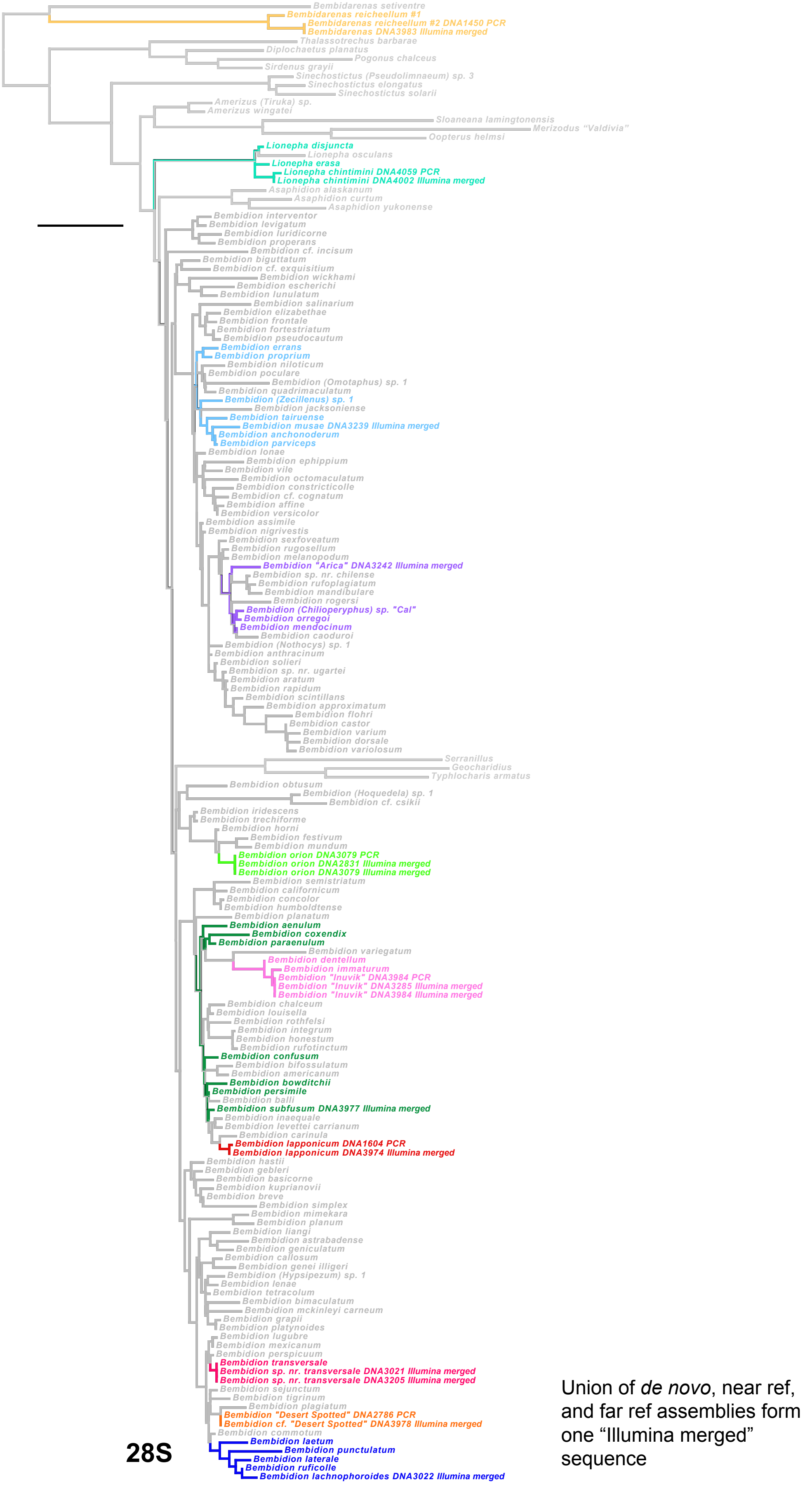
Bembidarenas setiventre
Bembidarenas reicheellum #1
Bembidarenas reicheellum #2 DNA1450 PCR
Bembidarenas DNA3983 Illumina merged
Thalassotrechus barbarae
Diplochaetus planatus
Pogonus chalceus
Sirdenus grayii
Sinechostictus (Pseudolimnaeum) sp. 3
Sinechostictus elongatus
Sinechostictus solarii
Amerizus (Tiruka) sp.
Amerizus wingatei
Sloaneana lamingtonensis
Merizodus "Valdivia"
Oopterus helmsi
Lionepha disjuncta
Lionepha osculans
Lionepha erasa
Lionepha chintimini DNA4059 PCR
Lionepha chintimini DNA4002 Illumina merged
Asaphidion alaskanum
Asaphidion curtum
Asaphidion yukonense
Bembidion interventor
Bembidion levigatum
Bembidion luridicorne
Bembidion properans
Bembidion cf. incisum
Bembidion biguttatum
Bembidion cf. exquisitum
Bembidion wickhami
Bembidion escherichi
Bembidion lunulatum
Bembidion salinarium
Bembidion elizabethae
Bembidion frontale
Bembidion fortestriatum
Bembidion pseudocautum
Bembidion errans
Bembidion proprium
Bembidion niloticum
Bembidion poculare
Bembidion (Omotaphus) sp. 1
Bembidion quadrimaculatum
Bembidion (Zecillenus) sp. 1
Bembidion jacksoniense
Bembidion tairuense
Bembidion musae DNA3239 Illumina merged
Bembidion anchonoderum
Bembidion parviceps
Bembidion lonae
Bembidion ephippium
Bembidion vile
Bembidion octomaculatum
Bembidion constricticolle
Bembidion cf. cognatum
Bembidion affine
Bembidion versicolor
Bembidion assimile
Bembidion nigrivestis
Bembidion sexfoveatum
Bembidion rugosellum
Bembidion melanopodum
Bembidion "Arica" DNA3242 Illumina merged
Bembidion sp. nr. chilense
Bembidion rufoplagiatum
Bembidion mandibulare
Bembidion rogersi
Bembidion (Chilioperyphus) sp. "Cal"
Bembidion orregoi
Bembidion mendocinum
Bembidion caoduroi
Bembidion (Nothocys) sp. 1
Bembidion anthracinum
Bembidion solieri
Bembidion sp. nr. ugartei
Bembidion aratum
Bembidion rapidum
Bembidion scintillans
Bembidion approximatum
Bembidion flohri
Bembidion castor
Bembidion varium
Bembidion dorsale
Bembidion variolosum
Serranillus
Geocharidius
Typhlocharis armatus
Bembidion obtusum
Bembidion (Hoquedela) sp. 1
Bembidion cf. csikii
Bembidion iridescens
Bembidion trechiforme
Bembidion horni
Bembidion festivum
Bembidion mundum
Bembidion orion DNA3079 PCR
Bembidion orion DNA2831 Illumina merged
Bembidion orion DNA3079 Illumina merged
Bembidion semistriatum
Bembidion californicum
Bembidion concolor
Bembidion humboldtense
Bembidion planatum
Bembidion aenulum
Bembidion coxendix
Bembidion paraenulum
Bembidion variegatum
Bembidion dentellum
Bembidion immaturum
Bembidion "Inuvik" DNA3984 PCR
Bembidion "Inuvik" DNA3285 Illumina merged
Bembidion "Inuvik" DNA3984 Illumina merged
Bembidion chalceum
Bembidion louisella
Bembidion rothfelsi
Bembidion integrum
Bembidion honestum
Bembidion rufotinctum
Bembidion confusum
Bembidion bifossulatum
Bembidion americanum
Bembidion bowditchii
Bembidion persimile
Bembidion balli
Bembidion subfusum DNA3977 Illumina merged
Bembidion inaequale
Bembidion levettei carrianum
Bembidion carinula
Bembidion lapponicum DNA1604 PCR
Bembidion lapponicum DNA3974 Illumina merged
Bembidion hastii
Bembidion gebleri
Bembidion basicorne
Bembidion kuprianovii
Bembidion breve
Bembidion simplex
Bembidion mimekara
Bembidion planum
Bembidion liangi
Bembidion astrabadense
Bembidion geniculatum
Bembidion callosum
Bembidion genei illigeri
Bembidion (Hypsipezum) sp. 1
Bembidion lenae
Bembidion tetracolum
Bembidion bimaculatum
Bembidion mckinleyi carneum
Bembidion grapii
Bembidion platynoides
Bembidion lugubre
Bembidion mexicanum
Bembidion perspicuum
Bembidion transversale
Bembidion sp. nr. transversale DNA3021 Illumina merged
Bembidion sp. nr. transversale DNA3205 Illumina merged
Bembidion sejunctum
Bembidion tigrinum
Bembidion plagiatum
Bembidion "Desert Spotted" DNA2786 PCR
Bembidion cf. "Desert Spotted" DNA3978 Illumina merged
Bembidion commotum
Bembidion laetum
Bembidion punctulatum
Bembidion laterale
Bembidion ruficolle
Bembidion lachnophoroides DNA3022 Illumina merged
28S
Union of *de novo*, near ref, and far ref assemblies form one "Illumina merged" sequence

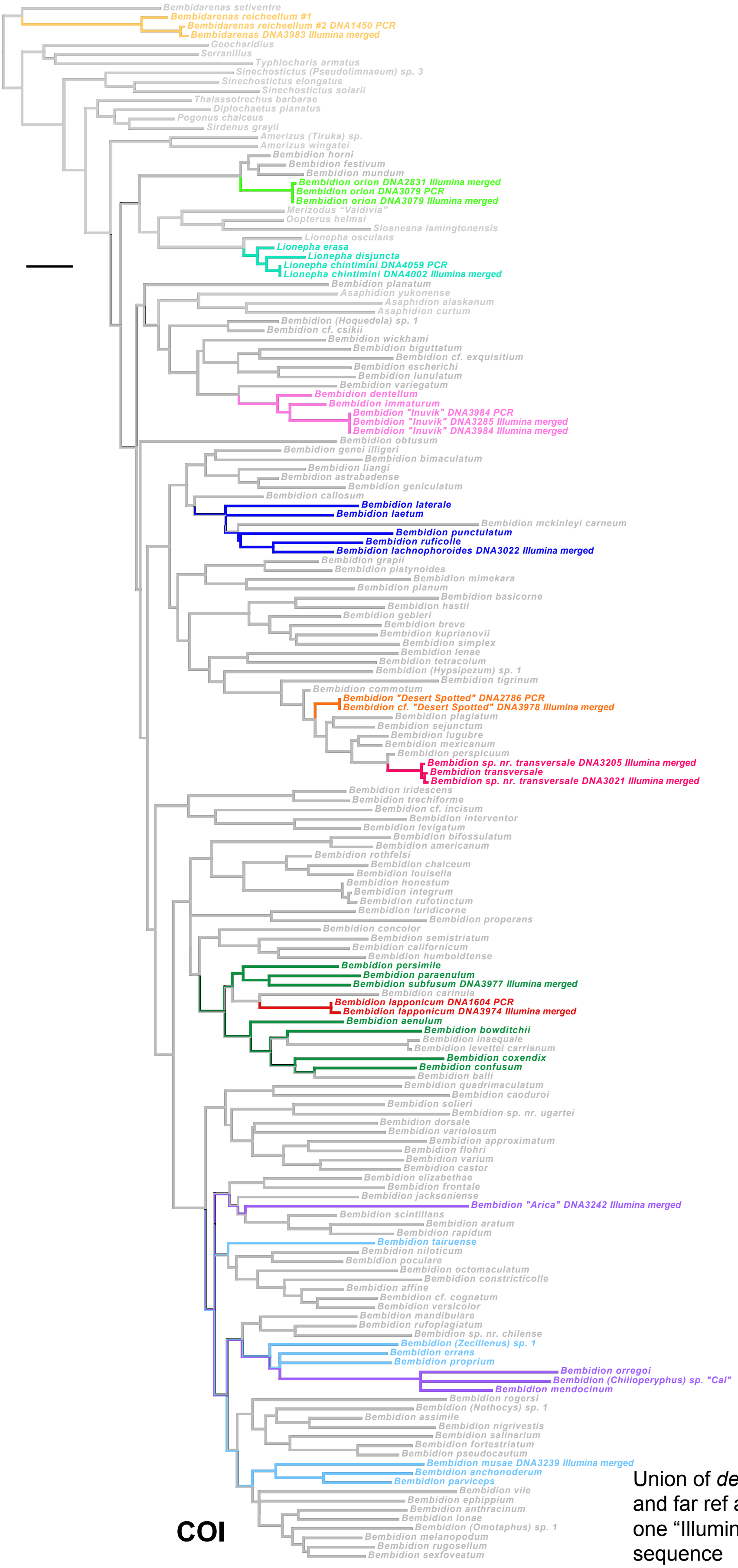

Union of *de novo*, near ref, and far ref assemblies form one "Illumina merged" sequence

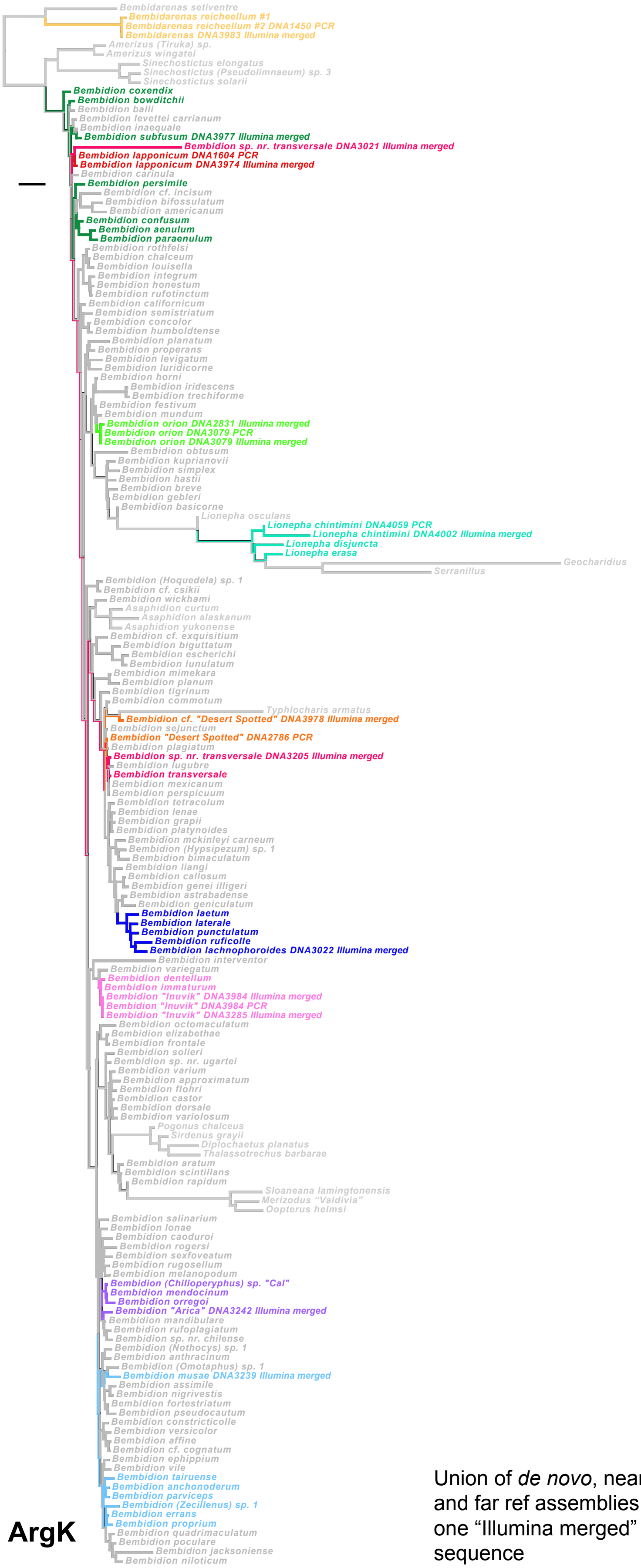

Union of *de novo*, near ref, and far ref assemblies form one "Illumina merged" sequence

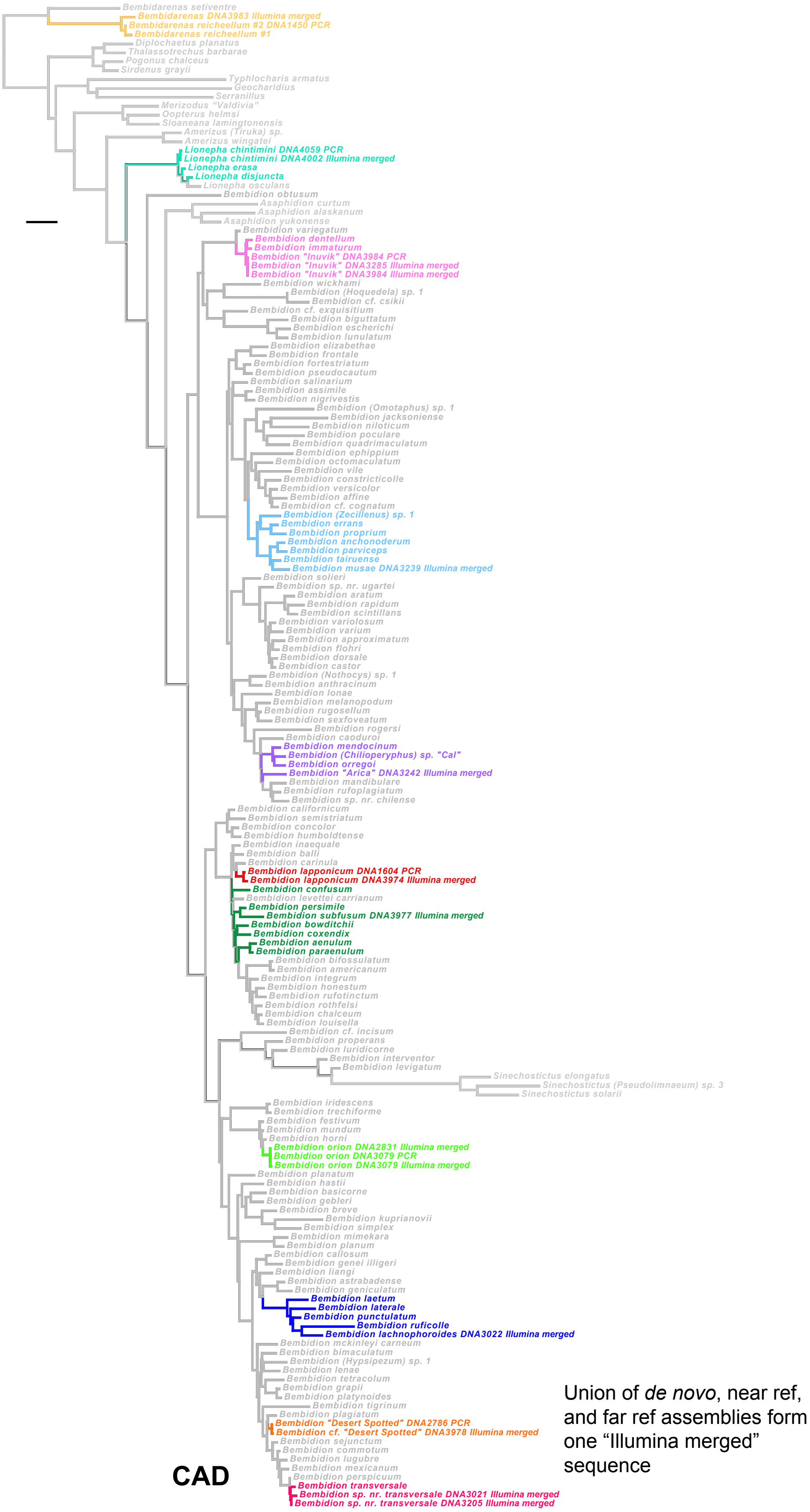
Bembidarenas setiventre
Bembidarenas DNA3983 Illumina merged
Bembidarenas reicheellum #2 DNA1450 PCR
Bembidarenas reicheellum #1
Diplochaetus planatus
Thalassotrechus barbarae
Pogonus chalceus
Sirdenus grayii
Typhlocharis armatus
Geocharidius
Serranillus
Merizodus "Valdivia"
Oopterus helmsi
Sloaneana lamingtonensis
Amerizus (Tiruka) sp.
Amerizus wingatei
Lionepha chintimini DNA4059 PCR
Lionepha chintimini DNA4002 Illumina merged
Lionepha erasa
Lionepha disjuncta
Lionepha osculans
Bembidion obtusum
Asaphidion curtum
Asaphidion alaskanum
Asaphidion yukonense
Bembidion variegatum
Bembidion dentellum
Bembidion immaturum
Bembidion "Inuvik" DNA3984 PCR
Bembidion "Inuvik" DNA3285 Illumina merged
Bembidion "Inuvik" DNA3984 Illumina merged
Bembidion wickhami
Bembidion (Hoquedela) sp. 1
Bembidion cf. csikii
Bembidion cf. exquisitium
Bembidion biguttatum
Bembidion escherichi
Bembidion lunulatum
Bembidion elizabethae
Bembidion frontale
Bembidion fortestriatum
Bembidion pseudocautum
Bembidion salinarium
Bembidion assimile
Bembidion nigrivestis
Bembidion (Omotaphus) sp. 1
Bembidion jacksoniense
Bembidion niloticum
Bembidion poculare
Bembidion quadrimaculatum
Bembidion ephippium
Bembidion octomaculatum
Bembidion vile
Bembidion constricticolle
Bembidion versicolor
Bembidion affine
Bembidion cf. cognatum
Bembidion (Zecillenus) sp. 1
Bembidion errans
Bembidion proprium
Bembidion anchonoderum
Bembidion parviceps
Bembidion tairuense
Bembidion musae DNA3239 Illumina merged
Bembidion solieri
Bembidion sp. nr. ugartei
Bembidion aratum
Bembidion rapidum
Bembidion scintillans
Bembidion variolosum
Bembidion varium
Bembidion approximatum
Bembidion flohri
Bembidion dorsale
Bembidion castor
Bembidion (Nothocys) sp. 1
Bembidion anthracinum
Bembidion lonae
Bembidion melanopodum
Bembidion rugosellum
Bembidion sexfoveatum
Bembidion rogersi
Bembidion caoduroi
Bembidion mendocinum
Bembidion (Chilioperyphus) sp. "Cal"
Bembidion orregoi
Bembidion "Arica" DNA3242 Illumina merged
Bembidion mandibulare
Bembidion rufoplagiatum
Bembidion sp. nr. chilense
Bembidion californicum
Bembidion semistriatum
Bembidion concolor
Bembidion humboldtense
Bembidion inaequale
Bembidion balli
Bembidion carinula
Bembidion lapponicum DNA1604 PCR
Bembidion lapponicum DNA3974 Illumina merged
Bembidion confusum
Bembidion levettei carrianum
Bembidion persimile
Bembidion subfusum DNA3977 Illumina merged
Bembidion bowditchii
Bembidion coxendix
Bembidion aenulum
Bembidion paraenulum
Bembidion bifossulatum
Bembidion americanum
Bembidion integrum
Bembidion honestum
Bembidion rufotinctum
Bembidion rothfelsi
Bembidion chalceum
Bembidion louisella
Bembidion cf. incisum
Bembidion properans
Bembidion luridicorne
Bembidion interventor
Bembidion levigatum
Sinechostictus elongatus
Sinechostictus (Pseudolimnaeum) sp. 3
Sinechostictus solarii
Bembidion iridescens
Bembidion trechiforme
Bembidion festivum
Bembidion mundum
Bembidion horni
Bembidion orion DNA2831 Illumina merged
Bembidion orion DNA3079 PCR
Bembidion orion DNA3079 Illumina merged
Bembidion planatum
Bembidion hastii
Bembidion basicorne
Bembidion gebleri
Bembidion breve
Bembidion kuprianovii
Bembidion simplex
Bembidion mimekara
Bembidion planum
Bembidion callosum
Bembidion genei illigeri
Bembidion liangi
Bembidion astrabadense
Bembidion geniculatum
Bembidion laetum
Bembidion laterale
Bembidion punctulatum
Bembidion ruficolle
Bembidion lachnophoroides DNA3022 Illumina merged
Bembidion mckinleyi carneum
Bembidion bimaculatum
Bembidion (Hypsipezum) sp. 1
Bembidion lenae
Bembidion tetracolum
Bembidion grapii
Bembidion platynoides
Bembidion tigrinum
Bembidion plagiatum
Bembidion "Desert Spotted" DNA2786 PCR
Bembidion cf. "Desert Spotted" DNA3978 Illumina merged
Bembidion sejunctum
Bembidion commotum
Bembidion lugubre
Bembidion mexicanum
Bembidion perspicuum
Bembidion transversale
Bembidion sp. nr. transversale DNA3021 Illumina merged
Bembidion sp. nr. transversale DNA3205 Illumina merged
CAD
Union of de novo, near ref, and far ref assemblies form one "Illumina merged" sequence

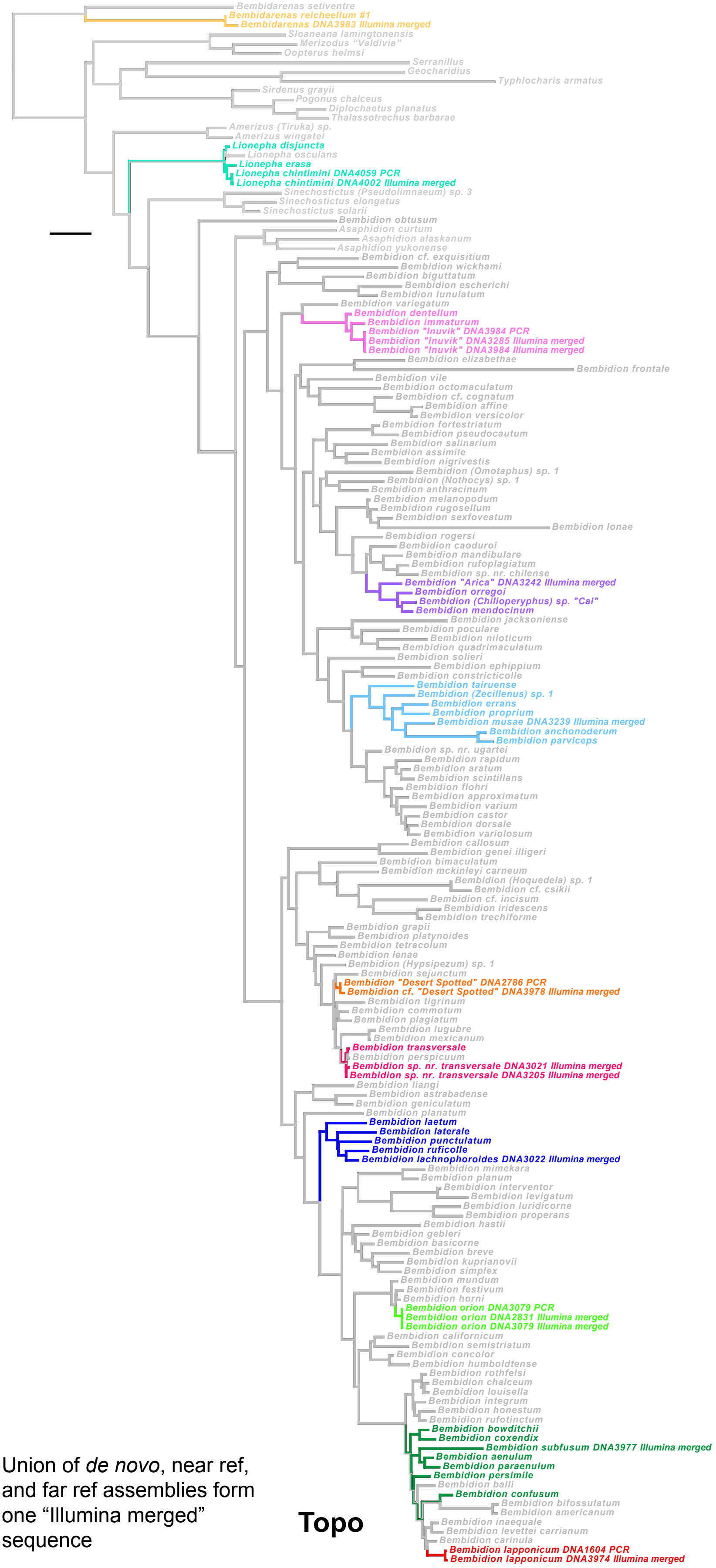

Union of *de novo*, near ref, and far ref assemblies form one “Illumina merged” sequence

Topo

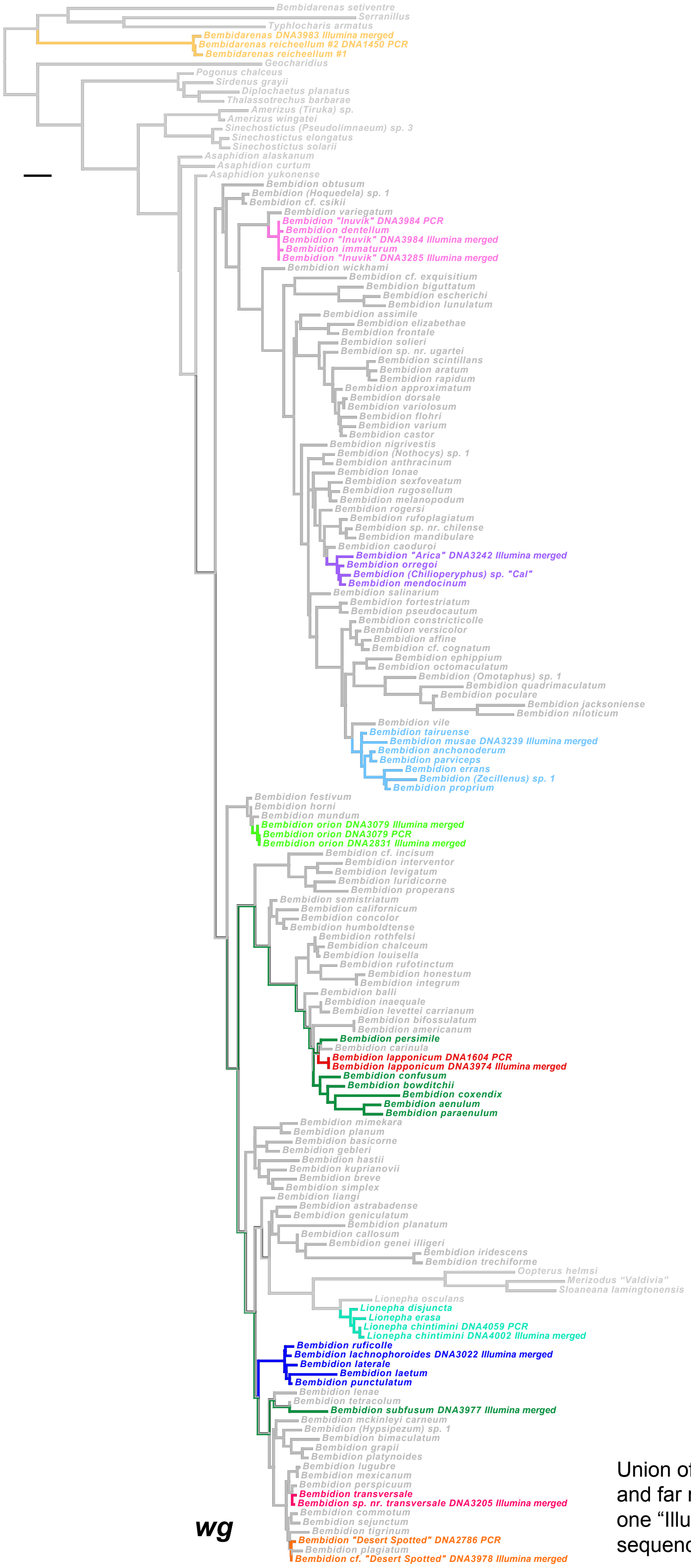

Union of *de novo*, near ref, and far ref assemblies form one "Illumina merged" sequence
