# Supplementary material for: Successful Recovery of Nuclear Protein-Coding Genes from Small Insects in Museums Using Illumina Sequencing
Source: PLoS One. 2015 Dec 30;10(12):e0143929. doi: 10.1371/journal.pone.0143929 (PMC4696846; doi:10.1371/journal.pone.0143929)
Supplement: S9 Fig — The placement of the IlluminaMerged sequences is shown relative to their prediction groups. Branches and taxon names of all specimens in the prediction group are indicated with a unique color. Bootstrap support values are given under nodes for branches that are supported with a bootstrap value greater than or equal to 50. (PDF) [file pone.0143929.s009.pdf]

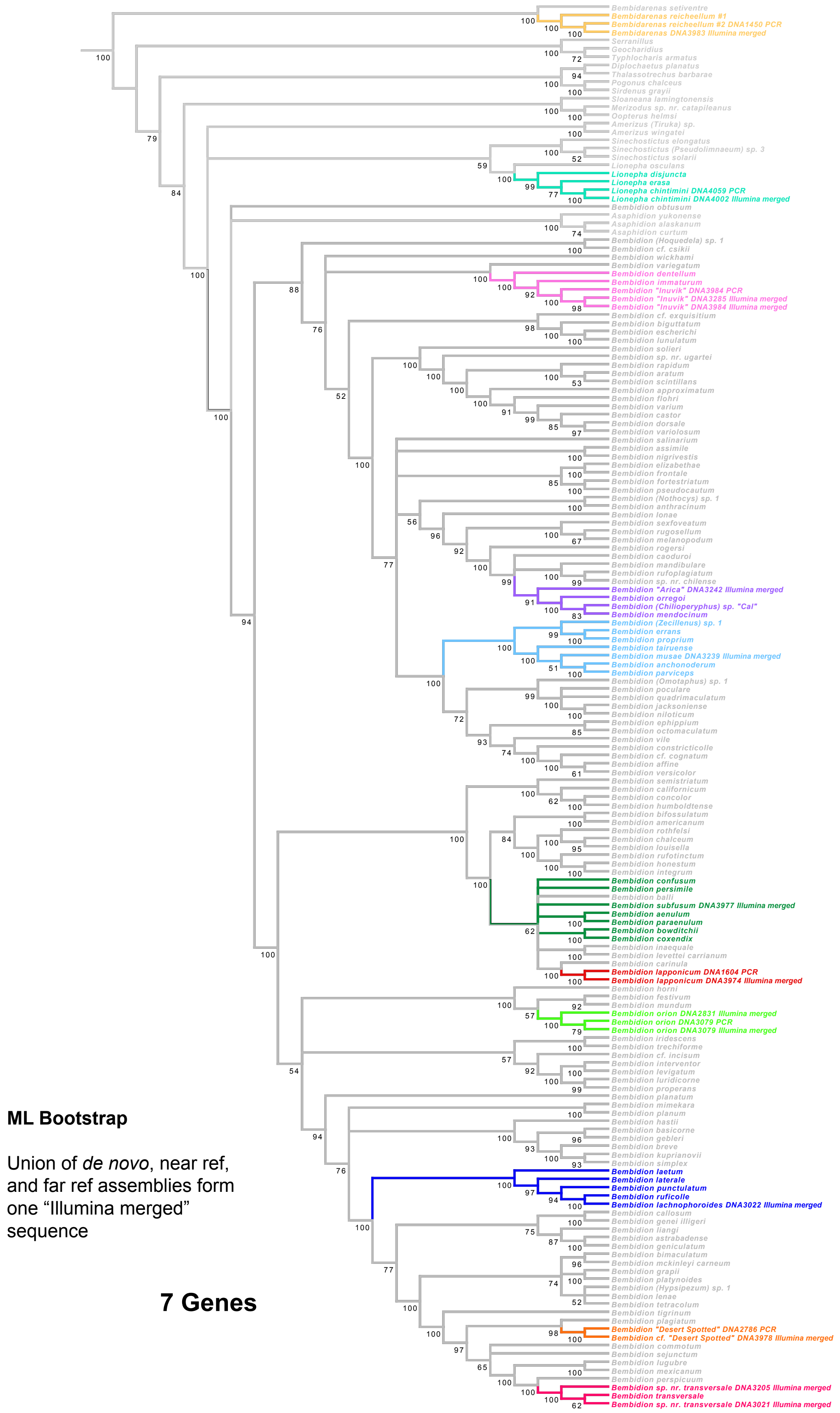

Bembidarenas setiventre
Bembidarenas reicheellum #1
Bembidarenas reicheellum #2 DNA1450 PCR
Bembidarenas DNA3983 Illumina merged
Serranillus
Geocharidius
Typhlocharis armatus
Diplochaetus planatus
Thalassotrechus barbarae
Pogonus chalceus
Sirdenus grayii
Sloaneana lamingtonensis
Merizodus sp. nr. catapileanus
Oopterus helmsi
Amerizus (Tiruka) sp.
Amerizus wingatei
Sinechostictus elongatus
Sinechostictus (Pseudolimnaeum) sp. 3
Sinechostictus solarii
Lionepha osculans
Lionepha disjuncta
Lionepha erasa
Lionepha chintimini DNA4059 PCR
Lionepha chintimini DNA4002 Illumina merged
Bembidion obtusum
Asaphidion yukonense
Asaphidion alaskanum
Asaphidion curtum
Bembidion (Hoquedela) sp. 1
Bembidion cf. csikii
Bembidion wickhami
Bembidion variegatum
Bembidion dentellum
Bembidion immaturum
Bembidion "Inuvik" DNA3984 PCR
Bembidion "Inuvik" DNA3285 Illumina merged
Bembidion "Inuvik" DNA3984 Illumina merged
Bembidion cf. exquisitium
Bembidion biguttatum
Bembidion escherichi
Bembidion lunulatum
Bembidion solieri
Bembidion sp. nr. ugartei
Bembidion rapidum
Bembidion aratum
Bembidion scintillans
Bembidion approximatum
Bembidion flohri
Bembidion varium
Bembidion castor
Bembidion dorsale
Bembidion variolosum
Bembidion salinarium
Bembidion assimile
Bembidion nigrivestis
Bembidion elizabethae
Bembidion frontale
Bembidion fortestriatum
Bembidion pseudocautum
Bembidion (Nothocys) sp. 1
Bembidion anthracinum
Bembidion lonae
Bembidion sexfoveatum
Bembidion rugosellum
Bembidion melanopodum
Bembidion rogersi
Bembidion caoduroi
Bembidion mandibulare
Bembidion rufoplagiatum
Bembidion sp. nr. chilense
Bembidion "Arica" DNA3242 Illumina merged
Bembidion orregoi
Bembidion (Chilioperyphus) sp. "Cal"
Bembidion mendocinum
Bembidion (Zecillenus) sp. 1
Bembidion errans
Bembidion proprium
Bembidion tairuense
Bembidion musae DNA3239 Illumina merged
Bembidion anchonoderum
Bembidion parviceps
Bembidion (Omotaphus) sp. 1
Bembidion poculare
Bembidion quadrimaculatum
Bembidion jacksoniense
Bembidion niloticum
Bembidion ephippium
Bembidion octomaculatum
Bembidion vile
Bembidion constricticolle
Bembidion cf. cognatum
Bembidion affine
Bembidion versicolor
Bembidion semistriatum
Bembidion californicum
Bembidion concolor
Bembidion humboldtense
Bembidion bifossulatum
Bembidion americanum
Bembidion rothfelsi
Bembidion chalceum
Bembidion louisella
Bembidion rufotinctum
Bembidion honestum
Bembidion integrum
Bembidion confusum
Bembidion persimile
Bembidion balli
Bembidion subfusum DNA3977 Illumina merged
Bembidion aenulum
Bembidion paraenulum
Bembidion bowditchii
Bembidion coxendix
Bembidion inaequale
Bembidion levettei carrianum
Bembidion carinula
Bembidion lapponicum DNA1604 PCR
Bembidion lapponicum DNA3974 Illumina merged
Bembidion horni
Bembidion festivum
Bembidion mundum
Bembidion orion DNA2831 Illumina merged
Bembidion orion DNA3079 PCR
Bembidion orion DNA3079 Illumina merged
Bembidion iridescens
Bembidion trechiforme
Bembidion cf. incisum
Bembidion interventor
Bembidion levigatum
Bembidion luridicorne
Bembidion properans
Bembidion planatum
Bembidion mimekara
Bembidion planum
Bembidion hastii
Bembidion basicorne
Bembidion gebleri
Bembidion breve
Bembidion kuprianovii
Bembidion simplex
Bembidion laetum
Bembidion laterale
Bembidion punctulatum
Bembidion ruficolle
Bembidion lachnophoroides DNA3022 Illumina merged
Bembidion callosum
Bembidion genei illigeri
Bembidion liangi
Bembidion astrabadense
Bembidion geniculatum
Bembidion bimaculatum
Bembidion mckinleyi carneum
Bembidion grapii
Bembidion platynoides
Bembidion (Hypsipezum) sp. 1
Bembidion lenae
Bembidion tetracolum
Bembidion tigrinum
Bembidion plagiatum
Bembidion "Desert Spotted" DNA2786 PCR
Bembidion cf. "Desert Spotted" DNA3978 Illumina merged
Bembidion commotum
Bembidion sejunctum
Bembidion lugubre
Bembidion mexicanum
Bembidion perspicuum
Bembidion sp. nr. transversale DNA3205 Illumina merged
Bembidion transversale
Bembidion sp. nr. transversale DNA3021 Illumina merged
ML Bootstrap
Union of de novo, near ref, and far ref assemblies form one "Illumina merged" sequence
7 Genes

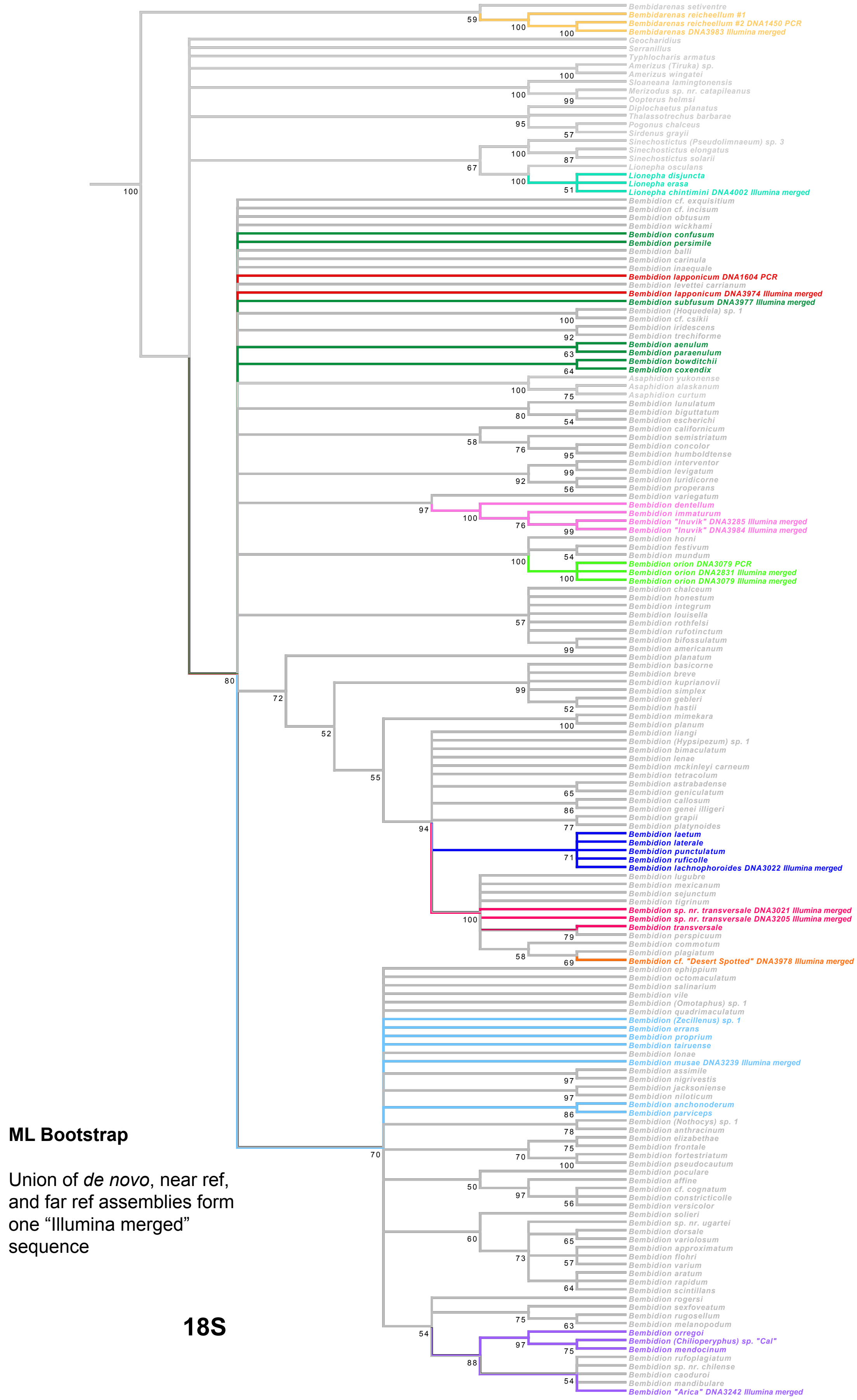

Bembidarenas setiventre
Bembidarenas reicheellum #1
Bembidarenas reicheellum #2 DNA1450 PCR
Bembidarenas DNA3983 Illumina merged
Geocharidius
Serranillus
Typhlocharis armatus
Amerizus (Tiruka) sp.
Amerizus wingatei
Sloaneana lamingtonensis
Merizodus sp. nr. catapileanus
Oopterus helmsi
Diplochaetus planatus
Thalassotrechus barbarae
Pogonus chalceus
Sirdenus grayii
Sinechostictus (Pseudolimnaeum) sp. 3
Sinechostictus elongatus
Sinechostictus solarii
Lionepha osculans
Lionepha disjuncta
Lionepha erasa
Lionepha chintimini DNA4002 Illumina merged
Bembidion cf. exquisitium
Bembidion cf. incisum
Bembidion obtusum
Bembidion wickhami
Bembidion confusum
Bembidion persimile
Bembidion balli
Bembidion carinula
Bembidion inaequale
Bembidion lapponicum DNA1604 PCR
Bembidion levettei carrianum
Bembidion lapponicum DNA3974 Illumina merged
Bembidion subfusum DNA3977 Illumina merged
Bembidion (Hoquedela) sp. 1
Bembidion cf. csikii
Bembidion iridescens
Bembidion trechiforme
Bembidion aenulum
Bembidion paraenulum
Bembidion bowditchii
Bembidion coxendix
Asaphidion yukonense
Asaphidion alaskanum
Asaphidion curtum
Bembidion lunulatum
Bembidion biguttatum
Bembidion escherichi
Bembidion californicum
Bembidion semistriatum
Bembidion concolor
Bembidion humboldtense
Bembidion interventor
Bembidion levigatum
Bembidion luridicorne
Bembidion properans
Bembidion variegatum
Bembidion dentellum
Bembidion immaturum
Bembidion "Inuvik" DNA3285 Illumina merged
Bembidion "Inuvik" DNA3984 Illumina merged
Bembidion horni
Bembidion festivum
Bembidion mundum
Bembidion orion DNA3079 PCR
Bembidion orion DNA2831 Illumina merged
Bembidion orion DNA3079 Illumina merged
Bembidion chalceum
Bembidion honestum
Bembidion integrum
Bembidion louisella
Bembidion rothfelsi
Bembidion rufotinctum
Bembidion bifossulatum
Bembidion americanum
Bembidion planatum
Bembidion basicorne
Bembidion breve
Bembidion kuprianovii
Bembidion simplex
Bembidion gebleri
Bembidion hastii
Bembidion mimekara
Bembidion planum
Bembidion liangi
Bembidion (Hypsipezum) sp. 1
Bembidion bimaculatum
Bembidion lenae
Bembidion mckinleyi carneum
Bembidion tetracolum
Bembidion astrabadense
Bembidion geniculatum
Bembidion callosum
Bembidion genei illigeri
Bembidion grapii
Bembidion platynoides
Bembidion laetum
Bembidion laterale
Bembidion punctulatum
Bembidion ruficolle
Bembidion lachnophoroides DNA3022 Illumina merged
Bembidion lugubre
Bembidion mexicanum
Bembidion sejunctum
Bembidion tigrinum
Bembidion sp. nr. transversale DNA3021 Illumina merged
Bembidion sp. nr. transversale DNA3205 Illumina merged
Bembidion transversale
Bembidion perspicuum
Bembidion commotum
Bembidion plagiatum
Bembidion cf. "Desert Spotted" DNA3978 Illumina merged
Bembidion ephippium
Bembidion octomaculatum
Bembidion salinarium
Bembidion vile
Bembidion (Omotaphus) sp. 1
Bembidion quadrimaculatum
Bembidion (Zecillenus) sp. 1
Bembidion errans
Bembidion proprium
Bembidion tairuense
Bembidion lonae
Bembidion musae DNA3239 Illumina merged
Bembidion assimile
Bembidion nigrivestis
Bembidion jacksoniense
Bembidion niloticum
Bembidion anchonoderum
Bembidion parviceps
Bembidion (Nothocys) sp. 1
Bembidion anthracinum
Bembidion elizabethae
Bembidion frontale
Bembidion fortestriatum
Bembidion pseudocautum
Bembidion poculare
Bembidion affine
Bembidion cf. cognatum
Bembidion constricticolle
Bembidion versicolor
Bembidion solieri
Bembidion sp. nr. ugartei
Bembidion dorsale
Bembidion variolosum
Bembidion approximatum
Bembidion flohri
Bembidion varium
Bembidion aratum
Bembidion rapidum
Bembidion scintillans
Bembidion rogersi
Bembidion sexfoveatum
Bembidion rugosellum
Bembidion melanopodum
Bembidion orregoi
Bembidion (Chilioperyphus) sp. "Cal"
Bembidion mendocinum
Bembidion rufoplagiatum
Bembidion sp. nr. chilense
Bembidion caoduroi
Bembidion mandibulare
Bembidion "Arica" DNA3242 Illumina merged
ML Bootstrap
Union of de novo, near ref, and far ref assemblies form one “Illumina merged” sequence
18S

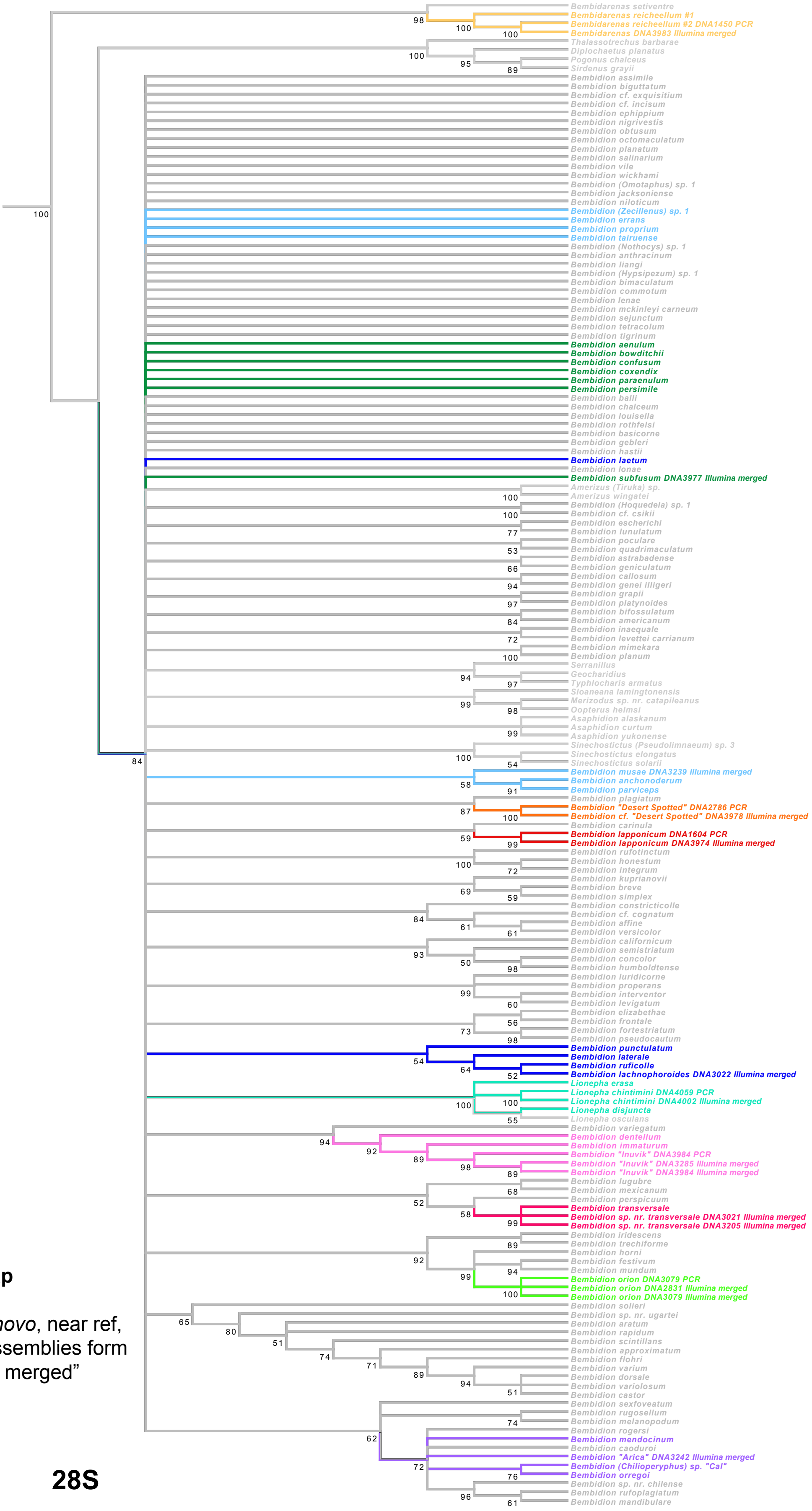

ML Bootstrap

Union of *de novo*, near ref, and far ref assemblies form one "Illumina merged" sequence

28S

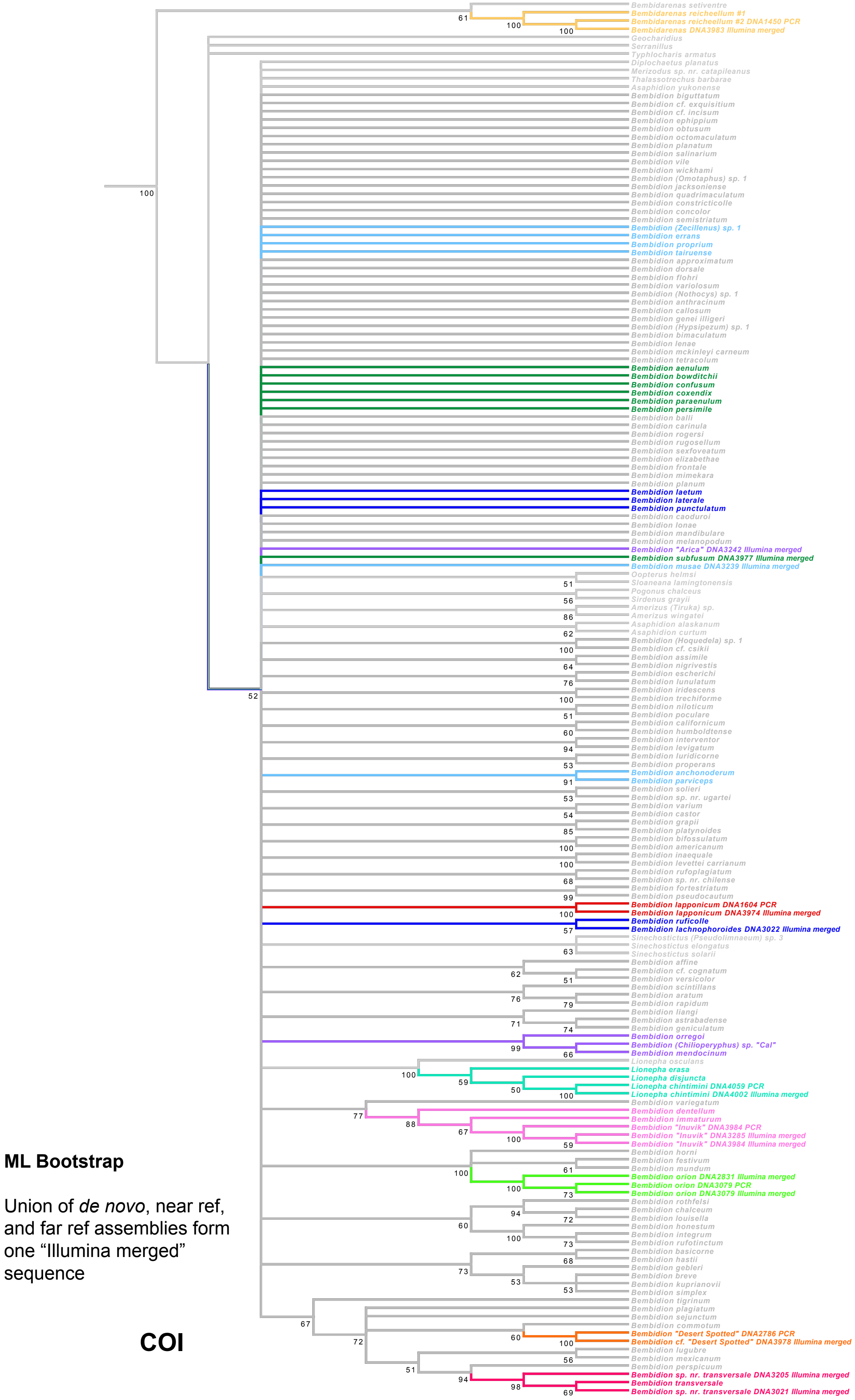
Bembidarenas setiventre
Bembidarenas reicheellum #1
Bembidarenas reicheellum #2 DNA1450 PCR
Bembidarenas DNA3983 Illumina merged
Geocharidius
Serranillus
Typhlocharis armatus
Diplochaetus planatus
Merizodus sp. nr. catapileanus
Thalassotrechus barbarae
Asaphidion yukonense
Bembidion biguttatum
Bembidion cf. exquisitium
Bembidion cf. incisum
Bembidion ephippium
Bembidion obtusum
Bembidion octomaculatum
Bembidion planatum
Bembidion salinarium
Bembidion vile
Bembidion wickhami
Bembidion (Omotaphus) sp. 1
Bembidion jacksoniense
Bembidion quadrimaculatum
Bembidion constricticolle
Bembidion concolor
Bembidion semistriatum
Bembidion (Zecillenus) sp. 1
Bembidion errans
Bembidion proprium
Bembidion tairuense
Bembidion approximatum
Bembidion dorsale
Bembidion flohri
Bembidion variolosum
Bembidion (Nothocys) sp. 1
Bembidion anthracinum
Bembidion callosum
Bembidion genei illigeri
Bembidion (Hypsipezum) sp. 1
Bembidion bimaculatum
Bembidion lenae
Bembidion mckinleyi carneum
Bembidion tetracolum
Bembidion aenulum
Bembidion bowditchii
Bembidion confusum
Bembidion coxendix
Bembidion paraenulum
Bembidion persimile
Bembidion balli
Bembidion carinula
Bembidion rogersi
Bembidion rugosellum
Bembidion sexfoveatum
Bembidion elizabethae
Bembidion frontale
Bembidion mimekara
Bembidion planum
Bembidion laetum
Bembidion laterale
Bembidion punctulatum
Bembidion caoduroi
Bembidion lonae
Bembidion mandibulare
Bembidion melanopodum
Bembidion "Arica" DNA3242 Illumina merged
Bembidion subfusum DNA3977 Illumina merged
Bembidion musae DNA3239 Illumina merged
Oopterus helmsi
Sloaneana lamingtonensis
Pogonus chalceus
Sirdenus grayii
Amerizus (Tiruka) sp.
Amerizus wingatei
Asaphidion alaskanum
Asaphidion curtum
Bembidion (Hoquedela) sp. 1
Bembidion cf. csikii
Bembidion assimile
Bembidion nigrivestis
Bembidion escherichi
Bembidion lunulatum
Bembidion iridescens
Bembidion trechiforme
Bembidion niloticum
Bembidion poculare
Bembidion californicum
Bembidion humboldtense
Bembidion interventor
Bembidion levigatum
Bembidion luridicorne
Bembidion properans
Bembidion anchonoderum
Bembidion parviceps
Bembidion solieri
Bembidion sp. nr. ugartei
Bembidion varium
Bembidion castor
Bembidion grapii
Bembidion platynoides
Bembidion bifossulatum
Bembidion americanum
Bembidion inaequale
Bembidion levettei carrianum
Bembidion rufoplagiatum
Bembidion sp. nr. chilense
Bembidion fortestriatum
Bembidion pseudocautum
Bembidion lapponicum DNA1604 PCR
Bembidion lapponicum DNA3974 Illumina merged
Bembidion ruficolle
Bembidion lachnophoroides DNA3022 Illumina merged
Sinechostictus (Pseudolimnaeum) sp. 3
Sinechostictus elongatus
Sinechostictus solarii
Bembidion affine
Bembidion cf. cognatum
Bembidion versicolor
Bembidion scintillans
Bembidion aratum
Bembidion rapidum
Bembidion liangi
Bembidion astrabadense
Bembidion geniculatum
Bembidion orregoi
Bembidion (Chilioperyphus) sp. "Cal"
Bembidion mendocinum
Lionepha osculans
Lionepha erasa
Lionepha disjuncta
Lionepha chintimini DNA4059 PCR
Lionepha chintimini DNA4002 Illumina merged
Bembidion variegatum
Bembidion dentellum
Bembidion immaturum
Bembidion "Inuvik" DNA3984 PCR
Bembidion "Inuvik" DNA3285 Illumina merged
Bembidion "Inuvik" DNA3984 Illumina merged
Bembidion horni
Bembidion festivum
Bembidion mundum
Bembidion orion DNA2831 Illumina merged
Bembidion orion DNA3079 PCR
Bembidion orion DNA3079 Illumina merged
Bembidion rothfelsi
Bembidion chalceum
Bembidion louisella
Bembidion honestum
Bembidion integrum
Bembidion rufotinctum
Bembidion basicorne
Bembidion hastii
Bembidion gebleri
Bembidion breve
Bembidion kuprianovii
Bembidion simplex
Bembidion tigrinum
Bembidion plagiatum
Bembidion sejunctum
Bembidion commotum
Bembidion "Desert Spotted" DNA2786 PCR
Bembidion cf. "Desert Spotted" DNA3978 Illumina merged
Bembidion lugubre
Bembidion mexicanum
Bembidion perspicuum
Bembidion sp. nr. transversale DNA3205 Illumina merged
Bembidion transversale
Bembidion sp. nr. transversale DNA3021 Illumina merged
ML Bootstrap
Union of de novo, near ref, and far ref assemblies form one "Illumina merged" sequence
COI

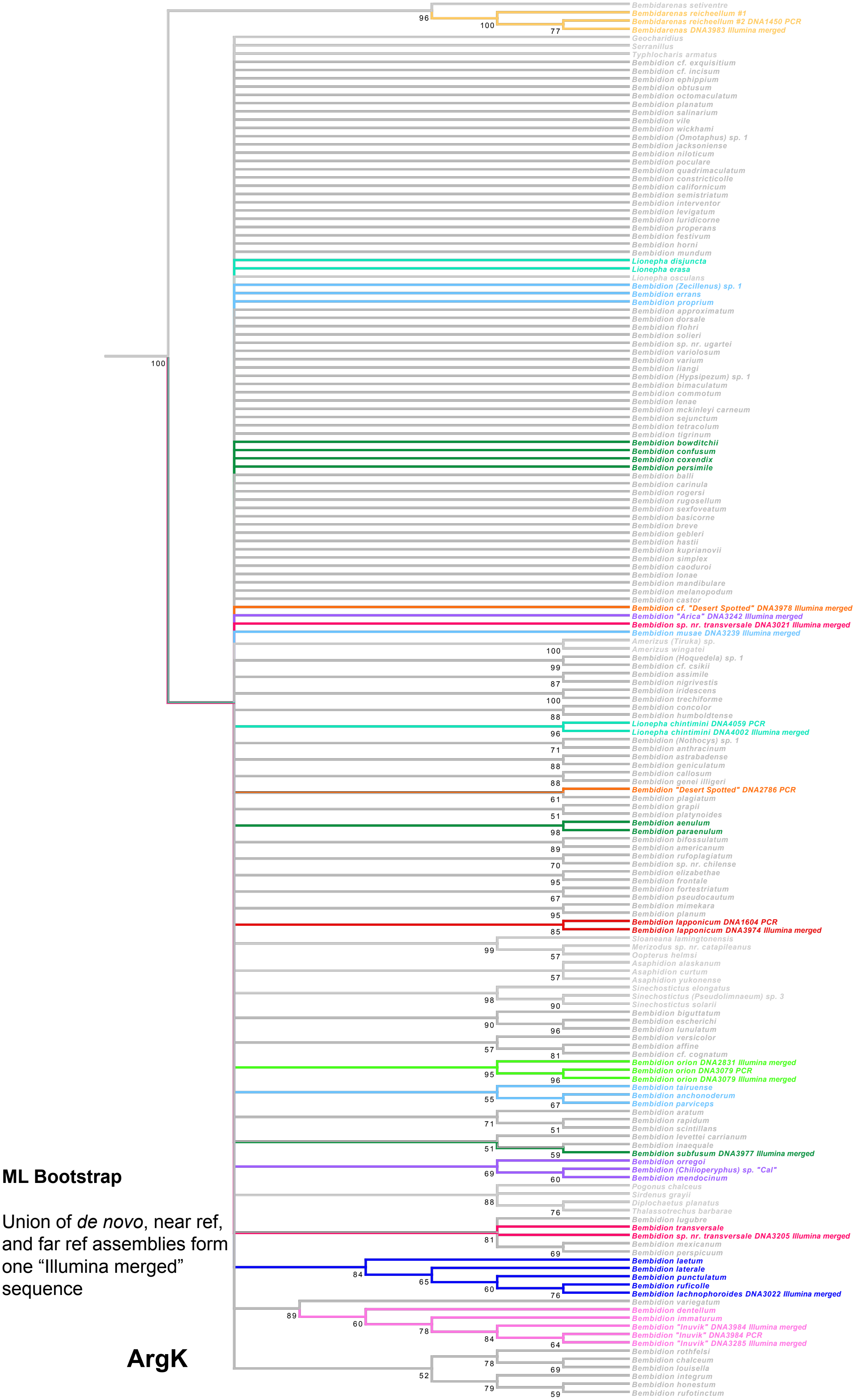

Bembidarenas setiventre
Bembidarenas reicheellum #1
Bembidarenas reicheellum #2 DNA1450 PCR
Bembidarenas DNA3983 Illumina merged
Geocharidius
Serranillus
Typhlocharis armatus
Bembidion cf. exquisitium
Bembidion cf. incisum
Bembidion ephippium
Bembidion obtusum
Bembidion octomaculatum
Bembidion planatum
Bembidion salinarium
Bembidion vile
Bembidion wickhami
Bembidion (Omotaphus) sp. 1
Bembidion jacksoniense
Bembidion niloticum
Bembidion poculare
Bembidion quadrimaculatum
Bembidion constricticolle
Bembidion californicum
Bembidion semistriatum
Bembidion interventor
Bembidion levigatum
Bembidion luridicorne
Bembidion properans
Bembidion festivum
Bembidion horni
Bembidion mundum
Lionepha disjuncta
Lionepha erasa
Lionepha osculans
Bembidion (Zecillenus) sp. 1
Bembidion errans
Bembidion proprium
Bembidion approximatum
Bembidion dorsale
Bembidion flohri
Bembidion solieri
Bembidion sp. nr. ugartei
Bembidion variolosum
Bembidion varium
Bembidion liangi
Bembidion (Hypsipezum) sp. 1
Bembidion bimaculatum
Bembidion commotum
Bembidion lenae
Bembidion mckinleyi carneum
Bembidion sejunctum
Bembidion tetracolum
Bembidion tigrinum
Bembidion bowditchii
Bembidion confusum
Bembidion coxendix
Bembidion persimile
Bembidion balli
Bembidion carinula
Bembidion rogersi
Bembidion rugosellum
Bembidion sexfoveatum
Bembidion basicorne
Bembidion breve
Bembidion gebleri
Bembidion hastii
Bembidion kuprianovii
Bembidion simplex
Bembidion caoduroi
Bembidion lonae
Bembidion mandibulare
Bembidion melanopodum
Bembidion castor
Bembidion cf. "Desert Spotted" DNA3978 Illumina merged
Bembidion "Arica" DNA3242 Illumina merged
Bembidion sp. nr. transversale DNA3021 Illumina merged
Bembidion musae DNA3239 Illumina merged
Amerizus (Tiruka) sp.
Amerizus wingatei
Bembidion (Hoquedela) sp. 1
Bembidion cf. csikii
Bembidion assimile
Bembidion nigrivestis
Bembidion iridescens
Bembidion trechiforme
Bembidion concolor
Bembidion humboldtense
Lionepha chintimini DNA4059 PCR
Lionepha chintimini DNA4002 Illumina merged
Bembidion (Nothocys) sp. 1
Bembidion anthracinum
Bembidion astrabadense
Bembidion geniculatum
Bembidion callosum
Bembidion genei illigeri
Bembidion "Desert Spotted" DNA2786 PCR
Bembidion plagiatum
Bembidion grapii
Bembidion platynoides
Bembidion aenulum
Bembidion paraenulum
Bembidion bifossulatum
Bembidion americanum
Bembidion rufoplagiatum
Bembidion sp. nr. chilense
Bembidion elizabethae
Bembidion frontale
Bembidion fortestriatum
Bembidion pseudocautum
Bembidion mimekara
Bembidion planum
Bembidion lapponicum DNA1604 PCR
Bembidion lapponicum DNA3974 Illumina merged
Sloaneana lamingtonensis
Merizodus sp. nr. catapileanus
Oopterus helmsi
Asaphidion alaskanum
Asaphidion curtum
Asaphidion yukonense
Sinechostictus elongatus
Sinechostictus (Pseudolimnaeum) sp. 3
Sinechostictus solarii
Bembidion biguttatum
Bembidion escherichi
Bembidion lunulatum
Bembidion versicolor
Bembidion affine
Bembidion cf. cognatum
Bembidion orion DNA2831 Illumina merged
Bembidion orion DNA3079 PCR
Bembidion orion DNA3079 Illumina merged
Bembidion tairuense
Bembidion anchonoderum
Bembidion parviceps
Bembidion aratum
Bembidion rapidum
Bembidion scintillans
Bembidion levettei carrianum
Bembidion inaequale
Bembidion subfusum DNA3977 Illumina merged
Bembidion orregoi
Bembidion (Chilioperyphus) sp. "Cal"
Bembidion mendocinum
Pogonus chalceus
Sirdenus grayii
Diplochaetus planatus
Thalassotrechus barbarae
Bembidion lugubre
Bembidion transversale
Bembidion sp. nr. transversale DNA3205 Illumina merged
Bembidion mexicanum
Bembidion perspicuum
Bembidion laetum
Bembidion laterale
Bembidion punctulatum
Bembidion ruficolle
Bembidion lachnophoroides DNA3022 Illumina merged
Bembidion variegatum
Bembidion dentellum
Bembidion immaturum
Bembidion "Inuvik" DNA3984 Illumina merged
Bembidion "Inuvik" DNA3984 PCR
Bembidion "Inuvik" DNA3285 Illumina merged
Bembidion rothfelsi
Bembidion chalceum
Bembidion louisella
Bembidion integrum
Bembidion honestum
Bembidion rufotinctum
ML Bootstrap
Union of de novo, near ref, and far ref assemblies form one "Illumina merged" sequence
ArgK

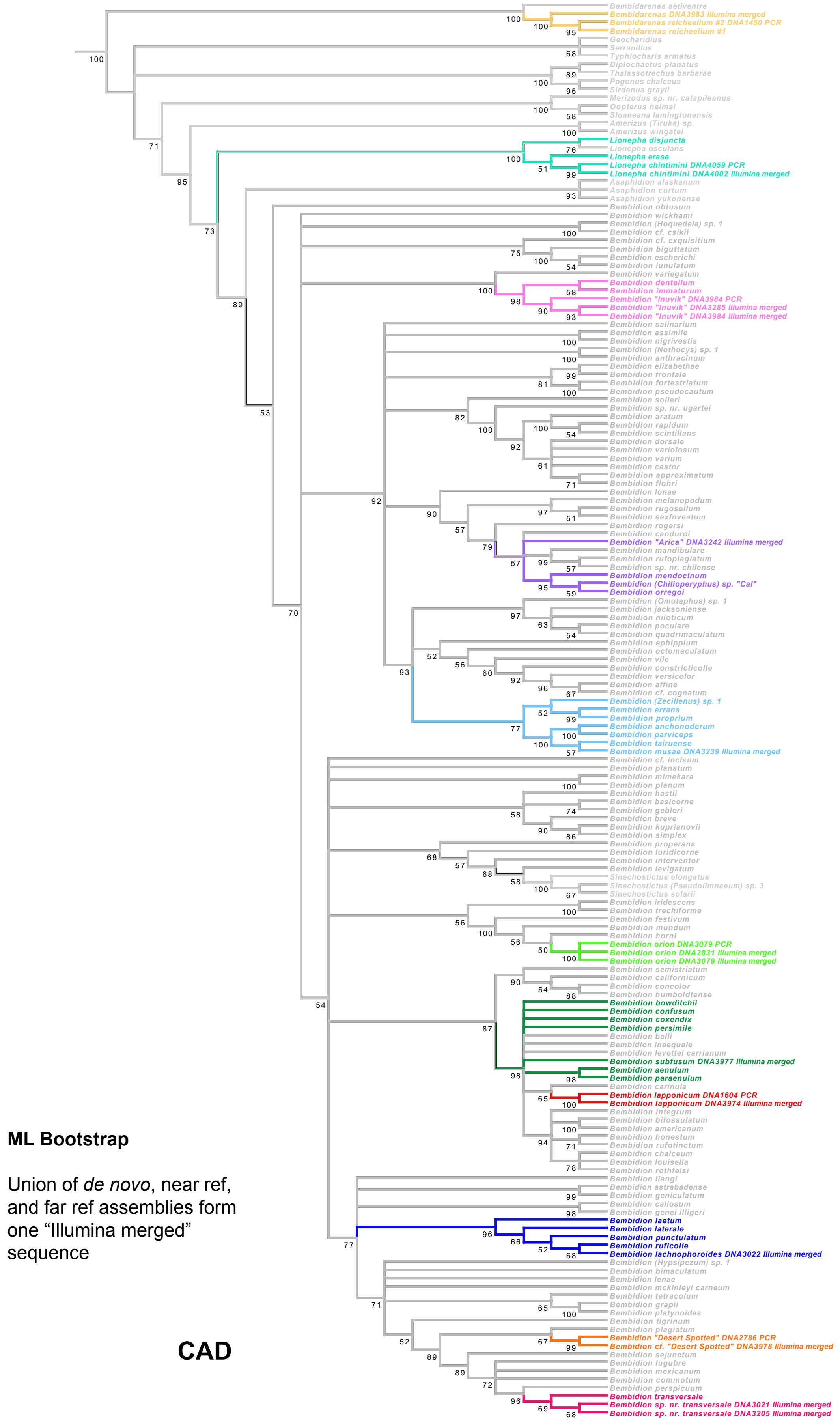
Bembidarenas setiventre
Bembidarenas DNA3983 Illumina merged
Bembidarenas reicheellum #2 DNA1450 PCR
Bembidarenas reicheellum #1
Geocharidius
Serranillus
Typhlocharis armatus
Diplochaetus planatus
Thalassotrechus barbarae
Pogonus chalceus
Sirdenus grayii
Merizodus sp. nr. catapileanus
Oopterus helmsi
Sloaneana lamingtonensis
Amerizus (Tiruka) sp.
Amerizus wingatei
Lionepha disjuncta
Lionepha osculans
Lionepha erasa
Lionepha chintimini DNA4059 PCR
Lionepha chintimini DNA4002 Illumina merged
Asaphidion alaskanum
Asaphidion curtum
Asaphidion yukonense
Bembidion obtusum
Bembidion wickhami
Bembidion (Hoquedela) sp. 1
Bembidion cf. csikii
Bembidion cf. exquisitium
Bembidion biguttatum
Bembidion escherichi
Bembidion lunulatum
Bembidion variegatum
Bembidion dentellum
Bembidion immaturum
Bembidion "Inuvik" DNA3984 PCR
Bembidion "Inuvik" DNA3285 Illumina merged
Bembidion "Inuvik" DNA3984 Illumina merged
Bembidion salinarium
Bembidion assimile
Bembidion nigrivestis
Bembidion (Nothocys) sp. 1
Bembidion anthracinum
Bembidion elizabethae
Bembidion frontale
Bembidion fortestriatum
Bembidion pseudocautum
Bembidion solieri
Bembidion sp. nr. ugartei
Bembidion aratum
Bembidion rapidum
Bembidion scintillans
Bembidion dorsale
Bembidion variolosum
Bembidion varium
Bembidion castor
Bembidion approximatum
Bembidion flohri
Bembidion lonae
Bembidion melanopodum
Bembidion rugosellum
Bembidion sexfoveatum
Bembidion rogersi
Bembidion caoduroi
Bembidion "Arica" DNA3242 Illumina merged
Bembidion mandibulare
Bembidion rufoplagiatum
Bembidion sp. nr. chilense
Bembidion mendocinum
Bembidion (Chilioperyphus) sp. "Cal"
Bembidion orregoi
Bembidion (Omotaphus) sp. 1
Bembidion jacksoniense
Bembidion niloticum
Bembidion poculare
Bembidion quadrimaculatum
Bembidion ephippium
Bembidion octomaculatum
Bembidion vile
Bembidion constricticolle
Bembidion versicolor
Bembidion affine
Bembidion cf. cognatum
Bembidion (Zecillenus) sp. 1
Bembidion errans
Bembidion proprium
Bembidion anchonoderum
Bembidion parviceps
Bembidion tairuense
Bembidion musae DNA3239 Illumina merged
Bembidion cf. incisum
Bembidion planatum
Bembidion mimekara
Bembidion planum
Bembidion hastii
Bembidion basicorne
Bembidion gebleri
Bembidion breve
Bembidion kuprianovii
Bembidion simplex
Bembidion properans
Bembidion luridicorne
Bembidion interventor
Bembidion levigatum
Sinechostictus elongatus
Sinechostictus (Pseudolimnaeum) sp. 3
Sinechostictus solarii
Bembidion iridescens
Bembidion trechiforme
Bembidion festivum
Bembidion mundum
Bembidion horni
Bembidion orion DNA3079 PCR
Bembidion orion DNA2831 Illumina merged
Bembidion orion DNA3079 Illumina merged
Bembidion semistriatum
Bembidion californicum
Bembidion concolor
Bembidion humboldtense
Bembidion bowditchii
Bembidion confusum
Bembidion coxendix
Bembidion persimile
Bembidion balli
Bembidion inaequale
Bembidion levettei carrianum
Bembidion subfusum DNA3977 Illumina merged
Bembidion aenulum
Bembidion paraenulum
Bembidion carinula
Bembidion lapponicum DNA1604 PCR
Bembidion lapponicum DNA3974 Illumina merged
Bembidion integrum
Bembidion bifossulatum
Bembidion americanum
Bembidion honestum
Bembidion rufotinctum
Bembidion chalceum
Bembidion louisella
Bembidion rothfelsi
Bembidion liangi
Bembidion astrabadense
Bembidion geniculatum
Bembidion callosum
Bembidion genei illigeri
Bembidion laetum
Bembidion laterale
Bembidion punctulatum
Bembidion ruficolle
Bembidion lachnophoroides DNA3022 Illumina merged
Bembidion (Hypsipezum) sp. 1
Bembidion bimaculatum
Bembidion lenae
Bembidion mckinleyi carneum
Bembidion tetracolum
Bembidion grapii
Bembidion platynoides
Bembidion tigrinum
Bembidion plagiatum
Bembidion "Desert Spotted" DNA2786 PCR
Bembidion cf. "Desert Spotted" DNA3978 Illumina merged
Bembidion sejunctum
Bembidion lugubre
Bembidion mexicanum
Bembidion commotum
Bembidion perspicuum
Bembidion transversale
Bembidion sp. nr. transversale DNA3021 Illumina merged
Bembidion sp. nr. transversale DNA3205 Illumina merged
ML Bootstrap
Union of *de novo*, near ref, and far ref assemblies form one "Illumina merged" sequence
CAD

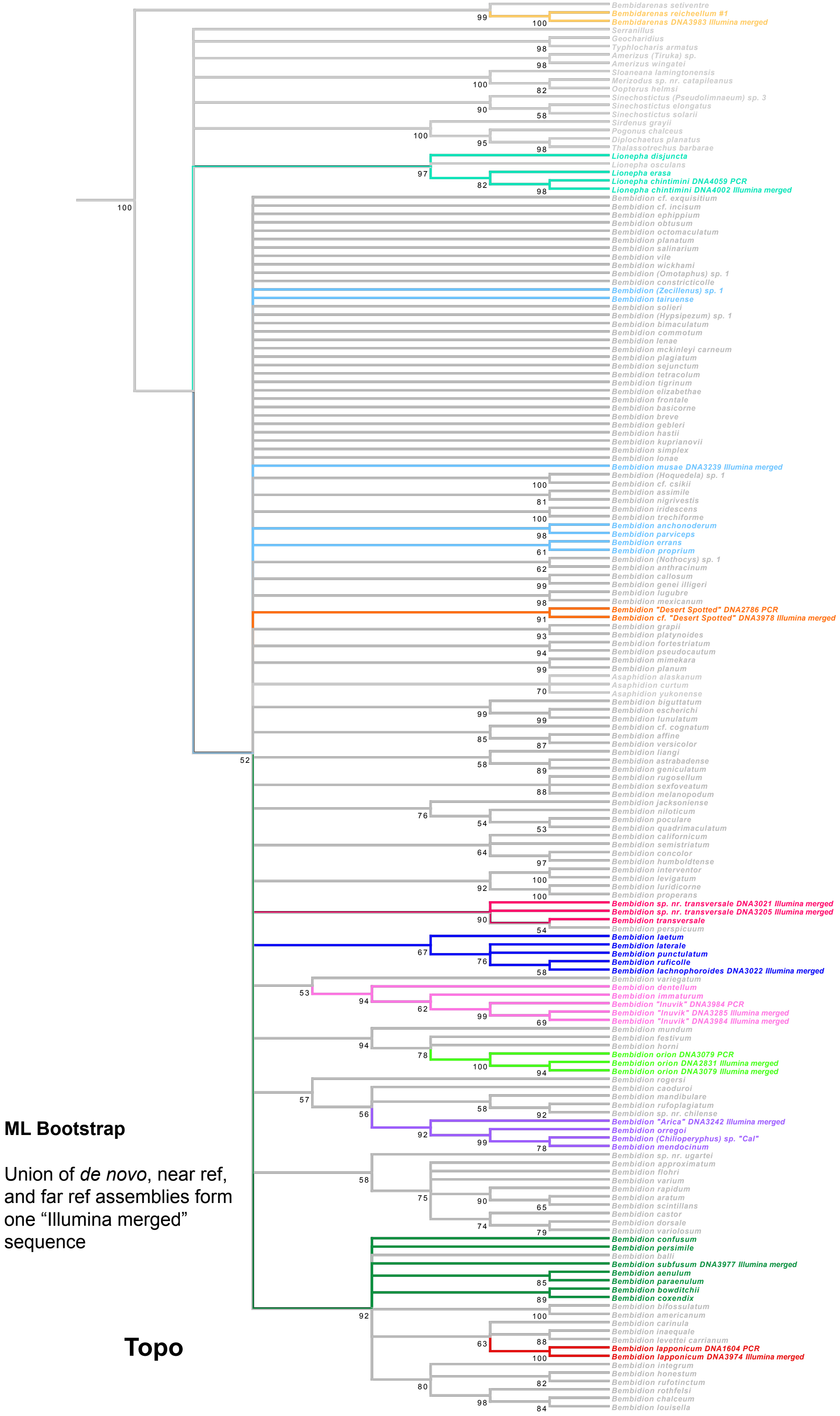
Bembidarenas setiventre
Bembidarenas reicheellum #1
Bembidarenas DNA3983 Illumina merged
Serranillus
Geocharidius
Typhlocharis armatus
Amerizus (Tiruka) sp.
Amerizus wingatei
Sloaneana lamingtonensis
Merizodus sp. nr. catapileanus
Oopterus helmsi
Sinechostictus (Pseudolimnaeum) sp. 3
Sinechostictus elongatus
Sinechostictus solarii
Sirdenus grayii
Pogonus chalceus
Diplochaetus planatus
Thalassotrechus barbarae
Lionepha disjuncta
Lionepha osculans
Lionepha erasa
Lionepha chintimini DNA4059 PCR
Lionepha chintimini DNA4002 Illumina merged
Bembidion cf. exquisitium
Bembidion cf. incisum
Bembidion ephippium
Bembidion obtusum
Bembidion octomaculatum
Bembidion planatum
Bembidion salinarium
Bembidion vile
Bembidion wickhami
Bembidion (Omotaphus) sp. 1
Bembidion constricticolle
Bembidion (Zecillenus) sp. 1
Bembidion tairuense
Bembidion solieri
Bembidion (Hypsipezum) sp. 1
Bembidion bimaculatum
Bembidion commotum
Bembidion lenae
Bembidion mckinleyi carneum
Bembidion plagiatum
Bembidion sejunctum
Bembidion tetracolum
Bembidion tigrinum
Bembidion elizabethae
Bembidion frontale
Bembidion basicorne
Bembidion breve
Bembidion gebleri
Bembidion hastii
Bembidion kuprianovii
Bembidion simplex
Bembidion lonae
Bembidion musae DNA3239 Illumina merged
Bembidion (Hoquedela) sp. 1
Bembidion cf. csikii
Bembidion assimile
Bembidion nigrivestis
Bembidion iridescens
Bembidion trechiforme
Bembidion anchonoderum
Bembidion parviceps
Bembidion errans
Bembidion proprium
Bembidion (Nothocys) sp. 1
Bembidion anthracinum
Bembidion callosum
Bembidion genei illigeri
Bembidion lugubre
Bembidion mexicanum
Bembidion "Desert Spotted" DNA2786 PCR
Bembidion cf. "Desert Spotted" DNA3978 Illumina merged
Bembidion grapii
Bembidion platynoides
Bembidion fortestriatum
Bembidion pseudocautum
Bembidion mimekara
Bembidion planum
Asaphidion alaskanum
Asaphidion curtum
Asaphidion yukonense
Bembidion biguttatum
Bembidion escherichi
Bembidion lunulatum
Bembidion cf. cognatum
Bembidion affine
Bembidion versicolor
Bembidion liangi
Bembidion astrabadense
Bembidion geniculatum
Bembidion rugosellum
Bembidion sexfoveatum
Bembidion melanopodum
Bembidion jacksoniense
Bembidion niloticum
Bembidion poculare
Bembidion quadrimaculatum
Bembidion californicum
Bembidion semistriatum
Bembidion concolor
Bembidion humboldtense
Bembidion interventor
Bembidion levigatum
Bembidion luridicorne
Bembidion properans
Bembidion sp. nr. transversale DNA3021 Illumina merged
Bembidion sp. nr. transversale DNA3205 Illumina merged
Bembidion transversale
Bembidion perspicuum
Bembidion laetum
Bembidion laterale
Bembidion punctulatum
Bembidion ruficolle
Bembidion lachnophoroides DNA3022 Illumina merged
Bembidion variegatum
Bembidion dentellum
Bembidion immaturum
Bembidion "Inuvik" DNA3984 PCR
Bembidion "Inuvik" DNA3285 Illumina merged
Bembidion "Inuvik" DNA3984 Illumina merged
Bembidion mundum
Bembidion festivum
Bembidion horni
Bembidion orion DNA3079 PCR
Bembidion orion DNA2831 Illumina merged
Bembidion orion DNA3079 Illumina merged
Bembidion rogersi
Bembidion caoduroi
Bembidion mandibulare
Bembidion rufoplagiatum
Bembidion sp. nr. chilense
Bembidion "Arica" DNA3242 Illumina merged
Bembidion orregoi
Bembidion (Chilioperyphus) sp. "Cal"
Bembidion mendocinum
Bembidion sp. nr. ugartei
Bembidion approximatum
Bembidion flohri
Bembidion varium
Bembidion rapidum
Bembidion aratum
Bembidion scintillans
Bembidion castor
Bembidion dorsale
Bembidion variolosum
Bembidion confusum
Bembidion persimile
Bembidion balli
Bembidion subfusum DNA3977 Illumina merged
Bembidion aenulum
Bembidion paraenulum
Bembidion bowditchii
Bembidion coxendix
Bembidion bifossulatum
Bembidion americanum
Bembidion carinula
Bembidion inaequale
Bembidion levettei carrianum
Bembidion lapponicum DNA1604 PCR
Bembidion lapponicum DNA3974 Illumina merged
Bembidion integrum
Bembidion honestum
Bembidion rufotinctum
Bembidion rothfelsi
Bembidion chalceum
Bembidion louisella
ML Bootstrap
Union of de novo, near ref, and far ref assemblies form one "Illumina merged" sequence
Topo

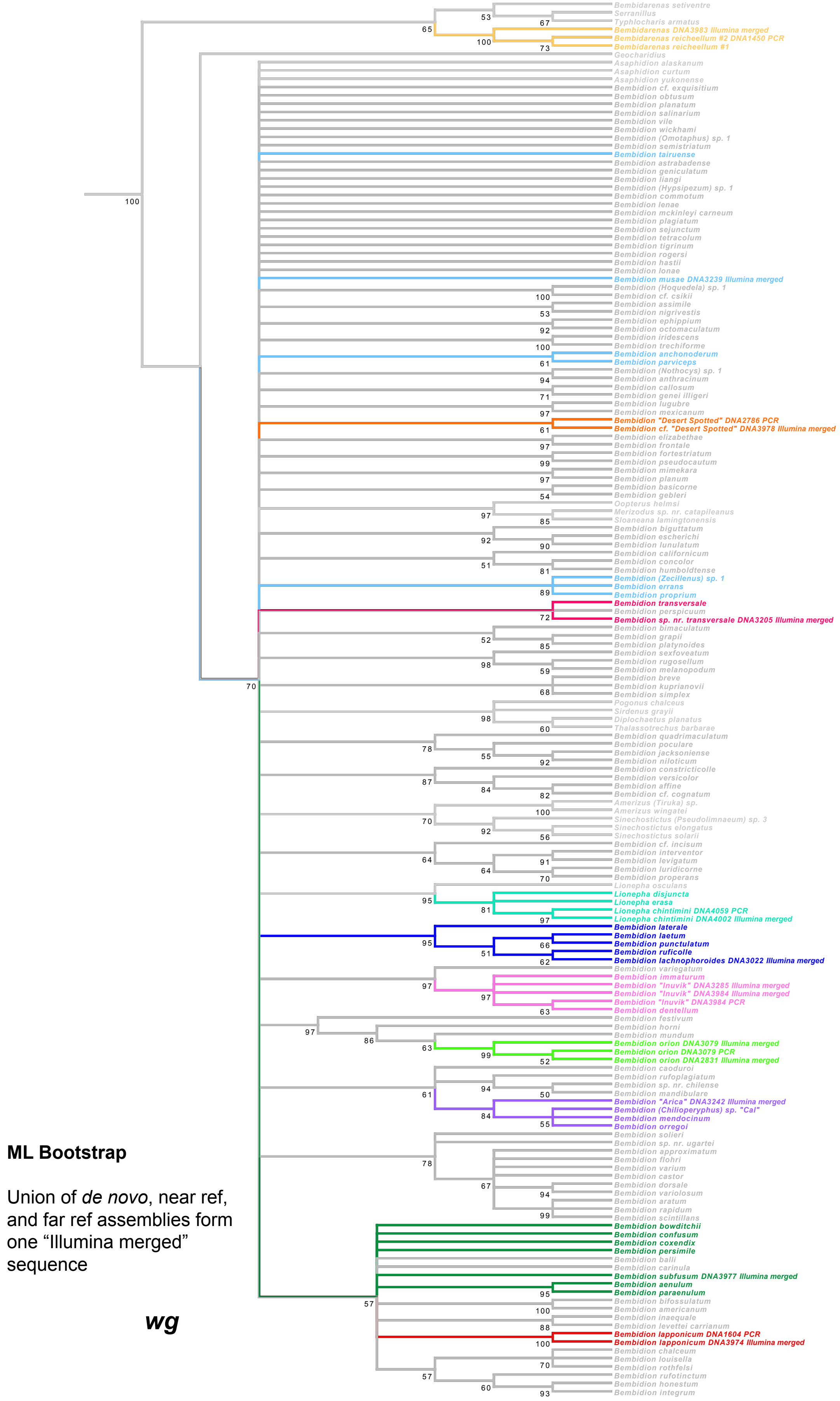
Bembidarenas setiventre
Serranillus
Typhlocharis armatus
Bembidarenas DNA3983 Illumina merged
Bembidarenas reicheellum #2 DNA1450 PCR
Bembidarenas reicheellum #1
Geocharidius
Asaphidion alaskanum
Asaphidion curtum
Asaphidion yukonense
Bembidion cf. exquisitium
Bembidion obtusum
Bembidion planatum
Bembidion salinarium
Bembidion vile
Bembidion wickhami
Bembidion (Omotaphus) sp. 1
Bembidion semistriatum
Bembidion tairuense
Bembidion astrabadense
Bembidion geniculatum
Bembidion liangi
Bembidion (Hypsipezum) sp. 1
Bembidion commotum
Bembidion lenae
Bembidion mckinleyi carneum
Bembidion plagiatum
Bembidion sejunctum
Bembidion tetracolum
Bembidion tigrinum
Bembidion rogersi
Bembidion hastii
Bembidion lonae
Bembidion musae DNA3239 Illumina merged
Bembidion (Hoquedela) sp. 1
Bembidion cf. csikii
Bembidion assimile
Bembidion nigrivestis
Bembidion ephippium
Bembidion octomaculatum
Bembidion iridescens
Bembidion trechiforme
Bembidion anchonoderum
Bembidion parviceps
Bembidion (Nothocys) sp. 1
Bembidion anthracinum
Bembidion callosum
Bembidion genei illigeri
Bembidion lugubre
Bembidion mexicanum
Bembidion "Desert Spotted" DNA2786 PCR
Bembidion cf. "Desert Spotted" DNA3978 Illumina merged
Bembidion elizabethae
Bembidion frontale
Bembidion fortestriatum
Bembidion pseudocautum
Bembidion mimekara
Bembidion planum
Bembidion basicorne
Bembidion gebleri
Oopterus helmsi
Merizodus sp. nr. catapileanus
Sloaneana tamingtonensis
Bembidion biguttatum
Bembidion escherichi
Bembidion lunulatum
Bembidion californicum
Bembidion concolor
Bembidion humboldtense
Bembidion (Zecillenus) sp. 1
Bembidion errans
Bembidion proprium
Bembidion transversale
Bembidion perspicuum
Bembidion sp. nr. transversale DNA3205 Illumina merged
Bembidion bimaculatum
Bembidion grapii
Bembidion platynoides
Bembidion sexfoveatum
Bembidion rugosellum
Bembidion melanopodum
Bembidion breve
Bembidion kuprianovii
Bembidion simplex
Pogonus chalceus
Sirdenus grayii
Diplochaetus planatus
Thalassotrechus barbarae
Bembidion quadrimaculatum
Bembidion poculare
Bembidion jacksoniense
Bembidion niloticum
Bembidion constricticolle
Bembidion versicolor
Bembidion affine
Bembidion cf. cognatum
Amerizus (Tiruka) sp.
Amerizus wingatei
Sinechostictus (Pseudolimnaeum) sp. 3
Sinechostictus elongatus
Sinechostictus solarii
Bembidion cf. incisum
Bembidion interventor
Bembidion levigatum
Bembidion luridicorne
Bembidion properans
Lionepha osculans
Lionepha disjuncta
Lionepha erasa
Lionepha chintimini DNA4059 PCR
Lionepha chintimini DNA4002 Illumina merged
Bembidion laterale
Bembidion laetum
Bembidion punctulatum
Bembidion ruficolle
Bembidion lachnophoroides DNA3022 Illumina merged
Bembidion variegatum
Bembidion immaturum
Bembidion "Inuvik" DNA3285 Illumina merged
Bembidion "Inuvik" DNA3984 Illumina merged
Bembidion "Inuvik" DNA3984 PCR
Bembidion dentellum
Bembidion festivum
Bembidion horni
Bembidion mundum
Bembidion orion DNA3079 Illumina merged
Bembidion orion DNA3079 PCR
Bembidion orion DNA2831 Illumina merged
Bembidion caduroi
Bembidion rufoplagiatum
Bembidion sp. nr. chilense
Bembidion mandibulare
Bembidion "Arica" DNA3242 Illumina merged
Bembidion (Chiliopheryphus) sp. "Cal"
Bembidion mendocinum
Bembidion orregoi
Bembidion solieri
Bembidion sp. nr. ugartei
Bembidion approximatum
Bembidion flohri
Bembidion varium
Bembidion castor
Bembidion dorsale
Bembidion variolosum
Bembidion aratum
Bembidion rapidum
Bembidion scintillans
Bembidion bowditchii
Bembidion confusum
Bembidion coxendix
Bembidion persimile
Bembidion balli
Bembidion carinula
Bembidion subfusum DNA3977 Illumina merged
Bembidion aenulum
Bembidion paraenulum
Bembidion bifossulatum
Bembidion americanum
Bembidion inaequale
Bembidion levettei carrianum
Bembidion lapponicum DNA1604 PCR
Bembidion lapponicum DNA3974 Illumina merged
Bembidion chalceum
Bembidion louisella
Bembidion rothfelsi
Bembidion rufotinctum
Bembidion honestum
Bembidion integrum
ML Bootstrap
Union of *de novo*, near ref, and far ref assemblies form one "Illumina merged" sequence
wg
