# Supplementary material for: Successful Recovery of Nuclear Protein-Coding Genes from Small Insects in Museums Using Illumina Sequencing
Source: PLoS One. 2015 Dec 30;10(12):e0143929. doi: 10.1371/journal.pone.0143929 (PMC4696846; doi:10.1371/journal.pone.0143929)
Supplement: S10 Fig — Values shown are the percent difference of the length of reference-based contig minus the length of the corresponding de novo contig. Positive values (blue) indicate the reference-base contig was longer, and negative values (red) indicate the de novo contig was longer. Gene fragments are ordered by average recovery as measured across both de novo and reference-based assemblies. Gene abbreviations are those used in Regier et al. [25]. Specimen abbreviations: Lag: Lagriinae n. gen. KK0290, subf: Bembidion subfusum 3977, snt1: B. sp. nr. transversale 3021, Lchi: Lionepha chintimini 4002, lach: B. lachnophoroides 3022, Bdrs: Bembidarenas 3983, ori1: B. orion 2831, inu1: B. "Inuvik" 3285, lapp: B. lapponicum 3974, aric: B. "Arica" 3242, dspt: B. cf. "Desert Spotted" 3978, mus: B. musae 3239, inu2: B. "Inuvik" 3984, ori2: B. orion 3079, snt2: B. sp. nr. transversale 3205. Four specimens with less than 34 million reads have specimen abbreviation and age shown in gray. (PDF) [file pone.0143929.s010.pdf]

	subf	snt1	Lchi	lach	bdrs	ori1	inu1	lapp	aric	dspt	mus	inu2	ori2	snt2	Average
8018fin1_2	0.00	0.00	0.77	0.00	0.32	-0.38	0.00	0.49	-0.71	0.57	0.00	0.29	-0.14	0.29	0.11
3044fin1_2	0.00	0.00	-0.48	-0.26	0.16	0.00	0.00	-0.08	-0.08	0.36	-0.45	0.16	0.00	-0.24	-0.06
8028fin1_2	0.00	0.00	-0.52	0.20	0.00	0.27	0.00	0.70	-0.02	0.56	0.29	-0.25	-0.04	0.42	0.10
197fin1_2	0.00	0.58	0.00	-0.55	0.00	-0.58	0.00	0.00	-0.51	0.24	0.00	-0.50	-0.21	0.00	-0.11
36fin1_2	0.15	0.28	0.18	-0.18	0.00	-0.05	0.35	0.00	-0.04	0.22	-0.05	0.21	0.23	0.33	0.09
aspec2_6	0.00	0.28	-0.41	0.00	0.00	-0.49	0.00	0.00	-0.45	0.30	0.30	-0.08	-1.00	0.00	-0.05
63fin2_3	0.00	0.26	0.18	-0.05	0.00	0.34	0.36	0.55	0.18	0.37	0.50	0.21	0.33	0.33	0.24
acc2_4	0.00	0.19	0.00	0.19	0.00	0.73	0.00	0.37	-0.37	0.00	0.00	-0.20	-0.15	0.32	0.08
25fin2_4	0.00	0.26	-0.01	-0.16	0.27	0.80	0.00	0.55	0.00	0.35	-0.05	0.68	0.65	0.98	0.22
270fin2_3	0.00	0.63	0.00	0.49	0.00	0.00	0.00	0.56	0.22	0.22	0.21	0.49	-0.11	0.00	0.23
42fin1_2	0.00	0.44	-0.26	-0.05	0.11	-0.14	0.00	0.16	-0.02	0.16	0.23	0.10	-0.13	0.07	0.06
44fin2_3	0.03	0.00	-0.07	0.14	0.17	0.98	0.00	0.16	0.29	0.57	0.50	0.88	0.98	1.00	0.30
3202fin1_3	0.00	0.62	0.20	0.18	0.00	0.22	0.19	0.28	0.36	0.00	0.19	-0.22	0.09	0.42	0.17
8029fin6_7	0.00	0.58	0.31	0.36	0.00	-0.01	0.00	0.39	-0.16	0.00	0.21	0.77	-0.14	0.43	0.20
3006fin1_2	0.00	0.38	-0.02	0.82	0.00	0.30	0.00	0.79	0.40	0.00	0.37	0.38	0.46	0.47	0.28
96fin1_3	0.00	0.06	0.28	0.17	0.00	0.13	0.00	0.31	-0.24	0.20	0.03	0.06	0.37	0.45	0.08
262fin1_2	0.21	0.70	0.41	0.61	0.16	0.17	0.00	0.18	0.09	-0.03	0.26	0.58	0.51	0.28	0.28
3007fin1_2	0.14	0.27	-0.08	-0.02	0.00	0.40	0.18	0.20	0.30	0.00	0.44	0.27	0.38	0.41	0.17
3152fin1_2	0.00	0.36	-0.15	0.22	0.00	0.34	0.00	0.32	-0.14	0.32	-0.28	-0.01	-0.15	0.00	0.08
8070fin1_3	0.03	0.39	-0.62	-0.21	0.00	-0.39	0.00	0.17	-0.05	0.74	0.20	0.00	-0.20	0.00	0.02
3012fin1_2	0.00	-0.10	-0.29	-0.55	0.00	0.28	0.18	0.65	-0.06	0.44	0.31	0.14	-0.18	0.00	0.08
40fin2_3	0.00	0.88	-0.16	-0.27	0.13	0.01	0.00	-0.12	-0.48	0.00	0.00	-0.43	-0.02	1.00	-0.04
3136fin1_2	0.02	0.63	0.05	0.71	0.11	0.87	0.18	0.45	0.20	0.39	0.41	0.86	0.99	1.00	0.41
113fin1_2	0.00	0.23	0.13	0.16	0.00	0.08	0.00	0.51	0.23	0.24	0.05	0.16	-0.16	0.68	0.15
58fin7_9	0.00	0.00	-0.13	-0.22	0.27	-0.03	0.00	0.41	-0.11	0.24	0.20	0.18	0.34	0.36	0.07
3064fin6_7	0.39	0.30	0.26	0.08	0.00	-0.18	0.17	0.60	0.15	0.14	-0.29	0.44	-0.38	0.25	0.17
3196fin5_6	0.13	0.34	-0.19	0.07	0.21	0.25	0.13	0.08	0.47	0.39	0.55	-0.10	-0.05	0.26	0.19
69fin2_3	0.03	-0.09	0.39	0.16	0.15	0.39	0.15	0.17	-0.08	0.13	-0.12	0.52	-0.01	0.18	0.15
58fin3_6	0.00	0.19	-0.05	0.41	0.00	-0.03	0.00	-0.30	-0.16	0.25	-0.17	0.01	0.33	0.41	0.01
62fin2_3	0.13	0.34	0.14	0.36	0.00	0.12	0.17	0.41	-0.16	0.51	0.48	-0.07	0.85	0.36	0.20
8053fin2_3	0.00	0.38	0.33	-0.02	0.00	0.12	0.00	0.26	0.15	0.36	0.38	0.52	0.19	-0.07	0.21
247fin1_2	0.00	0.41	0.36	-0.17	0.00	0.22	0.21	-0.07	-0.10	0.46	0.01	0.15	0.31	0.00	0.12
3114fin1_2	0.27	-0.08	0.26	0.39	0.00	-0.02	0.00	0.70	0.70	0.14	-0.19	0.05	-0.04	0.50	0.19
265fin2_3	0.00	0.01	0.00	-0.30	0.22	-0.05	0.00	0.74	-0.39	0.20	0.81	0.69	-0.06	-0.03	0.16
3121fin1_2	0.00	0.17	-0.24	0.13	0.23	0.05	0.18	0.28	-0.13	0.51	0.24	0.83	0.12	0.28	0.19
192fin1_2	0.00	0.23	-0.08	-0.01	0.00	-0.27	0.30	0.18	-0.32	0.71	0.22	0.42	-0.01	0.51	0.11
3094fin2_3	0.27	0.00	0.00	-0.13	0.22	0.23	0.21	-0.21	-0.22	0.36	0.48	-0.21	-0.07	0.00	0.08
8091fin1_2	0.00	0.49	-0.01	0.96	0.00	0.12	0.00	0.52	0.09	0.58	-0.06	0.01	0.06	0.10	0.23
166fin2_3	0.00	-0.05	0.25	0.28	0.00	0.70	0.53	0.30	-0.01	0.39	0.74	0.21	0.07	0.00	0.28
3017fin1_2	0.00	0.80	0.30	0.16	0.00	0.43	0.32	0.38	-0.30	0.45	0.49	0.25	0.43	0.53	0.27
149fin2_3	0.09	0.75	0.16	0.26	0.00	-0.28	0.00	0.38	-0.55	0.25	0.11	-0.05	-0.07	0.00	0.09
aspec11_12	0.00	0.33	-0.22	-0.35	0.00	-0.28	0.00	-0.14	-0.35	0.22	0.35	-0.25	-0.29	0.00	-0.06
220fin1_2	0.00	0.20	-0.05	0.15	0.16	-0.07	0.00	-0.10	-0.02	0.37	0.08	0.91	0.30	0.31	0.14
3031fin1_3	0.19	-0.02	-0.02	0.80	0.13	-0.06	0.15	0.52	0.68	0.60	-0.06	0.31	0.29	1.00	0.27
3066fin1_3	0.00	0.14	0.26	-0.06	0.18	0.04	0.08	0.47	0.28	0.25	0.68	0.47	0.50	0.00	0.23
EF-1α	0.18	0.82	0.43	0.45	0.32	0.98	0.31	0.31	0.97	0.66	0.45	0.52	0.98	1.00	0.53
2F3_4	0.00	-0.09	0.36	-0.03	0.00	-0.04	0.17	-0.16	-0.01	0.35	-0.12	-0.22	0.55	0.00	0.02
268fin1_2	0.02	0.40	0.17	0.00	0.00	0.17	0.02	0.29	-0.11	0.32	0.18	0.09	0.35	0.41	0.13
aspec19_21	0.23	0.00	-0.30	-0.09	0.00	-0.03	0.40	0.33	0.35	0.20	0.70	-0.03	-0.03	0.43	0.15
3153fin1_2	0.00	0.62	0.15	0.00	0.00	-0.26	0.16	-0.21	-0.08	0.29	0.23	0.06	-0.47	0.13	0.08
274fin1_2	0.00	0.49	0.08	-0.10	0.00	-0.15	0.00	-0.02	-0.53	0.39	-0.06	0.13	-0.01	0.47	0.02
109fin1_2	0.00	0.57	0.10	-0.06	0.00	0.33	0.21	0.40	-0.29	0.46	-0.34	0.49	-0.06	0.46	0.16
3196fin1_3	0.00	0.52	0.09	0.35	0.17	-0.07	0.23	0.65	-0.34	0.00	-0.34	-0.26	-0.02	0.59	0.08
3070fin4_5	0.13	0.33	-0.76	-0.14	0.00	-0.33	0.00	-0.25	-0.47	0.16	-0.49	-0.07	-0.39	0.00	-0.16
3031fin4_5	0.00	0.00	0.00	-0.05	0.00	1.00	0.00	0.48	0.82	0.86	0.32	0.99	1.00	1.00	0.37
73fin2_3	0.00	0.12	0.10	0.16	0.00	-0.06	0.14	0.14	0.03	0.72	0.05	-0.02	-0.02	0.00	0.11
EF-2	0.07	0.32	0.12	0.11	0.15	0.43	0.20	0.29	0.21	0.30	0.21	0.75	0.78	0.48	0.26
127fin1_2	0.00	0.74	-0.35	-0.01	0.00	0.00	0.20	0.74	0.03	0.11	0.58	-0.02	-0.05	0.00	0.17
6fin2_3	0.00	0.56	-0.39	-0.06	0.00	0.83	0.51	0.20	-0.10	0.46	0.36	0.82	-0.06	0.00	0.27
2F7_8	0.00	0.47	-0.34	0.10	0.33	-0.05	0.15	0.04	-0.30	0.52	0.43	-0.23	-0.08	0.00	0.09
26fin3_4	0.00	0.11	-0.01	0.15	0.28	0.06	0.31	0.56	0.00	0.49	-0.20	-0.04	0.45	0.49	0.14
3009fin2_3	0.04	-0.01	-0.02	-0.13	0.00	-0.12	0.00	0.00	-0.79	0.55	-0.29	-0.05	0.32	0.33	-0.07
226fin1_2	0.17	0.09	-0.23	-0.30	0.00	-0.11	0.16	0.64	-0.01	0.68	0.59	-0.03	-0.02	0.00	0.14
PolII	0.00	0.27	0.36	0.10	0.18	0.01	0.32	0.05	-0.02	0.35	0.37	0.03	0.19	0.00	0.17
3055fin2_3	0.36	0.75	0.09	0.00	0.00	0.00	0.00	0.00	0.00	0.54	0.58	-0.07	0.00	0.00	0.19
3059fin1_3	0.00	0.41	-0.09	0.10	0.15	-0.03	0.16	0.03	-0.02	0.40	-0.06	-0.13	-0.01	0.00	0.08
3089fin1_3	0.00	0.37	-0.14	-0.26	0.00	-0.07	0.63	-0.01	-0.10	0.00	0.71	-0.03	-0.05	0.00	0.09
**Average**	0.05	0.31	0.01	0.08	0.07	0.12	0.12	0.26	-0.03	0.34	0.19	0.19	0.13	0.29	0.14
**Number <0**	0	7	31	29	1	31	0	12	42	1	18	25	35	7	
**Fraction <0**	0	0.104	0.463	0.433	0.015	0.463	0.000	0.179	0.627	0.015	0.269	0.373	0.522	0.104	
