# Supplementary material for: Successful Recovery of Nuclear Protein-Coding Genes from Small Insects in Museums Using Illumina Sequencing
Source: PLoS One. 2015 Dec 30;10(12):e0143929. doi: 10.1371/journal.pone.0143929 (PMC4696846; doi:10.1371/journal.pone.0143929)
Supplement: S1 Methods — (DOCX) [file pone.0143929.s011.docx]

# Supplemental Methods

# Library preparation details

We used the DNA quality metrics described in the section “Assessing DNA quality of museum and reference specimens“ to inform our library preparation procedures for each DNA extraction (**Table 7**).

Category 1 extractions were prepared using the TruSeq ChIP Sample Prep Kit (Illumina). This kit is better optimized for lower starting amounts of DNA and shorter fragment length than the TruSeq Nano DNA or TruSeq DNA Sample Prep Kits (Illumina). We initially attempted to prepare the library for Lagriinae n. gen. KK0290 with the TruSeq DNA Sample Prep Kit due to its relatively high concentration of DNA (1700 ng). However, the sample failed to yield sufficient library product, and the library was redone using the TruSeq ChIP Prep Kit. No DNA in extractions from category 1 was sheared prior to library prep since natural degradation had already fragmented the DNA, and shearing would have resulted in loss of fragments at the lower end of the size distribution.

Category 2 and 3 extractions were prepared using the TruSeq DNA Sample Prep Kit (Illumina) and Apollo 324 NGS Prep System with the PrepX ILM DNA Library Kit (Wafergen). This protocol did not produce a usable library for *Bembidion* “Inuvik” 3285. The sequenced library for this sample was prepared using the remaining DNA and the TruSeq ChIP Sample Prep Kit. As with category 1, we did not shear DNA prior to library preparation for category 2 and 3 extractions.

Extractions in category 4 had high concentrations of both short and long DNA fragments. To obtain a uniform fragment size in the final libraries, we ran these DNA extractions on an agarose gel and excised bands representing longer (>350 bases) and shorter (150-350 bases) fragments.

Category 5 extractions contained high concentrations of longer fragments. We sheared these extractions using a Bioruptor® Pico Sonication System (Diagenode) with 10 cycles of 1 minute each (30 seconds on, 30 seconds off). Libraries were prepared with the Apollo 324 NGS Library Prep System using PrepX ILM DNA Library Kits (Wafergen).

We prepared libraries for reference specimens *Bembidion orion* DNA3079 and *Bembidion* sp. nr. *transversale* 3205 using the TruSeq DNA Sample Prep Kit. Prior to library preparation, we sheared DNA to approximately 300 base fragments using Bioruptor® Pico Sonication System (Diagenode) with 10 cycles of 1 minute each (30 seconds on, 30 seconds off).

# Query and reference sequences for 67 nuclear protein-coding gene fragments

To determine which of the Regier et al. [1] gene fragments were present in a *de novo* assembly, we began by creating a BLAST database of reads for each museum specimen. One fragment used in the original study, 267fin2_3, was entirely contained within one of the genes from our seven focal gene set, CAD, and was excluded from this analysis. We then queried each database using a reference sequence for each of the 67 Regier et al. targets. Because the Regier et al. study did not include any taxa that are closely related to our museum specimens (for example, no beetles were included in the original study), we first generated reference sequences for each target fragment from *Bembidion* sp. nr. *transversale* and *Tribolium castaneum*, in order to have query sequences that were likely to produce high scoring BLAST hits.

To identify orthologous sequences of the 67 gene fragments in *Bembidion* sp. nr. *transversale* and *Tribolium castaneum*, we acquired reference sequences for the 67 Regier et al. fragments from insect sequences on GenBank. As no single taxon had sequences available for all 67 fragments, it was necessary to select two representative insect species. The best available representative taxa were an earwig, *Forficula auricularia*, and a moth, *Cydia pomonella*, which together had representative sequences of all genes. *F. auricularia* was selected due its having 66 of the target fragments at least partially represented. Although *C. pomonella* was not used in the original Regier et al. [1] study, orthologous fragments were available on GenBank, serving as a backup in the event that the earwig sequence failed to recover a putative ortholog in our beetle genomic sequences.

We used each earwig or moth fragment as the query of a BLASTn [2] search (match/mismatch scoring 2 – 3, word size 11, open/extend gap cost 5 – 2, max e-value 1e-1) against a BLAST database of contigs from the *de novo* assembly of *Bembidion* sp. nr. *transversale* 3205 within Geneious v6.1.4 [3]. Putative orthologs were extracted if they were a convincing match for the query gene. A convincing match was defined as having a low e-value (<1e-50), no similar hits with low e-values that might indicate paralogs, and an amino acid sequence with no internal stop codons. The query earwig or moth sequences were constructed from RNA sequences, however, the *B.* sp. nr. *transversale* contigs were generated from genomes, thus introns were frequently encountered. We identified intron-exon boundaries by aligning *B.* sp. nr. *transversale* genome loci with corresponding loci from an unpublished *B*. sp. nr. *transversale* transcriptome assembly using MAFFT.

After extracting orthologs of the 67 gene fragments from *B.* sp. nr. *transversale*, each was aligned to the reference earwig query using MAFFT. For some loci, this revealed missing data in the *B.* sp. nr. *transversale* sequences. If this occurred, missing sequence data were filled in using the *B.* sp. nr. *transversale* transcriptome. Finally, the innermost primer sites (as indicated in [1] Table 2, column labeled “gene region”) were located and annotated. Primer sequences, which were taken from Appendix 1 of the same study, were found using the *Search* feature within Geneious. The final locus was labeled with the appropriate gene and locus name (taken from [1], Appendix 1) and retained to be used as BLAST queries against the databases HTS museum specimen data.

The Lagriinae n. gen. *de novo* assembly was queried using sequences from *Tribolium castaneum*. Query sequences were generated by conducting a BLAST search of a database of *T. castaneum* cDNA sequences [4] with the previously generated *B.* sp. nr. *transversale* sequences as the query. *T. castaneum* sequences were trimmed to match the start and end of the *B.* sp. nr. *transversale* sequences. We were unable to recover two gene fragments in *T. castaneum*, proteasome subunit (262fin1_2) and H transporting ATPase (96fin1_3)*.*

# Query and reference sequences for seven focal genes

### Query sequences

As with the 67-gene fragment set, query sequences for 28S, 18S, COI, ArgK, CAD, Topo, and *wg* were generated from the *de novo* assembly of *Bembidion* sp. nr. *transversale* 3205 or published *Tribolium castaneum* sequences.

Focal gene sequences for *Bembidion* sp. nr. *transversale* 3205 were obtained by BLAST searching of the *de novo* assembly using as queries sequences from GenBank of *Bembidion transversale* specimen number 2157 [5].

*T. castaneum* query sequences for ArgK, CAD, *wg*, and COI were generated by trimming GenBank sequences (XM008195213, XM967097, XM008194940, AJ312413) at the start and end of the outermost PCR primer sequences used to amplify target gene fragments (see below). For 18S and 28S query sequences, we used the untrimmed GenBank accessions HM156711 and HM156703. We did not generate a query sequence for Topo from *T. castaneum* since this gene was not used in the phylogenetic analyses of Lagriinae.

### Selection of taxa for reference-based assemblies

For all carabids, we used *Asaphidion yukonense,* a distantly related trechite carabid, as a reference, which we refer to as the “far reference”. *A. yukonense* is expected to be distantly but equally related to all *Bembidion* analyzed, somewhat more distantly related to *Lionepha chintimini*, and quite distantly related to *Bembidarenas* [6]. As a counterpart to this far reference, we chose a “near-reference” that varied among HTS specimens. Near references were chosen as species closely related to, but likely distinct from, the museum specimens, based upon the work by [6,7]. Reference sequences came from the same two studies.

The near references used were *Bembidion paraenulum* Maddison (specimen 1856) for *B. subfusum*; *Bembidion lugubre* (specimen 1712) for *B.* sp. nr. *transversale* and *B.* cf “Desert Spotted”; *Lionepha disjuncta* (specimen 1896) for *Lionepha chintimini*; *Bembidion punctulatum* (specimen 1713) for *B. lachnophoroides*; *Bembidion cooperi* Maddison (specimen 2133, sequences from [7]) for *Bembidion orion*; *Bembidion dentellum* (specimen 1714) for *Bembidion* “Inuvik”; *Bembidion levettei carrianum* (specimen 0108) for *Bembidion lapponicum*; *Bembidion mendocinum* (specimen 2333, sequences from [8]) for *Bembidion* “Arica”; and *Bembidion tairuense* (specimen 0607) for *Bembidion musae*. Sequences are from sources indicated in [6] unless otherwise stated. For *Bembidarenas*, two different near references from [6] were used: a near reference of *Bembidarenas setiventre* (specimen 2214), and a very near reference of *Bembidarenas reicheellum* #1 (specimen 2213). Our unpublished data suggests that *Bembidarenas setiventre* is quite distantly related to *B. reicheellum* #2, and likely not congeneric, whereas *Bembididarenas reicheellum* #1 is possibly (although unlikely) conspecific. Although one is more distant than our typical near references, and one to possibly too closely related, they are the most appropriate species known.

To simplify comparison among sequences from different species, which were initially of varying length, all reference fragments for a gene were aligned using MAFFT (with the L-INS-i algorithm and default parameter values), and a region of the alignment with overlapping DNA sequence from all the reference taxa was selected.

# Additional details of phylogenetic tests

Here we provide details of sequence alignment and phylogenetic methods used in the phylogenetic tests of sequence accuracy.

## Tenebrionidae

Alignment was not difficult for COI since there were no insertions or deletions. ArgK did not have any indels except for a 49-base intron in *Praeteus fuscus* and a 55-base intron in *Rhypasma* sp., which we excluded from phylogenetic analyses. CAD and *wg* exhibited numerous indels, including a 33-base intron in *Enicmosoma decorsei*. Introns were deleted prior to alignment. We aligned amino acid translations of CAD and *wg* genes using MAFFT v7.130b [9,10] and the L-INS-i algorithm with default parameter values. We then shifted nucleotide sequences to match the amino acid alignment using Mesquite [11]. 28S and 18S were also aligned using MAFFT. We identified and deleted ambiguously aligned regions using the server version of GBlocks 0.91b [12,13] with all options for less stringent block selection chosen.

We selected optimal data partitions and models of nucleotide evolution for individual protein-coding genes using PartitionFinder v1.1.1 [14] from initial partition schemes based on codon position. We restricted models to only those available in RAxML and compared them using the Bayesian Information Criterion (BIC). Nucleotide substitution models for the ribosomal genes were chosen with jModelTest 2.0 [15,16] using BIC to identify the optimal model. For all protein-coding genes, the optimal partitioning scheme had first and second codon position in one partition and third codon positions in a second partition. Among the models implemented in RAxML, GTR+G was identified as the optimal model for third codon positions of COI and *wg*, and GTR+I+G was identified as the optimal model for all other partitions.

We also combined the six genes into a concatenated matrix using Mesquite [11], and chose optimal data partitions and models of nucleotide evolution starting with a partition scheme based on genes and codons using PartitionFinder v1.1.1 [14]. The optimal partition scheme for the concatenated dataset had first and second codon positions of all protein-coding genes and 18S in one partition, third codon positions of ArgK and *wg* in a second partition, third codon positions of CAD and COI in a third partition, and 28S in a fourth partition. GTR+I+G was identified as the optimal model for all partitions.

We conducted Maximum Likelihood analyses on the single gene and concatenated datasets using RAxML v. 8.0.3 [17] using the optimal partitioning scheme and model of nucleotide evolution previously identified with the BIC when possible. COI and *wg* recovered different models for each of their two partitions. Since RAxML v. 8.0.3 can only accommodate a single model across partitions, we chose the more complex of the two (GTR+I+G) for both partitions. We analyzed each dataset with 500 independent search replicates and 1,000 bootstrap replicates.

## Carabidae

### Modifications to the carabid matrices based upon updated voucher identifications

The two specimens that were sequenced for *“B. umbratum”* in [6] belong to two different species: DNA voucher 2166 is *B. umbratum* and specimen 1757 belongs to *B. variolosum* Motschulsky. Thus, sequences of 28S, ArgK, Topoisomerase, and COI in [6] were from true *B. umbratum*, and the remaining genes from *B. variolosum*. 28S, ArgK, Topoisomerase, and COI were obtained for *B. variolosum* specimen 1757, and in our analyses the *B. umbratum* sequences are not included. There are three additional nomenclatorial changes required from the names used in [6]: (1) the specimen (voucher 1409) identified as *B. (Semicampa) muscicola* Hayward in [6] is actually a specimen of *B. nigrivestis* Bousquet; (2) the species called “*Merizodus* sp. nr. *catapileanus*” is not close to *catapileanus* Jeannel, and is called here by the informal name *Merizodus* “Valdivia”; (3) the species called *Bembidarenas reicheellum* is herein called *Bembidion reicheellum* #1, as there appear to be at least two cryptic species hiding within the concept “*Bembidarenas reicheellum*”. Museum specimen *Bembidarenas* 3983 is likely conspecific with the species herein called *Bembidarenas reicheellum* #2.

### Alignment and phylogenetic analyses

Alignment methods varied among the genes. For protein-coding genes, there were either a few, non-overlapping insertions of one or two amino acids in a very few taxa that could thus be easily aligned (*wg*) or there were no insertion and deletions evident in the analyzed sequences (remaining protein-coding genes). We aligned ribosomal genes in MAFFT version 7.130b [9,10] using the L-INS-i search option and default parameters. Regions with less certain alignment were excluded using GBlocks, as described in the preceding section.

A total of 23 matrices were analyzed: three matrices (“All Contigs”, “Three Separate”, and “Illumina Merged”) for each of the seven genes and two matrices concatenated matrices, one a multigene matrix formed by concatenation of the “Three Separate” matrices, and the other from the “Illumina Merged” matrices.

For individual gene analyses, the data were not partitioned. For multi-gene matrices, the data were subject to PartitionFinder 1.1.1 [14] analyses, with the beginning partition separating each ribosomal gene in its own part, and each codon of each protein-coding gene in its own part. The optimal partition (chosen by BIC) for both multi-gene matrices had third positions of COI in one part, third positions of all nuclear protein-coding genes in a another part, and all remaining sites in the last part, with the optimal single model (among those implemented by RAxML) being GTR+I+G.

Maximum likelihood analyses were conducted on the single gene and concatenated datasets using RAxML v. 8.0.3 [17,18]. Searches for the maximum likelihood tree were conducted using 200 search replicates; maximum likelihood bootstrap support values were obtained using 1000 bootstrap replicates.

# PCR protocols: HTS museum specimens

The following protocols were used to amplify DNA fragments in museum specimens. Refer to S2 Table for details about primers.

**28S:** We developed primers to amplify a 360-365 base and 650-750 base fragment of 28S. Both fragments are nested within the longer fragment amplified from fresh specimens. The 360-365 base fragment was amplified with primer pair D1 and LS1R **(S2 Table**). The longer fragment was amplified with primer pair LS1F and D3L. Both fragments were amplified using the thermocycling protocol C1 from Table A1 of [6].

**COI:** We used the DNA barcoding primers LCO1490 and HCO2198 [18] with the following thermocycler profile: two minutes at 94°C, then five cycles of 60 seconds at 94°C, 90 seconds of annealing at 45°C, and 90 seconds of extension at 72°C, followed by 35 equivalent cycles except with an annealing temperature of 50°C.

***wg*:** We used the same protocol as described in the following section.

# PCR protocols: Additional specimens for phylogenetic analyses

Additional carabid gene fragments were amplified using protocols described in [6]. The following PCR protocols were used to amplify gene fragments in Tenebrionidae and yield sequences for the majority of the family. Unless otherwise noted, sequencing primers are the same as amplification primers. Refer to table S6 Table for cycling protocol and S2 Table for details about primers.

**18S:** Use primer pair 518S and S1893R with cycler profile ST-50-C. Aliquot PCR product into three equal samples and sequence using 518S, S1893R, SS398F, SS1054F, SS1090R, and SS1554R.

**28S:** Use primers NLF184 and D3aR with cycler profile ST-52-B.  If this does not result in any bands or two closely spaced bands, use primers D1 and D3 with cycler profile ST-52-B. If both protocols fail and the extraction was not from fresh tissue, use the protocols described in section “PCR Protocols: HTS museum specimens” for amplifying short fragments of 28S.

**COI**: Use primers LCO1490 and HCO2198 with cycler profile ST-52-B.

**ArgK:** Use primers AK168F and AK939R with cycler profile ST-57-C. Use the PCR product of the first reaction as template in a nested reaction using primers AK183F and AK933R. The extension time is kept long to deal with the occasional long introns that are present throughout Tenebrionidae.  The extension time can be reduced to one minute if there are no introns.

**CAD:** Use primers CD439F and CD1098R2 with cycler profile TD-60-O. Use the PCR product of the first reaction as template in three separate nested PCRs using primer pairs CD439F/CD668R, CD667F/CD828R, and CD806F/CD1098R2. Use cycler profile ST-55-B for all of these reactions.

Depending on quality of the starting DNA and also freshness of reagents, amplification of the outer fragment may fail. If the previous protocol fails, the three regions may be amplified and sequenced individually. For the first piece of CAD, generate a template using primers CD338F and CD688R using cycler profile TD-60-M.  Use the PCR product of the first reaction as template in any forward/reverse combination of CD338F, CD439F, CD668R, and CD688R using cycler profile ST-55-B.

For the second piece, generate a template using primers CD660F and CD858R using cycler profile TD-60-M.  Use the PCR product of the first reaction as template in any forward/reverse combination of CD660F, CD667F, CD828R, and CD858R using cycler profile ST-55-B.

For the third piece, generate a template using primers CD806F and CD1098R2 using cycler profile TD-60-M.  Use the PCR product of the first reaction as template in a nested reaction using primers CD821F and CD1098R2 using cycler profile ST-55-B.

***wg*:** Use primers wg550F and wgABRZ with cycler profile ST-54-D.  Use the PCR product of the first reaction as template in a nested reaction using primers pair wg578F and wgABR with cycler profile ST-54-C. While the outer reaction will often produce a bright band, the outer primers sometimes do not sequence the target fragment well in Tenebrionidae, often resulting in sequencing of a paralog.

# References

1. Regier JC, Shultz JW, Ganley ARD, Hussey A, Shi D, et al. (2008) Resolving Arthropod Phylogeny: Exploring Phylogenetic Signal within 41 kb of Protein-Coding Nuclear Gene Sequence. Systematic Biology 57: 920-938.

2. Altschull SF, Gish W, Miller W, Myers EW, Lipman DJ (1990) Basic local alignment search tool. J Mol Biol 215: 403-410.

3. Kearse M, Moir R, Wilson A, Stones-Havas S, Cheung M, et al. (2012) Geneious Basic: an integrated and extendable desktop software platform for the organization and analysis of sequence data. Bioinformatics 28: 1647-1649.

4. Richards S, Gibbs RA, Weinstock GM, Brown SJ, Denell R, et al. (2008) The genome of the model beetle and pest Tribolium castaneum. Nature 452: 949-955.

5. Wild AL, Maddison DR (2008) Evaluating nuclear protein-coding genes for phylogenetic utility in beetles. Molecular Phylogenetics and Evolution 48: 877-891.

6. Maddison DR (2012) Phylogeny of *Bembidion* and related ground beetles (Coleoptera: Carabidae: Trechinae: Bembidiini: Bembidiina). Molecular Phylogenetics and Evolution 63: 533-576.

7. Maddison DR, Cooper KW (2014) Species delimitation in the ground beetle subgenus Liocosmius (Coleoptera: Carabidae: Bembidion), including standard and next-generation sequencing of museum specimens. Zoological Journal of the Linnean Society 172: 741-770.

8. Maddison DR, Toledano L, Sallenave S, Roig-Junent S (2013) Phylogenetic relationships of the South American ground beetle subgenus *Chilioperyphus* Jeannel (Coleoptera: Carabidae: Trechinae: Bembidiini: *Bembidion* Latreille). Zootaxa 3636: 547-560.

9. Katoh K, Kuma K, Toh H, Miyata T (2005) MAFFT version 5: improvement in accuracy of multiple sequence alignment. Nucleic Acids Research 33: 511-518.

10. Katoh K, Standley DM (2013) MAFFT multiple sequence alignment software version 7: improvements in performance and usability. Mol Biol Evol 30: 772-780.

11. Maddison WP, Maddison DR (2014) Mesquite: a modular system for evolutionary analysis. Version 3.0. <http://mesquiteproject.org>.

12. Castresana J (2000) Selection of conserved blocks from multiple alignments for their use in phylogenetic analysis. Molecular Biology and Evolution 17: 540-552.

13. Talavera G, Castresana J (2007) Improvement of phylogenies after removing divergent and ambiguously aligned blocks from protein sequence alignments. Systematic Biology 56: 564-577.

14. Lanfear R, Calcott B, Ho SYW, Guindon S (2012) PartitionFinder: combined selection of partitioning schemes and substitution models for phylogenetic analyses. Molecular Biology and Evolution 29: 1695-1701.

15. Darriba D, Taboada GL, Doallo R, Posada D (2012) jModelTest 2: more models, new heuristics and parallel computing. Nature Methods 9: 772.

16. Guindon S, Gascuel O (2003) A simple, fast, and accurate algorithm to estimate large phylogenies by maximum likelihood. Systematic Biology 52: 696-704.

17. Stamatakis A (2014) RAxML Version 8: A tool for Phylogenetic Analysis and Post-Analysis of Large Phylogenies. Bioinformatics.

18. Hebert PDN, Cywinska A, Ball SL, DeWaard JR (2003) Biological identifications through DNA barcodes. Proceedings of the Royal Society of London Series B-Biological Sciences 270: 313-321.
