# Supplementary material for: Successful Recovery of Nuclear Protein-Coding Genes from Small Insects in Museums Using Illumina Sequencing
Source: PLoS One. 2015 Dec 30;10(12):e0143929. doi: 10.1371/journal.pone.0143929 (PMC4696846; doi:10.1371/journal.pone.0143929)
Supplement: S1 Table — (DOCX) [file pone.0143929.s012.docx]

**S1 Table. Additional details of specimen provenance and extracted tissue.**

| **Taxon** | **Sample** | **Repository** | **Extracted tissue** | **Locality Data** |
| --- | --- | --- | --- | --- |
| Lagriinae n. gen. | KKDNA0290 | MCZ, EMEC, CAS | hind body | Colombia: N. W. Sierra N. de Sta. Marta |
| *Bembidion subfusum* | DNA3977 | MCZ | whole body | Philippines: Central Plains of Luzon Island |
| *Bembidion* sp. nr. *transversale* | DNA3021 | OSAC | whole body | USA: Idaho: Cassia Co., Sublett Res. |
| *Lionepha chintimini* | DNA4002 | OSAC | whole body | Canada: BC: 12 mi. Creek, Monashee Rd., Cherryville |
| *Bembidion lachnophoroides* | DNA3022 | UASM | whole body | Canada: Alberta: Bull's Head Creek 3 mi. E Medicine Hat. |
| *Bembidarenas* | DNA3983 | MCZ | whole body | Chile: vicinity Punta Arenas |
| *Bembidion orion* | DNA2831 | KWC | whole body | USA: California, Oakhurst, Big Creek. |
| *Bembidion* "Inuvik" | DNA3285 | DRM, UAIC, OSAC | whole body | Canada: NWT: Campbell Creek at route 8 |
| *Bembidion lapponicum* | DNA3974 | DRM, UAIC, OSAC | whole body | Canada: Saskatchewan: North Saskatchewan River at Deer Creek Bridge on highway 3 |
| *Bembidion* "Arica" | DNA3242 | CMNH | whole body,  except abdomen | Chile: Region I, Arica Prov., 7.5 km ENE Molinos, Rio Lluta |
| *Bembidion cf.* "Desert Spotted" | DNA3978 | KK, OSAC | whole body | USA: Utah: Glen Canyon, Escalante |
| *Bembidion musae* | DNA3239 | CMNH | whole body | New Zealand: South Island, Marlborough Province. Clarence, Clarence River |
| *Bembidion* "Inuvik" | DNA3984 | UAM | whole body | USA: Alaska: Selawik NWR, Kugarak River |
| *Bembidion orion* | DNA3079 | OSAC | male accessory  glands, testes | USA: California: El Dorado Co., Strawberry Creek at Sciots Camp |
| *Bembidion* sp. nr. *transversale* | DNA3205 | OSAC | whole abdomen | USA: Oregon: Benton Co., Corvallis, Willamette River |
| *Bembidion subfusum* | DNA2494 | MCZ | whole body | Philippines: Central Plains of Luzon Island |
| *Bembidion subfusum* | DNA1955 | MCZ | whole body | Philippines: Central Plains of Luzon Island |
| *Bembidarenas reicheellum* | DNA3973 | MCZ | whole body | Chile: 52°S. Rio Rubens |
| *Apteromimus platyderoides* | DNA3959 | MRCA | whole body | Sainte-Hélène: Centre High Central Ridge, Cabbage Tree Road |
| *Pseudophilochthus nubigena* | DNA3957 | MRCA | whole body | Sainte-Hélène: Centre High Central Ridge, Cabbage Tree Road |
| *Moirainpa amazona* | DNA3907 | USNM | whole body | Brazil: Amazonas: Manaus vicinity, Rio Solimões, Curari Island |
| *Tachysbembix* sp. | DNA3908 | USNM | whole body | Columbia: Magdalena: Lago Torno |
| *Bembidion* "Clearwater" | DNA2907 | DRM, UAIC, OSAC | whole body | USA: Florida: Pinellas Co., Clearwater |
| *Bembidion tencenti* | DNA3286 | UASM | whole body | Canada: Saskatchewan: Rosefield (s.e. of Val Marie) |
| *Bembidion* "Arica" | DNA3975 | CMNH | whole body | Chile: Region I, Arica Prov., 7.5 km ENE Molinos, Rio Lluta |
| *Bembidion* sp. nr. *germainianum* | DNA3976 | CMNH | whole body | Bolivia: Santa Cruz Prov. Andres Ibanez Santa Cruz de la Sierra, Rio Pirai |
| *Bembidion (Asioperyphus)* sp. | DNA4003 | CAS | whole body | USA: Alaska: Homer, Bishops Beach |
| *Chaetyllus* n. sp. 1 | KKDNA0280 | USNM | whole body | Ecuador: Orellana, Res. Ethnica Waorani, 1km S. Onkone Gare Camp |
| *Chaetyllus* n. sp. 11 | KKDNA0278 | SEMC | whole body | Venezuela: Lara, Sanare, 6.4 km SE |
| *Bembidion nesophilum* | DNA3240 | CMNH | whole body | New Zealand: North Island, Northland. Rarawa Beach |
| *Bembidion* "Kenosha Pass" | DNA4004 | CAS | whole body | Kenosha Pass, Colo., 10000 ft. vii.21.39 J.W. Green |
| *Bembidion sarpedon* | DNA2463 | OSAC | whole body | USA: Wyoming: Yellowstone National Park, Mammoth Hot Springs |
| *Lionepha casta* | DNA4005 | OSAC | whole body | ORE: Lostine R., Wallowa Co., Aug 17, 1952. F.C. French |
| *Bembidion (Notaphus)* "Sinaloa" | DNA3971 | UASM | whole body | Mexico: Sinaloa. nr. El Cameron, n. Mazatlan, nr sea beach 20 August 1962. |
| *Bembidion* "Talus" | DNA4006 | CAS | whole body | Grand Cyn. Ariz. 21 June 63. South Rim. damp rock, Br. Angel Tr. J.G. Edwards accession. |
| *Bembidion (Notaphus)* "SLP" | DNA3972 | UASM | whole body | Mexico: San Luis Potosi, Rio Anojoque, Tamazunchale, 400' 31 October 1965 |
| *Pseudophilochthus rufosuffusum* | DNA3960 | MRCA | whole body | Sainte-Hélène: Centre High Central Ridge, replat S.E. Diana's Peak |
| *Bembidion rufinum* | DNA4007 | CAS | whole body | Idaho: Nez Perce Co., 6 mi W Lenore, Clearwater River. 19 July 1970. D.H. Kavanaugh, H. Goulet collectors |
| *Bembidion* "Red River" | DNA4008 | CAS | whole body | USA: Colorado: Huerfano Co., Sangre de Cristo Range, 5 mi S. LaVeda, hwy 111, Cucharas R., 7600', 13 June 1971. D.H. Kavanaugh, E.A. Martinko. |
| *Bembidion orion* | DNA2826 | KWC | whole abdomen,  leg | USA: California: Alpine Col, 2.9 mi SW Silvercreek Campground. |
| *Chaetyllus* n. sp. 1 | KKDNA0285 | USNM | whole body | Peru: Loreto; Cocha Shinguito, Rio Samira |

**Repository**: codens of the museums in which specimens were housed prior to extraction. If multiple codens are listed a cell, that specimen moved from museum to museum during its history.
