# Supplementary material for: Successful Recovery of Nuclear Protein-Coding Genes from Small Insects in Museums Using Illumina Sequencing
Source: PLoS One. 2015 Dec 30;10(12):e0143929. doi: 10.1371/journal.pone.0143929 (PMC4696846; doi:10.1371/journal.pone.0143929)
Supplement: S2 Table — (DOCX) [file pone.0143929.s013.docx]

**S2 Table. PCR primers used in this study.**

| **Primer** | **Syn.** | **Type** | **Dir.** | **Sequence** | **Source** |
| --- | --- | --- | --- | --- | --- |
| 518S |  | Both | F | TATGCTTGTCTCAAAGATTAA |  |
| S1893R | 18L | Both | R | CACCYACGGAAACCTTGTTACGACTT |  |
| SS398F | 18sai | Seq | F | CCTGAGAAACGGCTACCACATC | [1] |
| SS1054F | 760F | Seq | F | ATCAAGAACGAAAGT | [1] |
| SS1090R | 18sbi | Seq | R | GAGTCTCGTTCGTTATCGGA | [1] |
| SS1554R | 909R | Seq | R | GTCCTGTTCCATTATTCCAT | [2] |
|  |  |  |  |  |  |
| LCO1490 |  | Both | F | GGTCAACAAATCATAAAGATATTGG | [3] |
| HCO2198 |  | Both | R | TAAACTTCAGGGTGACCAAAAAATCA | [3] |
|  |  |  |  |  |  |
| LS58F | D1 | Both | F | GGGAGGAAAAGAAACTAAC | [4] |
| LS998R | D3 | Both | R | GCATAGTTCACCATCTTTC | [4] |
| NLF184 |  | Both | F | ACCCGCTGAAYTTAAGCATAT | [5] |
| LS1041R | D3aR | Both | R | TACGGACRTCCATCAGGGTTTCCCCTGACTTC | [6] |
| LS1F |  | Both | F | AGAGTTCAAGAGTACGTGAAACCG | * |
| D3L |  | Both | R | GCATAGTTCACCATCTTTCGGG | * |
| LS1R |  | Both | R | TTTCGGGTKTCWCAGGTTTAC | * |
|  |  |  |  |  |  |
| AK168F |  | PCR | F | CAGGTTTGGARAAYCACGAYTCYGG | [7] |
| AK939R |  | PCR | R | GCCNCCYTCRGCYTCRGTGTGYTC | [7] |
| AK183F |  | Both | F | GATTCTGGAGTCGGNATYTAYGCNCCYGAYGC | [7] |
| AK939R |  | Both | R | GCCNCCYTCRGCYTCRGTGTGYTC | [7] |
|  |  |  |  |  |  |
| CD338F |  | Both | F | ATGAARTAYGGYAATCGTGGHCAYAA | [8] |
| CD439F |  | Both | F | TTCAGTGTACARTTYCAYCCHGARCAYAC | [7] |
| CD688R |  | Both | R | TGTATACCTAGAGGATCDACRTTYTCCATRTTRCA | [7] |
| CD668R |  | Both | R | ACGACTTCATAYTCNACYTCYTTCCA | [7] |
| CD660F |  | Both | F | ATCATCGACAAGTCTCTBAARGGHTGGAARGA | [9] |
| CD667F |  | Both | R | GGATGGAAGGAAGTDGARTAYGARGT | [7] |
| CD851R |  | Both | R | GGATCGAAGCCATTHACATTYTCRTCHACCAT | [7] |
| CD828R |  | Both | R | GCCATTACYTCNCCNACACTYTTCAT | [7] |
| CD821F |  | PCR | F | AGCACGAAAATHGGNAGYTCNATGAARAG | [7] |
| CD1098R2 |  | Both | R | GCTATGTTGTTNGGNAGYTGDCCNCCCAT | [7] |
| CD806F |  | Both | F | GTNGTNAARATGCCNMGNTGGGA | [8] |
|  |  |  |  |  |  |
| wg550F |  | Both | F | ATGCGTCAGGARTGYAARTGYCAYGGYATGTC | [7] |
| wg578F |  | Both | F | TGCACNGTGAARACYTGCTGGATG | [10] |
| wgABR |  | Both | R | YTCGCAGCACCARTGGAA | [10] |
| wgABRZ |  | Both | R | CACTTNACYTCRCARCACCARTG | [7] |
|  |  |  |  |  |  |
|  |  |  |  |  |  |
| TP643F |  | Both | F | GACGATTGGAARTCNAARGARATG | [7] |
| TP675F |  | Both | F | GAGGACCAAGCNGAYACNGTDGGTTGTTG | [7] |
| TP932R |  | Both | R | GGWCCDGCATCDATDGCCCA | [7] |

**Primer**: official primer name. **Syn**.: synonym of primer name in literature. **Type**: whether primer was used in PCR, cycle-sequencing, or both**. Dir**.: whether primer is a forward primer (F) or a reverse primer (R). **Source**: original source of primer sequence as follows: * this study; numbers in brackets refer to the literature listed below.

1. Wray CG, Lee JJ, Desalle R (1993) Extraction and Enzymatic Characterization of Foraminiferal DNA. Micropaleontology 39: 69-73.

2. Maddison DR, Baker MD, Ober KA (1999) Phylogeny of carabid beetles as inferred from 18S ribosomal DNA (Coleoptera: Carabidae). Systematic Entomology 24: 103-138.

3. Hebert PDN, Cywinska A, Ball SL, DeWaard JR (2003) Biological identifications through DNA barcodes. Proceedings of the Royal Society of London Series B-Biological Sciences 270: 313-321.

4. Ober KA (2002) Phylogenetic relationships of the carabid subfamily Harpalinae (Coleoptera) based on molecular sequence data. Molecular Phylogenetics and Evolution 24: 228-248.

5. Van der Auwera G, Chapelle S, De Wachter R (1994) Structure of the large ribosomal subunit RNA of *Phytophthora megasperma*, and phylogeny of the oomycetes. FEBS Letters 338: 133-136.

6. Maddison DR (2008) Systematics of the North American beetle subgenus *Pseudoperyphus* (Coleoptera: Carabidae: *Bembidion*) based upon morphological, chromosomal, and molecular data. Annals of Carnegie Museum 77: 147-193.

7. Wild AL, Maddison DR (2008) Evaluating nuclear protein-coding genes for phylogenetic utility in beetles. Molecular Phylogenetics and Evolution 48: 877-891.

8. Moulton JK, Wiegmann BM (2004) Evolution and phylogenetic utility of CAD (rudimentary) among Mesozoic-aged Eremoneuran Diptera (Insecta). Molecular Phylogenetics and Evolution 31: 363-378.

9. Maddison DR (2012) Phylogeny of *Bembidion* and related ground beetles (Coleoptera: Carabidae: Trechinae: Bembidiini: Bembidiina). Molecular Phylogenetics and Evolution 63: 533-576.

10. Ward PS, Downie DA (2005) The ant subfamily Pseudomyrmecinae (Hymenoptera: Formicidae): phylogeny and evolution of big-eyed arboreal ants. Systematic Entomology 30: 310-335.
