# Supplementary material for: Successful Recovery of Nuclear Protein-Coding Genes from Small Insects in Museums Using Illumina Sequencing
Source: PLoS One. 2015 Dec 30;10(12):e0143929. doi: 10.1371/journal.pone.0143929 (PMC4696846; doi:10.1371/journal.pone.0143929)
Supplement: S3 Table — (DOCX) [file pone.0143929.s014.docx]

**S3 Table. Comparison of Sanger sequenced gene fragments and corresponding Illumina sequenced region for museum specimens.**

| **Taxon** | **Sample** |  | **28S f1** | | |  | **28S f2** | | |  | ***wg*** | | |  | **COI** | | |
| --- | --- | --- | --- | --- | --- | --- | --- | --- | --- | --- | --- | --- | --- | --- | --- | --- | --- |
|  |  |  | **P≠I** | **P**⊃**I** | **P**⊂**I** |  | **P≠I** | **P**⊃**I** | **P**⊂**I** |  | **P≠I** | **P**⊃**I** | **P**⊂**I** |  | **P≠I** | **P**⊃**I** | **P**⊂**I** |
| *Lionepha chintimini* | 4002 |  | 0 | 0 | 0 |  | - | - | - |  | - | - | - |  | - | - | - |
| *Bembidion lachnophoroides* | 3022 |  | 0 | 0 | 0 |  | - | - | - |  | - | - | - |  | - | - | - |
| *Bembidion lapponicum* | 3974 |  | 0 | 0 | 0 |  | - | - | - |  | - | - | - |  | - | - | - |
| *Bembidion* "Arica" | 3242 |  | 0 | 0 | 0 |  | 0 | 0 | 0 |  | **1** | 0 | 0 |  | 0 | **11** | 0 |
| *Bembidion* "Desert Spotted" | 3978 |  | 0 | 0 | 1 |  | 0 | 0 | 0 |  | - | - | - |  | - | - | - |
| *Bembidion musae* | 3239 |  | **1** | **2** | 0 |  | - | - | - |  | **3** | 0 | **2** |  | - | - | - |
| *Bembidion* "Inuvik" | 3984 |  | 0 | 0 | 0 |  | 0 | 0 | 0 |  | 0 | **3** | 0 |  | 0 | **1** | 0 |

**P≠I**: Number of unambiguous base conflicts between PCR-based (**P)** and Illumina-based (**I**) assembly consensus sequences. **P**⊃**I**: Number of base conflicts due to an ambiguity in the PCR fragment at a position that is unambiguous in the Illumina sequence such that the PCR based fragment is a superset of the Illumina fragment (e.g. an ambiguity “R” was called in the PCR fragment due to a double peak in the chromatograms, but the Illumina fragment has a G at that position). **P**⊂**I**: Number of ambiguous base conflicts due to an ambiguity in the Illumina sequence at a position that is unambiguous in the PCR fragment such that the base in the Illumina fragment is a superset of the PCR fragment. "-" indicates a gene fragment for which PCR amplification was unsuccessful. All ambiguities in the Illumina sequence were due to our using a consensus sequence of the reference-based and *de novo* assemblies to compare to the PCR sequence. Because there were occasionally conflicts between the reference-based and *de novo* assemblies, ambiguities are present in the Illumina consensus sequence.
