# Supplementary material for: Successful Recovery of Nuclear Protein-Coding Genes from Small Insects in Museums Using Illumina Sequencing
Source: PLoS One. 2015 Dec 30;10(12):e0143929. doi: 10.1371/journal.pone.0143929 (PMC4696846; doi:10.1371/journal.pone.0143929)
Supplement: S4 Table — (DOCX) [file pone.0143929.s015.docx]

**S4 Table: Number of candidate contigs for each gene in the *de novo* assemblies, and the number chosen for subsequent analyses.**

|  |  | **18S** | | **28S** | | **COI** | | **ArgK** | | **CAD** | | **Topo** | | ***wg*** | |
| --- | --- | --- | --- | --- | --- | --- | --- | --- | --- | --- | --- | --- | --- | --- | --- |
| **Taxa** | **Sample** | **?** | **√** | **?** | **√** | **?** | **√** | **?** | **√** | **?** | **√** | **?** | **√** | **?** | **√** |
|  |  |  |  |  |  |  |  |  |  |  |  |  |  |  |  |
| Lagriinae n. gen. | KK0290 | 1 | 1 | 1 | 1 | 1 | 1 | 2 | 2^3^ | 2 | 2^3^ | - | - | x | x |
| *Bembidion subfusum* | 3977 | 3 | 1^1^ | 2 | 1^1^ | 1 | 1 | x | x | x | x | 1 | 1 | x | x |
| *Bembidion* sp. nr. *transversale* | 3021 | 1 | 1 | 1 | 1 | 8 | 1^1^ | 1 | 1 | 1 | 1 | 2 | 2^3^ | x | x |
| *Lionepha chintimini* | 4002 | 2 | 1^1^ | 1 | 1 | 1 | 1 | 2 | 0^2^ | 1 | 1 | 2 | 2^3^ | 1 | 1 |
| *Bembidion lachnophoroides* | 3022 | 20 | 1^1^ | 7 | 1^1^ | 5 | 1^1^ | 1 | 1 | 1 | 1 | 3 | 3^3^ | 1 | 1 |
| *Bembidarenas* | 3983 | 2 | 1^1^ | 1 | 1 | 1 | 1 | 1 | 1 | x | x | x | x | 1 | 1 |
| *Bembidion orion* | 2831 | 1 | 1 | 1 | 1 | 5 | 1^1^ | 2 | 1^1^ | 2 | 2^3^ | 1 | 1 | 2 | 2^3^ |
| *Bembidion* "Inuvik" | 3285 | 1 | 1 | 1 | 1 | 1 | 1 | x | x | x | x | x | x | x | x |
| *Bembidion lapponicum* | 3974 | 7 | 1^1^ | 6 | 1^1^ | 1 | 1 | x | x | 1 | 1 | 2 | 2^3^ | 1 | 1 |
| *Bembidion* "Arica" | 3242 | 11 | 1^1^ | 6 | 1^1^ | 2 | 1^1^ | 1 | 1 | 3 | 2^3^ | 3 | 2^3^ | 1 | 1 |
| *Bembidion* cf. "Desert Spotted" | 3978 | 2 | 2^3^ | 1 | 1 | 1 | 1 | x | x | 1 | 1 | x | x | x | x |
| *Bembidion musae* | 3239 | 4 | 1^1^ | 3 | 1^1^ | 2 | 1^1^ | x | x | 2 | 2^3^ | x | x | x | x |
| *Bembidion* "Inuvik" | 3984 | 3 | 1^1^ | 1 | 1 | x | x | 2 | 2^3^ | x | x | 2 | 2^3^ | 1 | 1 |
| *Bembidion orion* | 3079 | 1 | 1 | 2 | 1^1^ | 2 | 1^1^ | 2 | 2^3^ | 1 | 1 | 1 | 1 | 1 | 1 |
| *Bembidion* sp. nr. *transversale* | 3205 | 9 | 1^1^ | 6 | 1^1^ | 4 | 1^1^ | 1 | 1 | 1 | 1 | 1 | 1 | 1 | 1 |

**?**: the number of contigs that are candidate orthologs, having passed the first phase of our selection process (that is, after removal of sequences that BLAST to non-insects, or that overlap the analyzed region by less than 30 bases). **√**: the number of contigs that passed the second phase of our selection process (removal of fragments showing stop codons, and shorter fragments), and thus that were chosen to form the *de novo* sequence. An “**x**” indicates that no sequences were returned in BLAST searches of the *de novo* assembly, and “**-**” indicates gene was not sought for that sample.

^1^ Multiple contigs matched the query but only one of the hits spanned the entire query region.

^2^ Multiple contigs matched the query but our selection process failed to choose one.

^3^ Multiple contigs matched the query, all of which passed our selection process; contigs merged.
