# Supplementary material for: Successful Recovery of Nuclear Protein-Coding Genes from Small Insects in Museums Using Illumina Sequencing
Source: PLoS One. 2015 Dec 30;10(12):e0143929. doi: 10.1371/journal.pone.0143929 (PMC4696846; doi:10.1371/journal.pone.0143929)
Supplement: S5 Table — (DOCX) [file pone.0143929.s016.docx]

**S5 Table. Preservation and collection data for Tenebrionidae sampled for PCR and Sanger sequencing.**

| **Taxa** | **Sample** | **Repository** | **Preservation** | **Locality and collection date** |
| --- | --- | --- | --- | --- |
| *Cardiothorax* sp. | KK0316 | KK | >90% EtOH | Australia: Queensland: Bulburin Nat. Pk. Rainforest. 14.i.2008 |
| *Zeadelium gratiosum* | KK0228 | KK | >90% EtOH | New Zealand: Tasman: Abel Tasman N.P. nr Canaan Downs campg. 9.iv.2010 |
| *Adelonia* sp. | KK0099 | KK | >90% EtOH | Peru: Rio Tambopata Explorer’s Inn, Rio Tower. 6-13.xii.2003 |
| *Rhypasma* sp. | KK0312 | KK | >90% EtOH | USA: Texas: Cameron Co.: Resaca de la Palma SP. 15.xi.2013 |
| *Chaerodes trachyscelides* | KK0163 | KK | >90% EtOH | New Zealand: Auckland: Waimauku, Kerr Taylor Res. 27.xi.2010 |
| *Cossyphus* sp. | KK0100 | KK | >90% EtOH | South Africa: Zwa-Zulu Natal: Mkuze. 12.i.2004 |
| *Anaedus brunneus* | KK0128 | KK | >90% EtOH | USA: Maryland: Anne Arundel Co.: Patuxent Research Refuge, North Tract. 6.xiii.2010 |
| *Anaedus leleupi* | KK0314 | CAS | >90% EtOH | South Africa: Mpumalanga: Nelspruit Nature Reserve. 14.xii.2003 |
| *Goniadera* sp. | KK0342 | KK | >90% EtOH | Panama |
| *Paratenetus punctatus* | KK0075 | KK | >90% EtOH | USA: Kansas: Jefferson Co.: Perry Lake trail. |
| *Phymatestes* sp. | KK0046 | BYU | >90% EtOH | Bolivia: Santa Cruz: Conception. 12.i.2004 |
| *Prateus fuscus* | KK0295 | SEMC | dry | USA: Kansas: Chase Co.: Chase Co. State Fishing Lake. 8.x.2011 |
| *Pseudolyprops* sp. | KK0245 | KK | dry | Australia: Queensland: Cairns, Copperload Dam Rd, UV. 9.i.2008 |
| *Laena dilutela* | KK0340 | SMNS | >90% EtOH | Kirghizia: S Jengi-Jol. |
| *Laena dilutela* | KK0341 | SMNS | >90% EtOH | Kirghizia: S Jengi-Jol. |
| *Laena franzi* | KK0313 | SMNS | dry | Nepal: Mustang: Purano Marpha. 22-25.iv.1980 |
| *Mimolaena clarissae* | KK0324 | CAS | dry | Madagascar: Toliara: Foret de Mahavelo, Isantoria River. 31.i.2002 |
| *Lagria* sp. | KK0057 | KK | >90% EtOH | Madagascar: Toliara: nr Ft. Dauphin, Andohahela National Park, Parcel I. 23-26.xii.2008 |
| *Phobelius* sp. | KK0288 | USNM | dry | Bolivia: El Beni: Beni Stn., Palm Camp NE San Borja. 25-30.vii.1988 |
| *Statira pluripunctatus* | KK0055 | KK | >90% EtOH | USA: Arizona: Santa Cruz Co.: Atascosa Highlands, Walker Canyon. 9.vii.2009 |
| *Antennoluprops bremeri* | KK0321 | CAS | dry | Madagascar: Province d’Antananarivo: 3 km NE Andranomay. 2-13.xii.200 |
| *Coxelinus* sp. | KK0315 | KK | >90% EtOH | Madagascar: Vatovavy-Fitovinany: Roanomafana N. P., Centre ValBio Station. 10-14.xi.2008 |
| *Enicmosoma decorsei* | KK0235 | KK | >90% EtOH | Madagascar: Anosy: Berenty Reserve. 15-18.xi.2008 |
| *Lorelus* sp. | KK0168 | KK | >90% EtOH | New Zealand: Auckland: Waimauku, Kerr Taylor Res. 27.xi.2010 |
| *Luprops* sp. | KK0082 | BYU | >90% EtOH | India: Kerala: Thiruvananthapuram Pon Mundi. 23.v.2003 |
| *Chaetyllus* n. sp. 5 | KK0276 | SEMC | dry | Venezuela: Lara: Sanare, 6.4km SE. 17.v.1998 |
| Lagriinae n. gen. 2 | KK0148 | KK | >90% EtOH | Ecuador: Napo: Papallacta. 27.x.2010 |
| *Clamoris americana* | KK0065 | KK | >90% EtOH | USA: California: El Dorado Co.: El Dorado NF, 3 mi. S on Silverfork Rd. 9.vi.2008 |
| *Eupsophulus castaneus* | KK0018 | KK | >90% EtOH | USA: Arizona: Pinal Co.: Peppersauce Canyon. |
| *Peschalius dentiger* | KK0016 | KK | >90% EtOH | USA: Arizona: Pima Co.: Tucson. 12.ii.2009 |
| *Strongylium atrum* | KK0039 | KK | >90% EtOH | USA: Arizona: Santa Cruz Co.: Little Outfit Ranch. 1.viii.2008 |
| *Tenebrio molitor* | KK0266 | KK | >90% EtOH | Lab colony |
| *Metallonotus* sp. | KK0224 | KK | >90% EtOH | Zambia: Copperbelt: Kumasamba Lodge, ~10km SE Kitwe. 31.x.2010 |
| *Odontopezus* sp. | KK0042 | BYU | >90% EtOH | Ghana: Western Reg.: Ankasa Resource Res., Nkwanta Camp. 7-8.vi.2005 |

**Preservation**: How the specimen was preserved at the time of DNA extraction. **Repository**: Institution where the specimen is deposited. For explanations of codens, refer to text. Collectors include Rolf Aalbu, Robert Anderson, Seth Bybee, Steve Cameron, Taro Eldredge, Terry Erwin, Zach Falin, Martin Fikacek, Brian Fisher, Peter Grimbacher, Charles Griswold, Rich Leschen, Nathan Lord, Wayne Maddison, Kelly Miller, Geoff Monteith, Wendy Moore, Heath Ogden, Wolfgang Schawaller, Warren Steiner, Gavin Svenson, and Kipling Will.
