# Supplementary material for: Successful Recovery of Nuclear Protein-Coding Genes from Small Insects in Museums Using Illumina Sequencing
Source: PLoS One. 2015 Dec 30;10(12):e0143929. doi: 10.1371/journal.pone.0143929 (PMC4696846; doi:10.1371/journal.pone.0143929)
Supplement: S6 Table — (DOCX) [file pone.0143929.s017.docx]

**S6 Table. Thermocycler profiles used in PCR amplification of focal genes.**

| **Name** | **Annealing temp (ºC)** | **Extension time (sec.)** | **Cycles** |
| --- | --- | --- | --- |
|  |  |  |  |
| ST-50-C | 50 | 150 | 38 |
| ST-52-B | 52 | 75 | 38 |
| ST-57-C | 57 | 120 | 38 |
| ST-54-C | 54 | 75 | 38 |
| ST-54-D | 54 | 90 | 38 |
| ST-55-B | 55 | 90 | 38 |
| TD-60-O | 60 | 210 | 9 |
|  | 55 | 210 | 30 |
| TD-60-M | 60 | 90 | 9 |
|  | 55 | 90 | 30 |

**Name**: refers to those used in the PCR amplification protocols described in the S1 Supplemental Methods. All cycle profiles begin with a 2-minute denaturation at 94˚ C and end with a 5-minute extension at 72˚ C. TD-60-O and TD-60-M are touch-down protocols with nine cycles at 60˚ C followed by 30 cycles at 55˚ C.
