# Supplementary material for: Successful Recovery of Nuclear Protein-Coding Genes from Small Insects in Museums Using Illumina Sequencing
Source: PLoS One. 2015 Dec 30;10(12):e0143929. doi: 10.1371/journal.pone.0143929 (PMC4696846; doi:10.1371/journal.pone.0143929)
Supplement: S7 Table — (DOCX) [file pone.0143929.s018.docx]

**S7 Table. Proportion of recovered bases from 67-gene set: *de novo* assemblies.**

| **Gene region** | **Length** | **Lag** | **subf** | **snt1** | **Lchi** | **lach** | **Bdrs** | **ori1** | **inu1** | **lapp** | **aric** | **dspt** | **mus** | **inu2** | **ori2** | **snt2** | ***Average*** |
| --- | --- | --- | --- | --- | --- | --- | --- | --- | --- | --- | --- | --- | --- | --- | --- | --- | --- |
| 8018fin1_2 | **338** | 0.00 | 0.00 | 0.00 | 0.00 | 0.00 | 0.00 | 0.71 | 0.00 | 0.00 | 0.71 | 0.00 | 0.00 | 0.00 | 0.71 | 0.71 | *0.19* |
| 3044fin1_2 | **542** | 0.00 | 0.00 | 0.00 | 0.48 | 0.26 | 0.00 | 0.00 | 0.00 | 0.49 | 0.73 | 0.00 | 0.73 | 0.49 | 0.49 | 0.94 | *0.31* |
| 8028fin1_2 | **578** | 0.00 | 0.00 | 0.00 | 0.52 | 0.52 | 0.00 | 0.00 | 0.00 | 0.00 | 0.42 | 0.00 | 0.00 | 0.52 | 0.94 | 0.52 | *0.23* |
| 197fin1_2 | **428** | 0.50 | 0.00 | 0.00 | 0.00 | 1.00 | 0.00 | 1.00 | 0.00 | 0.00 | 0.54 | 0.00 | 0.00 | 0.50 | 1.00 | 1.00 | *0.37* |
| 36fin1_2 | **545** | 0.36 | 0.00 | 0.00 | 0.23 | 0.59 | 0.00 | 0.59 | 0.00 | 0.00 | 0.59 | 0.00 | 0.59 | 0.36 | 0.36 | 0.36 | *0.27* |
| aspec2_6 | **476** | 0.00 | 0.00 | 0.30 | 0.95 | 0.00 | 0.00 | 0.49 | 0.00 | 0.00 | 0.92 | 0.00 | 0.00 | 0.88 | 1.00 | 1.00 | *0.37* |
| 63fin2_3 | **977** | 0.32 | 0.00 | 0.00 | 0.00 | 0.52 | 0.00 | 0.52 | 0.00 | 0.00 | 0.25 | 0.00 | 0.00 | 0.61 | 0.52 | 0.52 | *0.22* |
| acc2_4 | **539** | 0.00 | 0.00 | 0.00 | 0.00 | 0.64 | 0.00 | 0.00 | 0.00 | 0.21 | 0.56 | 0.39 | 0.00 | 1.00 | 1.00 | 0.68 | *0.30* |
| 25fin2_4 | **488** | 0.00 | 0.00 | 0.32 | 0.69 | 0.57 | 0.00 | 0.00 | 0.00 | 0.00 | 0.00 | 0.00 | 0.69 | 0.31 | 0.31 | 0.00 | *0.19* |
| 270fin2_3 | **494** | 0.00 | 0.00 | 0.30 | 0.00 | 0.22 | 0.00 | 1.00 | 0.00 | 0.00 | 0.00 | 0.00 | 0.00 | 0.28 | 0.94 | 1.00 | *0.25* |
| 42fin1_2 | **494** | 0.00 | 0.00 | 0.00 | 0.68 | 0.44 | 0.00 | 0.62 | 0.00 | 0.10 | 0.62 | 0.23 | 0.13 | 0.53 | 0.73 | 0.73 | *0.32* |
| 44fin2_3 | **899** | 0.63 | 0.00 | 0.00 | 0.35 | 0.69 | 0.00 | 0.00 | 0.00 | 0.68 | 0.41 | 0.00 | 0.00 | 0.00 | 0.00 | 0.00 | *0.18* |
| 3202fin1_3 | **807** | 0.50 | 0.00 | 0.22 | 0.00 | 0.40 | 0.00 | 0.43 | 0.00 | 0.25 | 0.48 | 0.00 | 0.00 | 0.90 | 0.71 | 0.48 | *0.29* |
| 8029fin6_7 | **659** | 0.00 | 0.00 | 0.00 | 0.45 | 0.32 | 0.00 | 0.99 | 0.00 | 0.00 | 0.67 | 0.00 | 0.00 | 0.00 | 1.00 | 0.56 | *0.27* |
| 3006fin1_2 | **512** | 0.00 | 0.00 | 0.00 | 0.53 | 0.00 | 0.00 | 0.53 | 0.00 | 0.00 | 0.54 | 0.00 | 0.00 | 0.53 | 0.53 | 0.53 | *0.21* |
| 96fin1_3 | **644** | 0.00 | 0.00 | 0.38 | 0.00 | 0.60 | 0.00 | 0.55 | 0.00 | 0.00 | 0.87 | 0.00 | 0.42 | 0.55 | 0.55 | 0.55 | *0.30* |
| 262fin1_2 | **561** | - | 0.00 | 0.00 | 0.00 | 0.21 | 0.00 | 0.71 | 0.00 | 0.00 | 0.53 | 0.21 | 0.28 | 0.27 | 0.28 | 0.72 | *0.23* |
| 3007fin1_2 | **1010** | 0.93 | 0.00 | 0.43 | 0.23 | 0.56 | 0.00 | 0.48 | 0.00 | 0.00 | 0.00 | 0.00 | 0.33 | 0.57 | 0.57 | 0.57 | *0.31* |
| 3152fin1_2 | **797** | 0.00 | 0.00 | 0.00 | 0.79 | 0.57 | 0.00 | 0.40 | 0.00 | 0.00 | 0.79 | 0.00 | 0.28 | 0.79 | 0.79 | 0.79 | *0.35* |
| 8070fin1_3 | **476** | 0.00 | 0.00 | 0.00 | 0.86 | 0.83 | 0.00 | 1.00 | 0.00 | 0.00 | 0.74 | 0.00 | 0.00 | 0.23 | 0.96 | 1.00 | *0.37* |
| 3012fin1_2 | **623** | 0.58 | 0.00 | 0.48 | 0.29 | 0.84 | 0.00 | 0.45 | 0.00 | 0.00 | 0.50 | 0.00 | 0.00 | 0.57 | 1.00 | 1.00 | *0.38* |
| 40fin2_3 | **566** | 0.26 | 0.00 | 0.00 | 0.32 | 1.00 | 0.00 | 0.88 | 0.00 | 0.33 | 1.00 | 0.00 | 0.00 | 0.93 | 1.00 | 0.00 | *0.38* |
| 3136fin1_2 | **579** | 0.00 | 0.00 | 0.00 | 0.58 | 0.27 | 0.00 | 0.00 | 0.00 | 0.35 | 0.69 | 0.17 | 0.39 | 0.00 | 0.00 | 0.00 | *0.16* |
| 113fin1_2 | **374** | 0.61 | 0.00 | 0.29 | 0.29 | 0.68 | 0.00 | 0.71 | 0.00 | 0.00 | 0.49 | 0.00 | 0.31 | 0.36 | 0.98 | 0.31 | *0.34* |
| 58fin7_9 | **2306** | 0.76 | 0.00 | 0.00 | 0.53 | 0.73 | 0.00 | 0.75 | 0.00 | 0.00 | 0.89 | 0.00 | 0.36 | 0.53 | 0.53 | 0.53 | *0.37* |
| 3064fin6_7 | **632** | 0.36 | 0.00 | 0.00 | 0.28 | 0.69 | 0.00 | 0.59 | 0.00 | 0.00 | 0.53 | 0.25 | 0.29 | 0.47 | 1.00 | 0.75 | *0.35* |
| 3196fin5_6 | **701** | 0.00 | 0.00 | 0.00 | 0.82 | 0.60 | 0.00 | 0.47 | 0.00 | 0.19 | 0.17 | 0.00 | 0.14 | 0.70 | 0.96 | 0.70 | *0.32* |
| 69fin2_3 | **581** | 0.00 | 0.00 | 0.46 | 0.46 | 0.49 | 0.00 | 0.47 | 0.00 | 0.00 | 0.23 | 0.28 | 0.69 | 0.28 | 0.88 | 0.81 | *0.34* |
| 58fin3_6 | **1178** | 0.40 | 0.00 | 0.22 | 0.56 | 0.38 | 0.00 | 0.77 | 0.00 | 0.30 | 0.93 | 0.00 | 0.27 | 0.90 | 0.59 | 0.59 | *0.39* |
| 62fin2_3 | **459** | 0.00 | 0.00 | 0.25 | 0.23 | 0.53 | 0.00 | 0.79 | 0.00 | 0.00 | 0.90 | 0.00 | 0.21 | 0.91 | 0.00 | 0.64 | *0.30* |
| 8053fin2_3 | **668** | 0.00 | 0.00 | 0.13 | 0.44 | 0.67 | 0.00 | 0.67 | 0.00 | 0.00 | 0.43 | 0.23 | 0.29 | 0.43 | 0.67 | 1.00 | *0.33* |
| 247fin1_2 | **413** | 0.00 | 0.00 | 0.00 | 0.34 | 1.00 | 0.00 | 0.67 | 0.00 | 0.33 | 0.36 | 0.00 | 0.36 | 0.68 | 0.68 | 1.00 | *0.36* |
| 3114fin1_2 | **365** | 0.00 | 0.00 | 0.32 | 0.46 | 0.47 | 0.00 | 0.86 | 0.00 | 0.00 | 0.00 | 0.47 | 0.60 | 0.47 | 0.86 | 0.47 | *0.33* |
| 265fin2_3 | **782** | 0.00 | 0.00 | 0.49 | 0.00 | 1.00 | 0.00 | 1.00 | 0.00 | 0.00 | 0.93 | 0.00 | 0.00 | 0.00 | 1.00 | 1.00 | *0.36* |
| 3121fin1_2 | **1322** | 0.48 | 0.00 | 0.18 | 0.75 | 0.53 | 0.00 | 0.56 | 0.00 | 0.49 | 0.77 | 0.00 | 0.00 | 0.00 | 0.82 | 0.71 | *0.35* |
| 192fin1_2 | **362** | 0.00 | 0.00 | 0.00 | 0.73 | 0.83 | 0.00 | 0.96 | 0.00 | 0.00 | 1.00 | 0.00 | 0.00 | 0.54 | 0.96 | 0.43 | *0.36* |
| 3094fin2_3 | **377** | 0.00 | 0.00 | 0.00 | 0.00 | 1.00 | 0.00 | 0.49 | 0.00 | 0.57 | 1.00 | 0.00 | 0.00 | 0.87 | 1.00 | 1.00 | *0.40* |
| 8091fin1_2 | **263** | 0.00 | 0.00 | 0.00 | 0.44 | 0.00 | 0.00 | 0.81 | 0.00 | 0.00 | 0.40 | 0.00 | 0.68 | 0.95 | 0.86 | 0.87 | *0.33* |
| 166fin2_3 | **968** | 0.00 | 0.00 | 0.57 | 0.38 | 0.32 | 0.00 | 0.26 | 0.00 | 0.00 | 0.64 | 0.00 | 0.00 | 0.65 | 0.92 | 1.00 | *0.32* |
| 3017fin1_2 | **803** | 0.00 | 0.00 | 0.00 | 0.40 | 0.67 | 0.00 | 0.47 | 0.00 | 0.00 | 0.82 | 0.44 | 0.19 | 0.47 | 0.47 | 0.47 | *0.29* |
| 149fin2_3 | **887** | 0.64 | 0.00 | 0.00 | 0.00 | 0.62 | 0.00 | 0.93 | 0.00 | 0.27 | 0.94 | 0.00 | 0.25 | 0.78 | 1.00 | 1.00 | *0.43* |
| aspec11_12 | **704** | 0.00 | 0.00 | 0.35 | 0.94 | 0.54 | 0.00 | 0.97 | 0.00 | 0.49 | 0.97 | 0.00 | 0.00 | 0.97 | 0.97 | 0.97 | *0.48* |
| 220fin1_2 | **407** | 0.00 | 0.00 | 0.00 | 0.91 | 0.77 | 0.00 | 0.84 | 0.00 | 0.61 | 0.82 | 0.00 | 0.31 | 0.00 | 0.69 | 0.69 | *0.38* |
| 3031fin1_3 | **890** | 0.00 | 0.00 | 0.62 | 0.72 | 0.00 | 0.00 | 1.00 | 0.00 | 0.00 | 0.00 | 0.00 | 0.83 | 0.69 | 0.71 | 0.00 | *0.30* |
| 3066fin1_3 | **348** | 0.00 | 0.00 | 0.34 | 0.49 | 0.95 | 0.00 | 0.84 | 0.00 | 0.00 | 0.71 | 0.00 | 0.00 | 0.49 | 0.49 | 1.00 | *0.36* |
| EF-1α | **572** | 0.54 | 0.00 | 0.18 | 0.45 | 0.46 | 0.25 | 0.00 | 0.00 | 0.61 | 0.00 | 0.00 | 0.29 | 0.39 | 0.00 | 0.00 | *0.21* |
| 2F3_4 | **683** | 0.00 | 0.00 | 0.78 | 0.37 | 0.84 | 0.00 | 0.85 | 0.00 | 0.64 | 0.37 | 0.00 | 0.58 | 0.96 | 0.37 | 0.96 | *0.45* |
| 268fin1_2 | **656** | 0.28 | 0.00 | 0.19 | 0.57 | 0.85 | 0.00 | 0.65 | 0.00 | 0.00 | 0.91 | 0.13 | 0.57 | 0.79 | 0.57 | 0.57 | *0.41* |
| aspec19_21 | **440** | 0.00 | 0.00 | 0.31 | 0.99 | 1.00 | 0.00 | 0.79 | 0.00 | 0.35 | 0.47 | 0.00 | 0.00 | 0.80 | 0.89 | 0.54 | *0.41* |
| 3153fin1_2 | **554** | 0.00 | 0.00 | 0.00 | 0.66 | 0.77 | 0.00 | 0.95 | 0.00 | 0.86 | 0.74 | 0.00 | 0.24 | 0.81 | 1.00 | 0.88 | *0.46* |
| 274fin1_2 | **581** | 0.73 | 0.00 | 0.00 | 0.71 | 0.95 | 0.00 | 0.83 | 0.00 | 0.35 | 1.00 | 0.00 | 0.70 | 0.65 | 0.82 | 0.53 | *0.48* |
| 109fin1_2 | **506** | 0.00 | 0.00 | 0.27 | 0.34 | 0.49 | 0.00 | 0.52 | 0.00 | 0.47 | 0.70 | 0.50 | 0.90 | 0.51 | 1.00 | 0.54 | *0.42* |
| 3196fin1_3 | **317** | 0.50 | 0.00 | 0.00 | 0.76 | 0.42 | 0.00 | 1.00 | 0.00 | 0.00 | 0.92 | 0.00 | 0.85 | 1.00 | 1.00 | 0.41 | *0.46* |
| 3070fin4_5 | **281** | 0.75 | 0.00 | 0.27 | 0.98 | 0.99 | 0.00 | 0.89 | 0.00 | 0.41 | 0.81 | 0.35 | 0.49 | 1.00 | 1.00 | 1.00 | *0.60* |
| 3031fin4_5 | **749** | 0.00 | 0.00 | 0.78 | 1.00 | 1.00 | 0.00 | 0.00 | 0.44 | 0.41 | 0.00 | 0.00 | 0.58 | 0.00 | 0.00 | 0.00 | *0.28* |
| 73fin2_3 | **572** | 0.00 | 0.00 | 0.00 | 0.18 | 0.79 | 0.00 | 1.00 | 0.00 | 0.61 | 0.93 | 0.00 | 0.29 | 1.00 | 1.00 | 1.00 | *0.45* |
| EF-2 | **626** | 0.35 | 0.00 | 0.66 | 0.51 | 0.78 | 0.00 | 0.57 | 0.00 | 0.49 | 0.72 | 0.16 | 0.29 | 0.20 | 0.20 | 0.52 | *0.36* |
| 127fin1_2 | **590** | 0.50 | 0.00 | 0.00 | 0.79 | 0.91 | 0.00 | 1.00 | 0.00 | 0.00 | 0.61 | 0.00 | 0.00 | 0.99 | 1.00 | 1.00 | *0.45* |
| 6fin2_3 | **779** | 0.96 | 0.00 | 0.00 | 1.00 | 0.89 | 0.00 | 0.00 | 0.00 | 0.77 | 1.00 | 0.00 | 0.00 | 0.00 | 0.87 | 1.00 | *0.43* |
| 2F7_8 | **632** | 0.00 | 0.00 | 0.00 | 0.73 | 0.75 | 0.00 | 1.00 | 0.00 | 0.47 | 0.98 | 0.00 | 0.25 | 1.00 | 1.00 | 1.00 | *0.48* |
| 26fin3_4 | **333** | 0.00 | 0.00 | 0.40 | 1.00 | 0.70 | 0.00 | 0.94 | 0.00 | 0.00 | 0.68 | 0.00 | 0.87 | 1.00 | 0.51 | 0.51 | *0.44* |
| 3009fin2_3 | **752** | 0.00 | 0.00 | 0.40 | 0.67 | 0.98 | 0.46 | 0.99 | 0.00 | 0.67 | 0.79 | 0.31 | 0.58 | 1.00 | 0.67 | 0.67 | *0.55* |
| 226fin1_2 | **500** | 0.00 | 0.00 | 0.58 | 0.89 | 1.00 | 0.00 | 0.87 | 0.00 | 0.00 | 1.00 | 0.00 | 0.00 | 1.00 | 1.00 | 1.00 | *0.49* |
| PolII | **381** | 0.24 | 0.00 | 0.19 | 0.39 | 0.87 | 0.00 | 0.97 | 0.00 | 0.65 | 1.00 | 0.12 | 0.27 | 0.97 | 0.81 | 1.00 | *0.50* |
| 3055fin2_3 | **771** | 0.74 | 0.00 | 0.00 | 0.81 | 0.87 | 0.00 | 0.94 | 0.00 | 0.75 | 0.86 | 0.00 | 0.00 | 1.00 | 1.00 | 1.00 | *0.53* |
| 3059fin1_3 | **347** | 0.33 | 0.00 | 0.14 | 0.92 | 0.82 | 0.00 | 1.00 | 0.00 | 0.77 | 1.00 | 0.00 | 1.00 | 1.00 | 1.00 | 1.00 | *0.60* |
| 3089fin1_3 | **461** | 0.00 | 0.00 | 0.48 | 1.00 | 1.00 | 0.00 | 1.00 | 0.00 | 1.00 | 0.98 | 0.97 | 0.00 | 1.00 | 1.00 | 1.00 | *0.63* |
|  |  |  |  |  |  |  |  |  |  |  |  |  |  |  |  |  |  |
| ***Avg.*** |  | *0.20* | *0.00* | *0.18* | *0.51* | *0.64* | *0.01* | *0.66* | *0.01* | *0.24* | *0.64* | *0.08* | *0.27* | *0.60* | *0.73* | *0.68* | *0.36* |
| ***Avg. RS*** |  |  |  |  |  |  |  |  |  |  |  |  |  |  | *0.73* | *0.68* | *0.71* |
| ***Avg. MS*** |  | *0.20* | *0.00* | *0.18* | *0.51* | *0.64* | *0.01* | *0.66* | *0.01* | *0.24* | *0.64* | *0.08* | *0.27* | *0.60* |  |  | *0.31* |
| ***Avg. MS - LR*** |  | *0.20* |  | *0.18* | *0.51* | *0.64* |  | *0.66* |  | *0.24* | *0.64* |  | *0.27* | *0.60* |  |  | *0.44* |

**Gene region**: abbreviation used in [1], for 67 target genes. **Length**: length of fragment in query sequences from *Bembidion* sp. nr. *transversale* 3205. **Lag**: Lagriinae n. gen. KK0290. **subf**: *Bembidion subfusum* 3977. **snt1**: *Bembidion* sp. nr. *transversale* 3021. **Lchi**: *Lionepha chintimini* 4002. **lach**: *Bembidion lachnophoroides* 3022. **Bdrs**: *Bembidarenas* 3983. **ori1**: *Bembidion orion* 2831. **inu1**: *Bembidion* "Inuvik"3285. **lapp**: *Bembidion lapponicum* 3974. **aric**: *Bembidion* "Arica" 3242. **dspt**: *Bembidion* *cf*. "Desert Spotted" 3978. **mus**: *Bembidion musae* 3239. **inu2**: Bembidion "Inuvik" 3984. **ori2**: *Bembidion orion* 3079. **snt2**: *Bembidion* sp. nr. *transversale* 3205. **Avg.**: average proportion of bases recovered for all museum. specimens and both reference specimens. **Avg. RS**: average proportion of bases recovered for the two reference specimens. **Avg. MS**: average proportion of bases recovered for all museum specimens. **Avg. MS - LR**: average proportion of bases recovered for museum specimens, excluding the 4 museum specimens with less than 34 million reads.

1. Regier JC, Shultz JW, Ganley ARD, Hussey A, Shi D, et al. (2008) Resolving Arthropod Phylogeny: Exploring Phylogenetic Signal within 41 kb of Protein-Coding Nuclear Gene Sequence. Systematic Biology 57: 920-938.
