# Supplementary material for: Successful Recovery of Nuclear Protein-Coding Genes from Small Insects in Museums Using Illumina Sequencing
Source: PLoS One. 2015 Dec 30;10(12):e0143929. doi: 10.1371/journal.pone.0143929 (PMC4696846; doi:10.1371/journal.pone.0143929)
Supplement: S8 Table — (DOCX) [file pone.0143929.s019.docx]

**S8 Table. Proportion of recovered bases from 67-gene set: reference-based assemblies**

| **Gene region** | **Length** |  | **subf** | **snt1** | **Lchi** | **lach** | **Bdrs** | **ori1** | **inu1** | **lapp** | **aric** | **dspt** | **mus** | **inu2** | **ori2** | **snt2** | ***Average*** |
| --- | --- | --- | --- | --- | --- | --- | --- | --- | --- | --- | --- | --- | --- | --- | --- | --- | --- |
| 8018fin1_2 | **338** |  | 0.00 | 0.00 | 0.77 | 0.00 | 0.32 | 0.33 | 0.00 | 0.49 | 0.00 | 0.57 | 0.00 | 0.29 | 0.57 | 1.00 | *0.31* |
| 3044fin1_2 | **542** |  | 0.00 | 0.00 | 0.00 | 0.00 | 0.16 | 0.00 | 0.00 | 0.41 | 0.66 | 0.36 | 0.28 | 0.64 | 0.49 | 0.71 | *0.26* |
| 8028fin1_2 | **578** |  | 0.00 | 0.00 | 0.00 | 0.73 | 0.00 | 0.27 | 0.00 | 0.70 | 0.40 | 0.56 | 0.29 | 0.27 | 0.90 | 0.94 | *0.36* |
| 197fin1_2 | **428** |  | 0.00 | 0.58 | 0.00 | 0.45 | 0.00 | 0.42 | 0.00 | 0.00 | 0.03 | 0.24 | 0.00 | 0.00 | 0.79 | 1.00 | *0.25* |
| 36fin1_2 | **545** |  | 0.15 | 0.28 | 0.42 | 0.41 | 0.00 | 0.54 | 0.35 | 0.00 | 0.55 | 0.22 | 0.53 | 0.56 | 0.59 | 0.69 | *0.38* |
| aspec2_6 | **476** |  | 0.00 | 0.58 | 0.54 | 0.00 | 0.00 | 0.00 | 0.00 | 0.00 | 0.46 | 0.30 | 0.30 | 0.80 | 0.00 | 1.00 | *0.28* |
| 63fin2_3 | **977** |  | 0.00 | 0.26 | 0.18 | 0.47 | 0.00 | 0.86 | 0.36 | 0.55 | 0.43 | 0.37 | 0.50 | 0.82 | 0.85 | 0.85 | *0.47* |
| acc2_4 | **539** |  | 0.00 | 0.19 | 0.00 | 0.83 | 0.00 | 0.73 | 0.00 | 0.58 | 0.19 | 0.39 | 0.00 | 0.80 | 0.85 | 1.00 | *0.40* |
| 25fin2_4 | **488** |  | 0.00 | 0.57 | 0.68 | 0.41 | 0.27 | 0.80 | 0.00 | 0.55 | 0.00 | 0.35 | 0.64 | 0.98 | 0.95 | 0.98 | *0.51* |
| 270fin2_3 | **494** |  | 0.00 | 0.93 | 0.00 | 0.72 | 0.00 | 0.99 | 0.00 | 0.56 | 0.22 | 0.22 | 0.21 | 0.77 | 0.83 | 1.00 | *0.46* |
| 42fin1_2 | **494** |  | 0.00 | 0.44 | 0.43 | 0.39 | 0.11 | 0.48 | 0.00 | 0.26 | 0.60 | 0.40 | 0.37 | 0.63 | 0.60 | 0.79 | *0.39* |
| 44fin2_3 | **899** |  | 0.03 | 0.00 | 0.28 | 0.83 | 0.17 | 0.98 | 0.00 | 0.85 | 0.69 | 0.57 | 0.50 | 0.88 | 0.98 | 1.00 | *0.55* |
| 3202fin1_3 | **807** |  | 0.00 | 0.83 | 0.20 | 0.58 | 0.00 | 0.65 | 0.19 | 0.53 | 0.84 | 0.00 | 0.19 | 0.68 | 0.80 | 0.90 | *0.46* |
| 8029fin6_7 | **659** |  | 0.00 | 0.58 | 0.76 | 0.69 | 0.00 | 0.98 | 0.00 | 0.39 | 0.51 | 0.00 | 0.21 | 0.77 | 0.86 | 0.99 | *0.48* |
| 3006fin1_2 | **512** |  | 0.00 | 0.38 | 0.51 | 0.82 | 0.00 | 0.83 | 0.00 | 0.79 | 0.94 | 0.00 | 0.37 | 0.92 | 1.00 | 1.00 | *0.54* |
| 96fin1_3 | **644** |  | 0.00 | 0.44 | 0.28 | 0.76 | 0.00 | 0.68 | 0.00 | 0.31 | 0.63 | 0.20 | 0.45 | 0.62 | 0.93 | 1.00 | *0.45* |
| 262fin1_2 | **561** |  | 0.21 | 0.70 | 0.41 | 0.82 | 0.16 | 0.88 | 0.00 | 0.18 | 0.62 | 0.17 | 0.54 | 0.85 | 0.79 | 1.00 | *0.52* |
| 3007fin1_2 | **1010** |  | 0.14 | 0.70 | 0.15 | 0.54 | 0.00 | 0.88 | 0.18 | 0.20 | 0.30 | 0.00 | 0.77 | 0.84 | 0.95 | 0.98 | *0.47* |
| 3152fin1_2 | **797** |  | 0.00 | 0.36 | 0.65 | 0.79 | 0.00 | 0.74 | 0.00 | 0.32 | 0.66 | 0.32 | 0.00 | 0.78 | 0.65 | 0.79 | *0.43* |
| 8070fin1_3 | **476** |  | 0.03 | 0.39 | 0.24 | 0.62 | 0.00 | 0.61 | 0.00 | 0.17 | 0.69 | 0.74 | 0.20 | 0.23 | 0.77 | 1.00 | *0.41* |
| 3012fin1_2 | **623** |  | 0.00 | 0.38 | 0.00 | 0.29 | 0.00 | 0.73 | 0.18 | 0.65 | 0.44 | 0.44 | 0.31 | 0.70 | 0.82 | 1.00 | *0.42* |
| 40fin2_3 | **566** |  | 0.00 | 0.88 | 0.16 | 0.73 | 0.13 | 0.89 | 0.00 | 0.22 | 0.52 | 0.00 | 0.00 | 0.50 | 0.98 | 1.00 | *0.43* |
| 3136fin1_2 | **579** |  | 0.02 | 0.63 | 0.63 | 0.98 | 0.11 | 0.87 | 0.18 | 0.80 | 0.89 | 0.56 | 0.80 | 0.86 | 0.99 | 1.00 | *0.67* |
| 113fin1_2 | **374** |  | 0.00 | 0.52 | 0.43 | 0.84 | 0.00 | 0.79 | 0.00 | 0.51 | 0.73 | 0.24 | 0.36 | 0.52 | 0.81 | 0.98 | *0.48* |
| 58fin7_9 | **2306** |  | 0.00 | 0.00 | 0.40 | 0.51 | 0.27 | 0.72 | 0.00 | 0.41 | 0.78 | 0.24 | 0.56 | 0.71 | 0.87 | 0.89 | *0.45* |
| 3064fin6_7 | **632** |  | 0.39 | 0.30 | 0.55 | 0.77 | 0.00 | 0.40 | 0.17 | 0.60 | 0.68 | 0.38 | 0.00 | 0.91 | 0.62 | 1.00 | *0.48* |
| 3196fin5_6 | **701** |  | 0.13 | 0.34 | 0.64 | 0.67 | 0.21 | 0.72 | 0.13 | 0.27 | 0.64 | 0.39 | 0.69 | 0.60 | 0.91 | 0.96 | *0.52* |
| 69fin2_3 | **581** |  | 0.03 | 0.38 | 0.84 | 0.65 | 0.15 | 0.86 | 0.15 | 0.17 | 0.15 | 0.41 | 0.56 | 0.80 | 0.87 | 0.99 | *0.50* |
| 58fin3_6 | **1178** |  | 0.00 | 0.41 | 0.51 | 0.79 | 0.00 | 0.74 | 0.00 | 0.00 | 0.77 | 0.25 | 0.10 | 0.91 | 0.92 | 1.00 | *0.46* |
| 62fin2_3 | **459** |  | 0.13 | 0.59 | 0.37 | 0.89 | 0.00 | 0.91 | 0.17 | 0.41 | 0.74 | 0.51 | 0.68 | 0.85 | 0.85 | 1.00 | *0.58* |
| 8053fin2_3 | **668** |  | 0.00 | 0.52 | 0.78 | 0.65 | 0.00 | 0.79 | 0.00 | 0.26 | 0.59 | 0.59 | 0.67 | 0.95 | 0.86 | 0.93 | *0.54* |
| 247fin1_2 | **413** |  | 0.00 | 0.41 | 0.70 | 0.83 | 0.00 | 0.88 | 0.21 | 0.26 | 0.26 | 0.46 | 0.37 | 0.83 | 0.99 | 1.00 | *0.52* |
| 3114fin1_2 | **365** |  | 0.27 | 0.24 | 0.72 | 0.86 | 0.00 | 0.84 | 0.00 | 0.70 | 0.70 | 0.61 | 0.41 | 0.52 | 0.82 | 0.97 | *0.55* |
| 265fin2_3 | **782** |  | 0.00 | 0.50 | 0.00 | 0.70 | 0.22 | 0.95 | 0.00 | 0.74 | 0.54 | 0.20 | 0.81 | 0.69 | 0.94 | 0.97 | *0.52* |
| 3121fin1_2 | **1322** |  | 0.00 | 0.35 | 0.50 | 0.66 | 0.23 | 0.62 | 0.18 | 0.77 | 0.64 | 0.51 | 0.24 | 0.83 | 0.94 | 0.99 | *0.53* |
| 192fin1_2 | **362** |  | 0.00 | 0.23 | 0.65 | 0.83 | 0.00 | 0.70 | 0.30 | 0.18 | 0.68 | 0.71 | 0.22 | 0.95 | 0.95 | 0.95 | *0.52* |
| 3094fin2_3 | **377** |  | 0.27 | 0.00 | 0.00 | 0.87 | 0.22 | 0.72 | 0.21 | 0.37 | 0.78 | 0.36 | 0.48 | 0.66 | 0.93 | 1.00 | *0.49* |
| 8091fin1_2 | **263** |  | 0.00 | 0.49 | 0.43 | 0.96 | 0.00 | 0.93 | 0.00 | 0.52 | 0.49 | 0.58 | 0.62 | 0.96 | 0.91 | 0.98 | *0.56* |
| 166fin2_3 | **968** |  | 0.00 | 0.52 | 0.64 | 0.60 | 0.00 | 0.96 | 0.53 | 0.30 | 0.63 | 0.39 | 0.74 | 0.86 | 0.99 | 1.00 | *0.58* |
| 3017fin1_2 | **803** |  | 0.00 | 0.80 | 0.70 | 0.83 | 0.00 | 0.90 | 0.32 | 0.38 | 0.51 | 0.89 | 0.69 | 0.72 | 0.90 | 0.99 | *0.62* |
| 149fin2_3 | **887** |  | 0.09 | 0.75 | 0.16 | 0.88 | 0.00 | 0.65 | 0.00 | 0.65 | 0.40 | 0.25 | 0.36 | 0.73 | 0.93 | 1.00 | *0.49* |
| aspec11_12 | **704** |  | 0.00 | 0.68 | 0.72 | 0.18 | 0.00 | 0.69 | 0.00 | 0.35 | 0.62 | 0.22 | 0.35 | 0.72 | 0.68 | 0.97 | *0.44* |
| 220fin1_2 | **407** |  | 0.00 | 0.20 | 0.86 | 0.92 | 0.16 | 0.77 | 0.00 | 0.51 | 0.80 | 0.37 | 0.39 | 0.91 | 0.99 | 1.00 | *0.56* |
| 3031fin1_3 | **890** |  | 0.19 | 0.60 | 0.70 | 0.80 | 0.13 | 0.94 | 0.15 | 0.52 | 0.68 | 0.60 | 0.77 | 0.99 | 1.00 | 1.00 | *0.65* |
| 3066fin1_3 | **348** |  | 0.00 | 0.48 | 0.75 | 0.90 | 0.18 | 0.87 | 0.08 | 0.47 | 0.99 | 0.25 | 0.68 | 0.96 | 1.00 | 1.00 | *0.61* |
| EF-1α | **572** |  | 0.18 | 1.00 | 0.88 | 0.91 | 0.57 | 0.98 | 0.31 | 0.92 | 0.97 | 0.66 | 0.74 | 0.91 | 0.98 | 1.00 | *0.79* |
| 2F3_4 | **683** |  | 0.00 | 0.69 | 0.73 | 0.80 | 0.00 | 0.81 | 0.17 | 0.47 | 0.37 | 0.35 | 0.47 | 0.74 | 0.92 | 0.96 | *0.53* |
| 268fin1_2 | **656** |  | 0.02 | 0.59 | 0.74 | 0.85 | 0.00 | 0.81 | 0.02 | 0.29 | 0.80 | 0.45 | 0.75 | 0.88 | 0.92 | 0.99 | *0.58* |
| aspec19_21 | **440** |  | 0.23 | 0.31 | 0.69 | 0.90 | 0.00 | 0.75 | 0.40 | 0.69 | 0.81 | 0.20 | 0.70 | 0.76 | 0.86 | 0.97 | *0.59* |
| 3153fin1_2 | **554** |  | 0.00 | 0.62 | 0.81 | 0.78 | 0.00 | 0.70 | 0.16 | 0.65 | 0.66 | 0.29 | 0.47 | 0.87 | 0.53 | 1.00 | *0.54* |
| 274fin1_2 | **581** |  | 0.00 | 0.49 | 0.79 | 0.84 | 0.00 | 0.68 | 0.00 | 0.33 | 0.47 | 0.39 | 0.64 | 0.78 | 0.81 | 1.00 | *0.51* |
| 109fin1_2 | **506** |  | 0.00 | 0.84 | 0.44 | 0.43 | 0.00 | 0.85 | 0.21 | 0.86 | 0.42 | 0.95 | 0.56 | 1.00 | 0.94 | 1.00 | *0.61* |
| 3196fin1_3 | **317** |  | 0.00 | 0.52 | 0.85 | 0.77 | 0.17 | 0.93 | 0.23 | 0.65 | 0.59 | 0.00 | 0.50 | 0.74 | 0.98 | 1.00 | *0.57* |
| 3070fin4_5 | **281** |  | 0.13 | 0.59 | 0.22 | 0.86 | 0.00 | 0.57 | 0.00 | 0.15 | 0.34 | 0.51 | 0.00 | 0.93 | 0.61 | 1.00 | *0.42* |
| 3031fin4_5 | **749** |  | 0.00 | 0.78 | 1.00 | 0.95 | 0.00 | 1.00 | 0.44 | 0.89 | 0.82 | 0.86 | 0.90 | 0.99 | 1.00 | 1.00 | *0.76* |
| 73fin2_3 | **572** |  | 0.00 | 0.12 | 0.29 | 0.95 | 0.00 | 0.94 | 0.14 | 0.75 | 0.96 | 0.72 | 0.34 | 0.98 | 0.98 | 1.00 | *0.58* |
| EF-2 | **626** |  | 0.07 | 0.98 | 0.63 | 0.89 | 0.15 | 1.00 | 0.20 | 0.78 | 0.93 | 0.46 | 0.50 | 0.95 | 0.98 | 1.00 | *0.68* |
| 127fin1_2 | **590** |  | 0.00 | 0.74 | 0.44 | 0.90 | 0.00 | 1.00 | 0.20 | 0.74 | 0.64 | 0.11 | 0.58 | 0.97 | 0.95 | 1.00 | *0.59* |
| 6fin2_3 | **779** |  | 0.00 | 0.56 | 0.61 | 0.83 | 0.00 | 0.83 | 0.51 | 0.97 | 0.90 | 0.46 | 0.36 | 0.82 | 0.81 | 1.00 | *0.62* |
| 2F7_8 | **632** |  | 0.00 | 0.47 | 0.38 | 0.85 | 0.33 | 0.95 | 0.15 | 0.51 | 0.68 | 0.52 | 0.68 | 0.77 | 0.92 | 1.00 | *0.59* |
| 26fin3_4 | **333** |  | 0.00 | 0.51 | 0.99 | 0.84 | 0.28 | 1.00 | 0.31 | 0.56 | 0.68 | 0.49 | 0.68 | 0.96 | 0.96 | 1.00 | *0.66* |
| 3009fin2_3 | **752** |  | 0.04 | 0.39 | 0.65 | 0.85 | 0.46 | 0.87 | 0.00 | 0.67 | 0.00 | 0.86 | 0.29 | 0.95 | 0.99 | 1.00 | *0.57* |
| 226fin1_2 | **500** |  | 0.17 | 0.67 | 0.65 | 0.70 | 0.00 | 0.77 | 0.16 | 0.64 | 0.99 | 0.68 | 0.59 | 0.97 | 0.98 | 1.00 | *0.64* |
| PolII | **381** |  | 0.00 | 0.45 | 0.74 | 0.97 | 0.18 | 0.98 | 0.32 | 0.71 | 0.97 | 0.47 | 0.65 | 1.00 | 1.00 | 1.00 | *0.67* |
| 3055fin2_3 | **771** |  | 0.36 | 0.75 | 0.90 | 0.87 | 0.00 | 0.94 | 0.00 | 0.75 | 0.86 | 0.54 | 0.58 | 0.93 | 1.00 | 1.00 | *0.68* |
| 3059fin1_3 | **347** |  | 0.00 | 0.56 | 0.83 | 0.92 | 0.15 | 0.97 | 0.16 | 0.79 | 0.98 | 0.40 | 0.94 | 0.87 | 0.99 | 1.00 | *0.68* |
| 3089fin1_3 | **461** |  | 0.00 | 0.86 | 0.86 | 0.74 | 0.00 | 0.93 | 0.63 | 0.99 | 0.88 | 0.97 | 0.71 | 0.97 | 0.95 | 1.00 | *0.75* |
|  |  |  |  |  |  |  |  |  |  |  |  |  |  |  |  |  |  |
| ***Average*** |  |  | *0.05* | *0.49* | *0.51* | *0.71* | *0.08* | *0.77* | *0.13* | *0.50* | *0.61* | *0.41* | *0.46* | *0.78* | *0.86* | *0.97* | *0.53* |
| ***Avg. RS*** |  |  |  |  |  |  |  |  |  |  |  |  |  |  | *0.86* | *0.97* | *0.92* |
| ***Avg. MS*** |  |  | *0.05* | *0.49* | *0.51* | *0.71* | *0.08* | *0.77* | *0.13* | *0.50* | *0.61* | *0.41* | *0.46* | *0.78* |  |  | *0.46* |
| ***Avg. MS - LR*** |  |  |  | *0.49* | *0.51* | *0.71* |  | *0.77* |  | *0.50* | *0.61* |  | *0.46* | *0.78* |  |  | *0.61* |

**Gene region**: abbreviation used in [1], for 67 target genes. **Length**: length of fragment in *Bembidion* sp. nr. *transversale* DNA3205. **Lag**: Lagriinae n. gen. KK0290. **subf**: *Bembidion subfusum* 3977. **snt1**: *Bembidion* sp. nr. *transversale* 3021. **Lchi**: *Lionepha chintimini* 4002. **lach**: *Bembidion lachnophoroides* 3022. **Bdrs**: *Bembidarenas* 3983. **ori1**: *Bembidion orion* 2831. **inu1**: *Bembidion* "Inuvik"3285. **lapp**. *Bembidion lapponicum* 3974. **aric**: *Bembidion* "Arica" 3242. **dspt**: *Bembidion* *cf*. "Desert Spotted" 3978. **mus**: *Bembidion musae* 3239. **inu2**: Bembidion "Inuvik" 3984. **ori2**: *Bembidion orion* 3079. **snt2** *Bembidion* sp. nr. *transversale* 3205. **Avg.**: average proportion of bases recovered for all museum specimens and both reference specimens. **Avg. RS**: average proportion of bases recovered for the two reference specimens. **Avg. MS**: average proportion of bases recovered for all museum specimens. **Avg. MS - LR**: average proportion of bases recovered for museum specimens, excluding the 4 museum specimens with less than 34 million reads.

1. Regier JC, Shultz JW, Ganley ARD, Hussey A, Shi D, et al. (2008) Resolving Arthropod Phylogeny: Exploring Phylogenetic Signal within 41 kb of Protein-Coding Nuclear Gene Sequence. Systematic Biology 57: 920-938.
