# Supplementary material for: Successful Recovery of Nuclear Protein-Coding Genes from Small Insects in Museums Using Illumina Sequencing
Source: PLoS One. 2015 Dec 30;10(12):e0143929. doi: 10.1371/journal.pone.0143929 (PMC4696846; doi:10.1371/journal.pone.0143929)
Supplement: S9 Table — (DOCX) [file pone.0143929.s020.docx]

**S9 Table. Data used in regression study.**

| **Taxon** | **Sample** | **Age** | **Length** | **Killing** | **DNA** | **DNA Qual** | **Modal Frag** | **Reads** | **PCR 28S** | **PCR COI** | **PCR Any** | **DN50** | **DN80** | **Ref50** | **Ref80** |
| --- | --- | --- | --- | --- | --- | --- | --- | --- | --- | --- | --- | --- | --- | --- | --- |
| Lagriinae n. gen. | KK0290 | 84 | 9.7 | 1 | 1700 | 1 | 60 | 60 | 0 | 0 | 0 | 21 | 3 |  |  |
| *B. subfusum* | 3977 | 69 | 4.4 | 1 | 41.7 | 1 | 60 | 24.7 | 0 | 0 | 0 | 0 | 0 | 0 | 0 |
| *B.* sp. nr. *transversale* | 3021 | 60 | 6.9 | 1 | 164 | 2 | 120 | 62.7 | 0 | 0 | 0 | 9 | 0 | 52 | 10 |
| *Lionepha chintimini* | 4002 | 58 | 4.3 | 0 | 246 | 3 | 220 | 71.9 | 0 | 0 | 1 | 49 | 21 | 58 | 15 |
| *B. lachnophoroides* | 3022 | 56 | 4.4 | 2 | 9.9 | 1 | 80 | 63.7 | 0 | 0 | 1 | 72 | 34 | 84 | 49 |
| *Bembidarenas* | 3983 | 52 | 3.8 | 1 | 53.2 | 1 | 100 | 22.8 | 0 | 0 | 0 | 0 | 0 | 1 | 0 |
| *B. orion* | 2831 | 43 | 3.5 | 1 | 83.9 | 3 | 200 | 64.8 | 0 | 0 | 0 | 72 | 43 | 90 | 55 |
| *B.* "Inuvik" | 3285 | 32 | 5 | 0 | 168 | 2 | 50 | 33.9 | 0 | 0 | 0 | 0 | 0 | 4 | 0 |
| *B. lapponicum* | 3974 | 32 | 6.5 | 0 | 749 | 4 | 250 | 76.5 | 0 | 0 | 1 | 19 | 3 | 55 | 10 |
| *B.* "Arica" | 3242 | 20 | 4.3 | 1 | 539 | 4 | 150 | 70.2 | 1 | 1 | 1 | 72 | 37 | 72 | 24 |
| *B. cf.* "Desert Spotted" | 3978 | 10 | 4.8 | 0 | 412 | 4 | 260 | 26.8 | 1 | 0 | 1 | 1 | 1 | 34 | 7 |
| *B. musae* | 3239 | 9 | 6.4 | 1 | 3880 | 5 | 500 | 75.6 | 0 | 0 | 1 | 24 | 7 | 51 | 6 |
| *B.* "Inuvik" | 3984 | 4 | 5 | 2 | 3300 | 5 | 10000 | 71.4 | 1 | 1 | 1 | 64 | 34 | 93 | 58 |

**Age**: year from time of death to DNA extraction**. Length**: length of specimen in mm. **Killing**: substance used to kill beetle, 0: not ethanol, 1: probably 70-75% ethanol, 2: probably 95% ethanol. **DNA Qual**: DNA quality score as defined in *Assessing DNA quality of museum and reference specimens* section of *Methods*. **Modal Frag**: modal fragment length of DNA extraction determined by bioanalysis. **Reads**: number of reads in millions. **PCR 28S**: success or not of 28S PCR, 0: failure, 1: success. **PCR COI** success or not of COI PCR, 0: failure, 1: success. **PCR Any**: success or not of any of four genes for which PCR was attempted, 0: failure, 1: success. **DN50**, **DN80, Ref50**, and **Ref80**: see Table 12 for description.
