# Supplementary material for: Successful Recovery of Nuclear Protein-Coding Genes from Small Insects in Museums Using Illumina Sequencing
Source: PLoS One. 2015 Dec 30;10(12):e0143929. doi: 10.1371/journal.pone.0143929 (PMC4696846; doi:10.1371/journal.pone.0143929)
Supplement: S10 Table — (DOCX) [file pone.0143929.s021.docx]

**S10 Table: Gene fragments sampled for the phylogenetic analyses of Lagriinae.**

| **Taxon** | **Group** | **18S** | **28S** | **COI** | **ArgK** | **CAD** | ***wg*** |
| --- | --- | --- | --- | --- | --- | --- | --- |
| *Adelium*^1^ | Adeliini | KP418989 | KP419340 | KJ364028 | KP812161 | KP812736 | KP813319 |
| *Cardiothorax* | Adeliini | - | 316 | - | 316 | 316 | - |
| *Zeadelium gratiosum* | Adeliini | - | 228 | 228 | 228 | 228 | 228 |
| *Adelonia* | Belopini | 099 | 099 | - | 099 | 099 | 099 |
| *Rhypasma* | Belopini | - | 312 | - | 312 | 312 | 312 |
| *Cossyphus* | Cossyphini | 100 | 100 | - | 100 | 100 | 100 |
| *Chaerodes trachyscelides* | Chaerodini | 163 | 163 | 163 | - | 163 | 163 |
| *Anaedus brunneus* | Goniaderini | - | 128 | - | 128 | 128 | 128 |
| *Anaedus leleupi* | Goniaderini | - | - | 314 | 314 | 314 | 314 |
| *Goniadera* | Goniaderini | 342 | 342 | 342 | 342 | 342 | 342 |
| *Phymatestes* | Goniaderini | 046 | 046 | - | 046 | 046 | 046 |
| *Pseudolyprops* | Goniaderini | - | 245 | - | 245 | 245 | 245 |
| *Laena dilutella* | Laenini | 340 | 340 | 340 | 340 | 340/341^2^ | 340 |
| *Laena franzi* | Laenini | - | 313 | - | - | - | 313 |
| *Mimolaena clarissae* | Laenini | - | 324 | - | 324 | - | 324 |
| *Lagria* | Lagriini | 057 | 057 | 057 | 057 | 057 | 057 |
| *Phobelius* | Lagriini | - | - | - | - | - | 288 |
| *Statira* pluripunctatus^1^ | Lagriini | KP419299 | 055 | - | 055 | 055 | 055 |
| *Chaetyllus* n. sp. 5 | Predicted clade | - | 276 | - | - | 276 | 276 |
| *Lagriinae* n. gen.^3^ | Predicted clade | 290 | 290 | 290 | 290 | 290 | - |
| *Lagriinae* n. gen. 2 | Predicted clade | 148 | 148 | 148 | 148 | 148 | 148 |
| *Antennoluprops bremeri* | Lupropini | - | 321 | - | - | - | 321 |
| *Enicmosoma decorsei* | Lupropini | - | 235 | - | 235 | 235 | 235 |
| *Lorelus* | Lupropini | 168 | 168 | 168 | 168 | 168 | 168 |
| *Paratenetus punctatus* | Goniaderini | - | 075 | - | 075 | 075 | 075 |
| *Prateus fusculus* | Goniaderini | - | 295 | - | 295 | - | 295 |
| *Coxelinus* | Lupropini | - | - | - | 315 | 315 | 315 |
| *Luprops* | Lupropini | 082 | 082 | 082 | 082 | 082 | 082 |
| *Metallonotus* | Pycnocerini | - | 224 | - | 224 | 224 | - |
| *Odontopezus* | Pycnocerini | 042 | - | 042 | 042 | 042 | 042 |
| *Clamoris americana* | Out Group | - | 065 | 065 | 065 | 065 | 065 |
| *Eupsophulus castaneus* | Out Group | 018 | 018 | 018 | 018 | 018 | 018 |
| *Pechalius dentiger* | Out Group | 016 | 016 | - | 016 | 016 | 016 |
| *Strongylium atrum* | Out Group | - | 039 | - | 039 | 039 | 039 |
| *Tanylypa morio* | Out Group | KP419309 | KP419661 | - | KP812437 | KP813037 | KP813598 |
| *Tenebrio molitor^1^* | Out Group | X07801 | 266 | HQ559248 | 266 | 266 | 266 |
| *Tribolium castaneum^1^* | Out Group | HM156711 | EU677678 | KC139706 | XM966707 | EU677538 | XM962887 |

**Group**: Outgroups are indicated as such; for ingroup taxa, the current tribal assignment is given except for Lagriinae n. gen. and the two taxa with which it is expected to form a clade. *Chaetyllus* n. sp. 5 is currently assigned to the Goniaderini, but based upon examination of female internal genital characters, this placement is incorrect. Numbers in the gene columns indicate either KK DNA voucher IDs of newly sequenced specimens or GenBank accession codes for previously published sequences. The GenBank sequences come from [1-6].

^1^ Chimeric taxa from multiple specimens. *Adelium* may be a chimera of two species. The rest are chimeras of different specimens of the same species.

^2^ The CAD sequence for *Laena dilutela* is a chimera of sequences from extractions of two individuals collected at the same locality. The first region of CAD was sequenced from KK0341 and the second region of CAD was sequenced from KK0340.

^3^ Sequence data for Lagriinae n. gen. were recovered from BLAST searches of the *de novo* assembly. All other sequences were recovered using PCR and Sanger sequencing.

1. McKenna DD, Wild AL, Kanda K, Bellamy CL, Beutel RG, et al. (2015) The beetle tree of life reveals that Coleoptera survived end-Permian mass extinction to diversify during the Cretaceous terrestrial revolution. Systematic Entomology 40: 835-880.

2. Endo Y, Nash M, Hoffmann AA, Slatyer R, Miller AD (2015) Comparative phylogeography of alpine invertebrates indicates deep lineage diversification and historical refugia in the Australian Alps. Journal of Biogeography 42: 89-102.

3. Hendriks L, Broeckhoven C, Vandenberghe A, Van de Peer Y, De Wachiter R (1988) Primary and secondary structure of the 18S ribosomal RNA of the bird spider Eurypelma californica and evolutionary relationships among eukaryotic phyla. European Journal of Biochemistry 177: 15-20.

4. McKenna DD, Farrell BD (2010) 9-genes reinforce the phylogeny of holometabola and yield alternate views on the phylogenetic placement of Strepsiptera. PloS one 5: e11887.

5. Pentinsaari M, Hebert PD, Mutanen M (2014) Barcoding beetles: A regional survey of 1872 species reveals high identification success and unusually deep interspecific divergences.

6. Wild AL, Maddison DR (2008) Evaluating nuclear protein-coding genes for phylogenetic utility in beetles. Molecular Phylogenetics and Evolution 48: 877-891.
