# Supplementary material for: Successful Recovery of Nuclear Protein-Coding Genes from Small Insects in Museums Using Illumina Sequencing
Source: PLoS One. 2015 Dec 30;10(12):e0143929. doi: 10.1371/journal.pone.0143929 (PMC4696846; doi:10.1371/journal.pone.0143929)
Supplement: S11 Table — (DOCX) [file pone.0143929.s022.docx]

**S11 Table. Ethanol-killed specimens sequenced using PCR amplification and Sanger sequencing.**

| **Species** | **Sample** | **Locality** | **Genes sequenced** |
| --- | --- | --- | --- |
| *Bembidarenas reicheellum* #2 | 1450 | Argentina: Santa Cruz District, Intersection of Route 15 & Rio Centnela. 50.415°S, 72.512°W | 6 genes (no Topo) |
| *Lionepha chintimini* | 4059 | USA: Alaska: Thompson Pass el. 796 m, 61.13731°N 145.74487°W | 6 genes (no 18S) |
| *Bembidion* "Desert Spotted" | 2786 | Mexico: Baja California Norte, San Pedro Martir, Aguaje del Burro. 30.99580N, 115.46638W | 6 genes (no 18S) |
| *Bembidion approximatum* | 2141 | USA: California: Marin Co., Nicasio Reservoir | 7 genes |
| *Bembidion* sp.nr. *transversale* | 2162 | Canada: British Columbia: Hope, Fraser River near mouth of Coquihalla River, 49.3961°N 121.4351°W | 28S+COI+CAD+Topo |
| *Bembidion* sp.nr. *transversale* | 2165 | USA: Washington: Whatcom Co., Nooksack River 1.4 mi S of Deming, 70m, 48.8080°N 122.2019°W | 28S+COI+CAD+Topo |
| *Bembidion* sp.nr. *transversale* | 2175 | USA: California: Mono Co., Leavitt Creek near Leavitt Meadows, 2180m, 38.3280°N 119.5496°W | 28S+COI+CAD+Topo |
| *Bembidion* sp.nr. *transversale* | 2176 | USA: California: Alpine Co., Frog Lake near Carson Pass, 2700m, 38.6877°N 119.9860°W | 28S+COI+CAD+Topo |
| *Bembidion* sp.nr. *transversale* | 2179 | USA: California: Alpine Co., Frog Lake near Carson Pass, 2700m, 38.6877°N 119.9860°W | 28S+COI+CAD+Topo |
| *Bembidion* sp.nr. *transversale* | 2180 | USA: California: Sonoma Co., Russian River, 3 mi NE Heraldsburg | 28S+COI+CAD+Topo |
| *Bembidion* sp.nr. *transversale* | 2181 | USA: California: Marin Co., Nicasio Reservoir, 70m, 38.088°N 122.7383°W | 28S+COI+CAD+Topo |
| *Bembidion* sp.nr. *transversale* | 2194 | Canada: British Columbia: Hope, Fraser River near mouth of Coquihalla River, 49.3961°N 121.4351°W | 28S+COI+CAD+Topo |
| *Bembidion* sp.nr. *transversale* | 2973 | USA: Oregon: Benton Co., Corvallis, Willamette River, 60m, 44.5491°N 123.2451°W | 28S+COI+CAD+Topo |
| *Bembidion* sp.nr. *transversale* | 3772 | USA: California: Tehama Co., Red Bluff, Sacramento River, 73m, 40.1759°N 122.2290°W | 28S+COI+CAD+Topo |
| *Bembidion* sp.nr. *transversale* | 3205 | USA: Oregon: Benton Co., Corvallis, Willamette River, 60m, 44.5491°N 123.2451°W | 28S+COI+CAD+Topo |
| *Bembidion* sp.nr. *transversale* | 3559 | USA: California: Del Norte Co., Wilson Creek , 3m, 41.6051°N 124.1005°W | 28S+COI+CAD+Topo |
| *Bembidion* sp.nr. *transversale* | 3560 | USA: California: Del Norte Co., Wilson Creek , 3m, 41.6051°N 124.1005°W | 28S+COI+CAD+Topo |
| *Bembidion* sp.nr. *transversale* | 4032 | USA: Oregon: Coos Co., Crooked Creek S of Bandon, 7m, 43.0814°N 124.4335°W | 28S+COI+CAD+Topo |
| *Bembidion* sp.nr. *transversale* | 4034 | USA: Oregon: Curry Co., Floras Creek at route 124 SE Langlois, 21m, 42.9132°N 124.4251°W | 28S+COI+CAD+Topo |
| *Bembidion* sp.nr. *transversale* | 4052 | USA: California: Monterey Co., Big Sur River, Andrew Molera St Pk, 7m, 36.2850°N 121.8544°W | 28S+COI+CAD+Topo |
| *Bembidion* sp.nr. *transversale* | 4054 | USA: California: San Luis Obispo Co., Pismo State Beach, 4m, 35.0999°N 120.6267°W | 28S+COI+CAD+Topo |
| *Bembidion transversale* | 2486 | USA: Colorado: Fremont Co., Arkansas River at Texas Creek, 1880m, 38.4106°N 105.5844°W | CAD+Topo |
| *Bembidion transversale* | 2160 | Canada: Nova Scotia: Hantsport, Halfway River, 45.0487°N 64.1835°W | CAD+Topo |
| *Bembidion sarpedon* | 2514 | USA: Colorado: Huerfano Co., Huerfano River at Badito, 1960m, 37.7285°N 105.0167°W | 28S+COI+CAD+Topo |
| *Bembidion sarpedon* | 3009 | USA: Colorado: Huerfano Co., Muddy Creek at CR 555, 2220m, 37.8021°N 105.2534°W | 28S+COI+CAD+Topo |
| *Bembidion sarpedon* | 3761 | USA: Utah: Iron Co., Coal Creek, 1935m, 37.6617°N 112.9967°W | 28S+COI+CAD+Topo |
| *Bembidion sarpedon* | 3776 | USA: Utah: Emery Co., Muddy Creek, 1375m, 38.5304°N 110.9029°W | 28S+COI+CAD+Topo |
| *Bembidion perspicuum* | 2182 | USA: New Mexico: Grant Co., Gila River at route 211, Gila, 1370m, 32.9691°N 108.5872°W | CAD+Topo |
| *Bembidion perspicuum* | 2318 | USA: California: San Bernardino Co., Miller Canyon, San Bernadino Mtns, 1105m, 34.2717°N 117.2892°W | CAD+Topo |
| *Bembidion perspicuum* | 2485 | USA: Colorado: Fremont Co., Arkansas River at Texas Creek, 1880m, 38.4106°N 105.5844°W | CAD+Topo |
| *Bembidion perspicuum* | 3774 | USA: California: San Benito Co., San Benito River, 740m, 36.3585°N 120.7862°W | 28S+COI+CAD+Topo |
| *Bembidion perspicuum* | 3775 | USA: California: San Benito Co., San Benito River, 740m, 36.3585°N 120.7862°W | 28S+COI+CAD+Topo |

The first four listed specimens are included in the phylogenetic studies of all *Bembidion* and other carabids; the remaining specimens are included in the phylogenetic analysis of the *Bembidion transversale* species group.
